# An Enabling Peptide Ligation Induced by Thiol‐Salicylaldehyde Ester for Chemical Protein Synthesis

**DOI:** 10.1002/advs.202408538

**Published:** 2024-10-23

**Authors:** Cuicui Li, Wenge Ma, Kang Jin

**Affiliations:** ^1^ Department of Medicinal Chemistry, Key Laboratory of Chemical Biology (Ministry of Education), School of Pharmacy, Cheeloo College of Medicine Shandong University Jinan Shandong 250012 China

**Keywords:** chemical ligation, chemical protein synthesis, peptide thiol‐salicylaldehyde esters, peptides, proteins

## Abstract

Chemical protein synthesis by amide‐forming ligation of two unprotected peptide segments offers an effective strategy for the preparation of protein derivatives that are not accessible through bioengineering approaches. Herein, an unprecedented chemical ligation between peptides with C‐terminal 2‐mercaptobenzaldehyde (thiol‐salicylaldehyde, TSAL) esters and peptides bearing N‐terminal cysteine/penicillamine is reported. Reactive peptide TSAL esters can be obtained from peptide hydrazides in an operationally simple and highly effective manner. This chemoselective peptide ligation enables the rapid production of *N,S*‐benzylidene acetal intermediates, which can readily be converted into native amide bonds even at sterically hindered junctions. In addition, the current method can be applied compatibly in concert with other types of ligations and subsequent desulfurization chemistry, thereby facilitating convergent protein synthesis. The effectiveness of this new method is also showcased by the total synthesis of proteins ubiquitin and hyalomin‐3 (Hyal‐3), the efficient synthesis of protein ubiquitin‐fold modifier 1 (UFM1) via a C‐to‐N sequential TSAL ester‐induced ligation strategy, and the chemical synthesis of protein *Mtb* CM through a combined strategy of Ser/Thr ligation and TSAL ester‐induced ligations.

## Graphical abstract

A novel and powerful peptide ligation between peptide TSAL esters and peptides are reported bearing N‐terminal cysteine/penicillamine that enables effective coupling reactions even at the most sterically hindered Pro‐Val site. This new method will bring more opportunities and valuable options for the future convergent synthesis of otherwise unobtainable proteins.

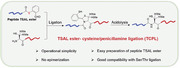

## 1 Introduction

Over the past few decades, peptide ligation chemistry has been dramatically developed and offered new opportunities for preparing proteins not accessible by expression or other bioengineering methods, such as proteins with site‐specifically post‐translational modifications, proteins with unnatural amino acids, and mirror‐image proteins.^[^ ^1^ ^]^ Chemical protein synthesis makes possible further exploration of life science and medical territory beyond traditionally recombinant technology.^[^ ^2^ ^]^ Key to the success of protein chemical synthesis is the availability of chemoselective ligation methods that can effectively conjugate unprotected peptide fragments by forming natural peptidic linkages.^[^ ^3^ ^]^ The native chemical ligation (NCL) of C‐terminal thioesters and N‐terminal cysteines, developed by Kent and co‐workers, is undoubtedly a transformative breakthrough in peptide ligation chemistry. Over the past two decades, with the development of effective strategies for preparing C‐terminal thioesters (or surrogates) and thiol‐derived amino acids, NCL has been widely used in the chemical synthesis of a variety of proteins.^[^ ^4^ ^]^ Inspired by NCL, a number of other chemoselective ligations with different mechanisms and terminal functionalities have also been developed as means of acquiring proteins, such as the Ser/Thr ligation (STL) relying on peptides with C‐terminal salicylaldehyde (SAL) esters and N‐terminal serine/threonine,^[^ ^5^ ^]^ the α‐ketoacid‐hydroxylamine (KAHA) ligation proceeding between peptides carrying C‐terminal ketoacids and N‐terminal hydroxylamines,^[^ ^6^ ^]^ the additive‐free diselenide‐selenoester ligation (DSL) utilizing peptides with C‐terminal selenoesters and N‐terminal selenocystines,^[^ ^7^ ^]^ and the cysteine/penicillamine ligation (CPL) ligating peptides bearing C‐terminal SAL esters and N‐terminal cysteine/penicillamine.^[^ ^8^ ^]^ As important complements to NCL, these methods provide more ligation disconnection sites and “orthogonal” synthetic strategies in the chemical preparation of proteins.

**Figure 1:**
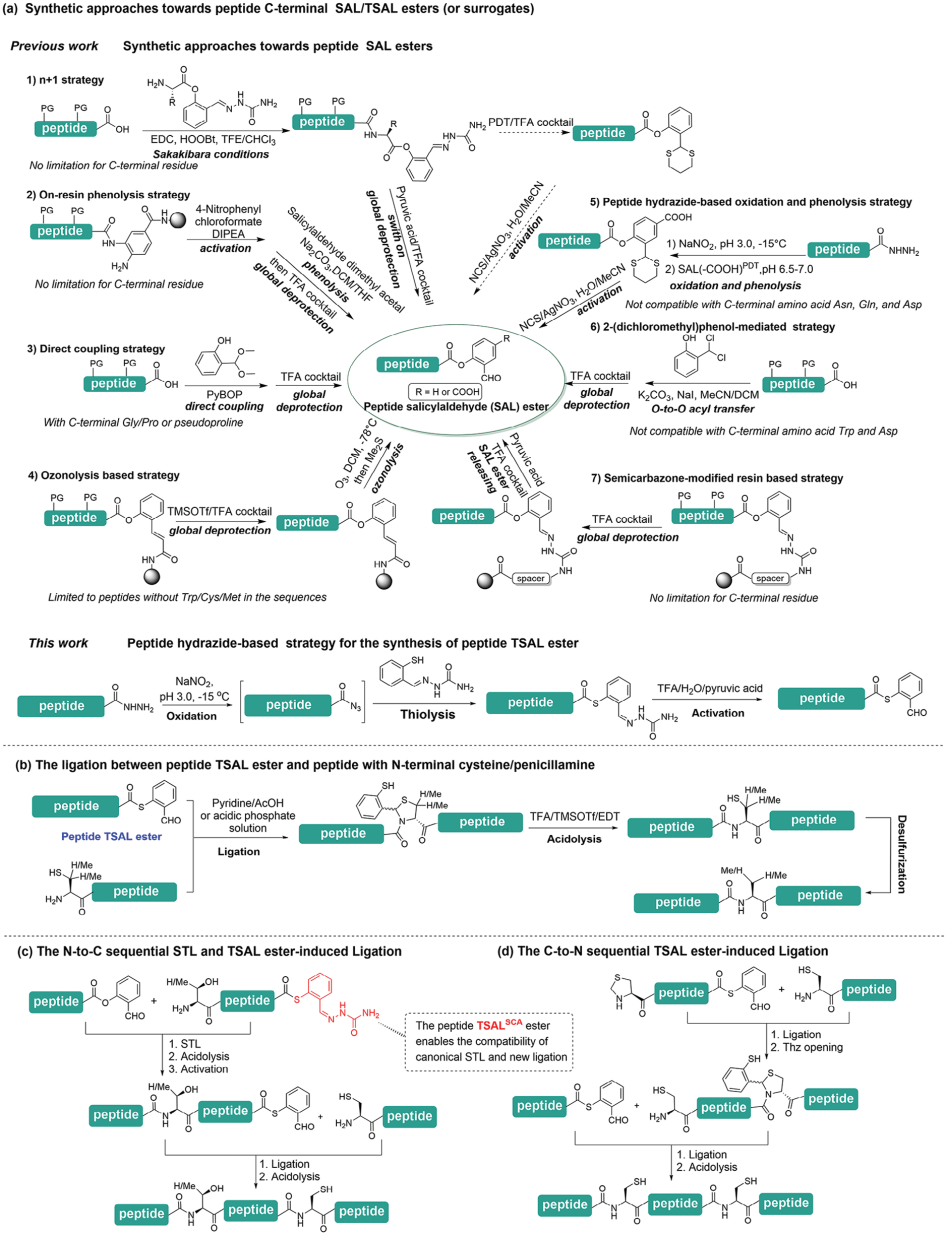
Chemoselective peptide ligation induced by TSAL ester. a) The synthetic approach toward peptide C‐terminal SAL/TSAL esters (or surrogates). b) The peptide ligation is induced by TSAL ester. c) The N‐to‐C sequential STL and TSAL ester‐induced ligation method. d) The C‐to‐N sequential TSAL ester‐induced ligation method.

However, these ligation methods also have their own limitations. For example, peptide thioesters are difficult to synthesize with Fmoc chemistry, and NCL barely proceeded with peptide thioesters carrying sterically demanding amino acids at the C‐terminus (especially proline and valine), due to the stereo‐hindrance effect of N^α^‐carbonyl and deactivating n → π^*^ interaction.^[^ ^9^ ^]^ The application of STL and CPL has always been restricted by the sophisticated syntheses of peptide SAL esters (surrogates).^[^ ^10^ ^]^ To address these shortcomings, many effective strategies have been developed, and one of the most widely used is peptide hydrazide chemistry.^[^ ^11^ ^]^ Through nitrite oxidation and thiolysis approaches, the peptide hydrazides could be readily converted to their corresponding peptide thioesters, and sequentially ligate to Cys‐peptides smoothly.^[^ ^4^, ^12^ ^]^ To date, a number of methods to prepare peptide SAL esters have been developed (**Figure** **1**a) to popularize the application of STL and CPL. For example, the on‐resin phenolysis of the N‐peptidyl benzimidazolinone (Nbz) by salicylaldehyde dimethyl acetal, direct coupling of the salicylaldehyde dimethyl acetal to the fully‐protected peptides with C‐terminal Gly/Pro or pseudoproline, the ‘n + 1’ strategy achieved by the coupling of the fully‐protected “n” peptide with the “1” unit bearing the semicarbazide‐protected salicylaldehyde (SAL^SCA^), ozonolysis/semicarbazone‐based strategy on aminomethyl (AM) resin compatible with Boc‐SPPS, and 2‐(dichloromethyl)phenol‐mediated strategy relying on *O*‐to‐*O* acyl transfer.^[^ ^5^, ^13^ ^]^ Very recently, He and co‐workers successfully prepared peptide 3‐(1,3‐dithian‐2‐yl)‐4‐hydroxybenzoic acid [SAL(‐COOH)^PDT^] esters via a peptide hydrazide‐based oxidation and phenolysis strategy, which could be activated and used for the following peptide ligations.^[^ ^14^ ^]^ Armed with these methods, some enhanced ligations have also been developed to solve the problems in the synthesis of membrane‐associated or mirror‐image proteins, such as the backbone‐installed split intein‐assisted ligation (BISIAL), the prior disulfide bond‐mediated STL and the enhanced NCL mediated by peptide conjugation in trifluoroacetic acid (TFA).^[^ ^15^ ^]^

While these advances have significantly expanded the applicable scope of corresponding ligation methods, many simultaneous issues in each method have not been solved effectively, such as the utilization of dangerously explosive and toxic reagents (e.g., AgNO_3_, smelly 1,3‐propanedithiol (PDT), etc.).^[^ ^14^ ^]^ Moreover, STL and CPL cannot be carried out when the peptide SAL esters with C‐terminal Lys/Glu/Asp due to their instability under standard ligation conditions.^[^ ^16^ ^]^ Thus, the development of novel and powerful ligation methods that can tolerate more unprotected functionalized groups and rely on easily obtained reactants, is still urgently demanding for synthetic protein chemistry.

In the present study, we describe an effective and easy‐to‐operate ligation reaction between peptides with C‐terminal 2‐mercaptobenzaldehyde (thiol‐salicylaldehyde, TSAL) esters and peptides with N‐terminal cysteine/penicillamine, that enables smooth generation of natural peptidic linkages, even at sterically hindered junctions (Figure 1b). The peptide TSAL esters can be facilely prepared from peptide hydrazides through an operationally simple strategy, and are sufficiently stable with unprotected Lys/Glu residues at the C‐terminus (Figure 1a). Notably, the peptide hydrazides can be readily obtained synthetically or recombinantly,^[^ ^17^ ^]^ which makes it possible to achieve both total and semi‐synthesis of proteins with TSAL ester‐induced ligation. In addition, the high stability of peptide TSAL ester precursor allows the combined utilization of multiple ligation strategies for convergent protein synthesis (Figure 1c,d). The validity and practicability of this new method are illustrated by the efficient synthesis of ubiquitin and Hyal‐3, C‐to‐N synthesis of UFM1, and chemical synthesis of *Mtb* CM via a combined strategy of STL and TSAL ester‐induced ligations.

## 2 Results and Discussion

### 2.1 Synthesis and Ligation of Peptide TSAL Esters

Considering that there are currently no effective synthetic means for peptide TSAL esters, our study began with the development of a simple and efficient method to prepare epimerization‐free peptides with C‐terminal TSAL esters. Direct coupling of TSAL ethylene acetal to the side‐chain protected peptides is indeed a very effective method, however, its application is always restricted to the peptides with C‐terminal Gly or Pro residues due to epimerization issues. We also explored the ‘n+1’ strategy which has been extensively used in the synthesis of peptide thioesters and SAL esters.^[^ ^2^, ^10^, ^18^ ^]^ However, all the synthetic yields of model peptide TSAL esters through the ‘n+1’ strategy are not very ideal (10%−13%, Figures S50−S53, Supporting Information), probably due to the poor solubility of protected peptides and undesired side reactions. Then we investigated the peptide‐hydrazide‐based method on model peptides by using 2‐mercaptobenzaldehyde semicarbazone (TSAL^SCA^) as the precursor of TSAL. Surprisingly, we found that all these peptide TSAL^SCA^ esters could be facilely obtained in good yields via nitrite oxidation and thiolysis, and sequentially converted to the corresponding peptide TSAL esters by treating them with a TFA/H_2_O/pyruvic acid mixture (**Table** **1**). Moreover, the peptide TSAL esters with C‐terminal Lys/Glu residues were also successfully obtained, whereas their SAL esters could not be prepared as stable compounds.^[^ ^16^ ^]^

**Table 1:** Preparation of peptide TSAL esters.

Entry	Peptide	Yield [%]^a)^
1	LARY**A**‐	40
2	LARY**E**‐	26
3	LARY**F**‐	27
4	LARY**G**‐	31
5	LARY**H**‐	16
6	LARY**K**‐	43
7	LARY**M**‐	30
8	LEAG**R**‐	23
9	LARY**S**‐	35
10	LARY**T**‐	43
11	LEAG**Y**‐	31
12	LARY**P**‐	37
13	LARY**V**‐	21
14	GDVG**L**‐	27
15	GDVG**I**‐	26

^a)^ Yield of isolated product following reversed‐phase HPLC purification by weight over 2 steps.

In consideration of the similarities between TSAL and SAL/thiophenol, it is incredibly hard to predict whether the reaction pathways of ligations between peptide TSAL esters and Cys‐peptides are identical to that of NCL or STL. A previous work by Li et al. found that the peptide TSAL esters could undergo direct aminolysis with peptides containing different N‐terminal amino acids (including Gly, Asp, Glu, Pro, Ser, and Thr).^[^ ^19^ ^]^ However, the peptides with N‐terminal Cys or any other thiol‐derived residues had not been explored, and the C‐terminal residues were only limited to Gly and Pro. At the outset of our study, we investigated the ligation between model peptides NH_2_‐LARYK‐CO‐TSAL ester and NH_2_‐CSALF‐COOH. Based on the ultra‐performance liquid chromatography‐mass spectrometry (UPLC‐MS) analysis, it was found that a *N,S*‐benzylidene acetal intermediate was generated as the product, no matter whether pyridine/acetic acid mixture or acidic phosphate solution (pH = 3.0). The following condition screening for the ligation of model peptides NH_2_‐LARYS‐CO‐TSAL ester and NH_2_‐CSALF‐COOH indicated that the reaction could reach completion within 4 h even at a low concentration in pyridine/acetic acid (1/6, mol/mol, 2 mM) buffer (**Table** **2**). The subsequent acidolysis of *N,S*‐benzylidene acetal intermediates has also been studied at different ligation sites (Gly‐Cys, Ala‐Cys, and Phe‐Cys). Excitingly, all these *N,S*‐benzylidene acetal intermediates could be smoothly converted into natural peptidic linkages under the optimal condition [trifluoroacetic acid (TFA)/trimethylsilyl trifluoromethanesulfonate (TMSOTf)/1,2‐ethanedithiol (EDT), v/v/v = 90/5/5, 0 °C] (Table S1 and Figures S57 and S58, Supporting Information).

**Table 2:** Optimization of the ligation conditions.

Entry	X	Reaction Conditions^a)^	*t* [h]	Conversion [%]^b)^
1	**S**	Pyridine	2 (4)	19 (23)
2	**S**	Acetic acid	2 (4)	54 (79)
3	**S**	Pyridine/Acetic acid = 1/1, (mol/mol)	2 (4)	81 (88)
4	**S**	Pyridine/Acetic acid = 1/3, (mol/mol)	2 (4)	82 (83)
5	**S**	Pyridine/Acetic acid = 1/6, (mol/mol)	2 (4)	83 (> 90)
6	**S**	Pyridine/Acetic acid = 3/1, (mol/mol)	2 (4)	49 (37)
7	**S**	Pyridine/Acetic acid = 6/1, (mol/mol)	2 (4)	38 (16)
8	**S**	DMF	16	–
9	**K**	Phosphate solution containing 6 m Gn·HCl, pH 3.0	24	> 90
10	**K**	Phosphate solution containing 6 m Gn·HCl, pH 7.0	24	–

^a)^ All reactions were performed on a 2 mM scale;

^b)^ Analysis by HPLC traces of the crude reaction mixture.

### 2.2 Design and Performance with Model Peptides

With the optimized conditions in hand, we next explored the scope and limitations of this ligation method. A wide range of unprotected peptide TSAL esters bearing a variety of C‐terminal residues were prepared to react with the model cysteine peptide (NH_2_‐CSALF‐COOH). In all cases, the ligation reactions were completed within 4−5 h, affording the *N,S*‐benzylidene acetal products in high yields (49−76%) after reversed‐phase HPLC (RP‐HPLC) purification (**Table** **3**). To our surprise, unprotected C‐terminal Lys, Glu, His, Arg, Ser, and Thr were all well compatible with the ligation. The ligation rates at more sterically hindered amino acids (e.g., Pro, Val, Ile, and Thr) were also fast (within 4 h), and the isolated yields of *N,S*‐benzylidene acetal products were extremely good (49−65%). After the subsequent acidolysis of the resulting *N,S*‐benzylidene acetal intermediates under TFA/TMSOTf/EDT conditions, the product peptides with natural peptidic linkages at ligation sites were obtained in excellent yields (53−89%). Notably, most acidolysis reactions proceeded smoothly and were completed in 4 h. However, in some cases (e.g., Thr, His, and Phe), the acidolysis step requires a longer time (11−24 h), but the yields are still good (53%–78%).

**Table 3:** Scope of TSAL ester‐Cys ligation.

Entry	Peptide C‐TSAL‐ester	N‐terminal	*t* [h]	Conversion [%]^a)^	Acidolysis [h]	Yield [%]^b)^
1	LARY**A**‐	CSALF	4	> 90	4	58
2	LARY**E**‐	CSALF	4	> 90	4	47
3	LARY**F**‐	CSALF	5	> 90	11	48
4	LARY**G**‐	CSALF	4	> 90	4	48
5	LARY**H**‐	CSALF	5	> 90	24	33
6	LARY**K**‐	CSALF	4	> 90	4	59
7	LARY**M**‐	CSALF	5	> 90	4	42
8	LEAG**R**‐	CSALF	5	> 90	4	30
9	LARY**S**‐	CSALF	4	> 90	4	44
10	LARY**T**‐	CSALF	4	> 90	11	51
11	LEAG**Y**‐	CSALF	4	> 90	4	42
12	LARY**P**‐	CSALF	4	> 90	4	46
13	LARY**V**‐	CSALF	4	> 90	4	40
14	GDVG**I**‐	CSALF	4	> 90	4	35
15	AGVEG**L**‐	CSALF	4	> 90	4	38

^a)^ Analysis by HPLC traces of the crude reaction mixture;

^b)^ Yield of isolated product following reversed‐phase HPLC purification by weight over 2 steps.

To address the potential epimerization issue at the C‐terminal amino acids, two model reactions have been conducted using NH_2_‐LARYP‐CO‐TSAL ester and NH_2_‐LARYp‐CO‐TSAL ester reacting with NH_2_‐CSALF‐COOH, respectively. Through the HPLC analysis of these two epimeric products (NH_2_‐LARY**PC**SALF‐COOH and NH_2_‐LARY**pC**SALF‐COOH), distinctive retention times were observed, which means the stereochemical integrity was retained during the ligation process (Figure S54, Supporting Information). Another model test has also been made at more epimerization‐prone C‐terminal Ser, and no epimerization was observed during the ligation process (Figure S54, Supporting Information).

Penicillamine is one of the most commonly used surrogates for N‐terminal Val in NCL/CPL.^[^ ^8^, ^20^ ^]^ To evaluate the reactivity of TSAL ester‐induced ligation at sterically demanding N‐terminal residues, a model peptide containing N‐terminal penicillamine (NH_2_‐PenSALF‐COOH) was prepared and reacted with a variety of peptide TSAL esters. Under the same conditions as previously used, all these ligations could be smoothly completed within 5 h, even at C‐terminal *β*‐branched residue sites (i.e., Pro, Val, and Thr). The resulting ligation intermediates were then successfully converted to Xaa‐Pen‐linked peptides under TFA/TMSOTf/EDT conditions, affording the desired products in 24−47% isolated yields over 2 steps (**Table** **4**). As another example, we also applied this new ligation method in the synthesis of an acetylated adrenocorticotropic hormone 24 (Ac‐ACTH 24) derivative at its Pro‐Val site (Table 4‐Entry 9; Figures S105–S107, Supporting Information). Indeed, the ligation was smoothly completed within 4 h. After acidolysis of the resulting *N,S*‐benzylidene acetal intermediate, the Pro‐Pen linkage was obtained and converted to Pro‐Val linkage via subsequent desulfurization, giving the product Ac‐ACTH 24 in a 23% isolated yield over 3 steps.

**Table 4:** Scope of TSAL ester‐Penicillamine ligation.

Entry	Peptide C‐ TSAL‐ester	N‐terminal	*t* [h]	Acidolysis [h]	Yield [%]^[^ ^a^ ^]^
1	LARY**A**‐	PenSALF	5	24	45
2	LARY**K**‐	PenSALF	5	24	47
3	LARY**M**‐	PenSALF	5	24	42
4	LEAG**R**‐	PenSALF	5	24	38
5	LARY**S**‐	PenSALF	5	24	24
6	LARY**T**‐	PenSALF	5	24	45
7	LARY**P**‐	PenSALF	4	24	29
8	LARY**V**‐	PenSALF	4	24	41
9	Ac‐SYSMEHF RWGK**P**‐	PenGKKRRP VKVYP	4	24	30

^a)^ Yield of isolated product following reversed‐phase HPLC purification by weight over 2 steps.

### 2.3 Synthesis of Ubiquitin and Hyalomin‐3 by TSAL Ester‐Induced Ligation

**Figure 2:**
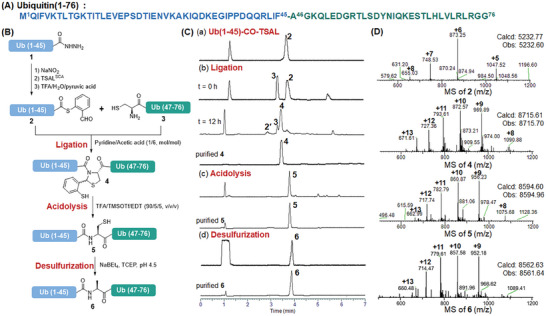
Synthesis of ubiquitin by TSAL ester‐induced ligation. A) Sequence of ubiquitin. B) Scheme for the synthesis of Ub via TSAL ester‐induced ligation. C) HPLC traces. (a‐b) Gradient: 20–90% ACN/H_2_O with 0.1% TFA over 7 min at a flow rate of 0.3 mL min^−1^. (c‐d) Gradient: 10–80% ACN/H_2_O with 0.1% TFA over 7 min at a flow rate of 0.3 mL min^−1^. D) ESI–MS characterizations of the major products. **2′**: hydrolysis of **2**.

Encouraged by the success of model peptides, we continued to test the TSAL ester‐induced ligation on the synthesis of a signaling protein named ubiquitin.^[^ ^21^ ^]^ Due to its key roles in proteasome‐dependent protein degradation,^[^ ^22^ ^]^ ubiquitin has been widely studied and synthesized via multiple strategies.^[^ ^23^ ^]^ In this work, we disconnected its sequence into two fragments, a peptide hydrazide segment Ub (1‐45)‐CO‐NHNH_2_ (**1**) and a peptide segment Ub (46‐76)‐COOH (**3**) bearing an N‐terminal Cys (**Figure** **2**A). Both peptide segments were prepared through Fmoc‐based solid phase peptide synthesis (Fmoc‐SPPS), and the peptide TSAL^SCA^ ester could be synthesized from its hydrazide precursor **1** by a one‐pot NaNO_2_ oxidation and TSAL^SCA^ treatment (Figure 2B). Subsequently, the purified Ub (1‐45)‐CO‐TSAL^SCA^ ester was subjected to TFA/H_2_O/pyruvic acid and smoothly converted to Ub (1‐45)‐CO‐TSAL ester (**2**) in 50% yield after RP‐HPLC purification. After that, the ligated reaction of peptides **2** and **3** was performed in pyridine/acetic acid (1/6, mol/mol) solution at a 2 mM concentration, to yield the *N,S*‐benzylidene acetal intermediate **4** within 12 h. The purified intermediate **4** was subsequently treated with TFA/TMSOTf/EDT to give the peptide **5** with a natural Phe‐Cys linkage at the ligation site, which was then desulfurized to convert Cys46 to Ala46. After RP‐HPLC purification, the final desired product **6** was obtained in 15% yield over 3 steps (Figure 2C,D).

**Figure 3:**
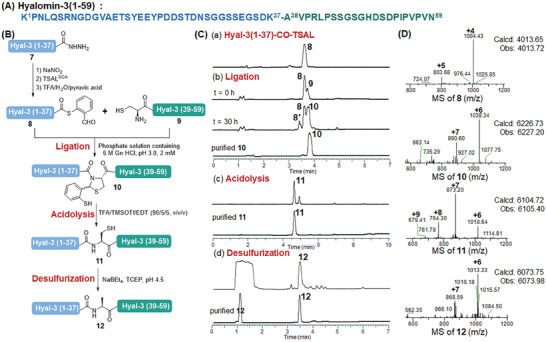
Synthesis of hyalomin‐3 by TSAL ester‐induced ligation. A) Sequence of hyalomin‐3. B) Scheme for the synthesis of Hyal‐3 via TSAL ester‐induced ligation. C) HPLC traces. (a‐b) Gradient: 10–80% ACN/H_2_O with 0.1% TFA over 7 min at a flow rate of 0.3 mL min^−1^. (c) Gradient: 10–80% ACN/H_2_O with 0.1% TFA over 10 min at a flow rate of 0.3 mL min^−1^. (d) Gradient: 10–80% ACN/H_2_O with 0.1% TFA over 7 min at a flow rate of 0.3 mL min^−1^. D) ESI–MS characterizations of the major products. **8′**: hydrolysis of **8**.

To further demonstrate theefficiency of this ligation method for the chemical synthesis of proteins, a thrombin‐inhibiting hyalomin protein, namely hyalomin‐3 (Hyal‐3),^[^ ^24^ ^]^ was selected as our next target. As shown in **Figure** **3**, the disconnection of Hyal‐3 was made at the Lys‐Ala site, while the Ala38 was replaced by cysteine residue to facilitate the TSAL ester‐induced ligation (Figure 3A). Accordingly, the C‐terminal hydrazide peptide fragment Hyal‐3 (1‐37)‐CO‐NHNH_2_ **7** and N‐terminal peptide **9** were synthesized on 2‐chlorotrityl chloride resin via Fmoc‐SPPS. After that, the peptide **7** was converted to **8** carrying a C‐terminal TSAL ester through the hydrazide‐based method as shown before, which was then ligated with fragment **9** in a 0.2 m phosphate solution containing 6 m Gn∙HCl (pH 3.0) for 30 h to get the target ligation intermediate **10** in 50% isolated yield (Figure 3B). The ligation has also been tried in pyridine/acetic acid solutions, but no expected result was obtained due to the poor solubility of fragment **8** (Figure S120, Supporting Information). Further, the intermediate **10** was subjected to acidolysis (TFA/TMSOTf/EDT, 90/5/5, v/v/v) to generate the Lys‐Cys linkage. The obtained product **11** was then treated with TCEP‐NaBEt_4_ for desulfurization, affording the final protein **12** in 53% yield over the last 2 steps after RP‐HPLC purification (Figure 3C,D).

### 2.4 Synthesis of Ubiquitin‐Fold Modifier 1 (UFM1) via a C‐to‐N Sequential TSAL Ester‐Induced Ligation Strategy

**Figure 4:**
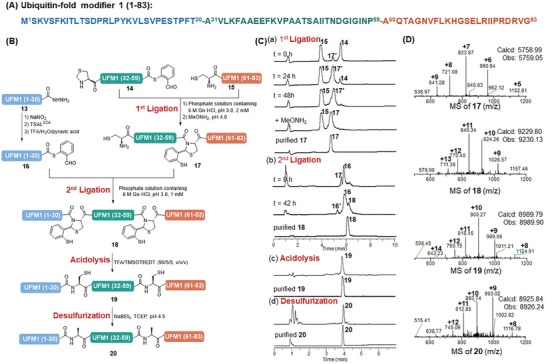
Synthesis of ubiquitin‐fold modifier 1 by TSAL ester‐induced ligation. A) Sequence of ubiquitin‐fold modifier 1. B) Scheme for the synthesis of UFM1 via a C‐to‐N Sequential TSAL ester‐induced ligation strategy. C) HPLC traces. (a) Gradient: 20–70% ACN/H_2_O with 0.1% TFA over 10 min at a flow rate of 0.3 mL min^−1^. (b) Gradient: 20–60% ACN/H_2_O with 0.1% TFA over 10 min at a flow rate of 0.3 mL min^−1^. (c) Gradient: 5–95% ACN/H_2_O with 0.1% TFA over 7 min at a flow rate of 0.3 mL min^−1^. (d) Gradient: 10–80% ACN/H_2_O with 0.1% TFA over 7 min at a flow rate of 0.3 mL min^−1^. D) ESI–MS characterizations of the major products. **16′**: hydrolysis of **16; 17′**: **17** without removal of the Thz group.

Next, we want to further investigate the extension of the TSAL ester‐induced ligation on the application of convergent strategy in the C‐to‐N direction. Firstly, we tested the C‐to‐N sequential ligation strategy on the synthesis of a protein named ubiquitin‐fold modifier 1 (UFM1), which plays important roles in multiple cellular processes as a post‐translational modifier.^[^ ^25^ ^]^ We assembled this 83‐residue‐containing protein via two TSAL ester‐induced ligations of three peptide segments at the Thr‐Ala and Pro‐Ala sites, in which both Ala31 and Ala60 were mutated to cysteine residues (**Figure** **4**A,B). All the peptide segments were synthesized by Fmoc‐SPPS, and peptide TSAL ester **14** was prepared from side‐chain protected UFM1 (31‐59)‐COOH with an N‐terminal A31Z (Z = Thz, thiazolidine) mutation, by condensation with 2‐mercaptobenzaldehyde ethylene acetal. Due to the poor solubility of **14** and **15** in the pyridine/acetic acid mixture, the first ligation was conducted in a pH 3.0 phosphate solution, giving a ligated product bearing *N,S*‐benzylidene acetal at the ligation site. Without acidolysis, the resulting *N,S*‐benzylidene acetal‐linked peptide was subsequently treated with MeONH_2_ to remove the Thz group, and purified by RP‐HPLC to afford **17** in a 49% overall yield. It is noteworthy that the *N,S*‐benzylidene acetal structure was kept intact during this progress, and no obvious byproducts were observed based on the UPLC‐MS analysis. Next, peptide **16** with a C‐terminal TSAL ester was prepared through the normal progress of nitrite oxidation, thiolysis, and activation by TFA/H_2_O/pyruvic acid. Then, the following ligation between peptide **16** and the *N,S*‐benzylidene acetal‐linked peptide **17** with an N‐terminal cysteine was conducted to obtain the product **18** in a 23% yield after RP‐HPLC purification (Figure 4B). Next, the ligated peptide **18** was subjected to acidolysis (TFA/TMSOTf/EDT) at 0 °C to generate the desired peptide **19** with Thr‐Cys and Pro‐Cys linkages at the ligation sites. Finally, after subsequent desulfurization, the full‐length UFM1 (**20**) was purified with RP‐HPLC to give an overall yield of 38% over 2 steps (Figure 4C,D).

### 2.5 Synthesis of *Mtb* CM Via Combining Chemical Ligation Approaches

**Figure 5:**
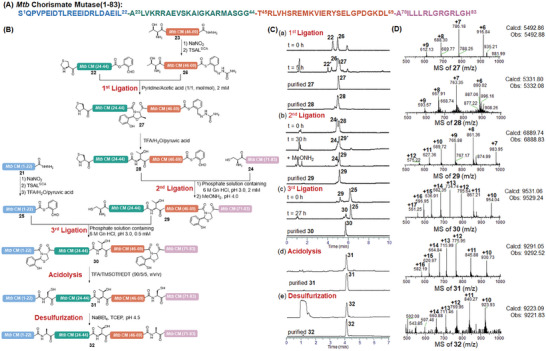
Synthesis of *Mtb* chorismate mutase by TSAL ester‐induced ligation combined with STL. A) Sequence of *Mtb* chorismate mutase. B) Scheme for the synthesis of *Mtb* CM via a combined STL and TSAL ester‐induced ligation in the N‐to‐C direction. C) HPLC traces. (a‐c) Gradient: 20–70% ACN/H_2_O with 0.1% formic acid over 10 min at a flow rate of 0.3 mL min^−1^. (d‐e) Gradient: 10–80% ACN/H_2_O with 0.1% formic acid over 7 min at a flow rate of 0.3 mL min^−1^. D) ESI–MS characterizations of the major products. **22′**: hydrolysis of **22; 29′**: **29** without removal of the Thz group.

N‐to‐C successive ligation strategy has always been considered challenging, due to the faster intramolecular reaction than intermolecular ligation.^[^ ^26^ ^]^ Having demonstrated the effectiveness of TSAL ester‐induced ligation and the C‐to‐N convergent strategy, we want to further investigate a comprehensive strategy to undergo STL and TSAL ester‐induced ligation in the N‐to‐C direction. To this end, an enzyme produced by *Mycobacterium tuberculosis* (*Mtb*), namely intracellular chorismate mutase (CM), was selected as a target. *Mtb* CM was reported to show high catalytic activity to convert chorismite to prephenate via a Claisen rearrangement.^[^ ^27^ ^]^ To maximize the protein synthesis efficiency, the full‐length *Mtb* CM was disconnected into four fragments: *Mtb* CM (1‐22)‐CO‐NHNH_2_ (**21**), side‐chain protected *Mtb* CM (23‐44)‐COOH with an N‐terminal A23Z mutation, *Mtb* CM (45‐69)‐CO‐NHNH_2_ (**23**), *Mtb* CM (70‐83)‐COOH (**24**) bearing an N‐terminal Cys (**Figure** **5**A,B).

Our initial attempt was to directly synthesize *Mtb* CM (23‐69, A23C)‐CO‐NHNH_2_ through the SPPS strategy. However, this fragment was extremely difficult to be prepared under standard Fmoc‐SPPS conditions, even with the assistance of pseudoproline dipeptides (Fmoc‐Tyr(tBu)‐Ser(*ψ^Me,Me^* Pro)‐OH, Fmoc‐Ala‐Ser(*ψ^Me,Me^* Pro)‐OH and Fmoc‐Val‐Ser(*ψ^Me,Me^* Pro)‐OH), leading to a mixture of unidentified truncated peptides. Thus, we performed its synthesis by using a Thr ligation at the Gly‐Thr site to solve the problems encountered in SPPS. Firstly, we synthesized the peptide **26** with a C‐terminal TSAL^SCA^ ester through the general procedure of oxidation‐thiolysis. The peptide SAL ester **22** was prepared through the coupling of side‐chain protected segment *Mtb* CM (23‐44)‐COOH to the salicylaldehyde dimethyl acetal, which was then ligated with peptide **26** to generate an *N,O*‐benzylidene acetal‐linked intermediate **27** in a 43% yield after RP‐HPLC purification. Then, the purified **27** was subjected to TFA/H_2_O/pyruvic acid and smoothly converted to Thz‐*Mtb* CM(24‐69)‐CO‐TSAL (**28**). The following ligation between peptides **28** and **24** proceeded smoothly in a 0.2 m phosphate solution containing 6 m Gn∙HCl (pH 3.0) and subsequently treated with MeONH_2_ (pH 4.0) to remove the Thz group. The resulting *N,S*‐benzylidene acetal‐linked peptide **29** was purified by RP‐HPLC to afford a 63% overall yield. The successful synthesis of fragment **29** has also proven the feasibility and validity of the N‐to‐C STL and TSAL ester‐induced ligation methodology (Figure 5B,C). Next, Peptide **29** with an N‐terminal Cys was ligated with peptide TSAL ester **25** which was generated from fragment **21**, affording peptide **30** in a 47% isolated yield. In this step, no STL product was observed based on the UPLC‐MS spectrum, which demonstrated that the peptide TSAL ester preferred to react with Cys under the ligation condition. The *N,S*‐benzylidene acetal intermediate **30** was then subjected to acidolysis to form peptide **31**. Finally, the mutated protein **31** was desulfurized to convert the two Leu‐Cys linkages to Leu‐Ala linkages, affording the full‐length *Mtb* CM (**32**) in a 38% yield of isolated product over the last 2 steps (Figure 5C,D).

## 3 Conclusion

In summary, we have herein developed a novel and powerful peptide ligation between peptide TSAL esters and peptides bearing N‐terminal cysteine/penicillamine that enables effective coupling reactions even at the most sterically hindered Pro‐Val site. Mechanistically, the reaction pathway seems to involve an imine formation between the N‐terminal amine group and the C‐terminal aldehyde group, followed by the attack of the thiolate to generate a thiazolidine, next affording the stable *N,S*‐benzylidene acetal intermediate through a fast S‐to‐N acyl transfer. The peptide TSAL esters can be facilely prepared through a peptide hydrazide‐based oxidation‐thiolysis‐acidolysis strategy, which makes this methodology simple and accessible. Importantly, the sufficient stability of peptide hydrazides and *N,S*‐benzylidene acetal linkages makes this ligation method compatible with other peptide ligation technologies. In addition, the peptide hydrazides can be readily obtained through SPPS or biological expression, which significantly expands the application of this methodology. Furthermore, the utilization of peptide TSAL^SCA^ ester as the precursor of peptide TSAL ester enables N‐to‐C successive STL and our new ligation method, which is another prominent advantage. The successful reaction in phosphate solution offers the possibility of ligating peptide fragments that are insoluble in pyridine/acetic acid mixtures. We believe that this TSAL ester‐cysteine/penicillamine ligation (TCPL) strategy will bring more opportunities and valuable options for the future convergent synthesis of otherwise unobtainable proteins.

## Conflict of Interest

The authors declare no conflict of interest.

## Supporting information



Supporting Information

## Data Availability

The data that support the findings of this study are available in the supplementary material of this article.
