# Supplementary material for: An Enabling Peptide Ligation Induced by Thiol‐Salicylaldehyde Ester for Chemical Protein Synthesis
Source: Adv Sci (Weinh). 2024 Oct 23;11(46):2408538. doi: 10.1002/advs.202408538 (PMC11633502; doi:10.1002/advs.202408538)
Supplement: Supplementary file 1 — Supporting Information [file ADVS-11-2408538-s001.docx]

Supporting Information

**An Enabling Peptide Ligation Induced by Thiol-salicylaldehyde Ester for Chemical Protein Synthesis**

Cuicui Li, Wenge Ma, and Kang Jin*

**Table of Contents**

1. **General remarks on materials and methods…………………………….……S3**
2. **General experimental procedures…………………………………………..…S3**
3. **Synthesis of model N-terminal Cys/penicillamine peptides………….………S7**
4. **Synthesis of model C-terminus peptide hydrazides………………………....S10**
5. **Synthesis of model C-terminus peptide TSAL^SCA^ -esters…………………...S25**
6. **Synthesis of model C-terminus peptide TSAL-esters…………………….…S40**
7. **Epimerization study of Cys ligation……………………………………….…S61**
8. **Ligation reaction condition screening for TCPL……………………………S63**
9. **Acidolysis condition screening for TCPL……………………………………S65**
10. **TSAL ester-Cys ligation of model peptides…………………………………..S68**
11. **TSAL ester-Penicillamine ligation of model peptides……………………...S103**
12. **Synthesis of Ubiquitin (1-76)……………………………………………...…S124**
13. **Synthesis of Hyalomin-3 (1-59)……………………………………………...S134**
14. **Synthesis of Ubiquitin-fold modifier 1 (1-83)……………………………....S143**
15. **Synthesis of *Mtb* Chorismate Mutase (1-83)………………………………..S155**
16. **Synthesis of thiol-salicylaldehyde semicarbazone………………………….S172**
17. **References…………………………………………………………………….S175**
18. **General remarks on materials and methods**

All solvents in reagent grade (FuYu Chem) or HPLC grade (Oceanpak) were used without purification. Commercially available materials were obtained from Bidepharm, Energy Chemicals, Adamas, or Sigma-Aldrich, Macklin, MREDA, J&K Scientific, Sinopharm, Aladdin, or Acmec. All commercially available amino acids, 1-hydroxybenzotriazole (HOBt), 2-(7-Azabenzotriazol-1-yl)-N,N,N',N'-tetramethyluronium hexafluorophosphate (HATU) and 2-Chorotrityl Chloridie resin were purchased from GL Biochem. The deionized water used in the experiments was Wahaha pure water (Wahaha Food and Beverage Ltd.). Analytical HPLC (Agilent 1260) was performed on a COSMOSIL C18 column (5 μm, 10 × 250 mm) or Agilent C18 column (2.7 μm, 2.1 × 100 mm) running at a flow rate of 2 mL/min or 0.3 mL/min with UV detection at 215 and 254 nm. Preparative HPLC was performed on a Waters system equipped with a UV/Visible detector (Waters 2489) using a Daisogel C18 Prep column (10 μm, 30 × 250 mm) running at a flow rate of 15 mL/min with UV detection at 215 and 254 nm. LC-MS was performed on a Thermo Scientific UltiMate 3000 UHPLC system equipped with LCQ Fleet Ion Trap Mass Spectrometer (Thermofisher Scientific, Bremen, Germany) on a Thermo Scientific C18 column (2.7 μm, 2.1 × 100 mm) running at a flow rate of 0.3 mL/min with UV detection at 215 nm and 254 nm. Mobile phases of HPLC used are as followed: Solvent A: 0.1 % TFA (v/v) in water; Solvent B: 0.1 % TFA (v/v) in MeCN. ^1^H and ^13^C NMR spectra were recorded on Bruker AMX-400 MHz spectrometers or Bruker Avance DRX 600 FT-NMR spectrometer. ^1^H NMR and ^13^C NMR chemical shifts were determined relative to internal standard TMS at δ 0.0 ppm. Chemical shifts (δ) are expressed in parts per million (ppm), and coupling constants (J) are in Hertz (Hz). The following abbreviations were used to indicate the corresponding multiplicities: s = singlet, d = doublet, m = multiplet, br = broad.

**2. General experimental procedures**

2.1 Fmoc-based Solid-phase Peptide Synthesis (SPPS)

The solid phase peptide synthesis was performed on 2-Cl-(Trt)-Cl resin (loading: ~0.5 mmol/g) manually. 2-Cl-(Trt)-Cl resin was initially swelled with DCM/DMF (1/1) about 30 min and then washed it with DCM (5 mL × 3), DMF (5 mL × 3), DCM (5 mL × 3). For the first amino acid, a solution of Fmoc-Xaa-COOH (4.0 equiv. relative to resin loading capacity) and DIEA (8.0 equiv. relative to resin capacity) in DCM was added and the resin was shaken at room temperature for 2 h. Then the resin was washed with DMF (5 mL × 3), DCM (5 mL × 3), DMF (5 mL × 3), and subsequently capped with a solution of DCM/MeOH/DIEA (17:2:1, v/v/v, 5 mL) for 1 h. The resin was washed, and then fmoc deprotections were performed with 20% piperidine in DMF for 20 min. Finally, it was submitted to iterative peptide assembly (Fmoc-SPPS) to give the corresponding peptide product. The following Fmoc amino acids with side-chain protecting groups were employed: Fmoc-Ala-OH, Fmoc-Cys(Trt)-OH, Fmoc-Asp(OtBu)-OH, Fmoc-Glu(OtBu)-OH, Fmoc-Phe-OH, Fmoc-Gly-OH, Fmoc-(DMB)Gly-OH, Fmoc-His(Trt)-OH, Fmoc-Ile-OH, Fmoc-Lys(Boc)-OH, Fmoc-Leu-OH, Fmoc-Met-OH, Fmoc-Asn(Trt)-OH, Fmoc-Pro-OH, Fmoc-D-Pro-OH, Fmoc-Gln(Trt)- OH, Fmoc-Arg(Pbf)-OH, Fmoc-Ser(tBu)-OH, Fmoc-Thr(tBu)-OH, Fmoc-Val-OH, Fmoc-Trp(Boc)-OH, Fmoc-Tyr(tBu)-OH, Boc-Pen(Trt)-OH, and Boc-Thz-OH. For the coupling step, a solution of Fmoc protected amino acid or Boc protected amino acid (4.0 equiv.), HATU (4.0 equiv.) and DIEA (8 equiv.) in DMF was gently agitated with the resin at room temperature for 1h. A double coupling strategy is needed for Fmoc-His(Trt)-OH, Fmoc-Cys(Trt)-OH, Fmoc-Asn(Trt)-OH, Fmoc-Ile-OH, Fmoc-Thr(tBu)-OH, Fmoc-Gln(Trt)-OH, Fmoc-Arg(Pbf)-OH, Fmoc-Val-OH and the amino acids to be coupled right after a Pro residue. The obtained resin bound peptide was treated with TFA cocktail (TFA/H_2_O/phenol/thioanisole/EDT = 82.5%/5%/5%/5%/2.5%) for 2-3 h. Remove most of the TFA by blowing nitrogen over the mixture. The crude peptides were acquired by precipitation with cold ether and centrifugation. After decanting diethyl ether, the remaining solid was ready for HPLC purification.

2.2 Preparation of Fmoc hydrazine 2-chlorotrityl chloride resin

The hydrazide resin was prepared by hydrazination of a 2-chlorotrityl-chloride resin as previously described^1^. 2-Cl-(Trt)-Cl resin was swelled in DCM at 0°C for 15 min. Fmoc-NHNH_2_ (4 equiv.) and DIEA (10 equiv.) in DMF were added into the resin at 0°C. The reaction mixture was gradually warmed to ambient temperature and stirred overnight. After completion, methanol was added, and the solution was stirred for 1h. Finally, the resin was filtered and washed with DMF, methanol, DCM, and diethyl ether. The resulting dried resin was stored at 4°C and ready for iterative peptide assembly (Fmoc-SPPS).

2.3 Cleavage fully protected peptide from 2-chloro-trityl chloride resin

After the standard Fmoc-SPPS, the on-resin fully protected peptide was washed with DCM for 3 times and then treated with mild acidic cleavage cocktail of DCM/AcOH/trifluoroethanol (8/1/1, v/v/v), 3 times for 1 h each. Following filtration, the resulting cleavage solutions bearing the desired peptidyl acid were combined and co-evaporated with hexane to give crude protected peptide.

2.4 General procedure for synthesis of model C-terminus peptide TSAL^SCA^ -esters

The peptide hydrazides were dissolved in 0.2 M Phosphate solution containing 6 M Gn·HCl (pH 3.0) and precooled at –15°C for 10 min. Then 10 equiv. of NaNO_2_ (prepared as 0.5 M solution in deionized water) was added and reacted at –15°C for 15 min. Subsequently, 20 equiv. of TSAL^SCA^ (dissolved in DMSO and then prepared as 0.2 M solution in 0.2 M Phosphate solution containing 6 M Gn·HCl (pH 7.0)) was added. The reaction was stirred for 1-2 h at room temperature. Afterwards, the reaction mixture was diluted with water for HPLC analysis and purification, and the isolated yield of desired products was calculated by weight.

2.5 General procedures for synthesis of model C-terminus peptide TSAL-esters

2.5.1 Pyruvic acid treatment of peptide TSAL^SCA^-esters for C-terminus peptide TSAL-esters

TFA/H_2_O (95/5, v/v) and pyruvic acid (100 equiv.) were added to the peptide TSAL^SCA^ -esters (5 mM) for 3 h. Remove most of the TFA by blowing nitrogen over the mixture. The crude products were obtained by precipitation with cold ether and centrifugation. Preparative HPLC purification followed by lyophilization afforded peptide TSAL-esters as white powder.

2.5.2 Direct coupling for C-terminus Gly and Pro peptide TSAL esters

Gly and Pro peptide TSAL esters can also be obtained according to previously described method^3^. The fully protected peptidyl acid (1.0 equiv.) was dissolved in DCM or DMF (10 mM), then benzotriazol-1-yl-oxytripyrrolidinophosphonium hexafluorophosphate (PyBOP) (3.0 equiv.), N,N-Diisopropylethylamine (DIEA) (6.0 equiv.) and 2-mercaptobenzaldehyde ethylene acetal (30 equiv.) were then added. The reaction mixture was stirred at room temperature for overnignt. After that, the solvent was removed under reduced pressure and the resulting residue was treated with TFA/H_2_O/TIPS (95/2.5/2.5, v/v/v). After global deprotection for 2 h, TFA was blown off and the oily residue was precipitated with ether and centrifuged. The resulting solid was purified by preparative HPLC and then lyophilization to give the peptide TSAL esters as white solid.

2.5.3 “N+1” strategy for C-terminus Ala, Val, and Leu peptide TSAL esters

The fully protected peptidyl acid (1.0 equiv.) was dissolved in CHCl_3_/trifluoroethanol (10 mM, 3/1, v/v), and then the corresponding amino L-Amino thio-salicylaldehyde semicarbazone ester hydrochloride (HCl·H_2_N-Xaa-CO-TSAL^SCA^) (6.0 equiv.) and Hydroxy-3,4-dihydro-4-oxo-1,2,3-benzotriazine (HOOBt) (3.0 equiv.) were then added. Finally, N-(3-dimethylaminopropyl)-N’-ethylcarbodiimide (EDC) (3.0 equiv.) was added. The reaction mixture was stirred for overnight to form the crude protected C-terminal peptide TSAL^SCA^ ester. After that, the solvent was removed under reduced pressure and the resulting residue was treated with TFA/H_2_O (95:5, v/v) containing pyruvic acid (100 equiv.) for 3 h. After that, TFA was blown off and the oily residue was triturated with diethyl ether and centrifuged. The precipitate was pelleted and the ether was subsequently decanted. The resulting solid was purified by HPLC and then lyophilization to give the peptide SAL esters as white solid.

2.6 General procedure for TSAL ester-Cys/ Penicillamine ligation

Peptide TSAL ester (1.0 equiv.) and N-terminal Cys/ Penicillamine peptide (1.1-1.5 equiv.) were incubated in pyridine/acetic acid (1/1 or 1/6, mol/mol) at a concentration of 2-5 mM at room temperature. After the completion of the reaction, which was monitored by LC-MS, the solvent was blown off under a stream of nitrogen gas. The residue was precipitated with diethyl ether and centrifuged. The precipitate was pelleted and the ether was subsequently decanted. The resulting solid was purified by HPLC and then lyophilization to give the peptide as white solid.

For peptide TSAL ester (1.0 equiv.) and N-terminal Cys/ Penicillamine peptide (1.1-1.5 equiv.) insoluble in pyridine/acetic acid were incubated in 0.2 M Phosphate solution containing 6 M Gn·HCl (pH 3.0) at a concentration of 2 mM at 25°C or 37°C. After the completion of the reaction, the solvent was diluted with water and purified by HPLC and then lyophilization to give the peptide as white solid.

2.7 General procedure for Acidolysis

The peptide was treated with acidolysis cocktail (TFA/EDT/TMSOTf (90/5/5, v/v/v)) at a concentration of 2 mM at 0°C for 4-24 h. After that, TFA was blown off and the residue was triturated with diethyl ether and centrifuged. The precipitate is pelleted and the ether is subsequently decanted. The resulting solid was purified by HPLC and then lyophilization to give the peptide as white solid.

2.8 General procedure for Desulfurization

Desulfurization reaction was carried out according to NaBEt_4_-based desulfurization method^2^. The protein was added to the pH 4.5 buffer containing 0.5 M citrate, 6 M Gn.HCl and 0.1 M TCEP at 1.0~10.0 mg/ml final protein concentration in a centrifuge tube for few minutes. Then in another separate tube, NaBEt_4_ was dissolved in H_2_O at a concentration of around 0.5-1.0 g/mL. As follows, the freshly prepared NaBEt_4_ solution was added to the substrate solution at the final concentration of 0.1 M. Subsequently, the cap of centrifuge tube was enclosed and the tube was shaken to mix the solution well for seconds. The desulfurization was triggered immediately with the generation of bubble after mixing. After the bubble was faded in 30s to few minutes, the reaction solution was taken for UPLC analysis to confirm the complete desulfurization. The remaining mixture was further diluted by H_2_O and then subjected to HPLC purification.

**3. Synthesis of model N-terminal Cys/penicillamine peptides**

3.1 H-CSALF-OH

H-CSALF-OH was assembled according to general Fmoc-SPPS procedure (0.2 mmol). The crude peptide was purified by preparative HPLC (10-40% ACN/H_2_O over 30 min) and lyophilized to afford the H-CSALF-OH (73.2 mg, 68% yield) as a white powder.


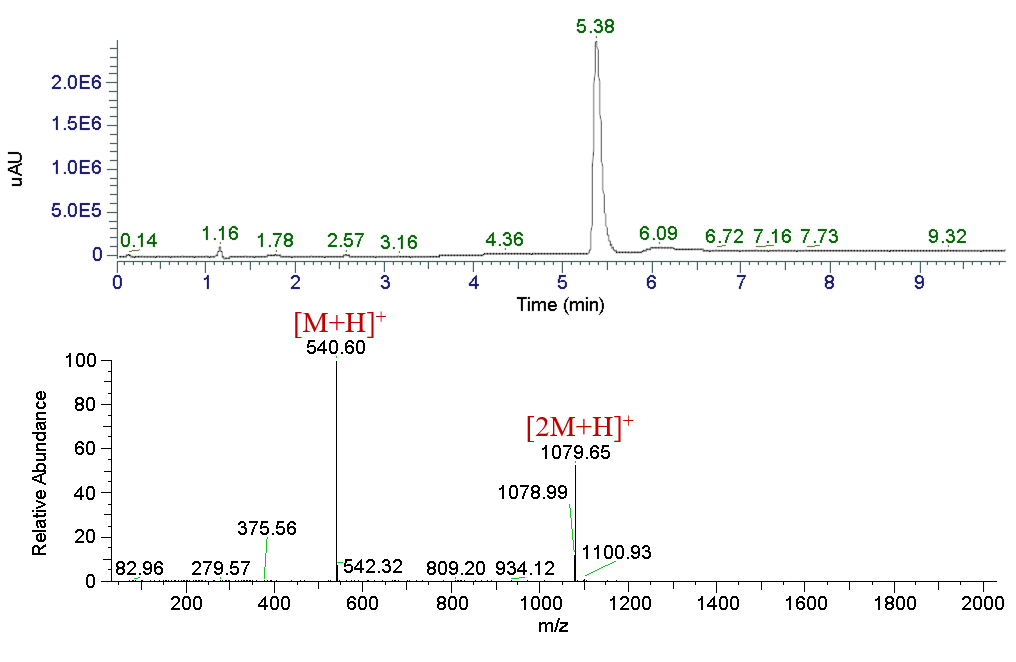


**Figure S1.** UV trace and corresponding MS from LC-MS analysis of purified H-CLAFS-OH. Gradient: 5-95% ACN/H_2_O with 0.1% TFA over 10 min at a flow rate of 0.3 mL/min. ESI-MS calcd.for C_24_H_37_N_5_O_7_S [M+H]^+^m/z = 540.24, found 540.60; [2M+H]^+^ m/z = 1079.48, found 1079.65.

3.2 H-PenSALF-OH

H-PenSALF-OH was assembled according to general Fmoc-SPPS procedure (0.1 mmol). The crude peptide was purified by preparative HPLC (10-40% ACN/H_2_O over 30 min) and lyophilized to afford the H-PenSALF-OH (35.1 mg, 62% yield) as a white powder.


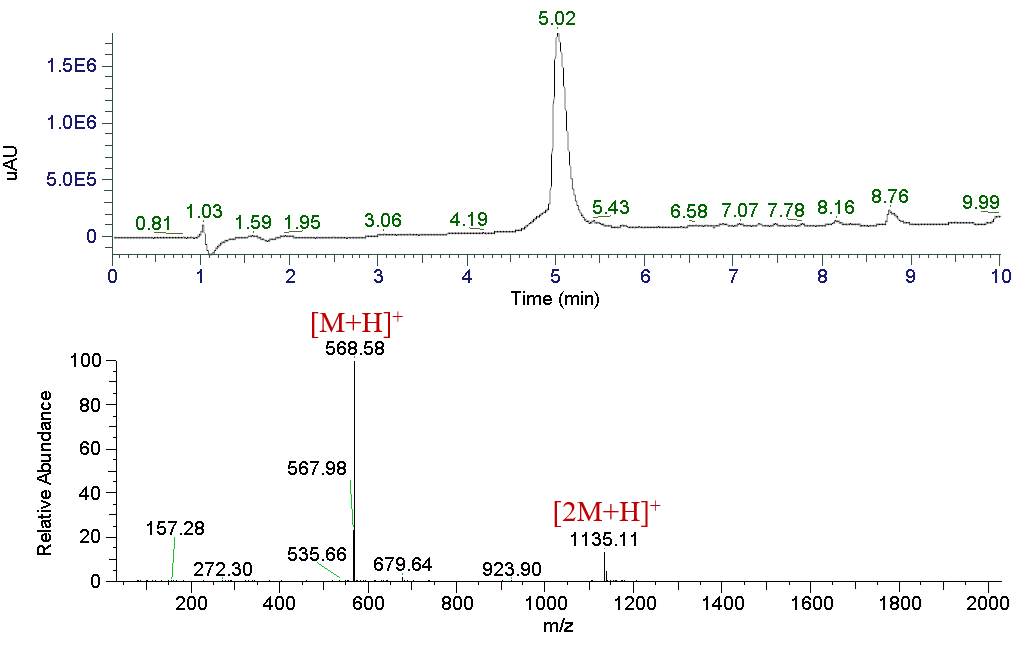


**Figure S2.** UV trace and corresponding MS from LC-MS analysis of purified H-PenLAFS-OH.

Gradient: 10-80% ACN/H_2_O with 0.1% TFA over 10 min at a flow rate of 0.3 mL/min. ESI-MS calcd.for C_26_H_41_N_5_O_7_S [M+H]^+^ m/z = 568.27, found 568.58; [2M+H]^+^ m/z = 1135.54, found 1135.11.

3.3 H-PenGKKRRPVKVYP-OH

H-PenGKKRRPVKVYP-OH was assembled according to general Fmoc-SPPS procedure (0.1 mmol). The crude peptide was purified by preparative HPLC (10-60% ACN/H_2_O over 50 min) and lyophilized to afford the H-PenGKKRRPVKVYP-OH (55.4 mg, 38% yield) as a white powder.


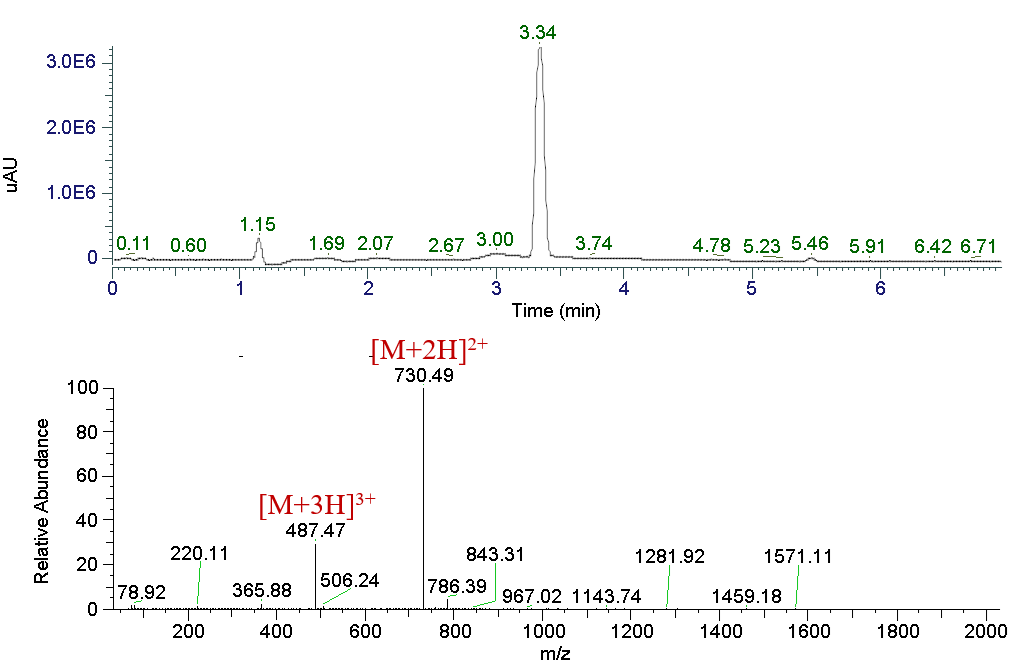


**Figure S3.** UV trace and corresponding MS from LC-MS analysis of purified H-PenGKKRRPVKVYP-OH. Gradient: 5-95% ACN/H_2_O with 0.1% TFA over 7 min at a flow rate of 0.3 mL/min. ESI-MS calcd.for C_66_H_115_N_21_O_14_S [M+2H]^2+^ m/z = 729.94, found 730.49; [M+3H]^3+^ m/z = 486.96, found 487.47.

**4. Synthesis of model C-terminus peptide hydrazides**

4.1 H-LARYA-NHNH_2_

H-LARYA-NHNH_2_ was assembled according to general Fmoc-SPPS procedure (0.2 mmol). The crude peptide was purified by preparative HPLC (10-60% ACN/H_2_O over 50 min) and lyophilized to afford the H-LARYA-NHNH_2_ (80.0 mg, 66% yield) as a white powder.


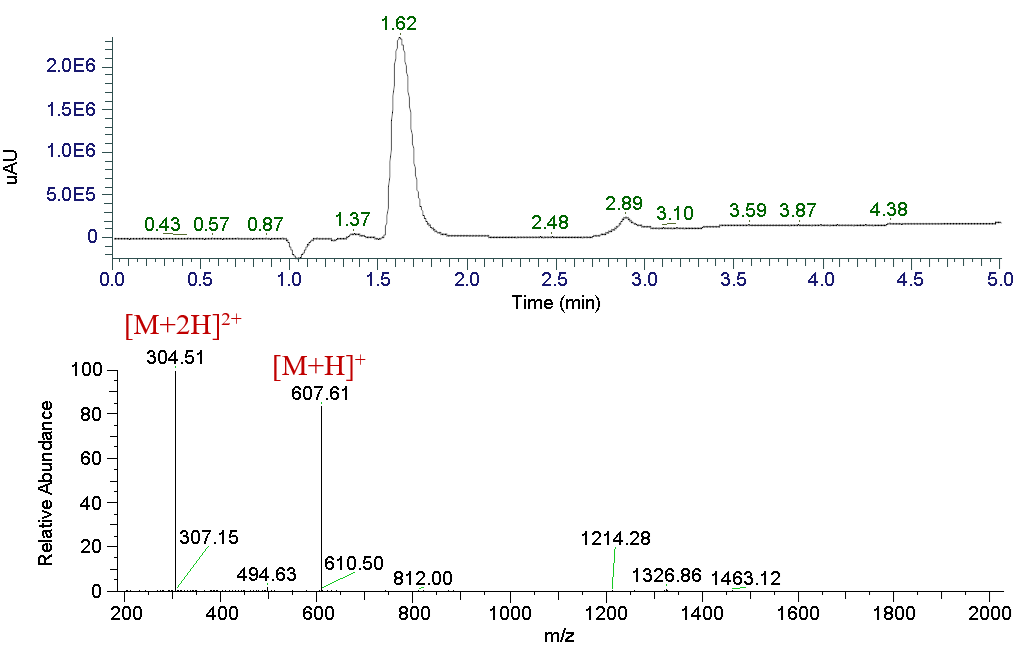


**Figure S4.** UV trace and corresponding MS from LC-MS analysis of purified H-LARYA-NHNH_2_. Gradient: 5-95% ACN/H_2_O with 0.1% TFA over 5 min at a flow rate of 0.3 mL/min. ESI-MS calcd.for C_27_H_46_N_10_O_6_ [M+H]^+^ m/z = 607.36, found 607.61; [M+2H]^2+^ m/z = 304.18, found 304.51.

4.2 H-LARYE-NHNH_2_

H-LARYE-NHNH_2_ was assembled according to general Fmoc-SPPS procedure (0.2 mmol). The crude peptide was purified by preparative HPLC (10-60% ACN/H_2_O over 50 min) and lyophilized to afford the H-LARYE-NHNH_2_ (73.1 mg, 55% yield) as a white powder.


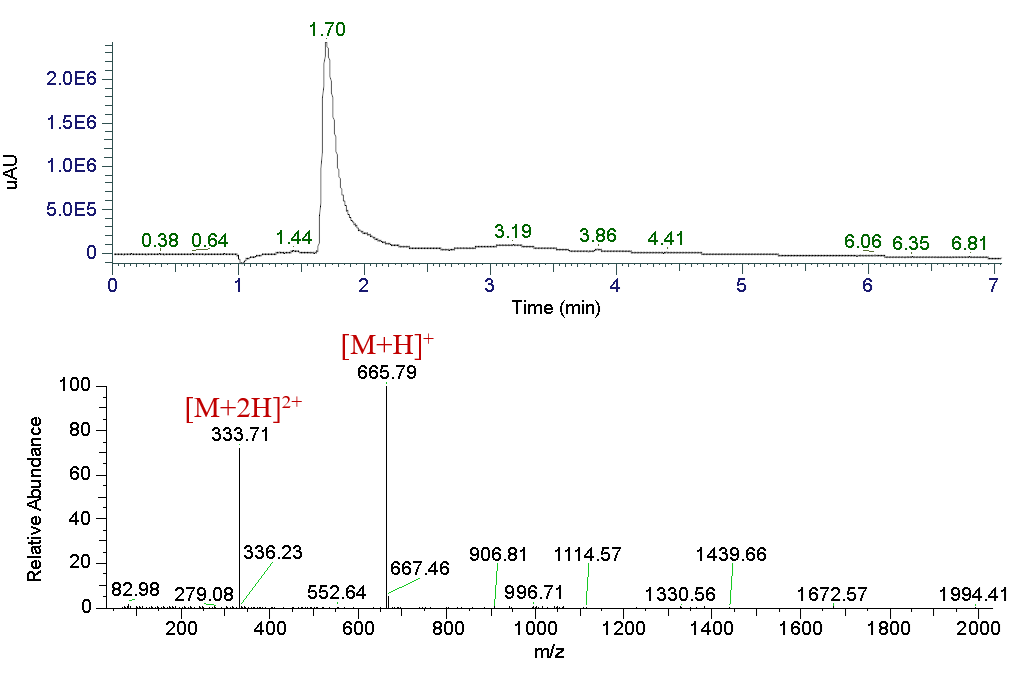


**Figure S5.** UV trace and corresponding MS from LC-MS analysis of purified H-LARYE-NHNH_2_. Gradient: 5-40% ACN/H_2_O with 0.1% TFA over 7 min at a flow rate of 0.3 mL/min. ESI-MS calcd.for C_29_H_48_N_10_O_8_ [M+H]^+^ m/z = 665.37, found 665.79; [M+2H]^2+^ m/z = 333.19, found 333.71.

4.3 H-LARYF-NHNH_2_

H-LARYF-NHNH_2_ was assembled according to general Fmoc-SPPS procedure (0.2 mmol). The crude peptide was purified by preparative HPLC (10-60% ACN/H_2_O over 50 min) and lyophilized to afford the H-LARYF-NHNH_2_ (93.7 mg, 68% yield) as a white powder.


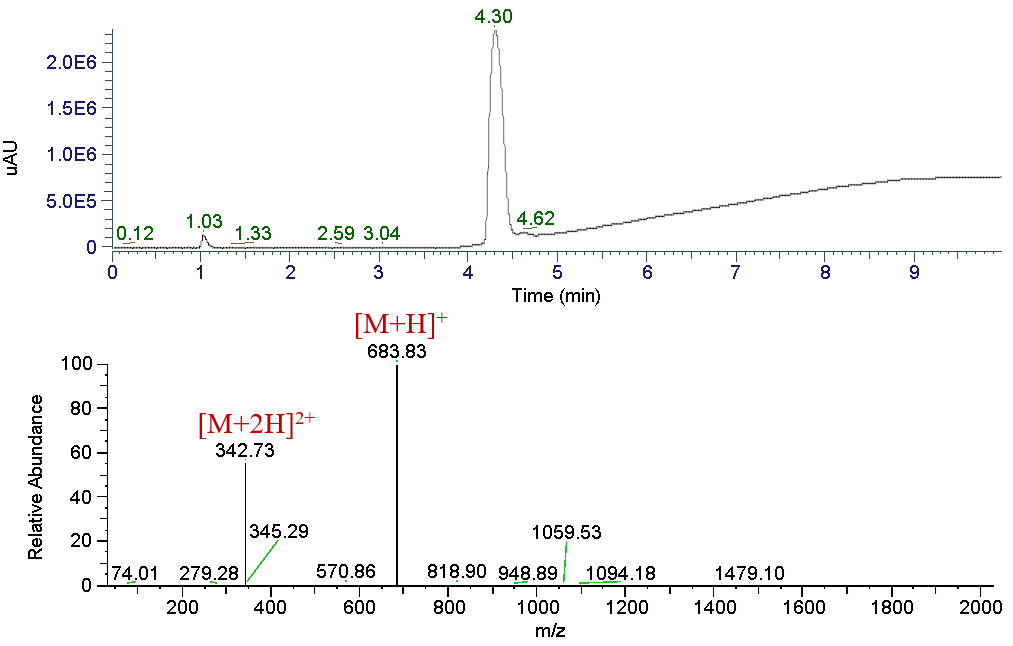


**Figure S6.** UV trace and corresponding MS from LC-MS analysis of purified H-LARYF-NHNH_2_. Gradient: 5-95% ACN/H_2_O with 0.1% TFA over 10 min at a flow rate of 0.3 mL/min. ESI-MS calcd.for C_33_H_50_N_10_O_6_ [M+H]^+^ m/z = 683.39, found 683.83; [M+2H]^2+^ m/z = 342.20, found 342.73.

4.4 H-LARYG-NHNH_2_

H-LARYG-NHNH_2_ was assembled according to general Fmoc-SPPS procedure (0.2 mmol). The crude peptide was purified by preparative HPLC (10-60% ACN/H_2_O over 50 min) and lyophilized to afford the H-LARYG-NHNH_2_ (71.2 mg, 60% yield) as a white powder.


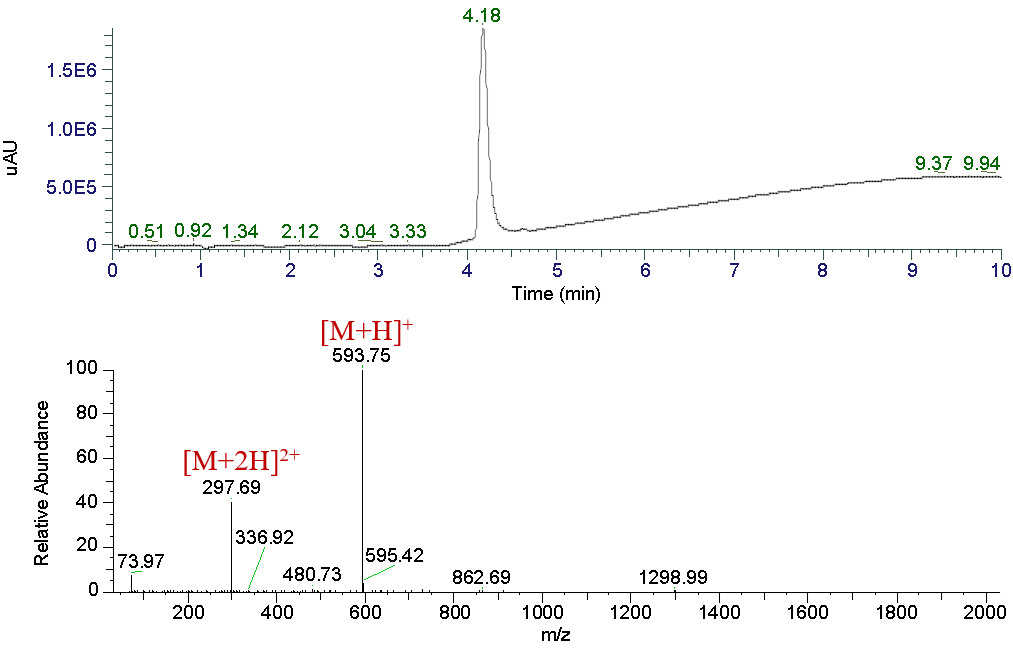


**Figure S7.** UV trace and corresponding MS from LC-MS analysis of purified H-LARYG-NHNH_2_. Gradient: 5-95% ACN/H_2_O with 0.1% TFA over 10 min at a flow rate of 0.3 mL/min. ESI-MS calcd.for C_26_H_44_N_10_O_6_ [M+H]^+^ m/z = 593.34, found 593.75; [M+2H]^2+^ m/z = 297.17, found 297.69.

4.5 H-LARYH-NHNH_2_

H-LARYH-NHNH_2_ was assembled according to general Fmoc-SPPS procedure (0.2 mmol). The crude peptide was purified by preparative HPLC (5-40% ACN/H_2_O over 50 min) and lyophilized to afford the H-LARYH-NHNH_2_ (63.0 mg, 47% yield) as a white powder.


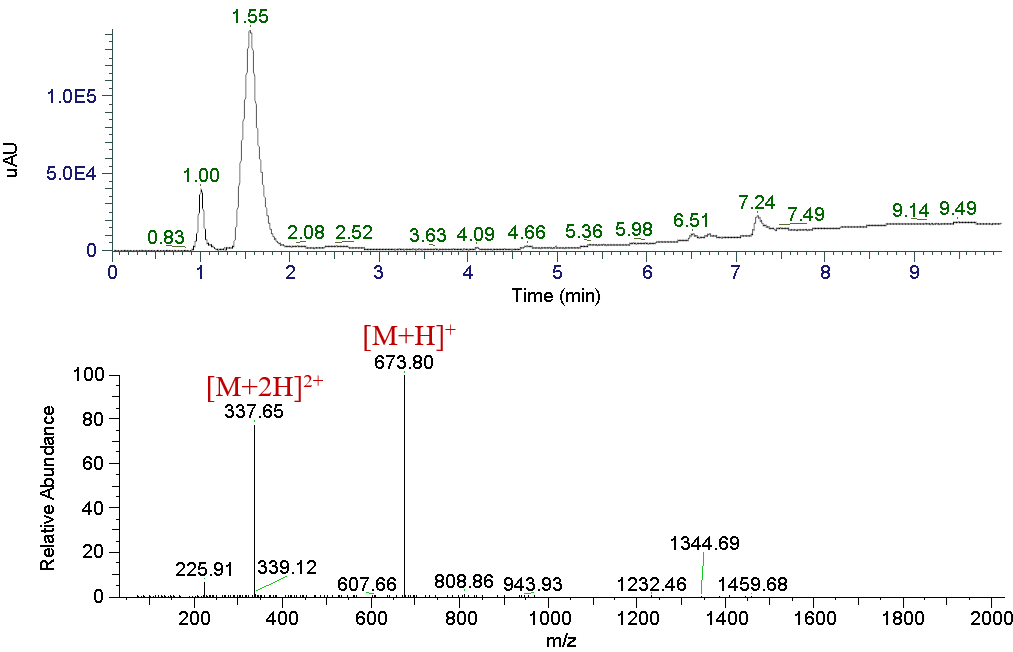


**Figure S8.** UV trace and corresponding MS from LC-MS analysis of purified H-LARYH-NHNH_2_. Gradient: 5-95% ACN/H_2_O with 0.1% TFA over 10 min at a flow rate of 0.3 mL/min. ESI-MS calcd.for C_30_H_48_N_12_O_6_ [M+H]^+^ m/z = 673.38, found 673.80; [M+2H]^2+^ m/z = 337.19, found 337.65.

4.6 H-LARYK-NHNH_2_

H-LARYK-NHNH_2_ was assembled according to general Fmoc-SPPS procedure (0.2 mmol). The crude peptide was purified by preparative HPLC (5-40% ACN/H_2_O over 30 min) and lyophilized to afford the H-LARYK-NHNH_2_ (69.3 mg,52% yield) as a white powder.


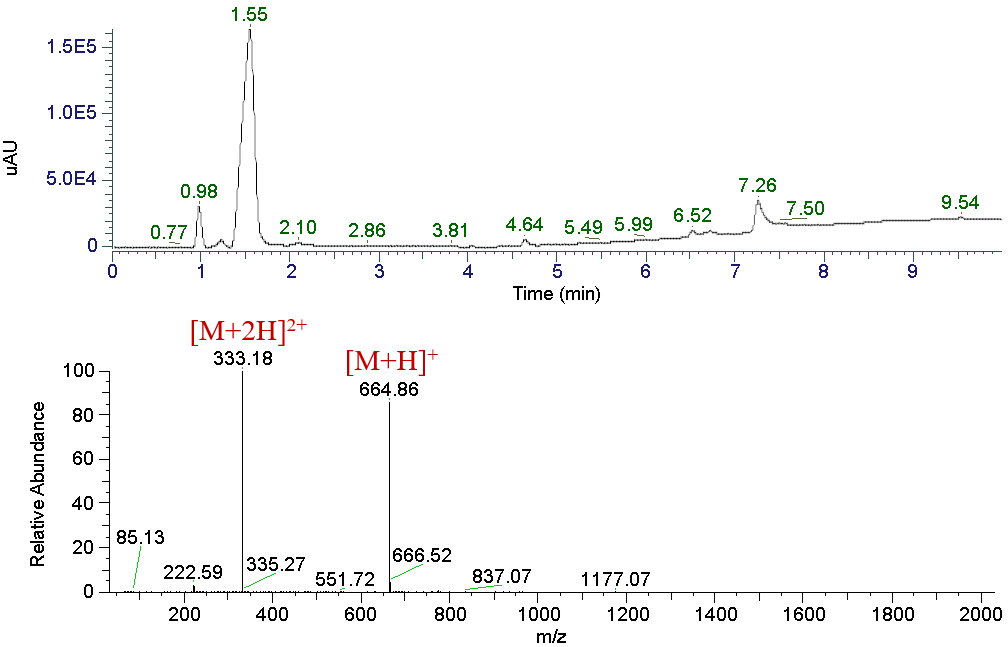


**Figure S9.** UV trace and corresponding MS from LC-MS analysis of purified H-LARYK-NHNH_2_. Gradient: 5-95% ACN/H_2_O with 0.1% TFA over 10 min at a flow rate of 0.3 mL/min. ESI-MS calcd.for C_30_H_53_N_11_O_6_ [M+H]^+^ m/z = 664.42, found 664.86; [M+2H]^2+^ m/z = 332.71, found 333.18.

4.7 H-LARYM-NHNH_2_

H-LARYM-NHNH_2_ was assembled according to general Fmoc-SPPS procedure (0.2 mmol). The crude peptide was purified by preparative HPLC (10-60% ACN/H_2_O over 50 min) and lyophilized to afford the H-LARYM-NHNH_2_ (59.9 mg, 44% yield) as a white powder.


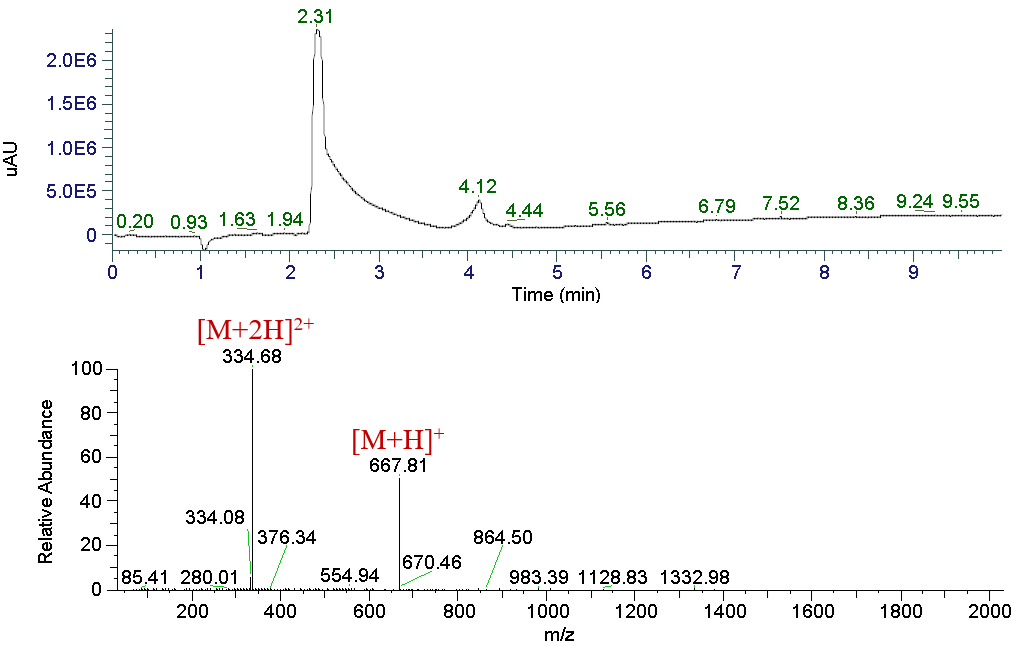


**Figure S10.** UV trace and corresponding MS from LC-MS analysis of purified H-LARYM-NHNH_2_. Gradient: 5-95% ACN/H_2_O with 0.1% TFA over 10 min at a flow rate of 0.3 mL/min. ESI-MS calcd.for C_29_H_50_N_10_O_6_S [M+H]^+^ m/z = 667.36, found 667.81; [M+2H]^2+^ m/z = 334.18, found 334.68.

4.8 H-LARYS-NHNH_2_

H-LARYS-NHNH_2_ was assembled according to general Fmoc-SPPS procedure (0.2 mmol). The crude peptide was purified by preparative HPLC (10-60% ACN/H_2_O over 50 min) and lyophilized to afford the H-LARYS-NHNH_2_ (61.5 mg, 49% yield) as a white powder.


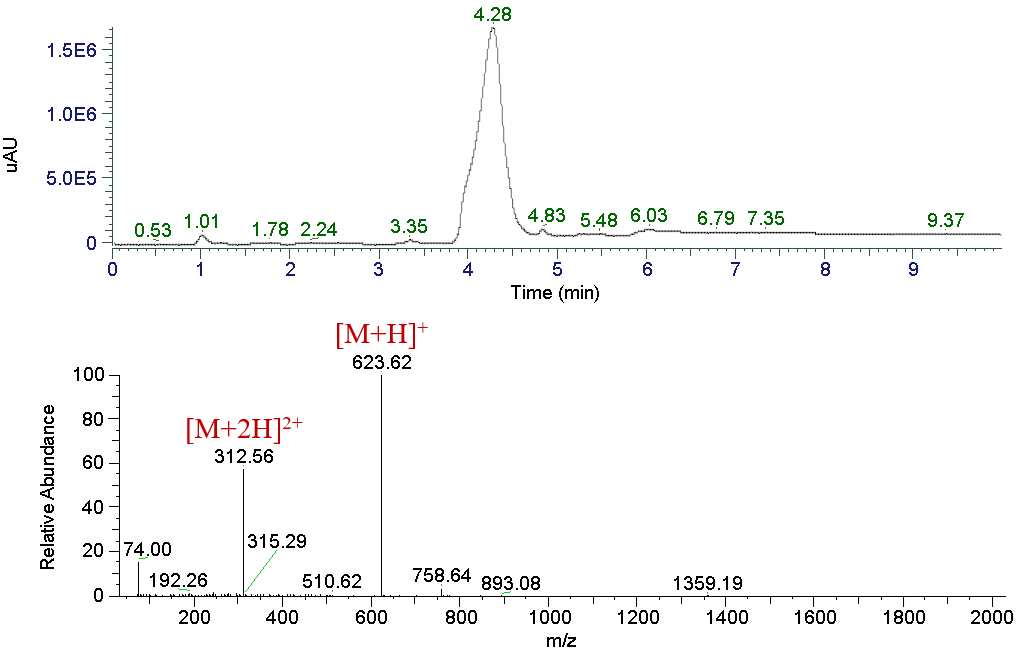


**Figure S11.** UV trace and corresponding MS from LC-MS analysis of purified H-LARYS-NHNH_2_. Gradient: 5-95% ACN/H_2_O with 0.1% TFA over 10 min at a flow rate of 0.3 mL/min. ESI-MS calcd.for C_27_H_46_N_10_O_7_ [M+H]^+^ m/z = 623.36, found 623.62; [M+2H]^2+^ m/z = 312.18, found 312.56.

4.9 H-LARYT-NHNH_2_

H-LARYT-NHNH_2_ was assembled according to general Fmoc-SPPS procedure (0.2 mmol). The crude peptide was purified by preparative HPLC (10-60% ACN/H_2_O over 50 min) and lyophilized to afford the H-LARYT-NHNH_2_ (69.0 mg, 54% yield) as a white powder.


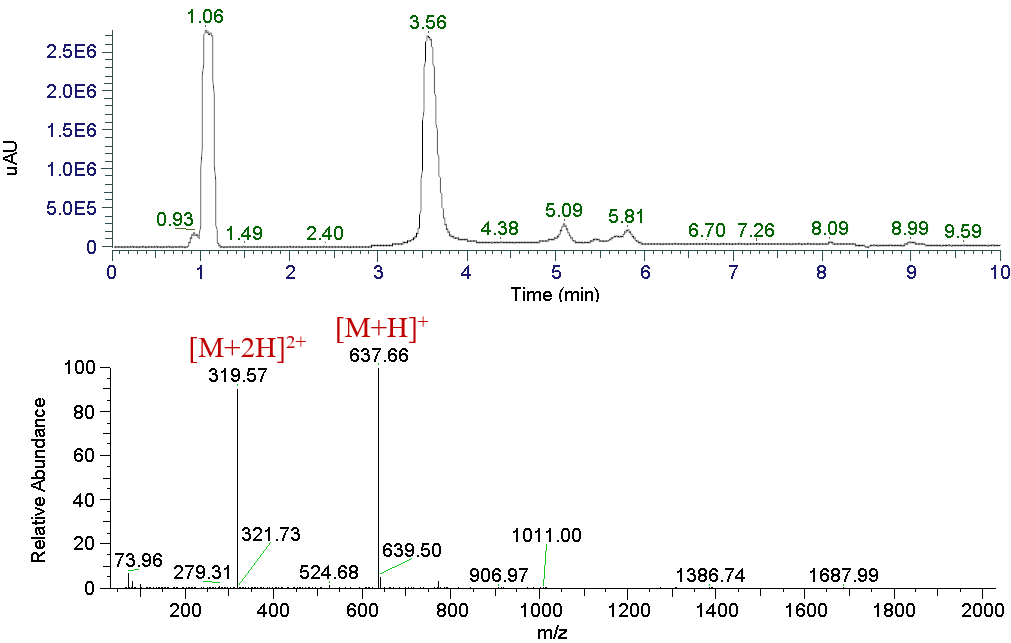


**Figure S12.** UV trace and corresponding MS from LC-MS analysis of purified H-LARYT-NHNH_2_. Gradient: 5-95% ACN/H_2_O with 0.1% TFA over 10 min at a flow rate of 0.3 mL/min. ESI-MS calcd.for C_28_H_48_N_10_O_7_ [M+H]^+^ m/z = 637.37, found 637.66; [M+2H]^2+^ m/z = 319.19, found 319.57.

4.10 H-LARYP-NHNH_2_

H-LARYP-NHNH_2_ was assembled according to general Fmoc-SPPS procedure (0.1 mmol). The crude peptide was purified by preparative HPLC (10-60% ACN/H_2_O over 50 min) and lyophilized to afford the H-LARYP-NHNH_2_ (39.6 mg, 62% yield) as a white powder.


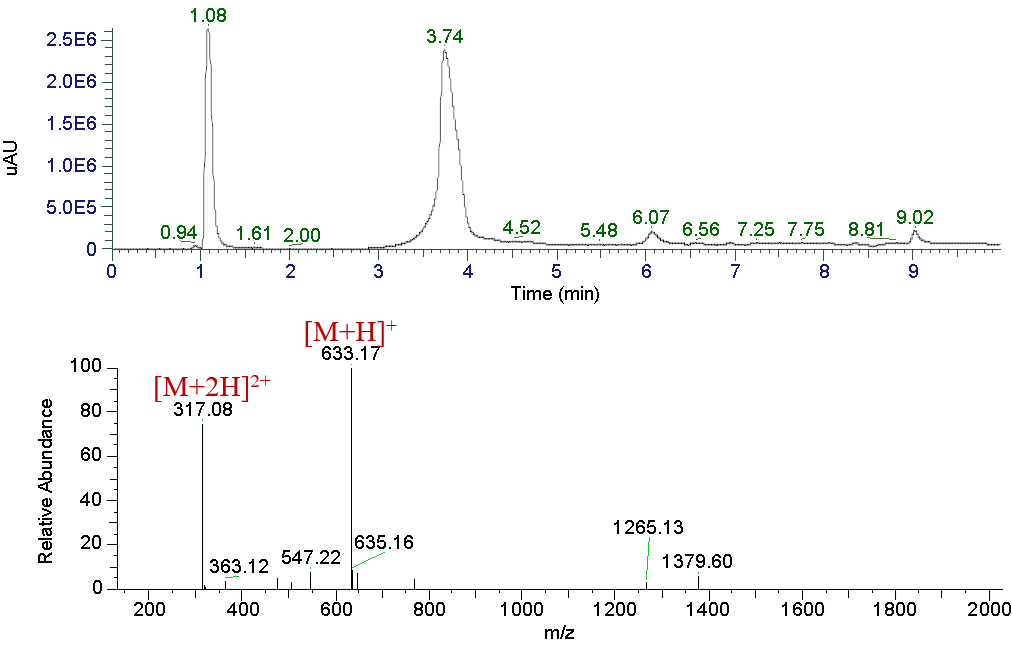


**Figure S13.** UV trace and corresponding MS from LC-MS analysis of purified H-LARYP-NHNH_2_. Gradient: 5-60% ACN/H_2_O with 0.1% TFA over 10 min at a flow rate of 0.3 mL/min. ESI-MS calcd.for C_29_H_48_N_10_O_6_ [M+H]^+^ m/z = 633.38, found 633.17; [M+2H]^2+^ m/z = 317.19, found 317.08.

4.11 H-LARYV-NHNH_2_

H-LARYV-NHNH_2_ was assembled according to general Fmoc-SPPS procedure (0.1 mmol). The crude peptide was purified by preparative HPLC (10-60% ACN/H_2_O over 50 min) and lyophilized to afford the H-LARYV-NHNH_2_ (41.1 mg, 65% yield) as a white powder.


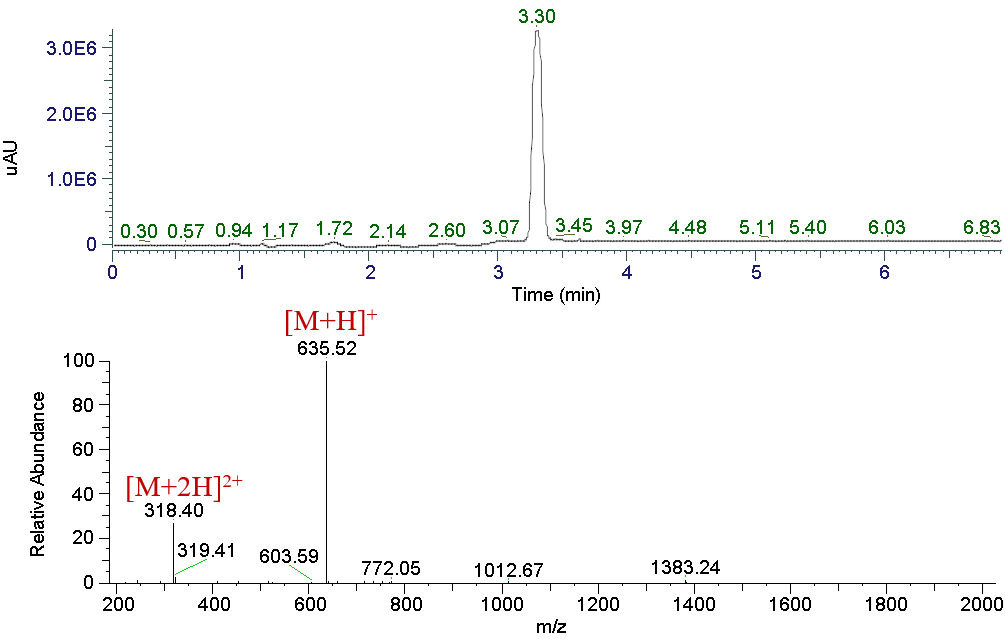


**Figure S14.** UV trace and corresponding MS from LC-MS analysis of purified H-LARYV-NHNH_2_. Gradient: 5-95% ACN/H_2_O with 0.1% TFA over 10 min at a flow rate of 0.3 mL/min. ESI-MS calcd.for C_29_H_50_N_10_O_6_ [M+H]^+^ m/z = 635.39, found 635.52; [M+2H]^2+^ m/z = 318.20, found 318.40.

4.12 H-LEAGR-NHNH_2_

H-LEAGR-NHNH_2_ was assembled according to general Fmoc-SPPS procedure (0.2 mmol). The crude peptide was purified by preparative HPLC (5-40% ACN/H_2_O over 30 min) and lyophilized to afford the H-LEAGR-NHNH_2_ (63.6 mg, 56% yield) as a white powder.


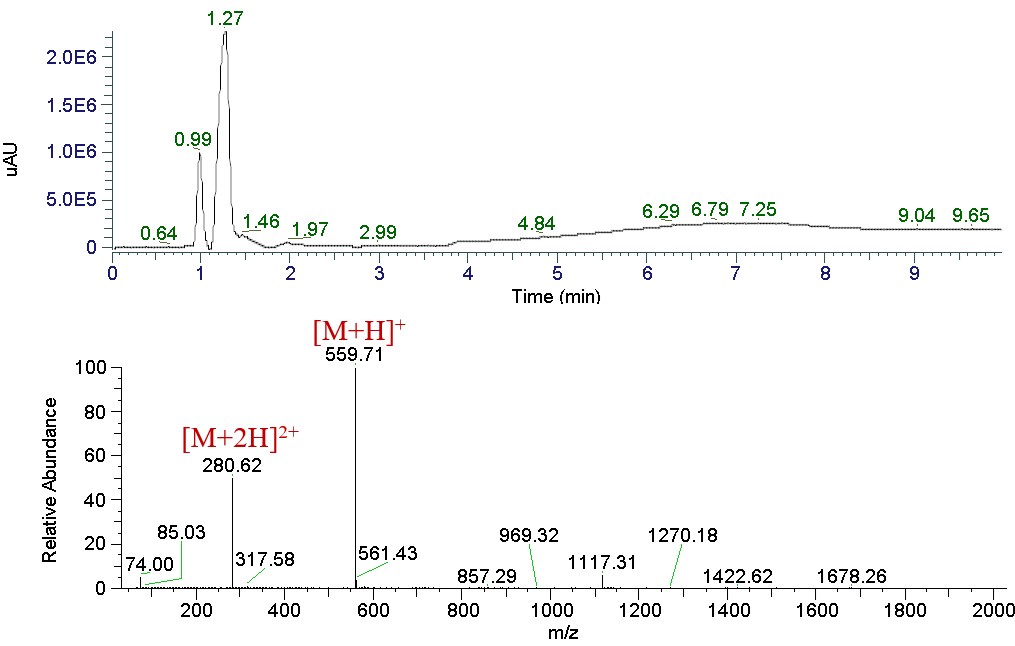


**Figure S15.** UV trace and corresponding MS from LC-MS analysis of purified H-LEAGR-NHNH_2_. Gradient: 5-95% ACN/H_2_O with 0.1% TFA over 10 min at a flow rate of 0.3 mL/min. ESI-MS calcd.for C_22_H_42_N_10_O_7_ [M+H]^+^ m/z = 559.32, found 559.71; [M+2H]^2+^ m/z = 280.16, found 280.62.

4.13 H-GDVGI-NHNH_2_

H-GDVGI-NHNH_2_ was assembled according to general Fmoc-SPPS procedure (0.1 mmol). The crude peptide was purified by preparative HPLC (10-60% ACN/H_2_O over 50 min) and lyophilized to afford the H-GDVGI-NHNH_2_ (35.8 mg, 74% yield) as a white powder.


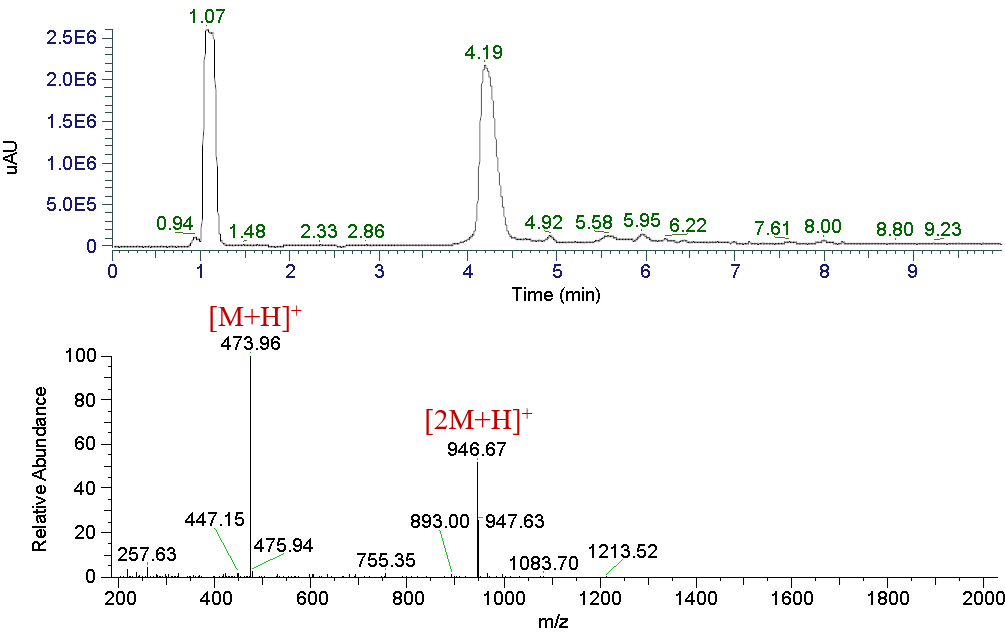


**Figure S16.** UV trace and corresponding MS from LC-MS analysis of purified H-GDVGI-NHNH_2_. Gradient: 5-95% ACN/H_2_O with 0.1% TFA over 10 min at a flow rate of 0.3 mL/min. ESI-MS calcd.for C_19_H_35_N_7_O_7_ [M+H]^+^ m/z = 474.26, found 473.96; [2M+H]^+^ m/z = 947.52, found 946.67.

4.14 H-GDVGL-NHNH_2_

H-GDVGL-NHNH_2_ was assembled according to general Fmoc-SPPS procedure (0.1 mmol). The crude peptide was purified by preparative HPLC (10-60% ACN/H_2_O over 50 min) and lyophilized to afford the H-GDVGL-NHNH_2_ (28.2 mg, 59% yield) as a white powder.


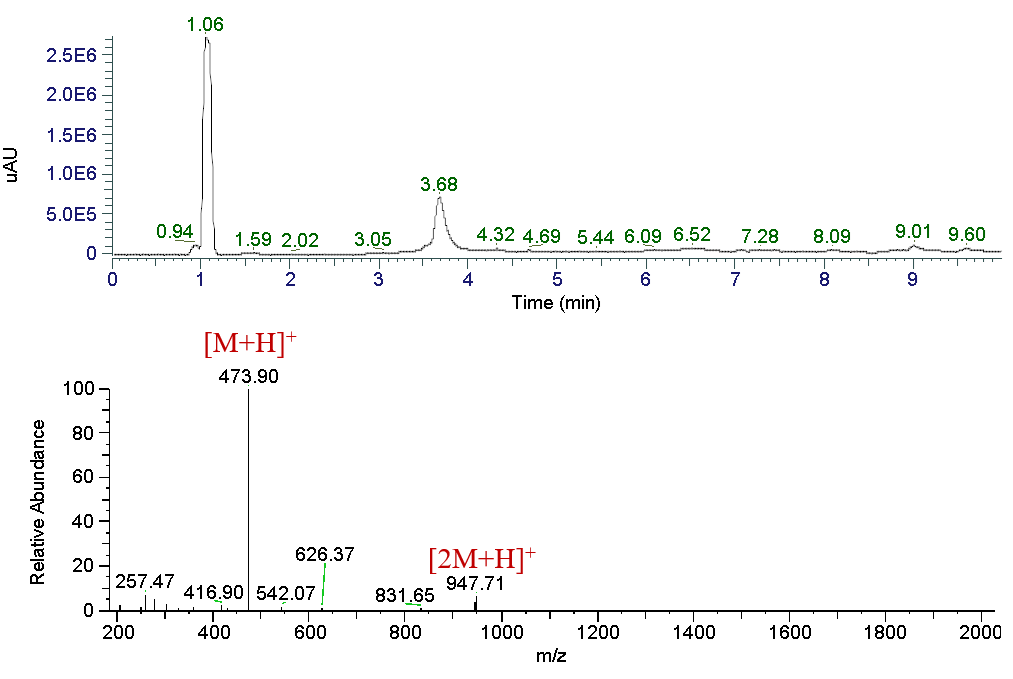


**Figure S17.** UV trace and corresponding MS from LC-MS analysis of purified H-GDVGL-NHNH_2_. Gradient: 5-60% ACN/H_2_O with 0.1% TFA over 7 min at a flow rate of 0.3 mL/min. ESI-MS calcd.for C_19_H_35_N_7_O_7_ [M+H]^+^ m/z = 474.26, found 473.90; [2M+H]^+^ m/z = 947.52, found 947.71.

4.15 H-LEAGY-NHNH_2_

H-LEAGY-NHNH_2_ was assembled according to general Fmoc-SPPS procedure (0.2 mmol). The crude peptide was purified by preparative HPLC (10-60% ACN/H_2_O over 50 min) and lyophilized to afford the H-LEAGY-NHNH_2_ (75.0 mg, 66% yield) as a white powder.


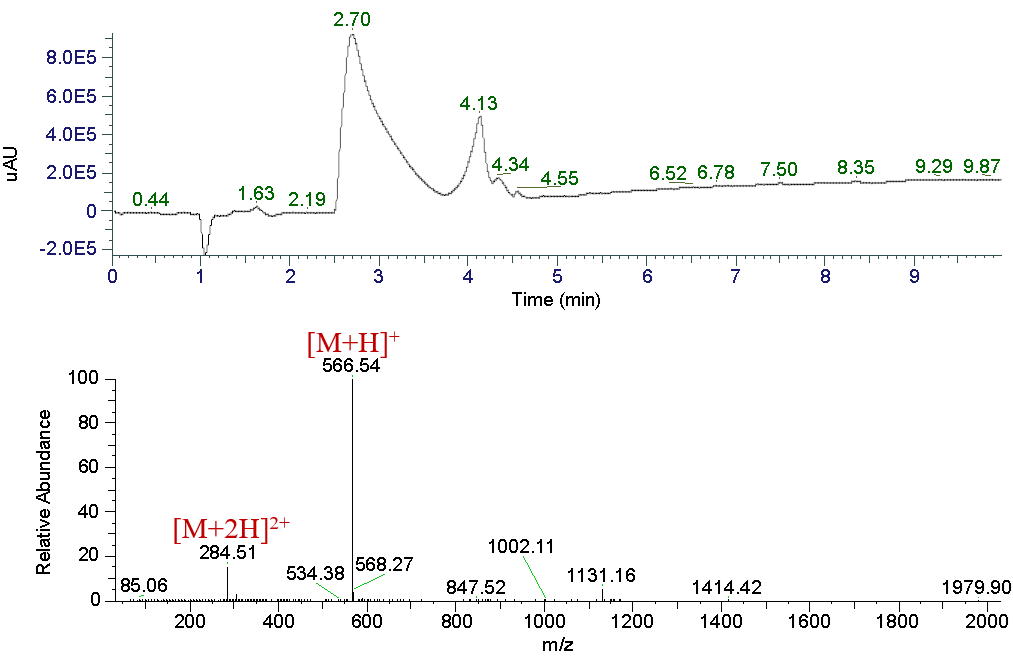


**Figure S18.** UV trace and corresponding MS from LC-MS analysis of purified H-LEAGY-NHNH_2_. Gradient: 5-95% ACN/H_2_O with 0.1% TFA over 10 min at a flow rate of 0.3 mL/min. ESI-MS calcd.for C_25_H_39_N_7_O_8_ [M+H]^+^ m/z = 566.29, found 566.54; [M+2H]^2+^ m/z = 283.65, found 284.51.

**5. Synthesis of model C-terminus peptide TSAL^SCA^ -esters**

5.1 H-LARYA-CO-TSAL^SCA^

H-LARYA-CO-TSAL^SCA^ was obtained according to **General procedure for synthesis of model C-terminus peptide TSAL^SCA^ -esters** on 40.0 mg LARYA-NHNH_2_. The crude peptide was purified by preparative HPLC (10-60% ACN/H_2_O over 50 min) and lyophilized to afford the H-LARYA-CO-TSAL^SCA^ (23.1 mg, 45% yield) as a white powder.


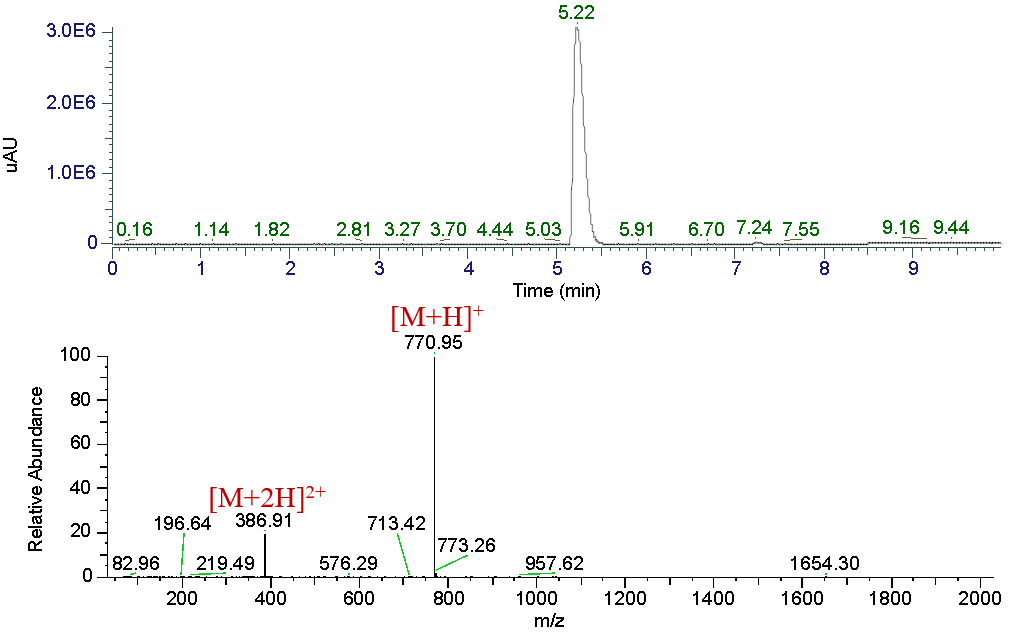


**Figure S19.** UV trace and corresponding MS from LC-MS analysis of purified H-LARYA-CO-TSAL^SCA^. Gradient: 5-95% ACN/H_2_O with 0.1% TFA over 10 min at a flow rate of 0.3 mL/min. ESI-MS calcd.for C_35_H_51_N_11_O_7_S [M+H]^+^ m/z = 770.37, found 770.95; [M+2H]^2+^ m/z = 385.69, found 386.91.

5.2 H-LARYE-CO-TSAL^SCA^

H-LARYE-CO-TSAL^SCA^ was obtained according to **General procedure for synthesis of model C-terminus peptide TSAL^SCA^ -esters** on 50.1 mg H-LARYE-NHNH_2_. The crude peptide was purified by preparative HPLC (10-60% ACN/H_2_O over 50 min) and lyophilized to afford the H-LARYE-CO-TSAL^SCA^ (21.8 mg, 35% yield) as a white powder.


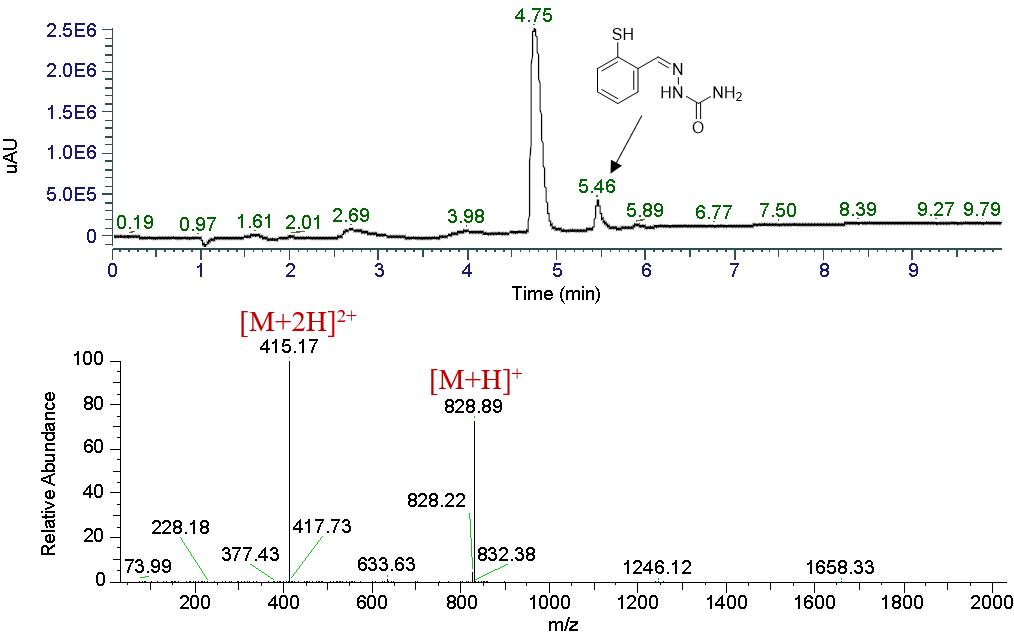


**Figure S20.** UV trace and corresponding MS from LC-MS analysis of purified H-LARYE-CO-TSAL^SCA^. Gradient: 5-95% ACN/H_2_O with 0.1% TFA over 10 min at a flow rate of 0.3 mL/min. ESI-MS calcd.for C_37_H_53_N_11_O_9_S [M+H]^+^ m/z = 828.37, found 828.89; [M+2H]^2+^ m/z = 414.69, found 415.17.

5.3 H-LARYF-CO-TSAL^SCA^

H-LARYF-CO-TSAL^SCA^ was obtained according to **General procedure for synthesis of model C-terminus peptide TSAL^SCA^ -esters** on 40.2 mg H-LARYF-NHNH_2_. The crude peptide was purified by preparative HPLC (10-60% ACN/H_2_O over 50 min) and lyophilized to afford the H-LARYF-CO-TSAL^SCA^ (16.4 mg, 33% yield) as a white powder.


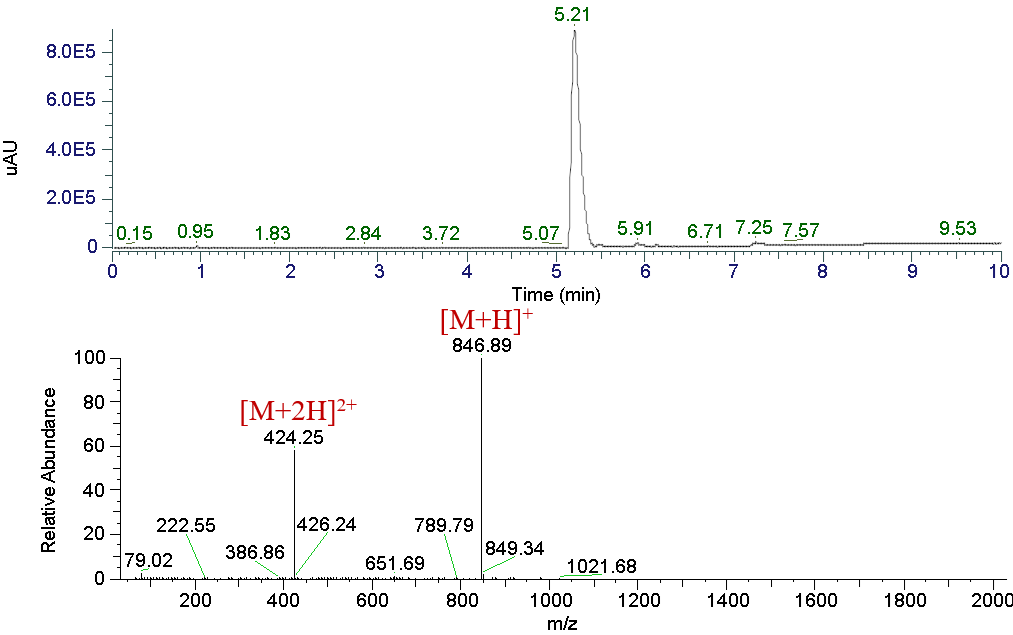


**Figure S21.** UV trace and corresponding MS from LC-MS analysis of purified H-LARYF-CO-TSAL^SCA^. Gradient: 5-95% ACN/H_2_O with 0.1% TFA over 10 min at a flow rate of 0.3 mL/min. ESI-MS calcd.for C_41_H_55_N_11_O_7_S [M+H]^+^ m/z = 846.40, found 846.89; [M+2H]^2+^ m/z = 423.70, found 424.25.

5.4 H-LARYG-CO-TSAL^SCA^

H-LARYG-CO-TSAL^SCA^ was obtained according to **General procedure for synthesis of model C-terminus peptide TSAL^SCA^ -esters** on 60.0 mg H-LARYG-NHNH_2_. The crude peptide was purified by preparative HPLC (10-60% ACN/H_2_O over 50 min) and lyophilized to afford the H-LARYG-CO-TSAL^SCA^ (36.2 mg, 48% yield) as a white powder.


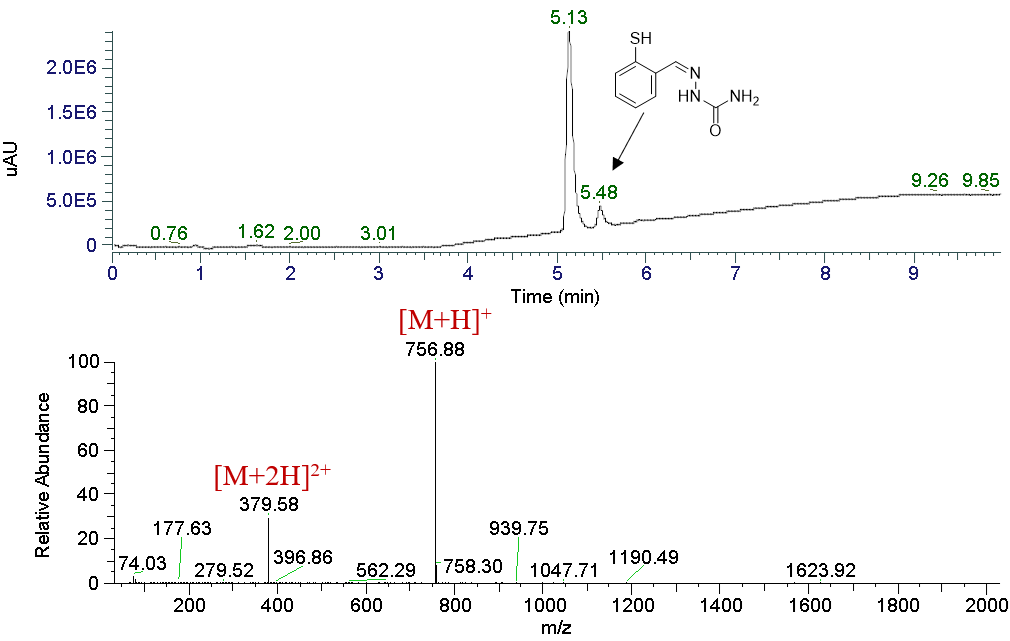


**Figure S22.** UV trace and corresponding MS from LC-MS analysis of purified H-LARYG-CO-TSAL^SCA^. Gradient: 5-95% ACN/H_2_O with 0.1% TFA over 10 min at a flow rate of 0.3 mL/min. ESI-MS calcd.for C_34_H_49_N_11_O_7_S [M+H]^+^ m/z = 756.35, found 756.88; [M+2H]^2+^ m/z = 378.68, found 379.58.

5.5 H-LARYH-CO-TSAL^SCA^

H-LARYH-CO-TSAL^SCA^ was obtained according to **General procedure for synthesis of model C-terminus peptide TSAL^SCA^ -esters** on 86.1 mg H-LARYH-NHNH_2_. The crude peptide was purified by preparative HPLC (10-60% ACN/H_2_O over 50 min) and lyophilized to afford the H-LARYH-CO-TSAL^SCA^ (28.0 mg, 26% yield) as a white powder.


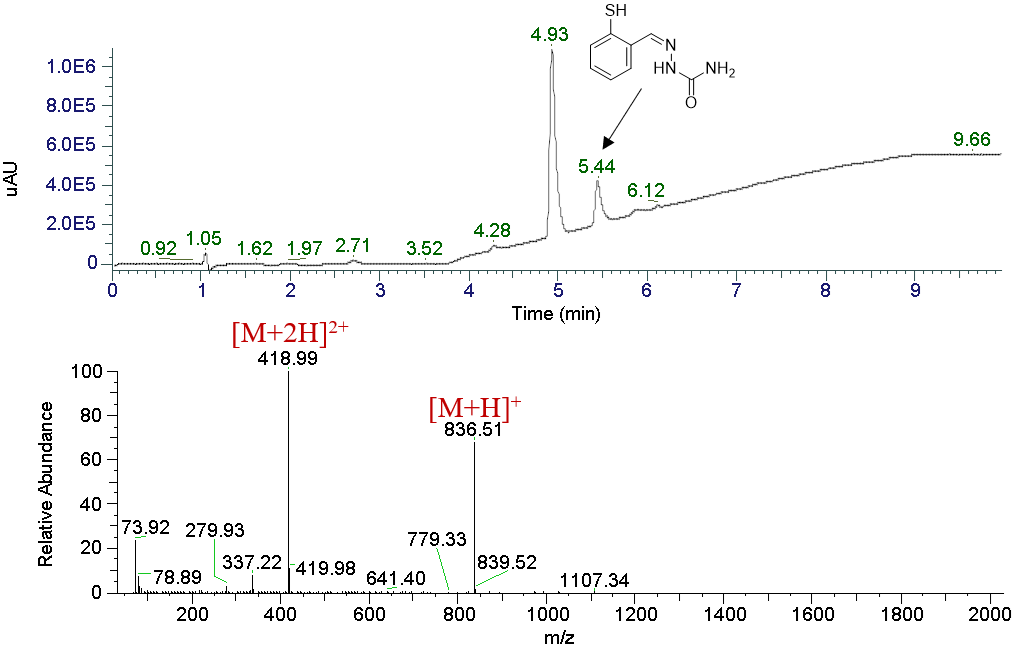


**Figure S23.** UV trace and corresponding MS from LC-MS analysis of purified H-LARYH-CO-TSAL^SCA^. Gradient: 5-95% ACN/H_2_O with 0.1% TFA over 10 min at a flow rate of 0.3 mL/min. ESI-MS calcd.for C_38_H_53_N_13_O_7_S [M+H]^+^ m/z = 836.39, found 836.51; [M+2H]^2+^ m/z = 418.70, found 418.99.

5.6 H-LARYK-CO-TSAL^SCA^

H-LARYK-CO-TSAL^SCA^ was obtained according to **General procedure for synthesis of model C-terminus peptide TSAL^SCA^ -esters** on 107.0 mg H-LARYK-NHNH_2_. The crude peptide was purified by preparative HPLC (10-60% ACN/H_2_O over 50 min) and lyophilized to afford the H-LARYK-CO-TSAL^SCA^ (73.0 mg, 55% yield) as a white powder.


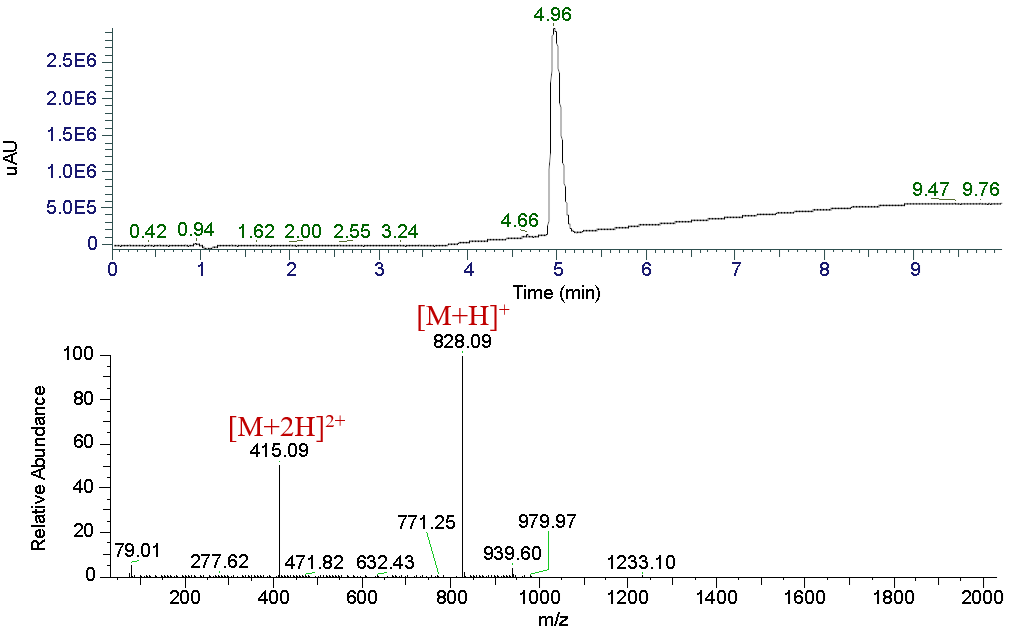


**Figure S24.** UV trace and corresponding MS from LC-MS analysis of purified H-LARYK-CO-TSAL^SCA^. Gradient: 5-95% ACN/H_2_O with 0.1% TFA over 10 min at a flow rate of 0.3 mL/min. ESI-MS calcd.for C_38_H_58_N_12_O_7_S [M+H]^+^ m/z = 827.43, found 828.09; [M+2H]^2 +^m/z = 414.22, found 415.09.

5.7 H-LARYM-CO-TSAL^SCA^

H-LARYM-CO-TSAL^SCA^ was obtained according to **General procedure for synthesis of model C-terminus peptide TSAL^SCA^ -esters** on 55.0 mg H-LARYM-NHNH_2_. The crude peptide was purified by preparative HPLC (10-60% ACN/H_2_O over 50 min) and lyophilized to afford the H-LARYM-CO-TSAL^SCA^ (30.5 mg, 45% yield) as a white powder.


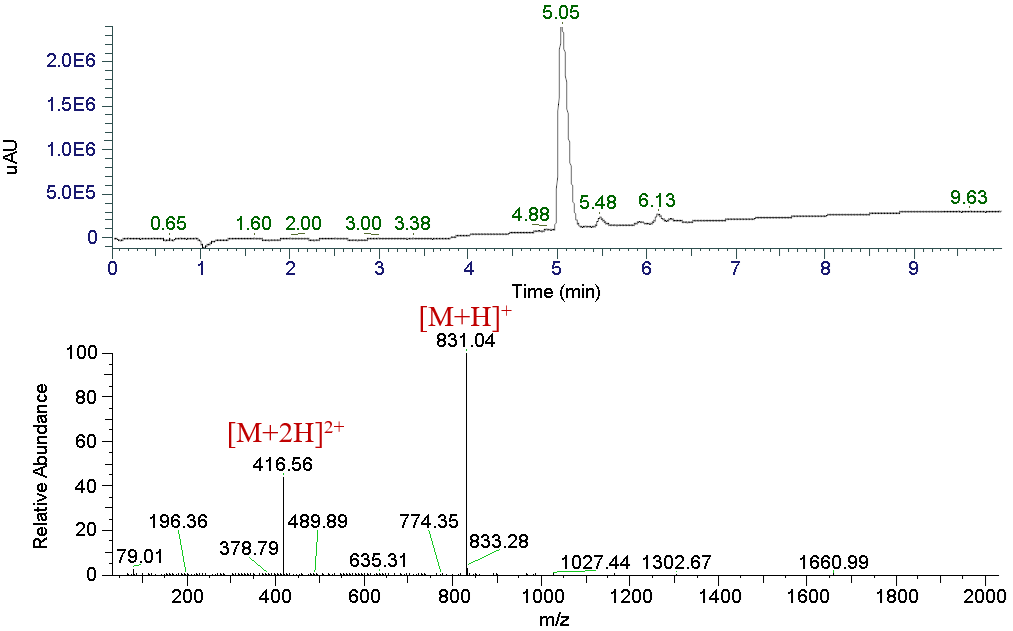


**Figure S25.** UV trace and corresponding MS from LC-MS analysis of purified H-LARYM-CO-TSAL^SCA^. Gradient: 5-95% ACN/H_2_O with 0.1% TFA over 10 min at a flow rate of 0.3 mL/min. ESI-MS calcd.for C_37_H_55_N_11_O_7_S_2_ [M+H]^+^ m/z = 830.37, found 831.04; [M+2H]^2+^ m/z = 415.69, found 416.56.

5.8 H-LARYS-CO-TSAL^SCA^

H-LARYS-CO-TSAL^SCA^ was obtained according to **General procedure for synthesis of model C-terminus peptide TSAL^SCA^ -esters** on 80.0 mg H-LARYS-NHNH_2_. The crude peptide was purified by preparative HPLC (10-60% ACN/H_2_O over 50 min) and lyophilized to afford the H-LARYS-CO-TSAL^SCA^ (54.9 mg, 54% yield) as a white powder.


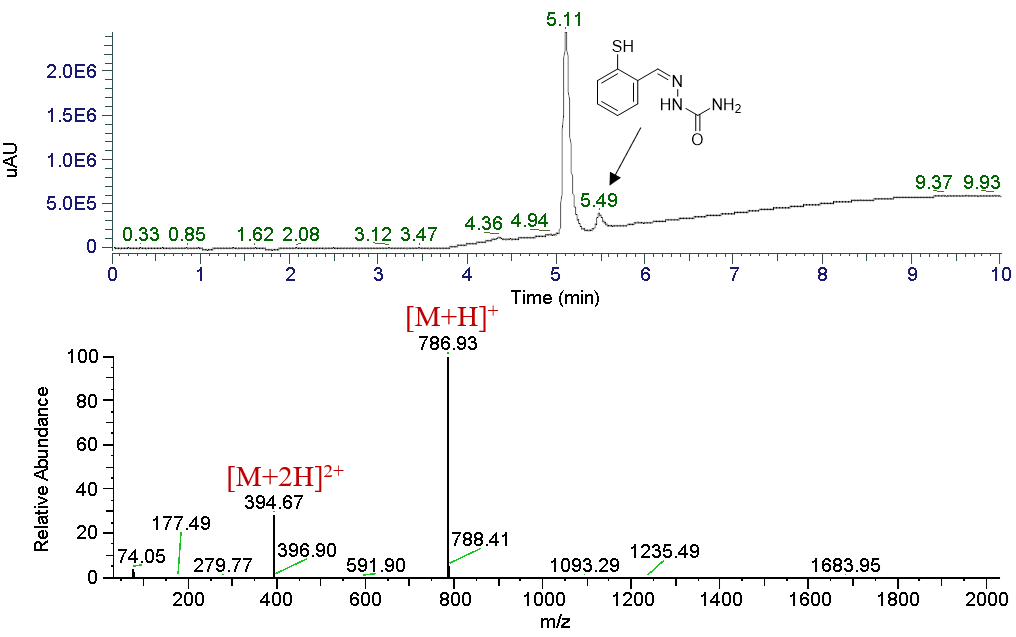


**Figure S26.** UV trace and corresponding MS from LC-MS analysis of purified H-LARYS-CO-TSAL^SCA^. Gradient: 5-95% ACN/H_2_O with 0.1% TFA over 10 min at a flow rate of 0.3 mL/min. ESI-MS calcd.for C_35_H_51_N_11_O_8_S [M+H]^+^ m/z = 786.36, found 786.93; [M+2H]^2+^ m/z=393.68, found 394.67.

5.9 H-LARYT-CO-TSAL^SCA^

H-LARYT-CO-TSAL^SCA^ was obtained according to **General procedure for synthesis of model C-terminus peptide TSAL^SCA^ -esters** on 30.1 mg H-LARYT-NHNH_2_. The crude peptide was purified by preparative HPLC (10-60% ACN/H_2_O over 50 min) and lyophilized to afford the H-LARYT-CO-TSAL^SCA^ (22.9 mg, 61% yield) as a white powder.


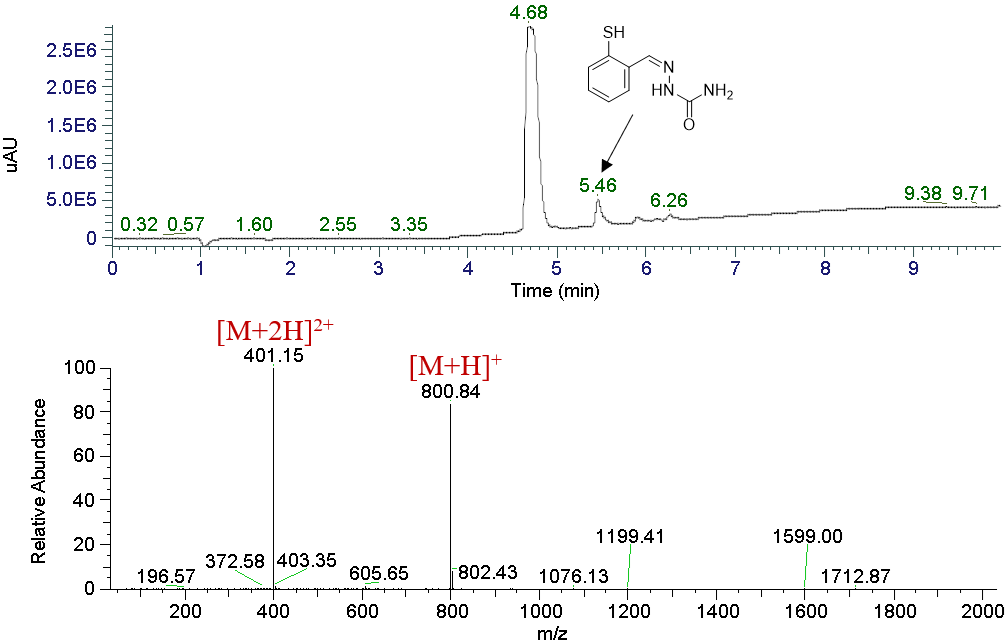


**Figure S27.** UV trace and corresponding MS from LC-MS analysis of purified H-LARYT-CO-TSAL^SCA^. Gradient: 5-95% ACN/H_2_O with 0.1% TFA over 10 min at a flow rate of 0.3 mL/min. ESI-MS calcd.for C_36_H_53_N_11_O_8_S [M+H]^+^ m/z = 800.38, found 800.84; [M+2H]^2+^ m/z = 400.69, found 401.15.

5.10 H-LARYP-CO-TSAL^SCA^

H-LARYP-CO-TSAL^SCA^ was obtained according to **General procedure for synthesis of model C-terminus peptide TSAL^SCA^ -esters** on 90.0 mg H-LARYP-NHNH_2_. The crude peptide was purified by preparative HPLC (10-60% ACN/H_2_O over 50 min) and lyophilized to afford the H-LARYP-CO-TSAL^SCA^ (57.1 mg, 50% yield) as a white powder.

^
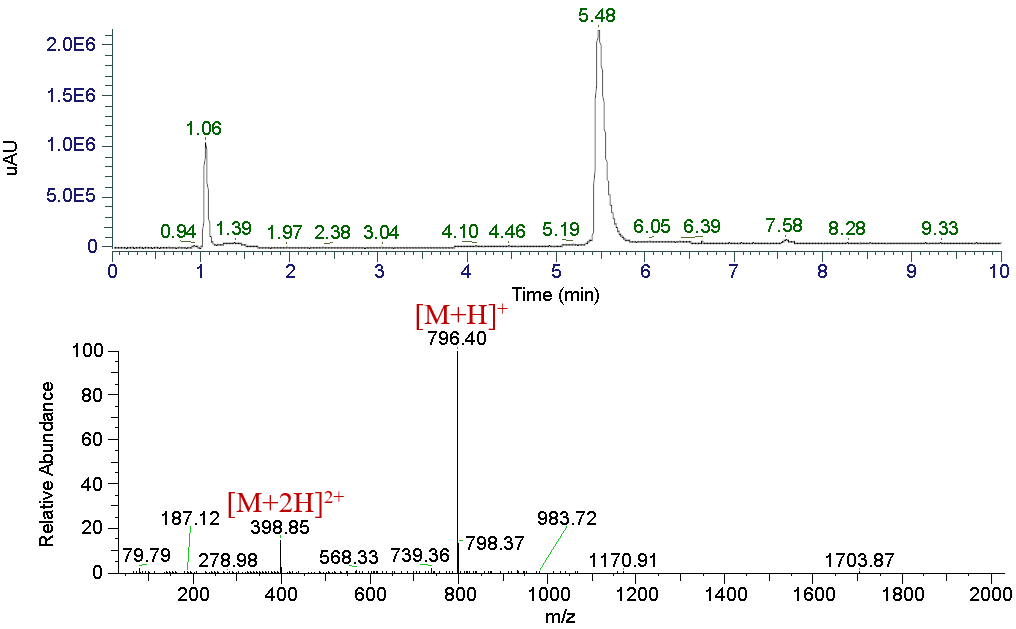
^

**Figure S28.** UV trace and corresponding MS from LC-MS analysis of purified H-LARYP-CO-TSAL^SCA^. Gradient: 5-95% ACN/H_2_O with 0.1% TFA over 10 min at a flow rate of 0.3 mL/min. ESI-MS calcd.for C_37_H_53_N_11_O_7_S [M+H]^+^ m/z = 796.39, found 796.40; [M+2H]^2+^ m/z = 398.70, found 398.85.

5.11 H-LARYV-CO-TSAL^SCA^

H-LARYV-CO-TSAL^SCA^ was obtained according to **General procedure for synthesis of model C-terminus peptide TSAL^SCA^ -esters** on 44.3 mg H-LARYV-NHNH_2_. The crude peptide was purified by preparative HPLC (10-60% ACN/H_2_O over 50 min) and lyophilized to afford the H-LARYV-CO-TSAL^SCA^ (16.7 mg, 30% yield) as a white powder.


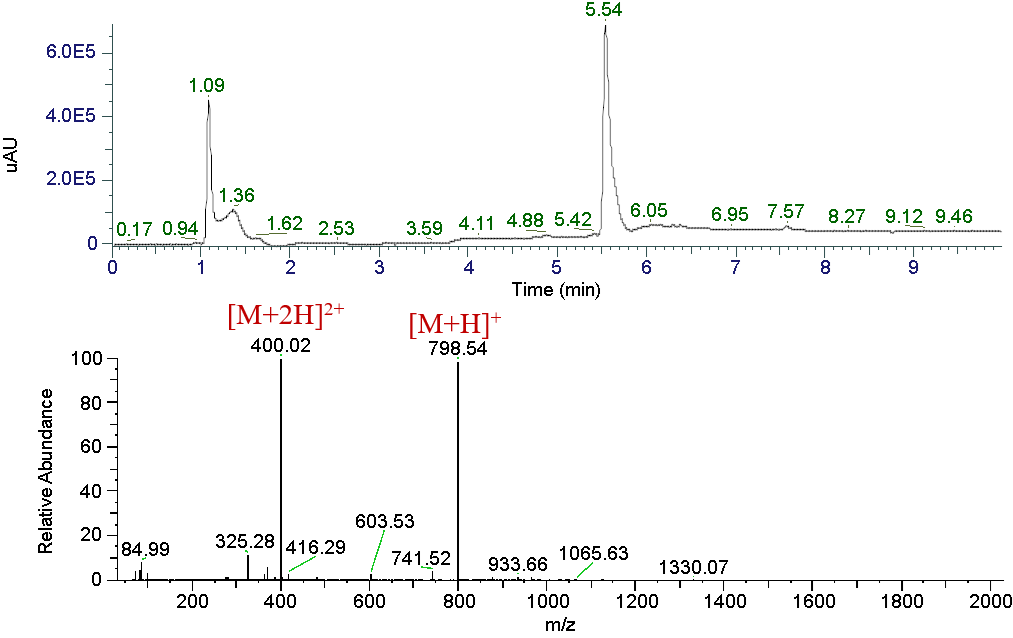


**Figure S29.** UV trace and corresponding MS from LC-MS analysis of purified H-LARYV-CO-TSAL^SCA^. Gradient: 5-95% ACN/H_2_O with 0.1% TFA over 10 min at a flow rate of 0.3 mL/min. ESI-MS calcd.for C_37_H_55_N_11_O_7_S [M+H]^+^ m/z = 798.40, found 798.54; [M+2H]^2+^ m/z = 399.70, found 400.02.

5.12 H-LEAGR-CO-TSAL^SCA^

H-LEAGR-CO-TSAL^SCA^ was obtained according to **General procedure for synthesis of model C-terminus peptide TSAL^SCA^ -esters** on 50.2 mg H-LEAGR-NHNH_2_. The crude peptide was purified by preparative HPLC (10-60% ACN/H_2_O over 50 min) and lyophilized to afford the H-LEAGR-CO-TSAL^SCA^ (24.6 mg, 38% yield) as a white powder.


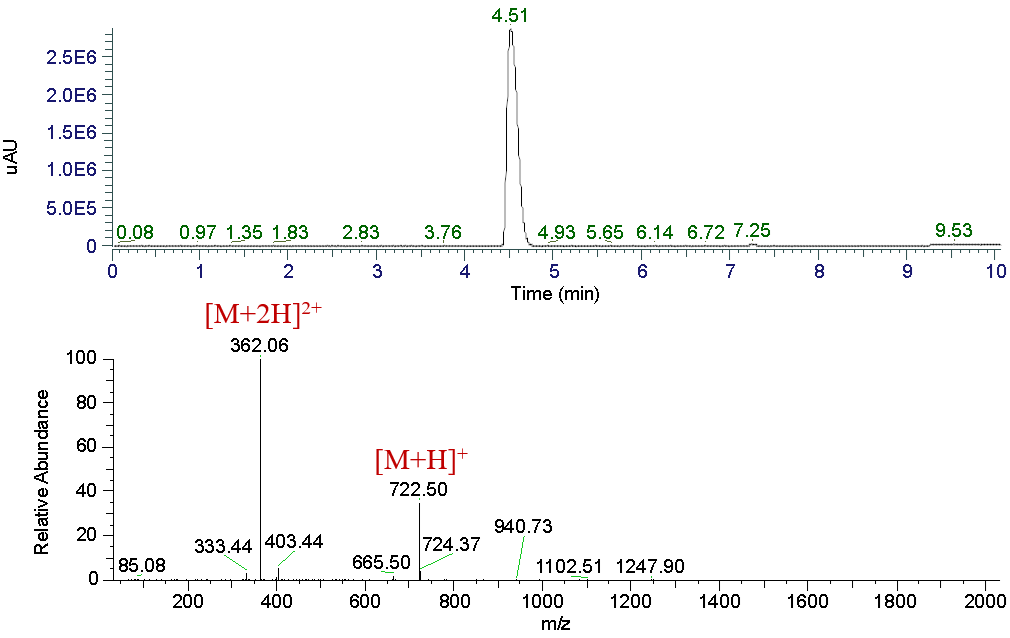


**Figure S30.** UV trace and corresponding MS from LC-MS analysis of purified H-LEAGR-CO-TSAL^SCA^. Gradient: 5-95% ACN/H_2_O with 0.1% TFA over 10 min at a flow rate of 0.3 mL/min. ESI-MS calcd.for C_30_H_47_N_11_O_8_S [M+H]^+^ m/z = 722.33, found 722.50; [M+2H]^2+^ m/z = 361.67, found 362.06.

5.13 H-GDVGI-CO-TSAL^SCA^

H-GDVGI-CO-TSAL^SCA^ was obtained according to **General procedure for synthesis of model C-terminus peptide TSAL^SCA^ -esters** on 49.0 mg H-GDVGI-NHNH_2_. The crude peptide was purified by preparative HPLC (10-60% ACN/H_2_O over 50 min) and lyophilized to afford the H-GDVGI-CO-TSAL^SCA^ (22.2 mg, 34% yield) as a white powder.


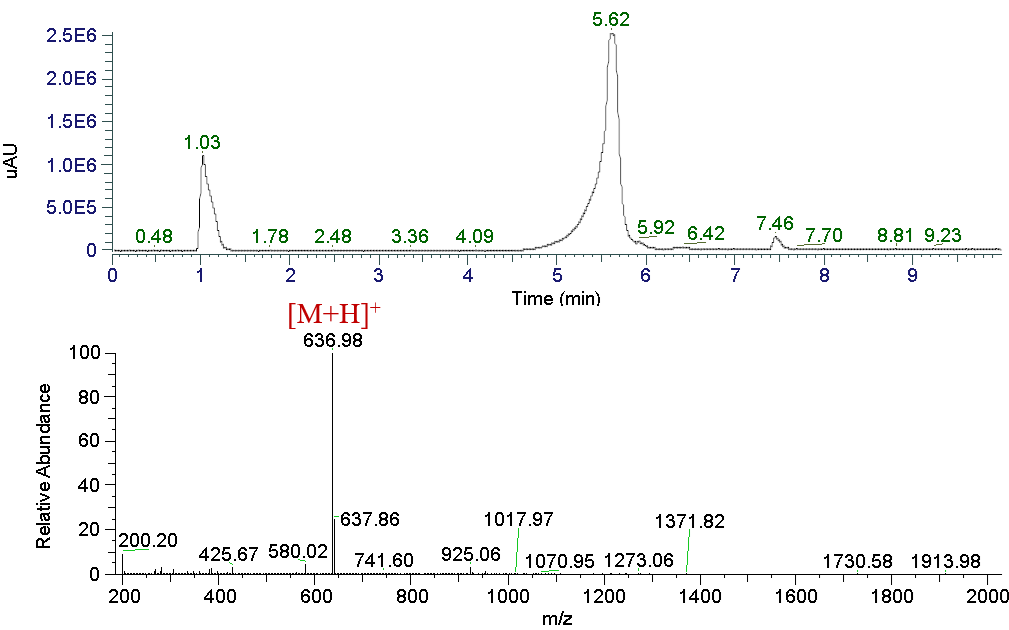


**Figure S31.** UV trace and corresponding MS from LC-MS analysis of purified H-GDVGI-CO-TSAL^SCA^. Gradient: 5-95% ACN/H_2_O with 0.1% TFA over 10 min at a flow rate of 0.3 mL/min. ESI-MS calcd.for C_27_H_40_N_8_O_8_S [M+H]^+^ m/z = 637.27, found 636.98.

5.14 H-GDVGL-CO-TSAL^SCA^

H-GDVGL-CO-TSAL^SCA^ was obtained according to **General procedure for synthesis of model C-terminus peptide TSAL^SCA^ -esters** on 10.0 mg H-GDVGL-NHNH_2_. The crude peptide was purified by preparative HPLC (10-60% ACN/H_2_O over 50 min) and lyophilized to afford the H-GDVGL-CO-TSAL^SCA^ (5.4 mg, 40% yield) as a white powder.


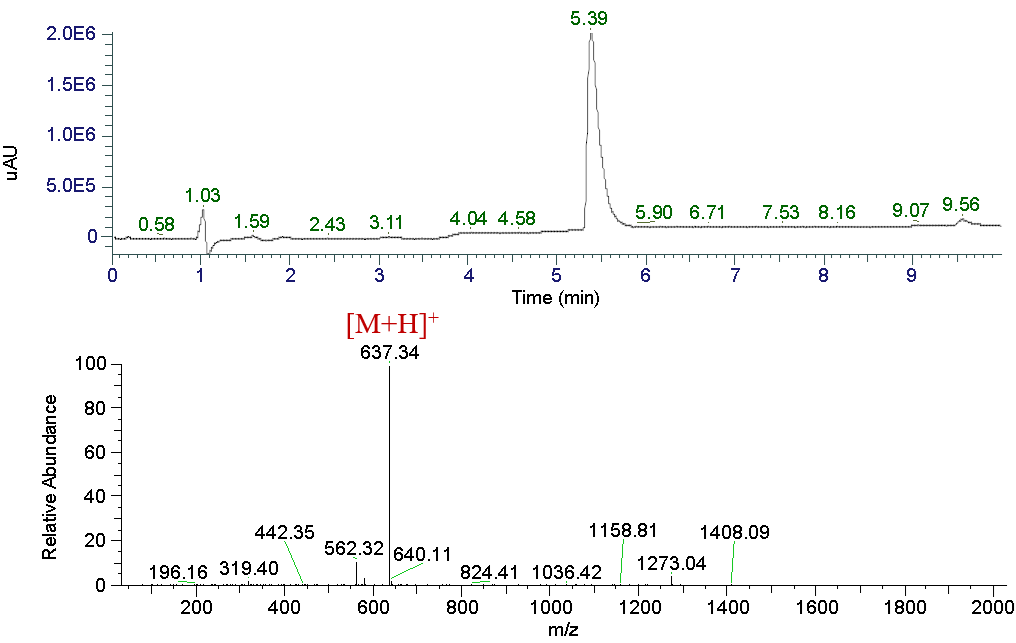


**Figure S32.** UV trace and corresponding MS from LC-MS analysis of purified H-GDVGL-CO-TSAL^SCA^. Gradient: 5-95% ACN/H_2_O with 0.1% TFA over 10 min at a flow rate of 0.3 mL/min. ESI-MS calcd.for C_27_H_40_N_8_O_8_S [M+H]^+^ m/z = 637.27, found 637.34.

5.15 H-LEAGY-CO-TSAL^SCA^

H-LEAGY-CO-TSAL^SCA^ was obtained according to **General procedure for synthesis of model C-terminus peptide TSAL^SCA^ -esters** on 60.0 mg H-LEAGY-NHNH_2_. The crude peptide was purified by preparative HPLC (10-60% ACN/H_2_O over 50 min) and lyophilized to afford the H-LEAGY-CO-TSAL^SCA^ (30.5 mg, 39% yield) as a white powder.


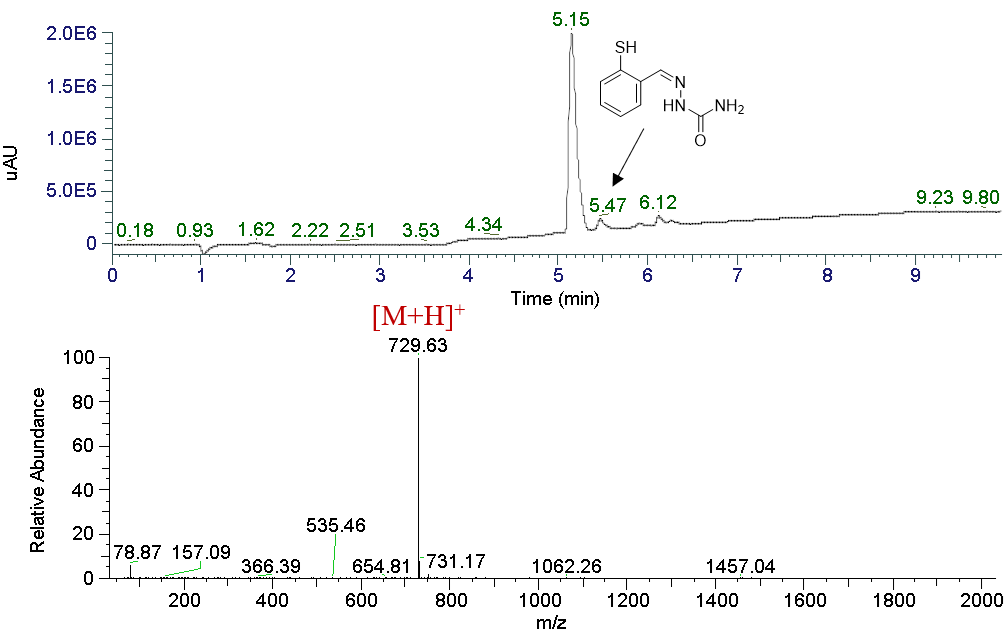


**Figure S33.** UV trace and corresponding MS from LC-MS analysis of purified H-LEAGY-CO-TSAL^SCA^. Gradient: 5-95% ACN/H_2_O with 0.1% TFA over 10 min at a flow rate of 0.3 mL/min. ESI-MS calcd.for C_33_H_44_N_8_O_9_S [M+H]^+^ m/z = 729.30, found 729.63.

**6. Synthesis of model C-terminus peptide TSAL-esters**

6.1 H-LARYA-CO-TSAL

H-LARYA-CO-TSAL was obtained according to **Pyruvic acid treatment of peptide TSAL^SCA^-esters for C-terminus peptide TSAL-esters** on 20.1 mg H-LARYA-CO-TSAL^SCA^. The crude peptide was purified by preparative HPLC (10-60% ACN/H_2_O over 50 min) and lyophilized to afford the H-LARYA-CO-TSAL (16.4 mg, 89% yield) as a white powder.


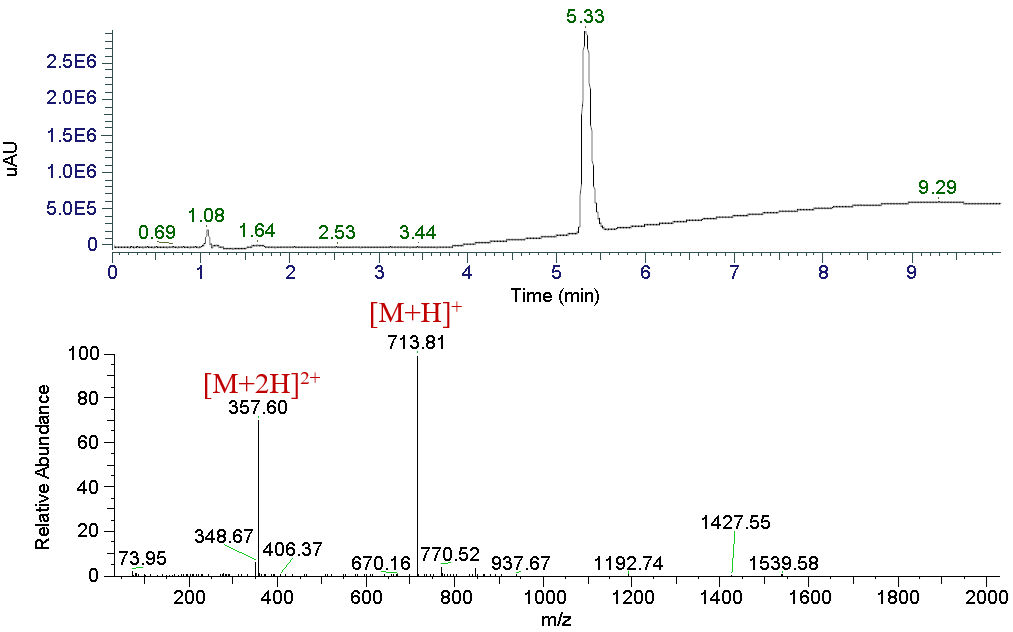


**Figure S34.** UV trace and corresponding MS from LC-MS analysis of purified H-LARYA-CO-TSAL. Gradient: 5-95% ACN/H_2_O with 0.1% TFA over 10 min at a flow rate of 0.3 mL/min. ESI-MS calcd.for C_34_H_48_N_8_O_7_S [M+H]^+^ m/z = 713.34, found 713.81; [M+2H]^2+^ m/z = 357.17, found 357.60.

6.2 H-LARYE-CO-TSAL

H-LARYE-CO-TSAL was obtained according to **Pyruvic acid treatment of peptide TSAL^SCA^-esters for C-terminus peptide TSAL-esters** on 21.0 mg H-LARYE-CO-TSAL^SCA^. The crude peptide was purified by preparative HPLC (10-60% ACN/H_2_O over 50 min) and lyophilized to afford the H-LARYE-CO-TSAL (14.3 mg, 73% yield) as a white powder.


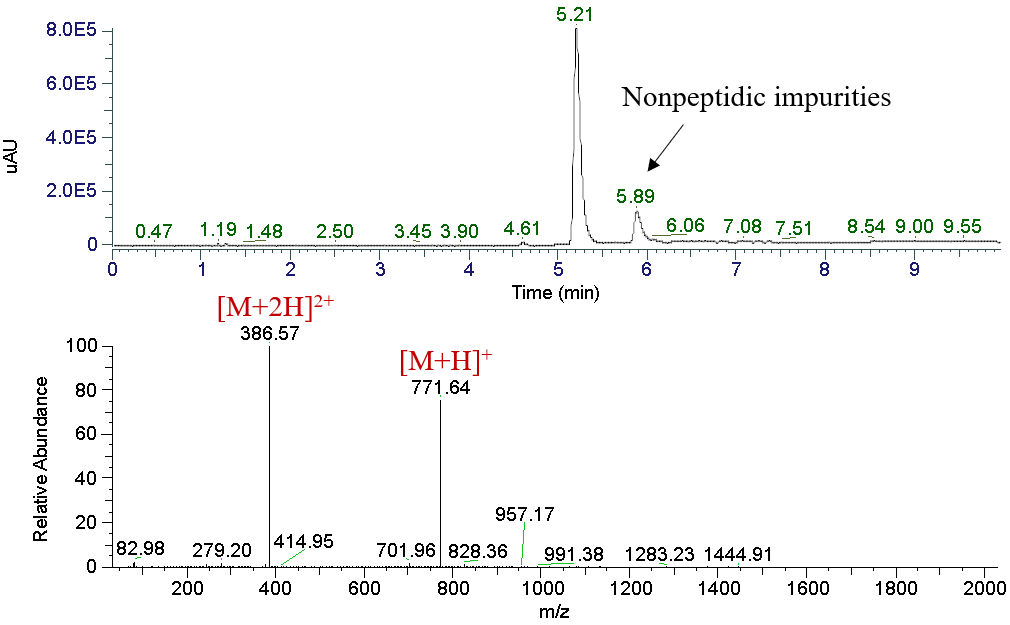


**Figure S35** UV trace and corresponding MS from LC-MS analysis of purified H-LARYE-CO-TSAL. Gradient: 5-95% ACN/H_2_O with 0.1% TFA over 10 min at a flow rate of 0.3 mL/min. ESI-MS calcd.for C_36_H_50_N_8_O_9_S [M+H]^+^ m/z = 771.34, found 771.64; [M+2H]^2+^ m/z = 386.17, found 386.57.

6.3 H-LARYF-CO-TSAL

H-LARYF-CO-TSAL was obtained according to **Pyruvic acid treatment of peptide TSAL^SCA^-esters for C-terminus peptide TSAL-esters** on 20.0 mg H-LARYF-CO-TSAL^SCA^. The crude peptide was purified by preparative HPLC (10-60% ACN/H_2_O over 50 min) and lyophilized to afford the H-LARYF-CO-TSAL (15.5 mg, 83% yield) as a white powder.


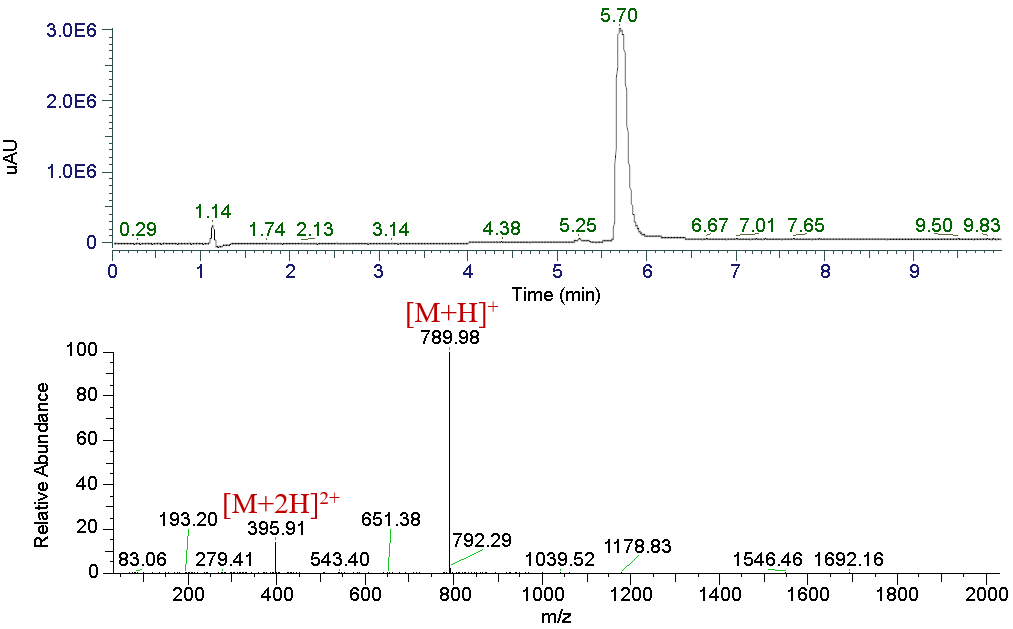


**Figure S36.** UV trace and corresponding MS from LC-MS analysis of purified H-LARYF-CO-TSAL. Gradient: 5-95% ACN/H_2_O with 0.1% TFA over 10 min at a flow rate of 0.3 mL/min. ESI-MS calcd.for C_40_H_52_N_8_O_7_S [M+H]^+^ m/z = 789.37, found 789.98; [M+2H]^2+^ m/z = 395.19, found 395.91.

6.4 H-LARYG-CO-TSAL

H-LARYG-CO-TSAL was obtained according to **Pyruvic acid treatment of peptide TSAL^SCA^-esters for C-terminus peptide TSAL-esters** on 10.0 mg H-LARYG-CO-TSAL^SCA^. The crude peptide was purified by preparative HPLC (10-60% ACN/H_2_O over 50 min) and lyophilized to afford the H-LARYG-CO-TSAL (5.9 mg, 64% yield) as a white powder.


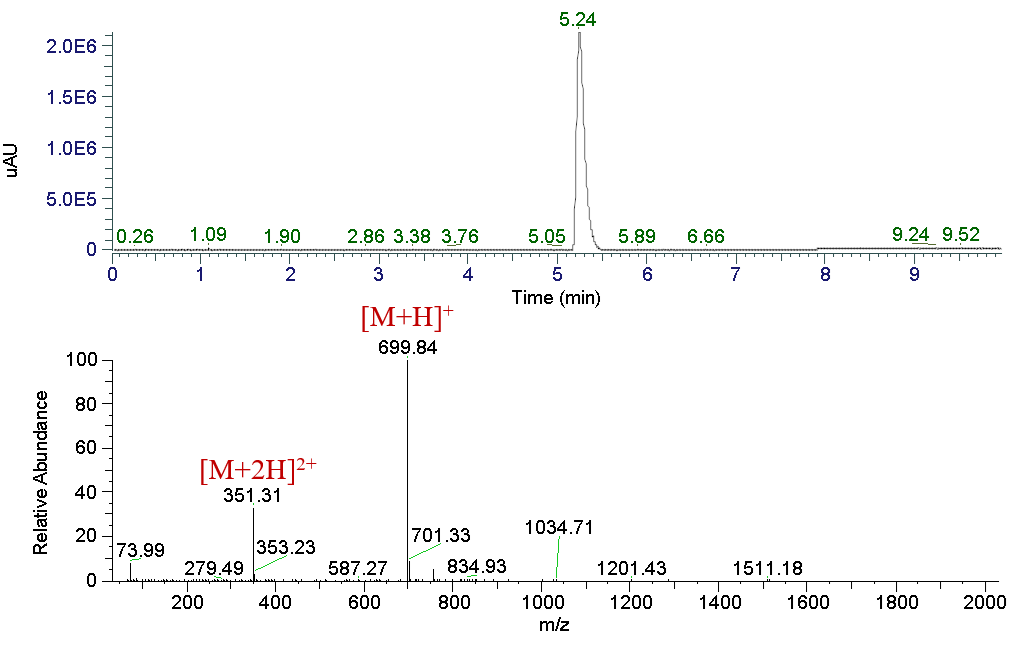


**Figure S37.** UV trace and corresponding MS from LC-MS analysis of purified H-LARYG-CO-TSAL. Gradient: 5-95% ACN/H_2_O with 0.1% TFA over 10 min at a flow rate of 0.3 mL/min. ESI-MS calcd.for C_33_H_46_N_8_O_7_S [M+H]^+^ m/z = 699.32, found 699.84; [M+2H]^2+^ m/z = 350.16, found 351.31.

6.5 H-LARYH-CO-TSAL

H-LARYH-CO-TSAL was obtained according to **Pyruvic acid treatment of peptide TSAL^SCA^-esters for C-terminus peptide TSAL-esters** on 10.0 mg H-LARYH-CO-TSAL^SCA^. The crude peptide was purified by preparative HPLC (10-60% ACN/H_2_O over 50 min) and lyophilized to afford the H-LARYH-CO-TSAL (5.8 mg, 62% yield) as a white powder.


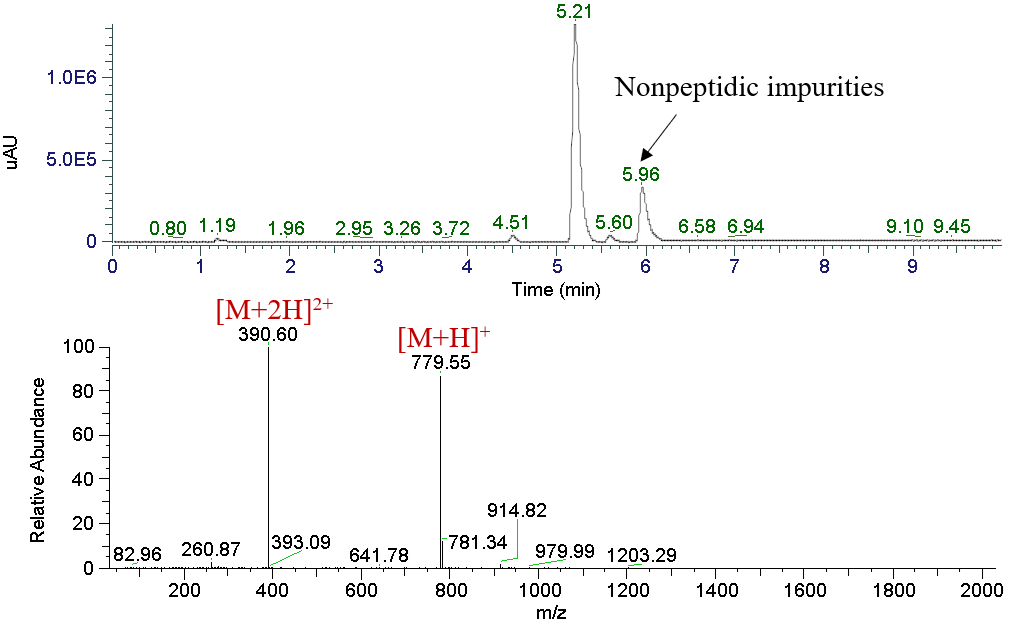


**Figure S38.** UV trace and corresponding MS from LC-MS analysis of purified H-LARYH-CO-TSAL. Gradient: 5-95% ACN/H_2_O with 0.1% TFA over 10 min at a flow rate of 0.3 mL/min. ESI-MS calcd.for C_37_H_50_N_10_O_7_S [M+H]^+^ m/z = 779.36, found 779.55; [M+2H]^2+^ m/z = 390.18, found 390.60.

6.6 H-LARYK-CO-TSAL

H-LARYK-CO-TSAL was obtained according to **Pyruvic acid treatment of peptide TSAL^SCA^-esters for C-terminus peptide TSAL-esters** on 17.6 mg H-LARYK-CO-TSAL^SCA^. The crude peptide was purified by preparative HPLC (10-60% ACN/H_2_O over 50 min) and lyophilized to afford the H-LARYK-CO-TSAL (13.1 mg, 78% yield) as a white powder.


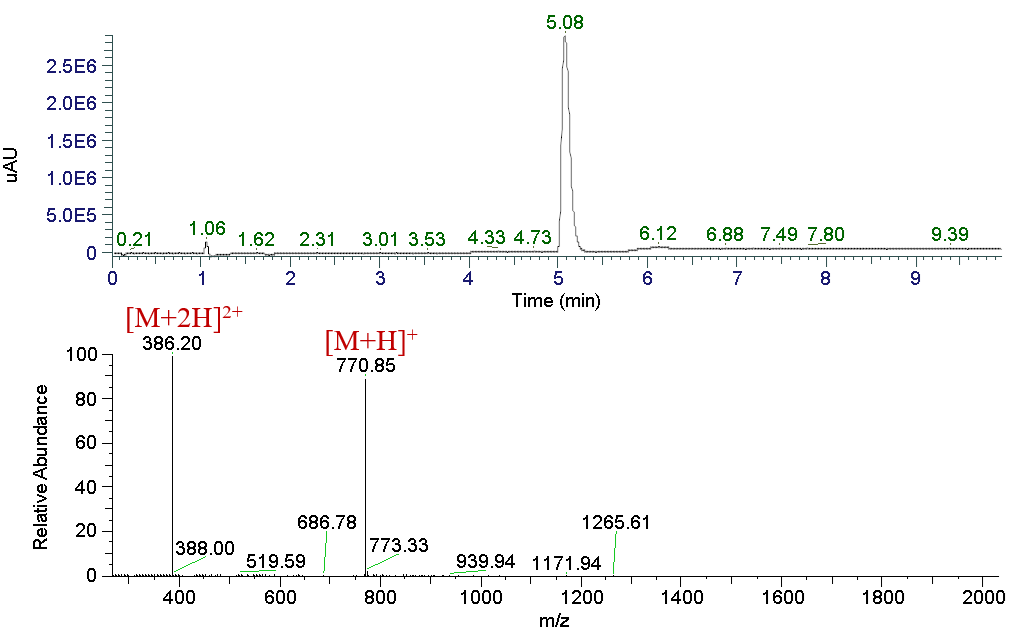


**Figure S39.** UV trace and corresponding MS from LC-MS analysis of purified H-LARYK-CO-TSAL. Gradient: 5-95% ACN/H_2_O with 0.1% TFA over 10 min at a flow rate of 0.3 mL/min. ESI-MS calcd.for C_37_H_55_N_9_O_7_S [M+H]^+^ m/z = 770.39, found 770.85; [M+2H]^2+^ m/z = 385.70, found 386.20.

6.7 H-LARYM-CO-TSAL

H-LARYM-CO-TSAL was obtained according to **Pyruvic acid treatment of peptide TSAL^SCA^-esters for C-terminus peptide TSAL-esters** on 15.0 mg H-LARYM-CO-TSAL^SCA^. The crude peptide was purified by preparative HPLC (10-60% ACN/H_2_O over 50 min) and lyophilized to afford the H-LARYM-CO-TSAL (9.4 mg, 67% yield) as a white powder.


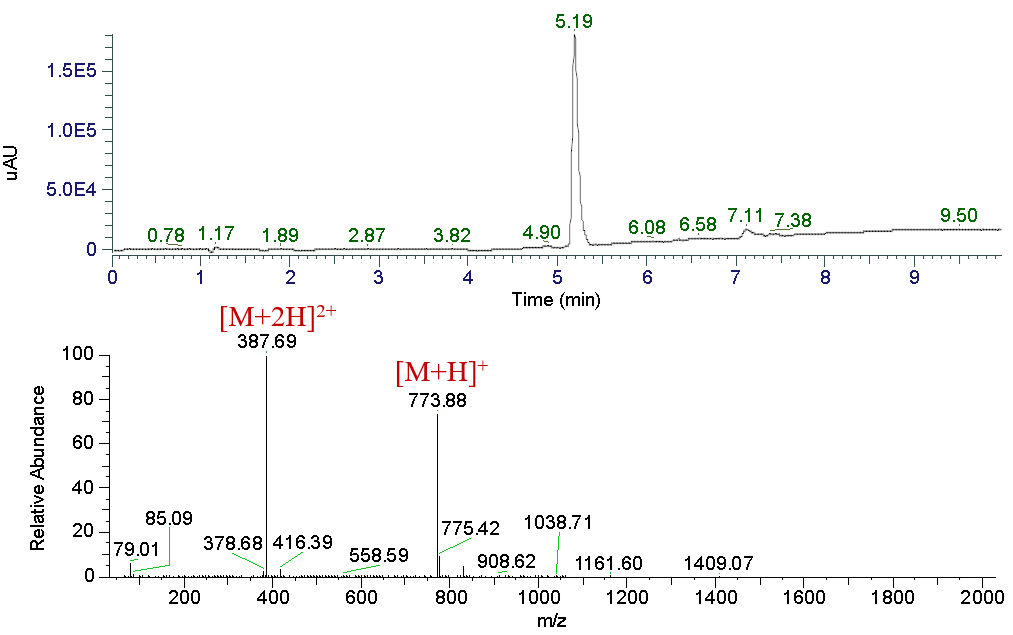


**Figure S40.** UV trace and corresponding MS from LC-MS analysis of purified H-LARYM-CO-TSAL. Gradient: 5-95% ACN/H_2_O with 0.1% TFA over 10 min at a flow rate of 0.3 mL/min. ESI-MS calcd.for C_36_H_52_N_8_O_7_S_2_ [M+H]^+^ m/z = 773.34, found 773.88; [M+2H]^2+^ m/z = 387.17, found 387.69.

6.8 H-LARYS-CO-TSAL

H-LARYS-CO-TSAL was obtained according to **Pyruvic acid treatment of peptide TSAL^SCA^-esters for C-terminus peptide TSAL-esters** on 10.1 mg H-LARYS-CO-TSAL^SCA^. The crude peptide was purified by preparative HPLC (10-60% ACN/H_2_O over 50 min) and lyophilized to afford the H-LARYS-CO-TSAL (5.9 mg, 64% yield) as a white powder.


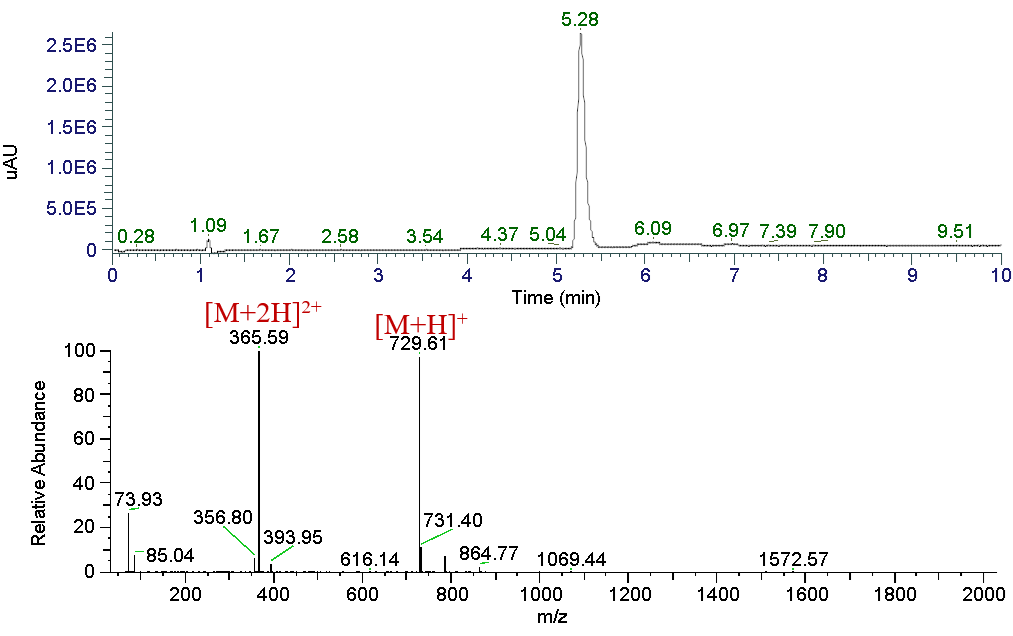


**Figure S41.** UV trace and corresponding MS from LC-MS analysis of purified H-LARYS-CO-TSAL. Gradient: 5-95% ACN/H_2_O with 0.1% TFA over 10 min at a flow rate of 0.3 mL/min. ESI-MS calcd.for C_34_H_48_N_8_O_8_S [M+H]^+^ m/z = 729.33, found 729.61; [M+2H]^2+^ m/z = 365.17, found 365.59.

6.9 H-LARYT-CO-TSAL

H-LARYT-CO-TSAL was obtained according to **Pyruvic acid treatment of peptide TSAL^SCA^-esters for C-terminus peptide TSAL-esters** on 10.0 mg H-LARYT-CO-TSAL^SCA^. The crude peptide was purified by preparative HPLC (10-60% ACN/H_2_O over 50 min) and lyophilized to afford the H-LARYT-CO-TSAL (6.6 mg, 71% yield) as a white powder.


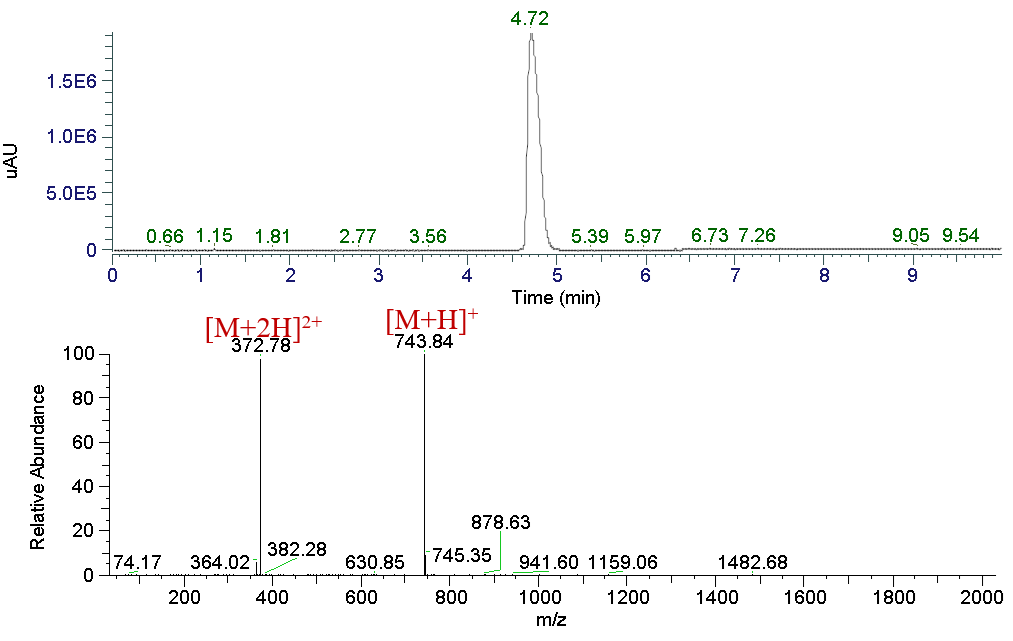


**Figure S42.** UV trace and corresponding MS from LC-MS analysis of purified H-LARYT-CO-TSAL. Gradient: 5-95% ACN/H_2_O with 0.1% TFA over 10 min at a flow rate of 0.3 mL/min. ESI-MS calcd.for C_35_H_50_N_8_O_8_S [M+H]^+^ m/z = 743.35, found 743.84; [M+2H]^2+^ m/z = 372.18, found 372.78.

6.10 H-LARYP-CO-TSAL

H-LARYP-CO-TSAL was obtained according to **Pyruvic acid treatment of peptide TSAL^SCA^-esters for C-terminus peptide TSAL-esters** on 57.1 mg H-LARYP-CO-TSAL^SCA^. The crude peptide was purified by preparative HPLC (10-60% ACN/H_2_O over 50 min) and lyophilized to afford the H-LARYP-CO-TSAL (38.5 mg, 73% yield) as a white powder.


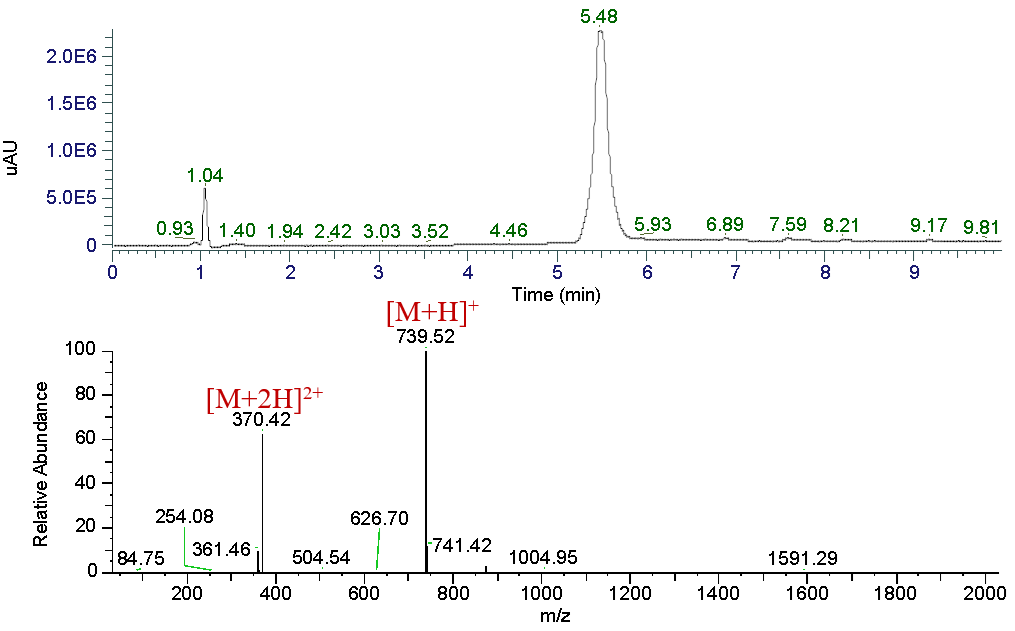


**Figure S43.** UV trace and corresponding MS from LC-MS analysis of purified H-LARYP-CO-TSAL. Gradient: 5-95% ACN/H_2_O with 0.1% TFA over 10 min at a flow rate of 0.3 mL/min. ESI-MS calcd.for C_36_H_50_N_8_O_7_S [M+H]^+^ m/z = 739.35, found 739.52; [M+2H]^2+^ m/z = 370.18, found 370.42.

6.11 H-LARYV-CO-TSAL

H-LARYV-CO-TSAL was obtained according to **Pyruvic acid treatment of peptide TSAL^SCA^-esters for C-terminus peptide TSAL-esters** on 16.7 mg H-LARYV-CO-TSAL^SCA^. The crude peptide was purified by preparative HPLC (10-60% ACN/H_2_O over 50 min) and lyophilized to afford the H-LARYV-CO-TSAL (11.0 mg, 71% yield) as a white powder.


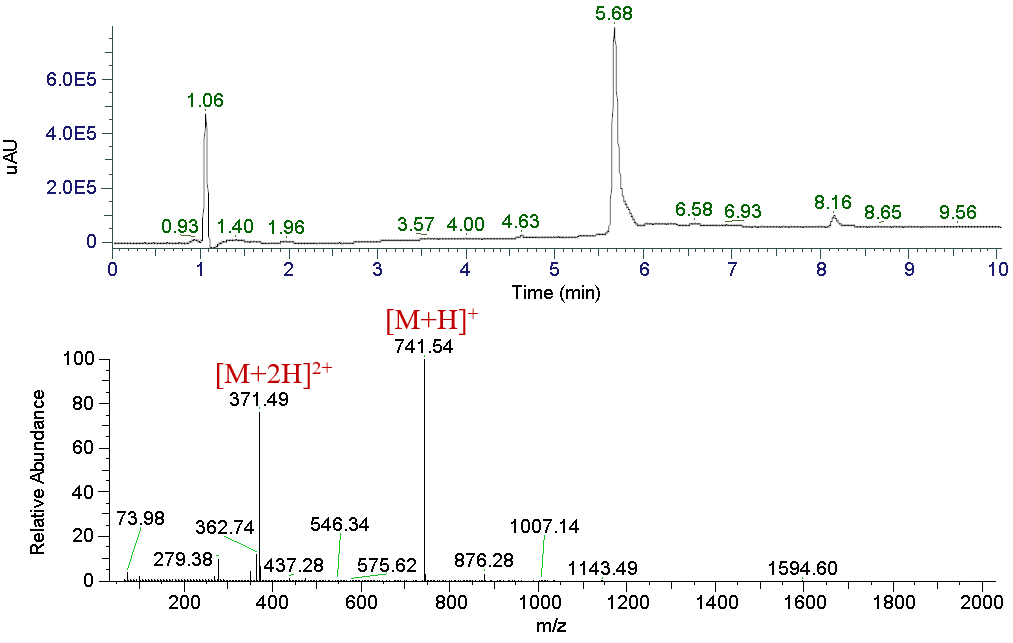


**Figure S44.** UV trace and corresponding MS from LC-MS analysis of purified H-LARYV-CO-TSAL. Gradient: 5-95% ACN/H_2_O with 0.1% TFA over 10 min at a flow rate of 0.3 mL/min. ESI-MS calcd.for C_36_H_52_N_8_O_7_S [M+H]^+^ m/z = 741.37, found 741.54; [M+2H]^2+^ m/z = 371.19, found 371.49.

6.12 H-LEAGR-CO-TSAL

H-LEAGR-CO-TSAL was obtained according to **Pyruvic acid treatment of peptide TSAL^SCA^-esters for C-terminus peptide TSAL-esters** on 16.2 mg H-LEAGR-CO-TSAL^SCA^. The crude peptide was purified by preparative HPLC (10-60% ACN/H_2_O over 50 min) and lyophilized to afford the H-LEAGR-CO-TSAL (7.5 mg, 60% yield) as a white powder.


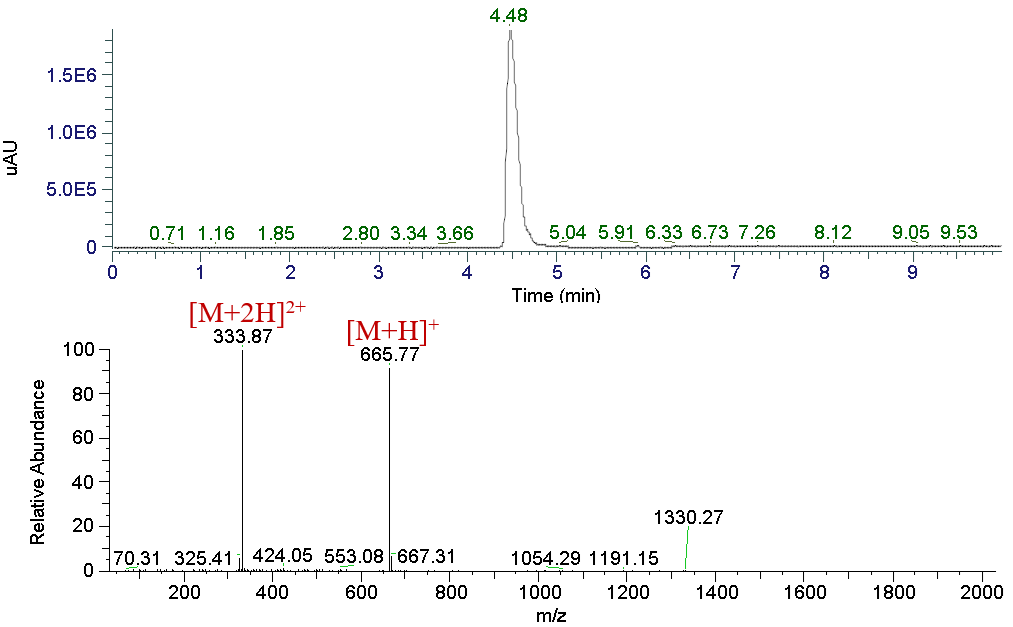


**Figure S45.** UV trace and corresponding MS from LC-MS analysis of purified H-LEAGR-CO-TSAL. Gradient: 5-95% ACN/H_2_O with 0.1% TFA over 10 min at a flow rate of 0.3 mL/min. ESI-MS calcd.for C_29_H_44_N_8_O_8_S [M+H]^+^ m/z = 665.30, found 665.77; [M+2H]^2+^ m/z = 333.15, found 333.87.

6.13 H-GDVGI-CO-TSAL

H-GDVGI-CO-TSAL was obtained according to **Pyruvic acid treatment of peptide TSAL^SCA^-esters for C-terminus peptide TSAL-esters** on 14.8 mg H-GDVGI-CO-TSAL^SCA^. The crude peptide was purified by preparative HPLC (10-60% ACN/H_2_O over 50 min) and lyophilized to afford the H-GDVGI-CO-TSAL (10.1 mg, 75% yield) as a white powder.


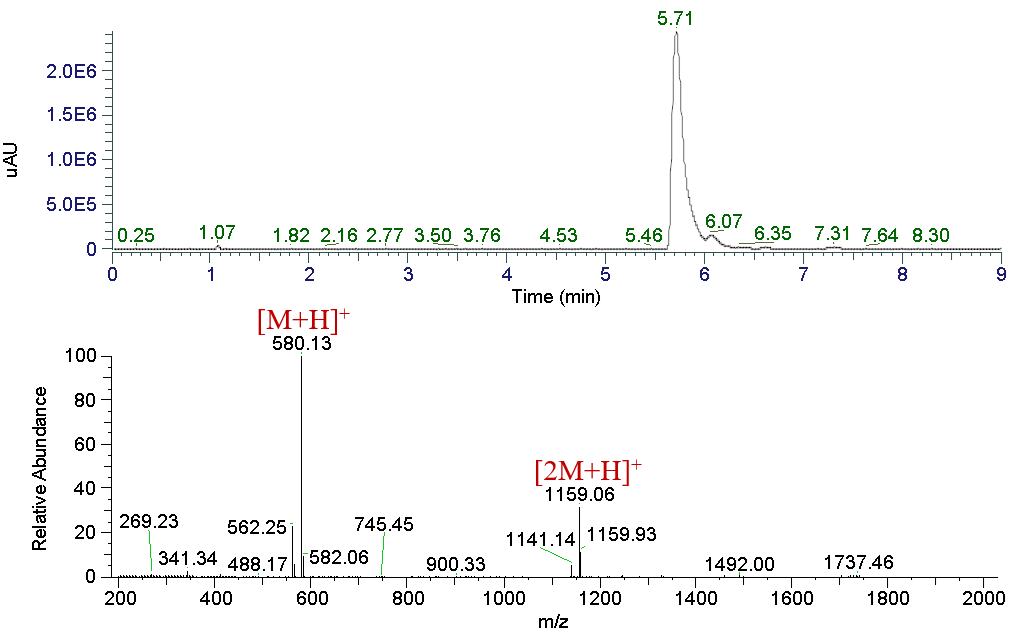


**Figure S46.** UV trace and corresponding MS from LC-MS analysis of purified H-GDVGI-CO-TSAL. Gradient: 5-95% ACN/H_2_O with 0.1% TFA over 9 min at a flow rate of 0.3 mL/min. ESI-MS calcd.for C_26_H_37_N_5_O_8_S [M+H]^+^ m/z = 580.24, found 580.13; [2M+H]^+^ m/z = 1159.48, found 1159.06.

6.14 H-GDVGL-CO-TSAL

H-GDVGL-CO-TSAL was obtained according to **Pyruvic acid treatment of peptide TSAL^SCA^-esters for C-terminus peptide TSAL-esters** on 5.2 mg H-GDVGL-CO-TSAL^SCA^. The crude peptide was purified by preparative HPLC (10-60% ACN/H_2_O over 50 min) and lyophilized to afford the H-GDVGL-CO-TSAL (3.2 mg, 68% yield) as a white powder.


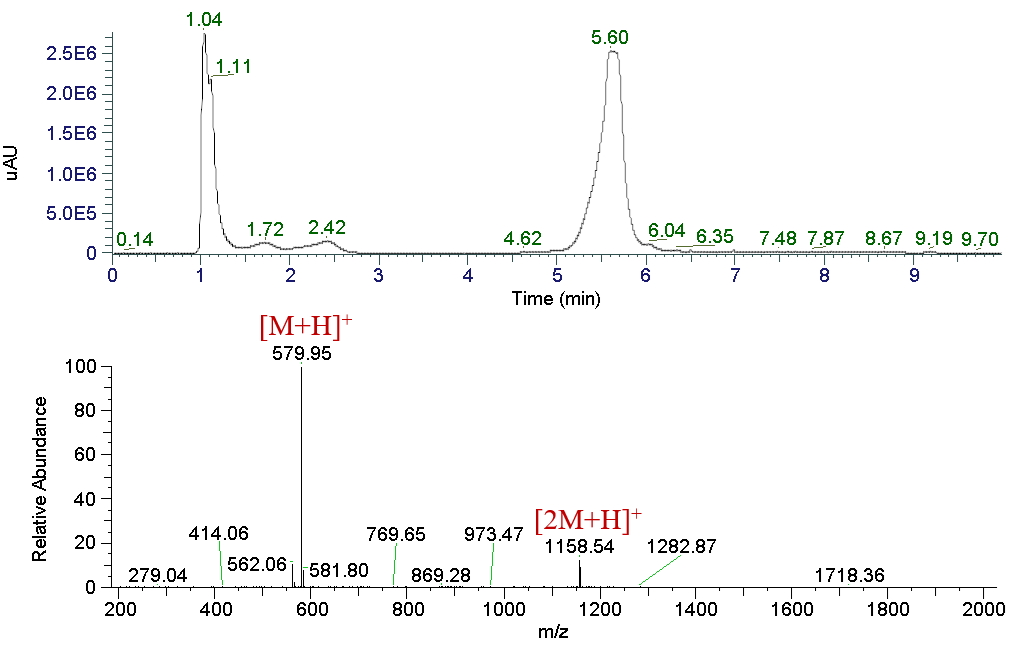


**Figure S47.** UV trace and corresponding MS from LC-MS analysis of purified H-GDVGL-CO-TSAL. Gradient: 5-95% ACN/H_2_O with 0.1% TFA over 9 min at a flow rate of 0.3 mL/min. ESI-MS calcd.for C_26_H_37_N_5_O_8_S [M+H]^+^ m/z = 580.24, found 579.95; [2M+H]^+^ m/z = 1159.48, found 1158.54.

6.15 H-LEAGY-CO-TSAL

H-LEAGY-CO-TSAL was obtained according to **Pyruvic acid treatment of peptide TSAL^SCA^-esters for C-terminus peptide TSAL-esters** on 20.0 mg H-LEAGY-CO-TSAL^SCA^. The crude peptide was purified by preparative HPLC (10-60% ACN/H_2_O over 50 min) and lyophilized to afford the H-LEAGY-CO-TSAL (14.7 mg, 80% yield) as a white powder.


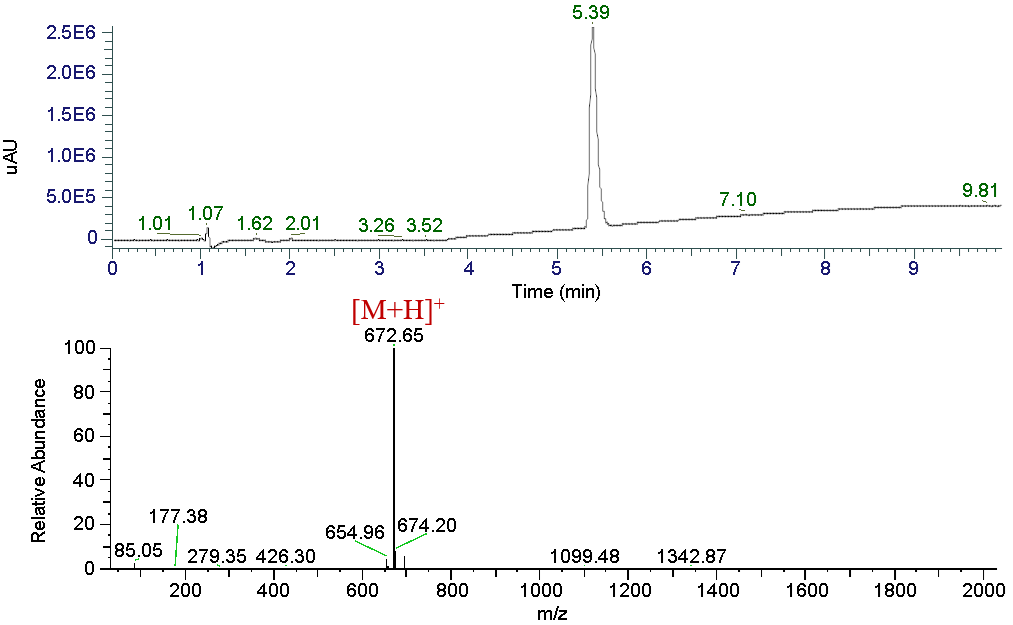


**Figure S48.** UV trace and corresponding MS from LC-MS analysis of purified H-LEAGY-CO-TSAL. Gradient: 5-95% ACN/H_2_O with 0.1% TFA over 10 min at a flow rate of 0.3 mL/min. ESI-MS calcd.for C_32_H_41_N_5_O_9_S [M+H]^+^ m/z = 672.26, found 672.65.

6.16 Ac-SYSMEHFRWGKP-CO-TSAL

Ac-SYSMEHFRWGKP-CO-TSAL was obtained according to **Direct coupling for C-terminus Gly and Pro peptide TSAL esters** on 16.7 mg fully protected crude peptide Ac-SYSMEHFRWGKP-COOH. The crude peptide was purified by preparative HPLC (20-60% ACN/H_2_O over 40 min) and lyophilized to afford the Ac-SYSMEHFRWGKP-CO-TSAL (5.0 mg, 49% yield) as a white powder.


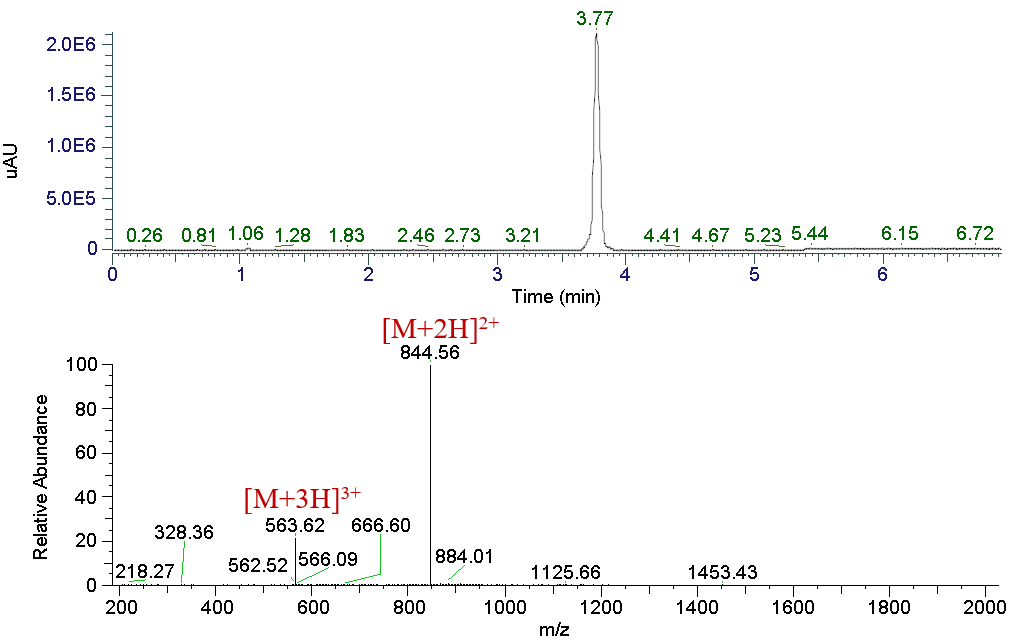


**Figure S49.** UV trace and corresponding MS from LC-MS analysis of purified Ac-SYSMEHFRWGKP-CO-TSAL. Gradient: 5-95% ACN/H_2_O with 0.1% TFA over 7 min at a flow rate of 0.3 mL/min. ESI-MS calcd.for C_79_H_103_N_19_O_19_S_2_ [M+2H]^2+^ m/z = 843.86, found 844.56; [M+3H]^3+^ m/z = 562.90, found 563.62.

6.17 H-AGVEGL-CO-TSAL

H-AGVEGL-CO-TSAL was obtained according to **“N+1” strategy for C-terminus Ala, Val, and Leu peptide TSAL esters** on 23.7 mg fully protected crude peptide H-AGVEG-COOH. The crude peptide was purified by preparative HPLC (10-60% ACN/H_2_O over 50 min) and lyophilized to afford the H-AGVEGL-CO-TSAL (2.6 mg, 10% yield) as a white powder.

_
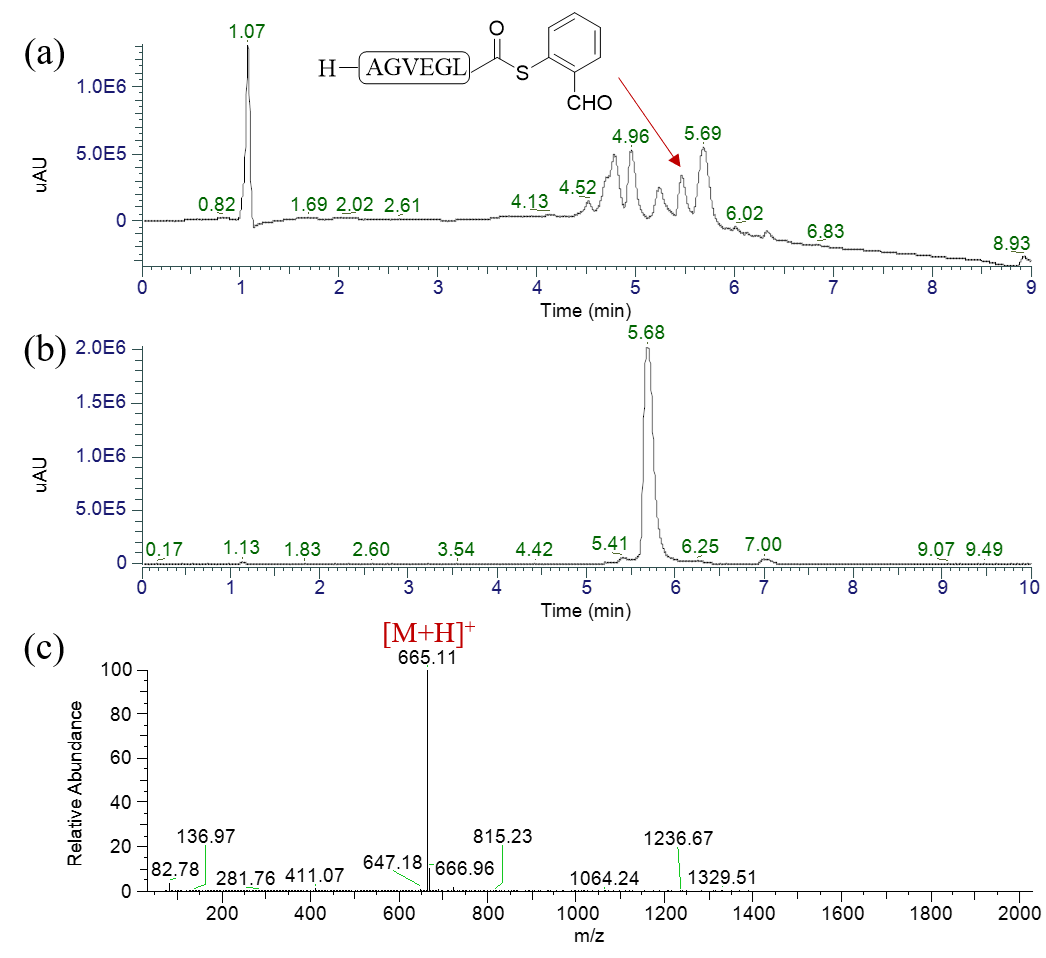
_

**Figure S50.** (a) Crude HPLC trace for “N+1” strategy to generate H-AGVEGL-CO-TSAL. Gradient: 10-80% ACN/H_2_O with 0.1% TFA over 9 min at a flow rate of 0.3 mL/min; (b) UV trace of purified H-AGVEGL-CO-TSAL. Gradient: 5-95% ACN/H_2_O with 0.1% TFA over 10 min at a flow rate of 0.3 mL/min; (c) Corresponding MS of purified H-AGVEGL-CO-TSAL. ESI-MS calcd.for C_30_H_44_N_6_O_9_S [M+H]^+^ m/z = 665.29, found 665.11.

6.18 H-ATVAGA-CO-TSAL

H-AGVEGA-CO-TSAL was obtained according to **“N+1” strategy for C-terminus Ala, Val, and Leu peptide TSAL esters** on 35.2 mg fully protected crude peptide H-AGVEG-COOH. The crude peptide was purified by preparative HPLC (10-60% ACN/H_2_O over 50 min) and lyophilized to afford the H-AGVEGA-CO-TSAL (4.5 mg, 12% yield) as a white powder.

_
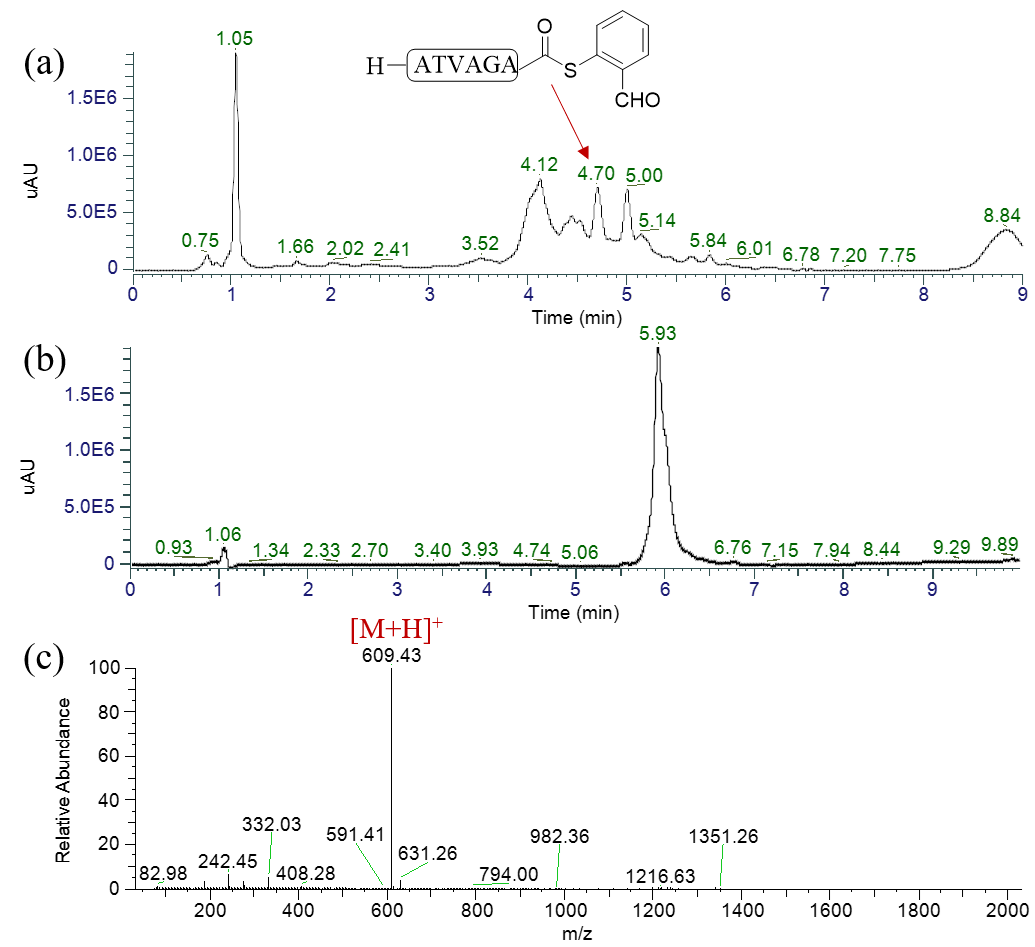
_

**Figure S51.** (a) Crude HPLC trace for “N+1” strategy to generate H-AGVEGA-CO-TSAL. Gradient: 10-80% ACN/H_2_O with 0.1% TFA over 9 min at a flow rate of 0.3 mL/min; (b) UV trace of purified H-AGVEGA-CO-TSAL. Gradient: 5-95% ACN/H_2_O with 0.1% TFA over 10 min at a flow rate of 0.3 mL/min; (c) Corresponding MS of purified H-AGVEGA-CO-TSAL. ESI-MS calcd.for C_27_H_40_N_6_O_8_S [M+H]^+^ m/z = 609.26, found 609.43.

6.19 H-LSKQGL-CO-TSAL

H-LSKQGL-CO-TSAL was obtained according to **“N+1” strategy for C-terminus Ala, Val, and Leu peptide TSAL esters** on 10.0 mg fully protected crude peptide H-LSKQG-COOH. The crude peptide was purified by preparative HPLC (10-60% ACN/H_2_O over 50 min) and lyophilized to afford the H-LSKQGL-CO-TSAL (0.7 mg, 10% yield) as a white powder.

_
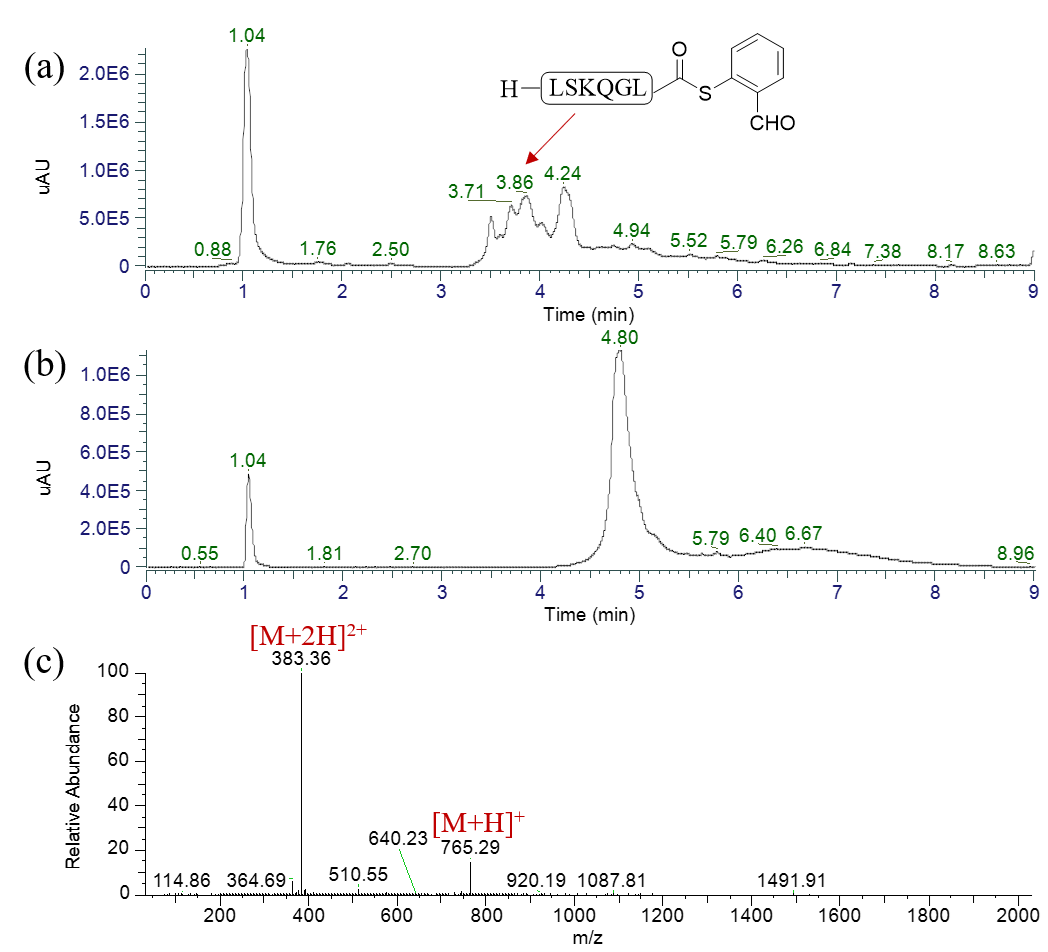
_

**Figure S52.** (a) Crude HPLC trace for “N+1” strategy to generate H-LSKQGL-CO-TSAL. Gradient: 10-80% ACN/H_2_O with 0.1% TFA over 9 min at a flow rate of 0.3 mL/min; (b) UV trace of purified H-LSKQGL-CO-TSAL. Gradient: 5-95% ACN/H_2_O with 0.1% TFA over 10 min at a flow rate of 0.3 mL/min; (c) Corresponding MS of purified H-LSKQGL-CO-TSAL. ESI-MS calcd.for C_35_H_56_N_8_O_9_S [M+H]^+^ m/z = 765.39, found 765.29; [M+2H]^2+^ m/z = 383.20, found 383.36.

6.20 H-LSKQGV-CO-TSAL

H-LSKQGV-CO-TSAL was obtained according to **“N+1” strategy for C-terminus Ala, Val, and Leu peptide TSAL esters** on 10.0 mg fully protected crude peptide H-LSKQG-COOH. The crude peptide was purified by preparative HPLC (10-60% ACN/H_2_O over 50 min) and lyophilized to afford the H-LSKQGV-CO-TSAL (0.9 mg, 13% yield) as a white powder.

_
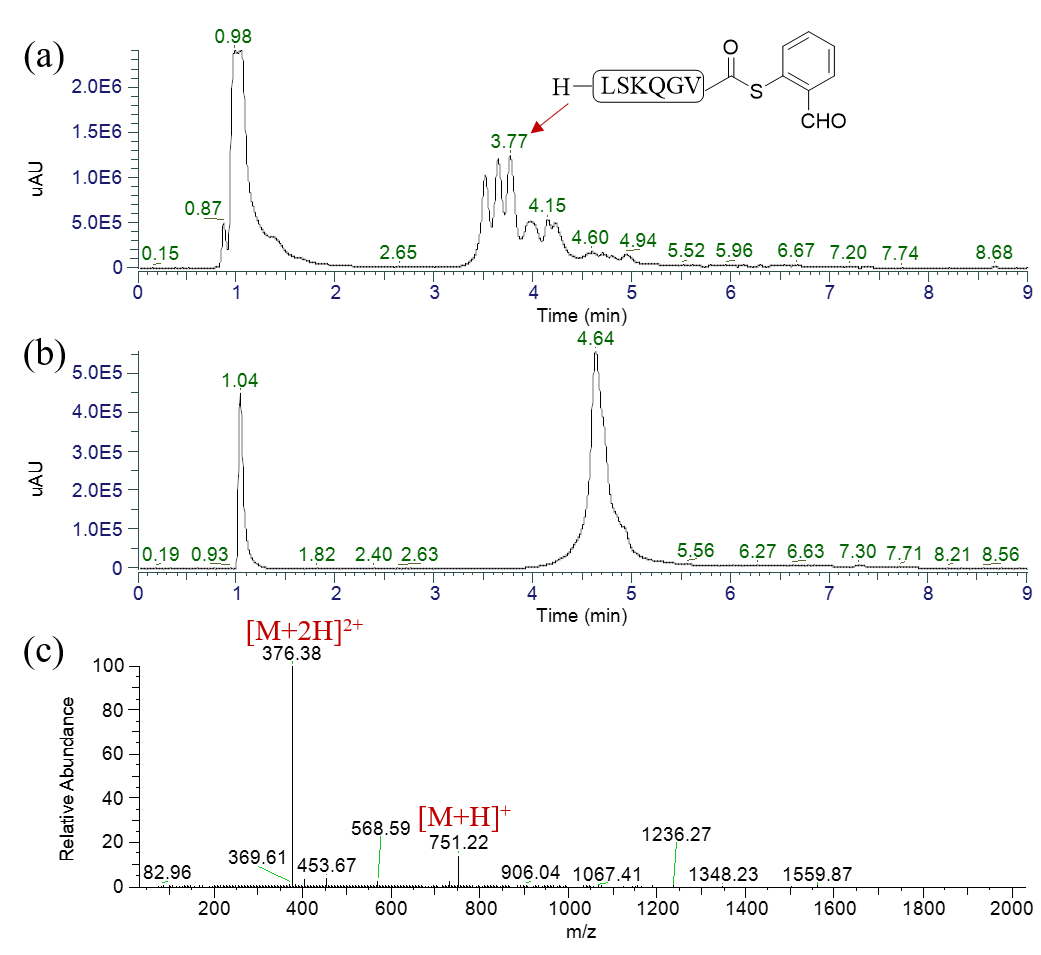
_

**Figure S53.** (a) Crude HPLC trace for “N+1” strategy to generate H-LSKQGV-CO-TSAL. Gradient: 10-80% ACN/H_2_O with 0.1% TFA over 9 min at a flow rate of 0.3 mL/min. (b) UV trace of purified H-LSKQGV-CO-TSAL. Gradient: 5-95% ACN/H_2_O with 0.1% TFA over 10 min at a flow rate of 0.3 mL/min. (c) Corresponding MS of purified H-LSKQGV-CO-TSAL. ESI-MS calcd.for C_34_H_54_N_8_O_9_S [M+H]^+^ m/z = 751.37, found 751.22; [M+2H]^2+^ m/z = 376.19, found 376.38.

**7. Epimerization study of Cys ligation**

An epimerization study of TCPL at C-terminal was performed using H-LARYP-CO-TSAL ester and H-LARYp-CO-TSAL ester reacting with H-CSALF-OH, respectively (see below). These ligated products were analyzed by LC-MS. The epimerization study was also performed using H-LARY**S**-CO-TSAL ester reacting with H-CSALF-OH and H-LARY**s**CSALF-OH synthesized by standard SPPS, respectively. The results were analyzed by analytical HPLC (Agilent 1260). No epimerization at C-terminal during ligations were observed.

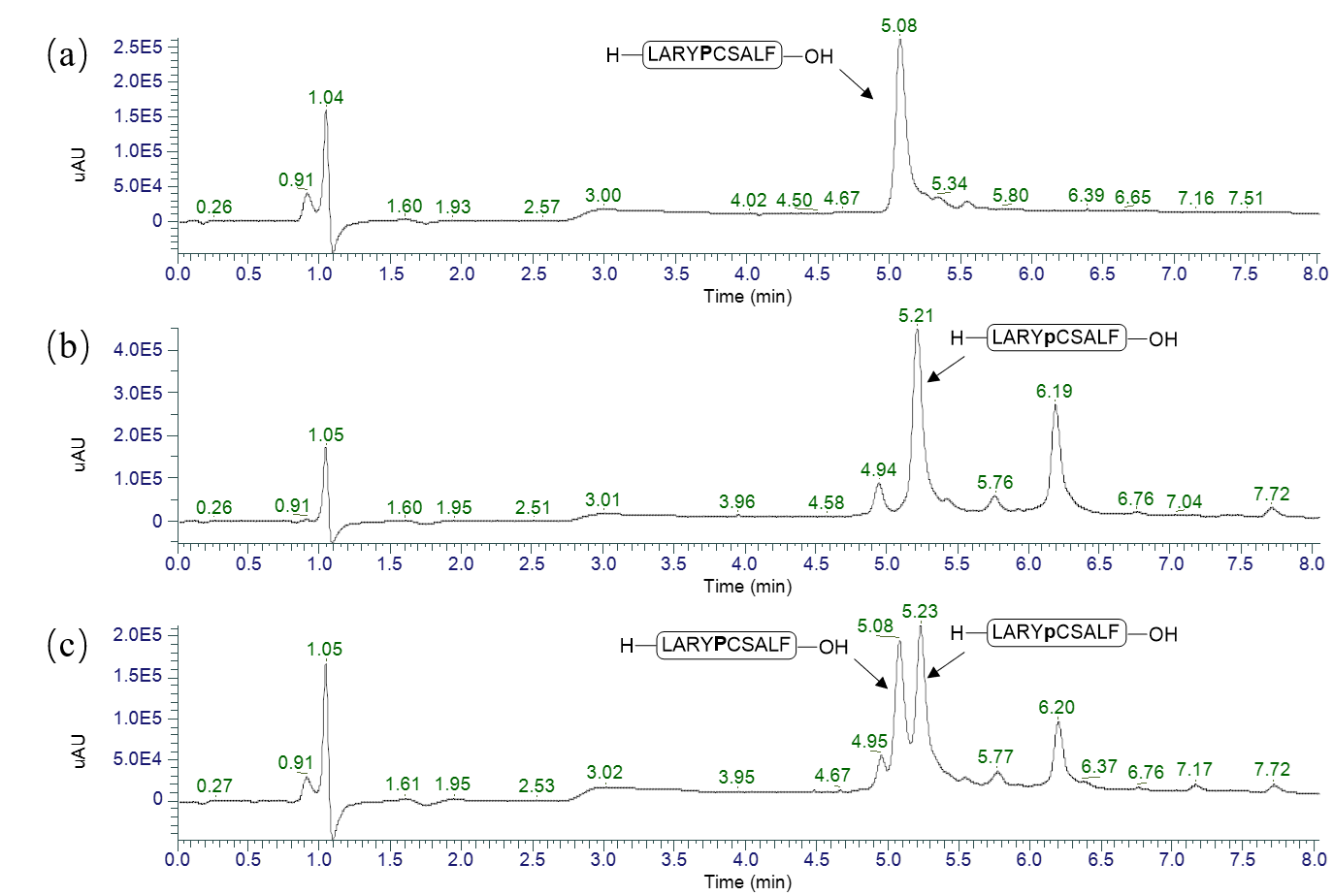


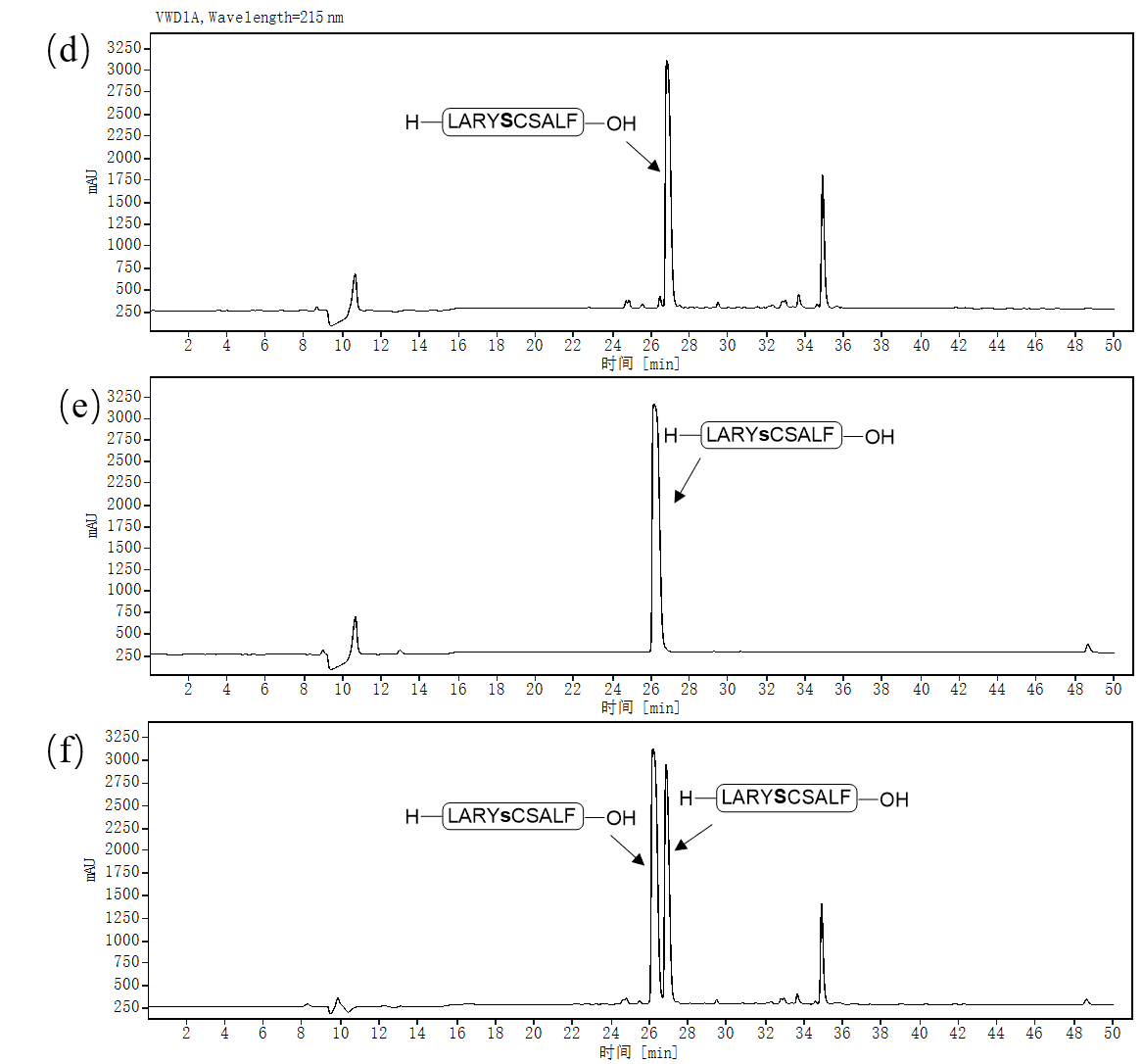


Figure S54. HPLC traces of the ligation and acidolysis reaction revealing that no epimerization at C-terminal occurred. (a) Ligation and acidolysis product of H-LARY**P**CSALF-OH; (b) Crude ligation and acidolysis product of H-LARY**p**CSALF-OH; (c) Co-injection of two ligation and acidolysis products (H-LARY**P**CSALF-OH and H-LARY**p**CSALF-OH). (d) Crude ligation and acidolysis product of H-LARY**S**CSALF-OH; (e) Purified standard SPPS product of H-LARY**s**CSALF-OH; (f) Co-injection of H-LARY**S**CSALF-OH and H-LARY**s**CSALF-OH.

**8. Ligation reaction condition screening for TCPL**

**
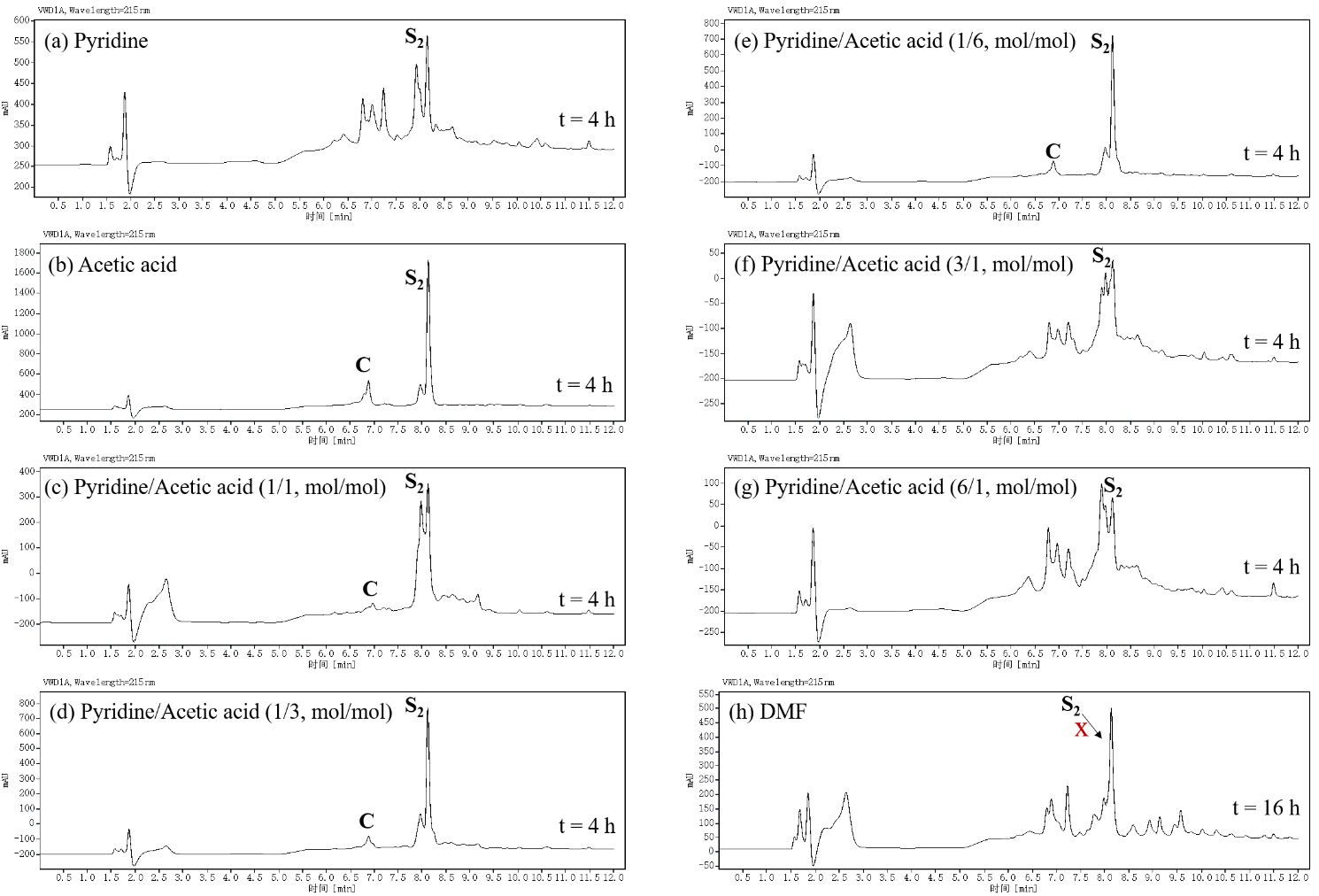
**

Figure S55. Crude HPLC traces of different ligation reaction conditions of H-LARYS-CO-TSAL and H-CSALF-OH. Gradient: 20-90 % ACN/H_2_O with 0.1% TFA over 12 min at a flow rate of 0.3 mL/min.

**
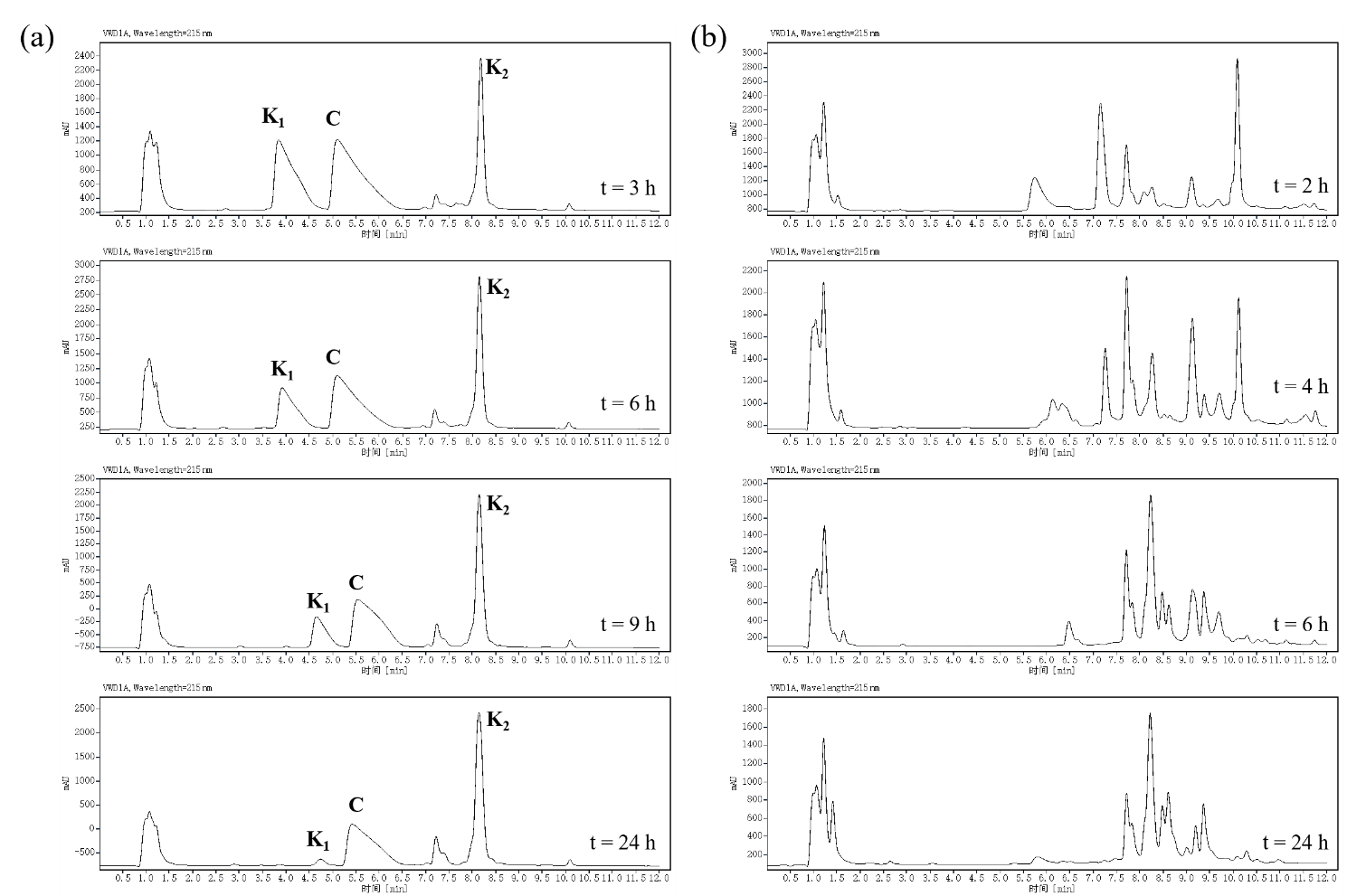
**

Figure S56. Crude HPLC traces of different ligation reaction conditions of H-LARYK-CO-TSAL and H-CSALF-OH. Gradient: 20-70 % ACN/H_2_O with 0.1% TFA over 12 min at a flow rate of 0.3 mL/min. (a) 0.2 M Phosphate solution containing 6 M Gn∙HCl, pH 3.0, 25°C, 2 mM; (b) 0.2 M Phosphate solution containing 6 M Gn∙HCl, pH 7.0, 25°C, 2 mM.

**9. Acidolysis condition screening for TCPL**

**Table S1.** Acidolysis condition screening for TCPL at Gly site.

| Entry | Reaction Conditions | *t*[h] | T [℃] | Conversion [%]^[a]^ |
| --- | --- | --- | --- | --- |
| 1 | TFA/TMSOTf/EDT = 90/5/5, (V/V/V) | 4 | 25 | 27% |
| 2 | TFA/TMSOTf/EDT = 90/5/5, (V/V/V) | 4 | 4 | 71% |
| 3 | TFA/TMSOTf/EDT = 90/5/5, (V/V/V) | 4 | 0 | 86% |
| 4 | TFA/TMSOTf/EDT = 90/5/5, (V/V/V) | 4 | -15 | 86% |
| 5 | TFA/TMSOTf/EDT = 90/5/5, (V/V/V) | 2 | 0 | 85% |
| 6 | TFA/TMSOTf/EDT = 95/2.5/2.5, (V/V/V) | 4 | -15 | 77% |
| 7 | TFA/TMSOTf/EDT = 80/10/10, (V/V/V) | 4 | -15 | 83% |
| 8 | TFA/TBSOTf/EDT = 90/5/5, (V/V/V) | 4 | 0 | 80% |
| 9 | TFA/TfOH/EDT = 90/5/5, (V/V/V) | 4 | 0 | 10% |
| 10 | TFA/TMSOTf/H_2_O = 90/5/5, (V/V/V) | 4 | 0 | 35% |
| 11 | TFA/TMSOTf/DODT = 90/5/5, (V/V/V) | 5 | 0 | Messy |
| 12 | HCl/EDT/TFE = 90/5/5, (V/V/V) | 4 | 0 | Messy |
| 13 | TFA/TMSOTf/EDT/TIPS/Phenol = 80/5/5/5/5, (V/V/V/V/V) | 4 | -15 | 65% |

[a]Analysis by HPLC traces of the crude reaction mixture.

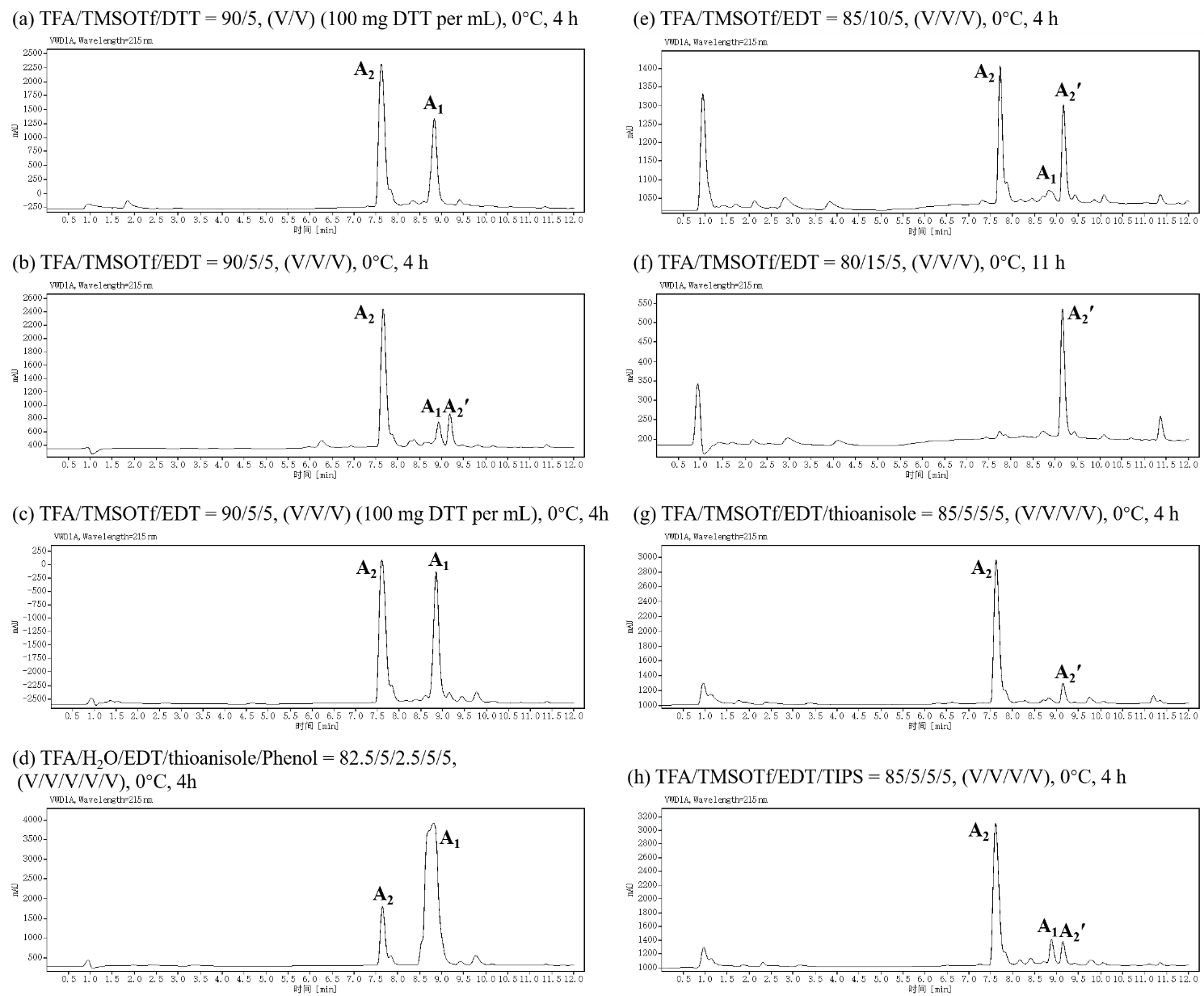


Figure S57.Crude HPLC traces of different acidolysis conditions for TCPL at Ala site. Gradient: 20-70 % ACN/H_2_O with 0.1% TFA over 12 min at a flow rate of 0.3 mL/min. A_2_′: Cys-EDT adduct and Arg or Tyr sulfonation of A_2_.

**
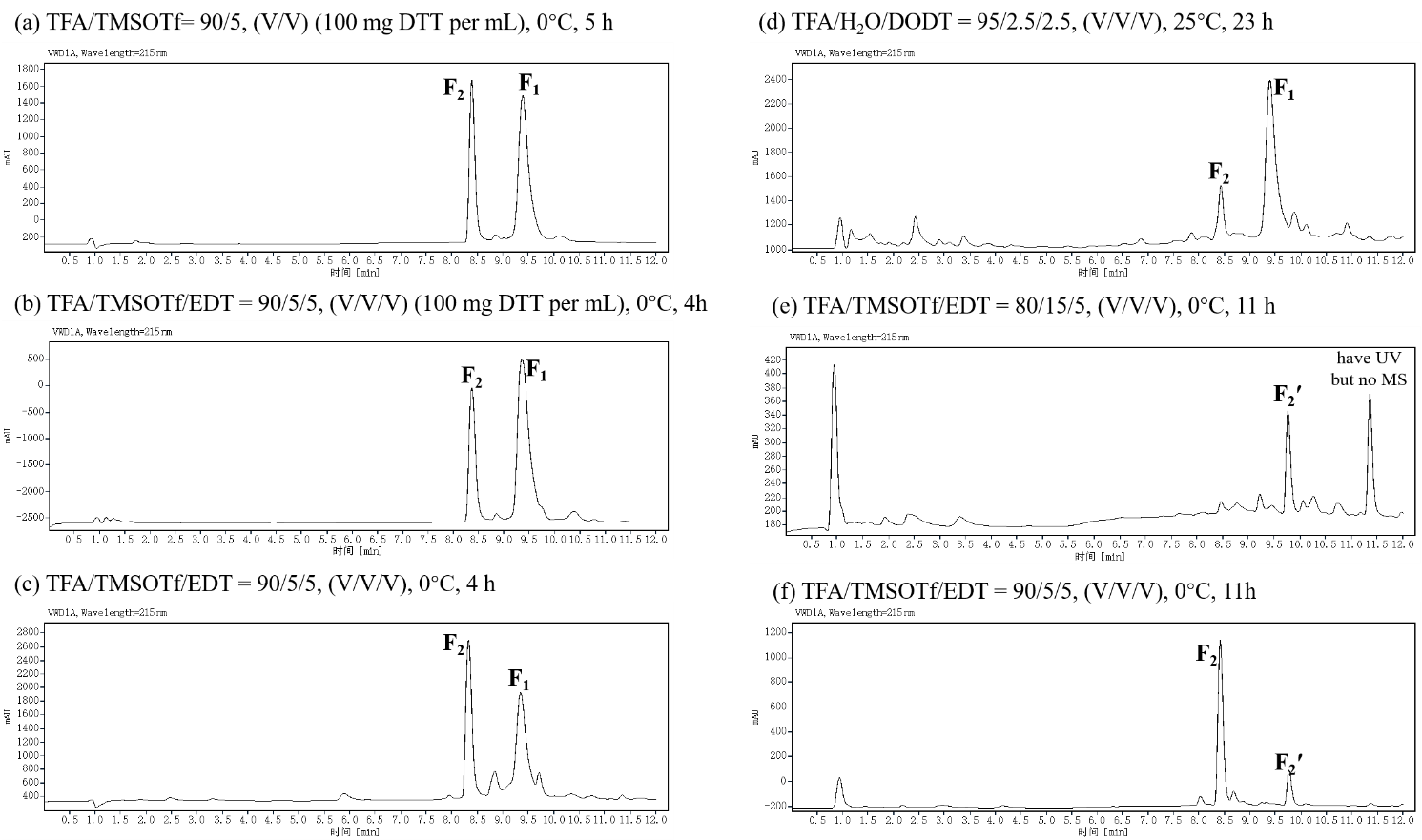
**

Figure S58.Crude HPLC traces of different acidolysis conditions for TCPL at Phe site. Gradient: 20-70 % ACN/H_2_O with 0.1% TFA over 12 min at a flow rate of 0.3 mL/min. F_2_′: Cys-EDT adduct and Arg or Tyr sulfonation of F_2_.

**10. TSAL ester-Cys ligation of model peptides**

10.1 H-LARYA-CO-TSAL + H-CSALF-OH

The ligation between H-LARYA-CO-TSAL (3.8 mg, 5.3 μmol) and H-CSALF-OH (3.5 mg, 6.5 μmol) was performed as described in the **General procedure for TSAL ester-Cys/Penicillamine ligation**. Purification via preparative HPLC (20-70% ACN/H_2_O over 40 min, 0.1% TFA) followed by lyophilization afforded the N,S-benzylidene acetal intermediate (4.4 mg, 67% yield) as white solids. And the intermediate (2.0 mg) was then treated with TFA/EDT/TMSOTf (90/5/5, v/v/v) for 4 h and carried out as the **General procedure for Acidolysis**. Purification via preparative HPLC (20-70% ACN/H_2_O over 40 min, 0.1%TFA) followed by lyophilization afforded the H-LARYACSALF-OH (1.2 mg, 87% yield) as white solids.


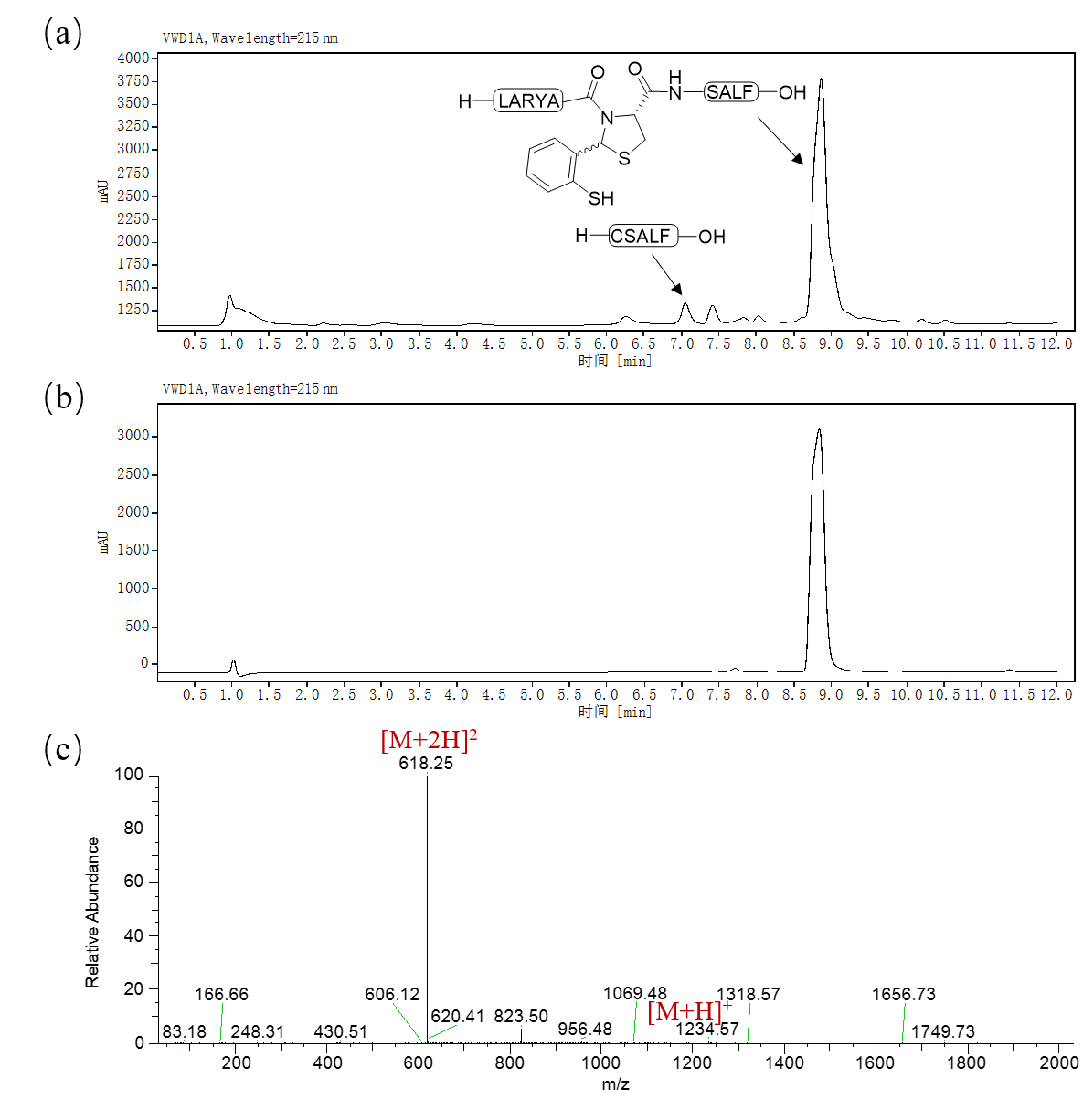


**Figure S59.** (a) Crude HPLC trace for the TSAL ester-Cys ligation between H-LARYA-CO-TSAL and H-CSALF-OH. Gradient: 20-70% ACN/H_2_O with 0.1% TFA over 12 min at a flow rate of 0.3 mL/min. (b) UV trace of the purified N,S-benzylidene acetal intermediate. Gradient: 20-70% ACN/H_2_O with 0.1% TFA over 12 min at a flow rate of 0.3 mL/min. (c) Corresponding MS of the purified N,S-benzylidene acetal intermediate. ESI-MS calcd.for C_58_H_83_N_13_O_13_S_2_ [M+H]^+^ m/z = 1234.57, found 1234.57; [M+2H]^2+^ m/z = 617.79, found 618.25.


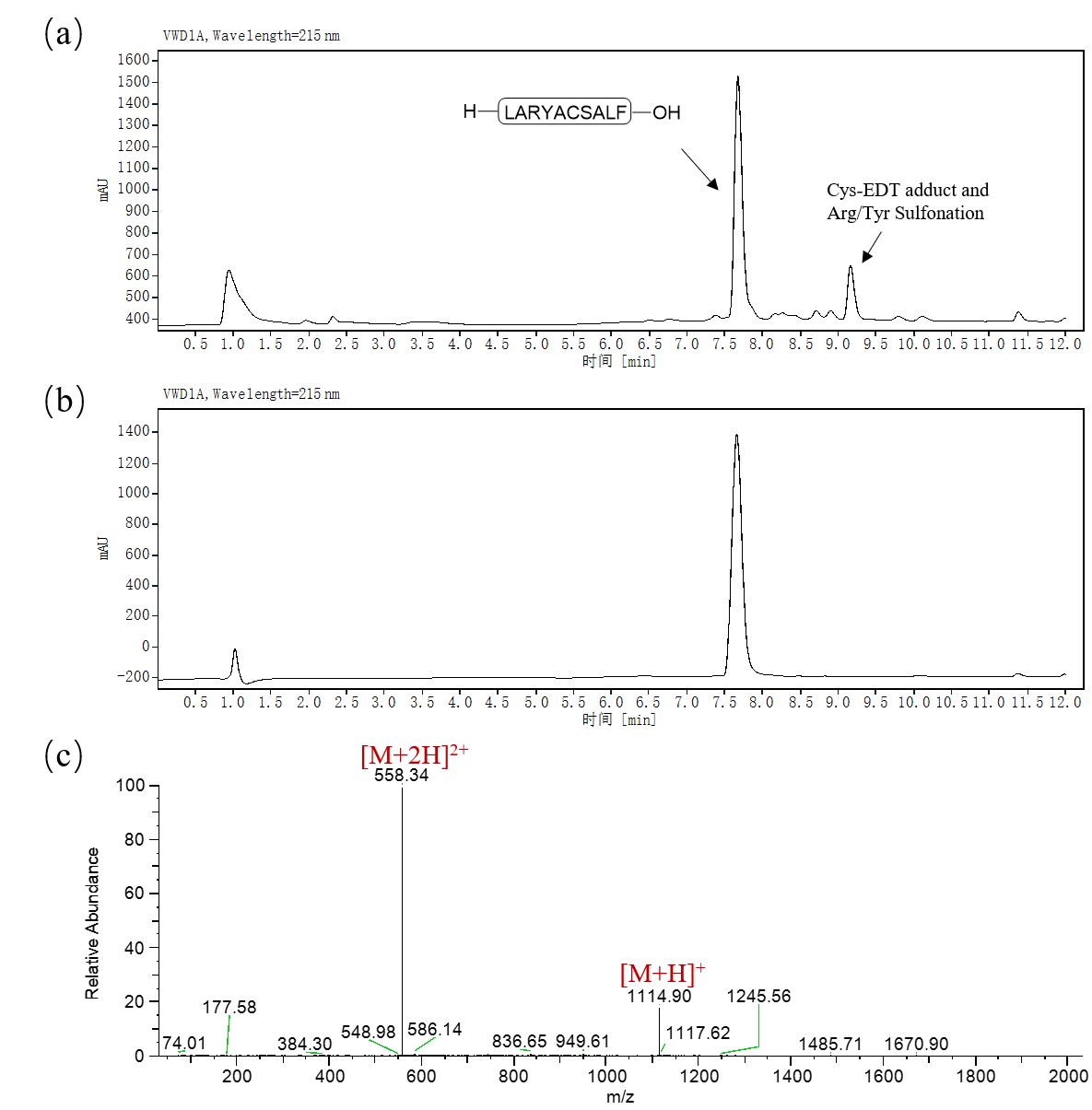


**Figure S60.** (a) Crude HPLC trace for acidolysis of the N,S-benzylidene acetal intermediate to generate H-LARYACSALF-OH. Gradient: 20-70% ACN/H_2_O with 0.1% TFA over 12 min at a flow rate of 0.3 mL/min. (b) UV trace of purified H-LARYACSALF-OH. Gradient: 20-70% ACN/H_2_O with 0.1% TFA over 12 min at a flow rate of 0.3 mL/min. (c) Corresponding MS of purified H-LARYACSALF-OH. ESI-MS calcd.for C_51_H_79_N_13_O_13_S [M+H]^+^ m/z = 1114.56, found 1114.90; [M+2H]^2+^ m/z = 557.78, found 558.34.

10.2 H-LARYE-CO-TSAL + H-CSALF-OH

The ligation between H-LARYE-CO-TSAL (2.5 mg, 3.3 μmol) and H-CSALF-OH (2.1 mg, 3.9 μmol) was performed as described in the **General procedure for TSAL ester-Cys/Penicillamine ligation**. Purification via preparative HPLC (20-70% ACN/H_2_O over 40 min, 0.1% TFA) followed by lyophilization afforded the N,S-benzylidene acetal intermediate (2.3 mg, 55% yield) as white solids. And the intermediate (0.9 mg) was then treated with TFA/EDT/TMSOTf (90/5/5, v/v/v) for 4 h and carried out as the **General procedure for Acidolysis**. Purification via preparative HPLC (20-70% ACN/H_2_O over 40 min, 0.1%TFA) followed by lyophilization afforded the H-LARYECSALF-OH (0.7 mg, 85% yield) as white solids.


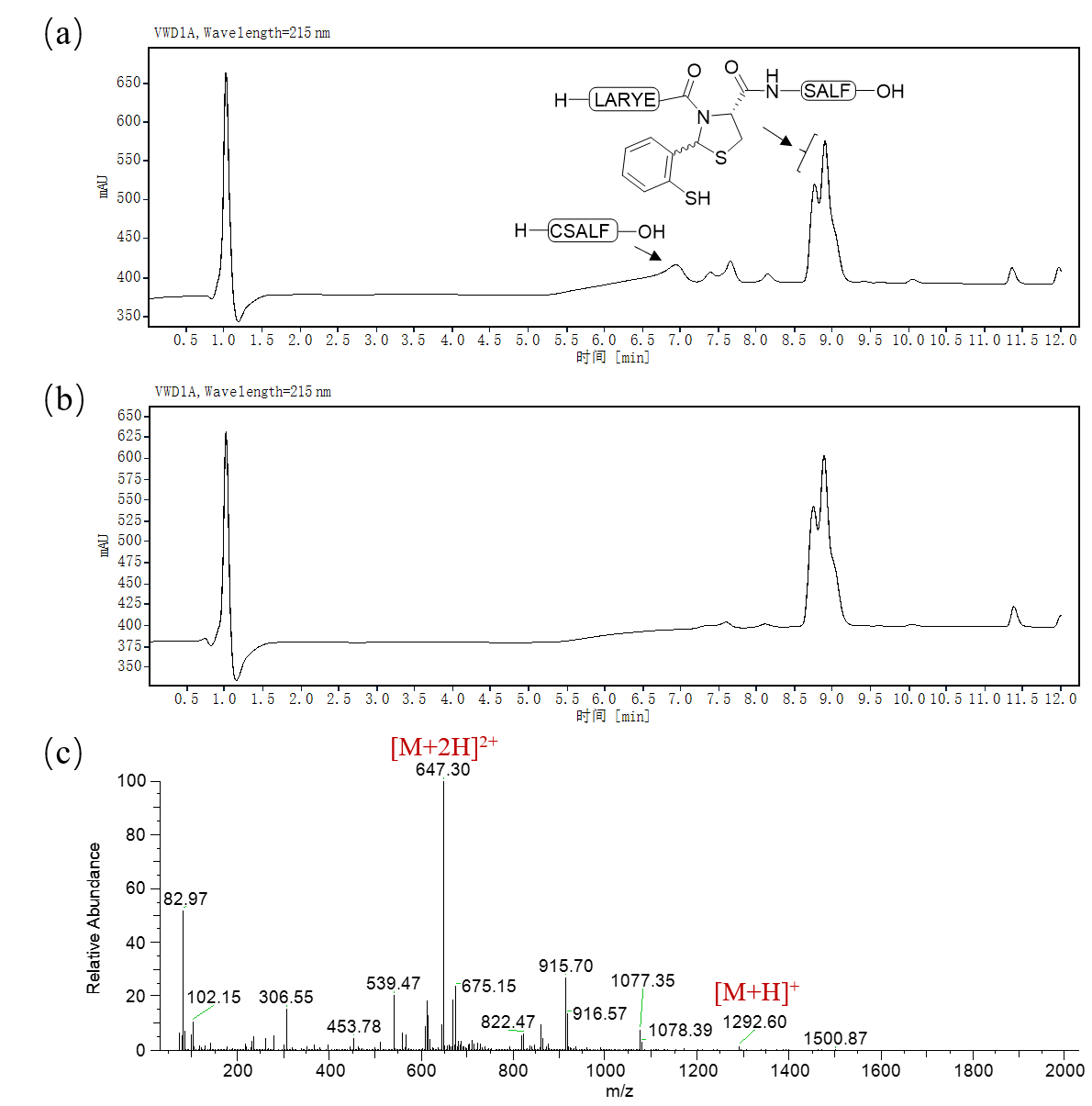


**Figure S61.** (a) Crude HPLC trace for the TSAL ester-Cys ligation between H-LARYE-CO-TSAL and H-CSALF-OH. Gradient: 20-70% ACN/H_2_O with 0.1% TFA over 12 min at a flow rate of 0.3 mL/min. (b) UV trace of the purified N,S-benzylidene acetal intermediate. Gradient: 20-70% ACN/H_2_O with 0.1% TFA over 12 min at a flow rate of 0.3 mL/min. (c) Corresponding MS of the purified N,S-benzylidene acetal intermediate. ESI-MS calcd.for C_60_H_85_N_13_O_15_S_2_ [M+H]^+^ m/z = 1292.57, found 1292.60; [M+2H]^2+^ m/z = 646.79, found 647.30.


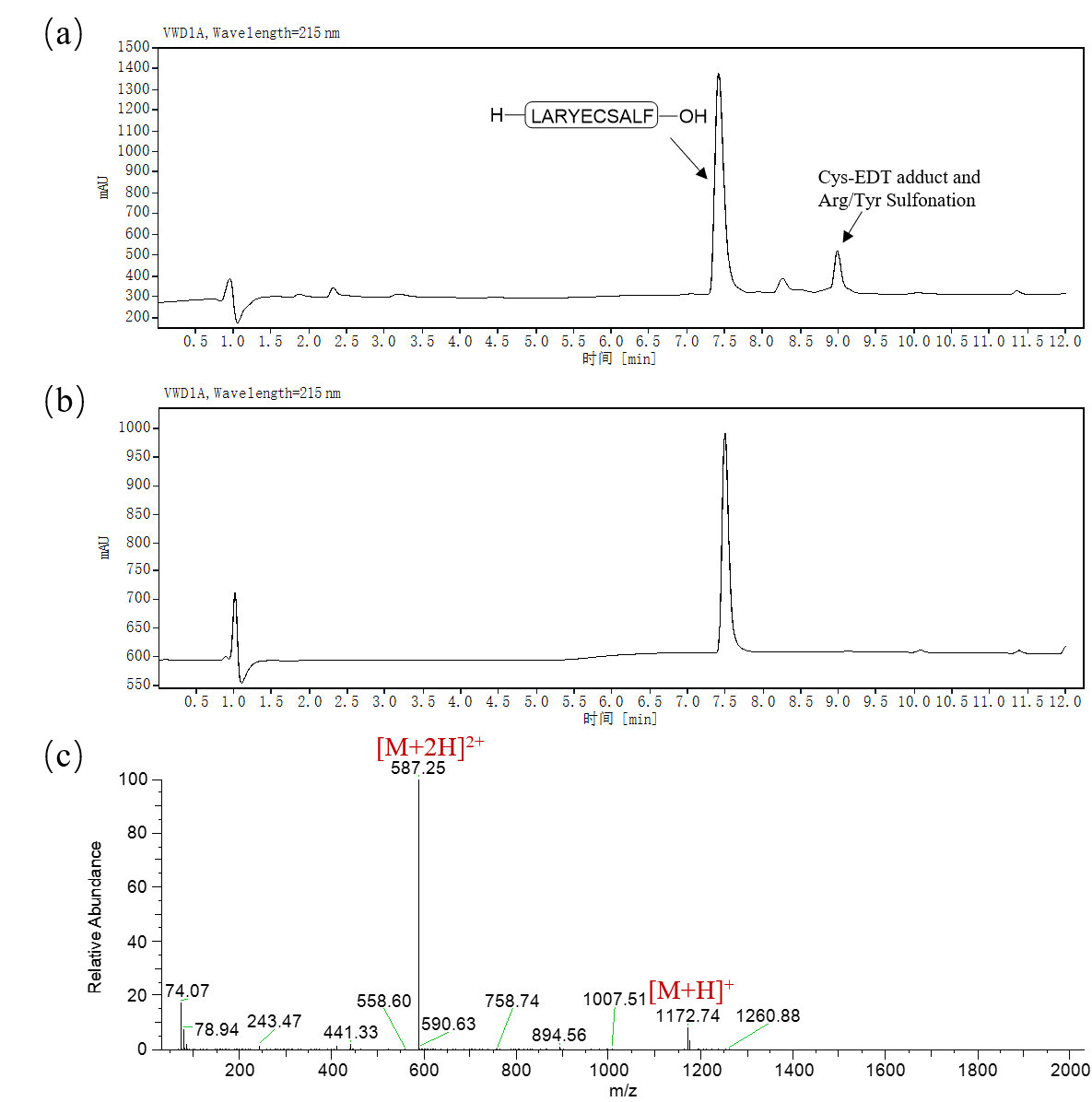


**Figure S62.** (a) Crude HPLC trace for acidolysis of the N,S-benzylidene acetal intermediate to generate H-LARYECSALF-OH. Gradient: 20-70% ACN/H_2_O with 0.1% TFA over 12 min at a flow rate of 0.3 mL/min. (b) UV trace of purified H-LARYECSALF-OH. Gradient: 20-70% ACN/H_2_O with 0.1% TFA over 12 min at a flow rate of 0.3 mL/min. (c) Corresponding MS of purified H-LARYECSALF-OH. ESI-MS calcd.for C_53_H_81_N_13_O_15_S [M+H]^+^ m/z = 1172.57, found 1172.74; [M+2H]^2+^ m/z = 586.79, found 587.25.

10.3 H-LARYF-CO-TSAL + H-CSALF-OH

The ligation between H-LARYF-CO-TSAL (2.9 mg, 3.7 μmol) and H-CSALF-OH (3.0 mg, 5.6 μmol) was performed as described in the **General procedure for TSAL ester-Cys/Penicillamine ligation**. Purification via preparative HPLC (20-70% ACN/H_2_O over 40 min, 0.1% TFA) followed by lyophilization afforded the N,S-benzylidene acetal intermediate (3.0 mg, 63% isolated yield) as white solids. And the intermediate (1.3 mg) was then treated with TFA/EDT/TMSOTf (90/5/5, v/v/v) for 11 h and carried out as the **General procedure for Acidolysis**. Purification via preparative HPLC (20-70% ACN/H_2_O over 40 min, 0.1%TFA) followed by lyophilization afforded the H-LARYFCSALF-OH (0.7 mg, 76% yield) as white solids.


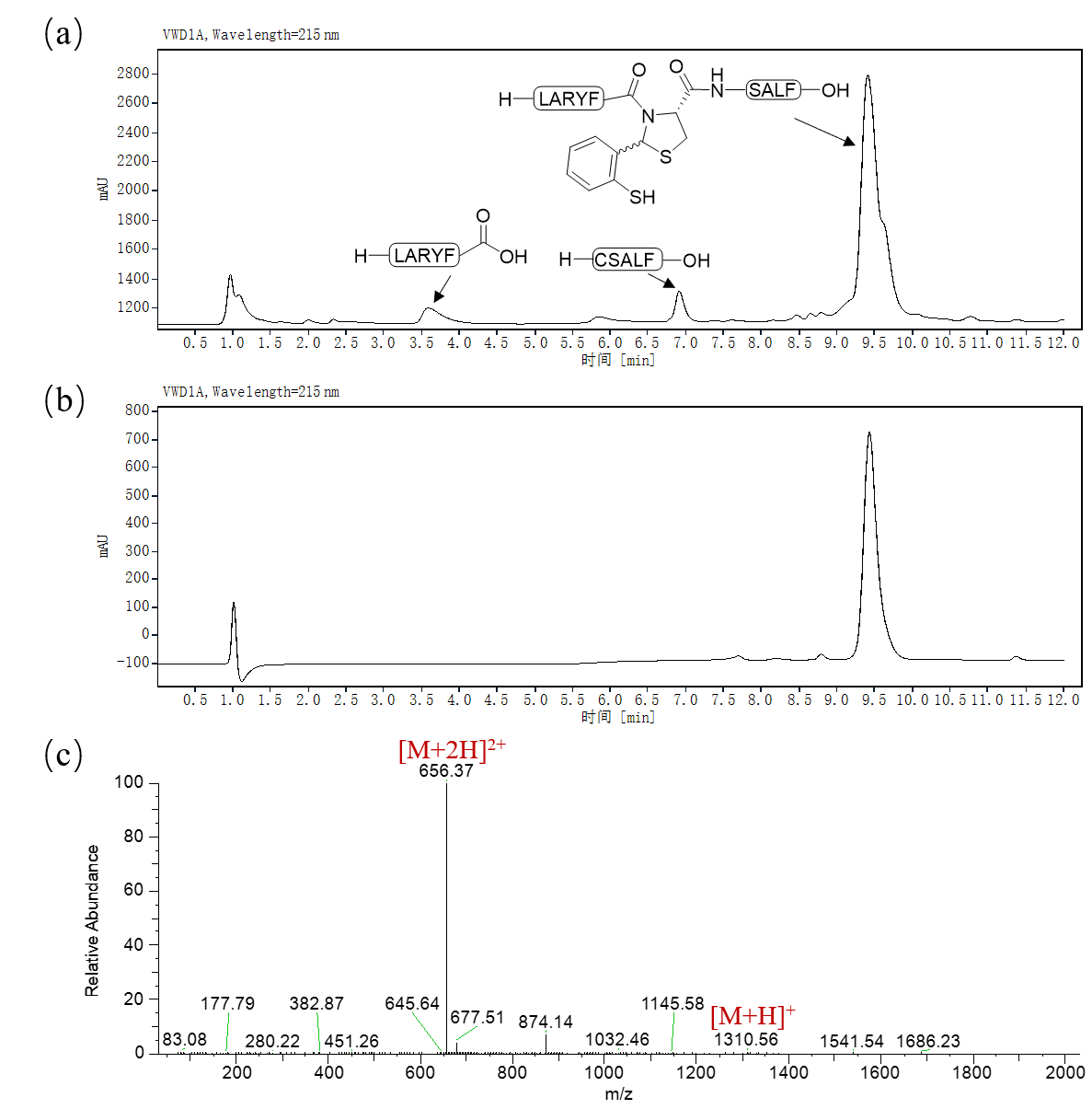


**Figure S63.** (a) Crude HPLC trace for the TSAL ester-Cys ligation between H-LARYF-CO-TSAL and H-CSALF-OH. Gradient: 20-70% ACN/H_2_O with 0.1% TFA over 12 min at a flow rate of 0.3 mL/min. (b) UV trace of the purified N,S-benzylidene acetal intermediate. Gradient: 20-70% ACN/H_2_O with 0.1% TFA over 12 min at a flow rate of 0.3 mL/min. (c) Corresponding MS of the purified N,S-benzylidene acetal intermediate. ESI-MS calcd.for C_64_H_87_N_13_O_13_S_2_ [M+H]^+^ m/z = 1310.60, found 1310.56; [M+2H]^2+^ m/z = 655.80, found 656.37.


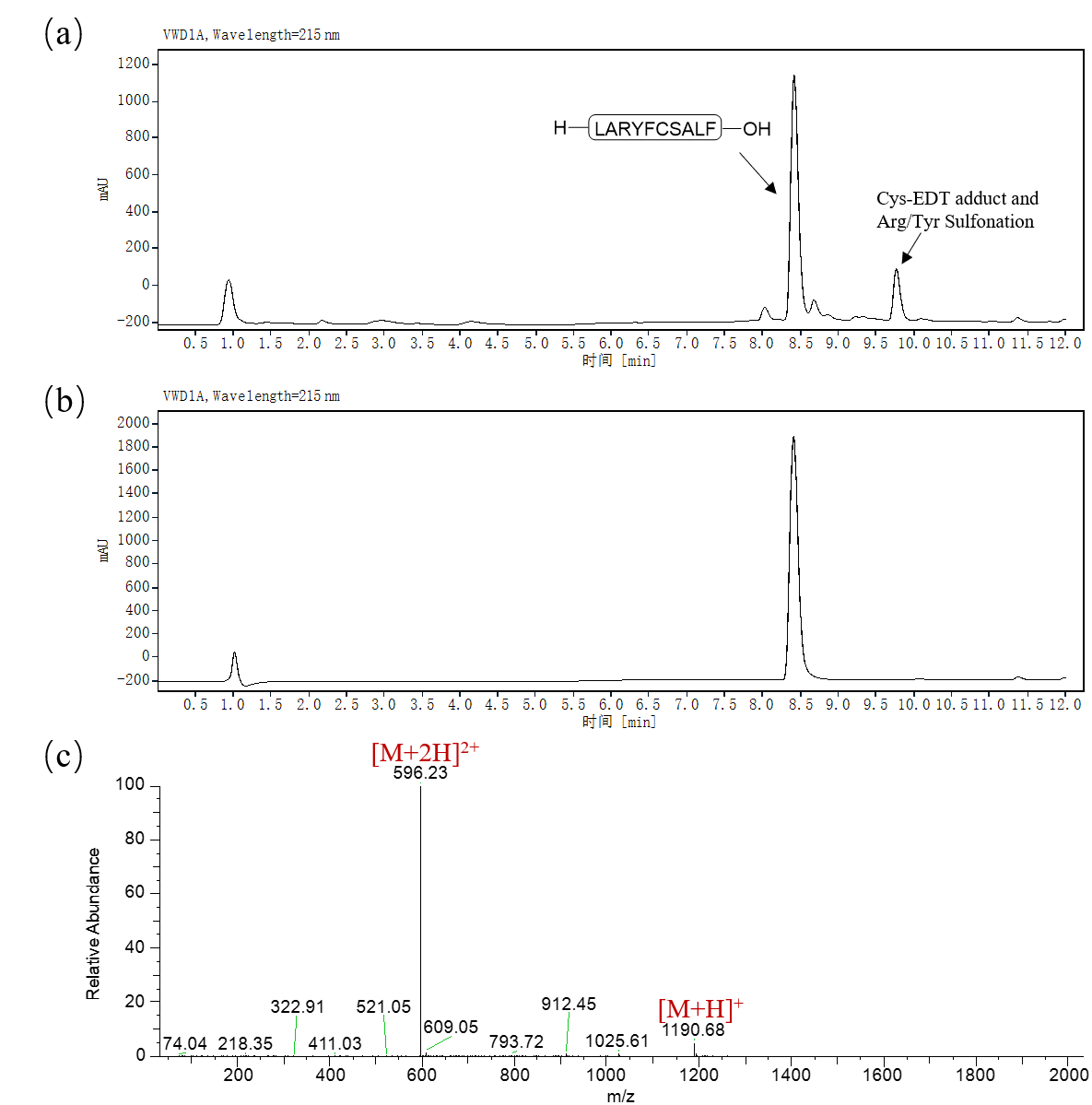


**Figure S64.** (a) Crude HPLC trace for acidolysis of the N,S-benzylidene acetal intermediate to generate H-LARYFCSALF-OH. Gradient: 20-70% ACN/H_2_O with 0.1% TFA over 12 min at a flow rate of 0.3 mL/min. (b) UV trace of purified H-LARYFCSALF-OH. Gradient: 20-70% ACN/H_2_O with 0.1% TFA over 12 min at a flow rate of 0.3 mL/min. (c) Corresponding MS of purified H-LARYFCSALF-OH. ESI-MS calcd.for C_57_H_83_N_13_O_13_S [M+H]^+^ m/z = 1190.60, found 1190.68; [M+2H]^2+^ m/z = 595.80, found 596.23.

10.4 H-LARYG-CO-TSAL + H-CSALF-OH

The ligation between H-LARYG-CO-TSAL (3.5 mg, 5.0 μmol) and H-CSALF-OH (3.1 mg, 5.8 μmol) was performed as described in the **General procedure for TSAL ester-Cys/Penicillamine ligation**. Purification via preparative HPLC (20-70% ACN/H_2_O over 40 min, 0.1% TFA) followed by lyophilization afforded the N,S-benzylidene acetal intermediate (3.3 mg, 54% yield) as white solids. And the intermediate (1.0 mg) was then treated with TFA/EDT/TMSOTf (90/5/5, v/v/v) for 4 h and carried out as the **General procedure for Acidolysis**. Purification via preparative HPLC (20-70% ACN/H_2_O over 40 min, 0.1%TFA) followed by lyophilization afforded the H-LARYGCSALF-OH (0.8 mg, 89% yield) as white solids.


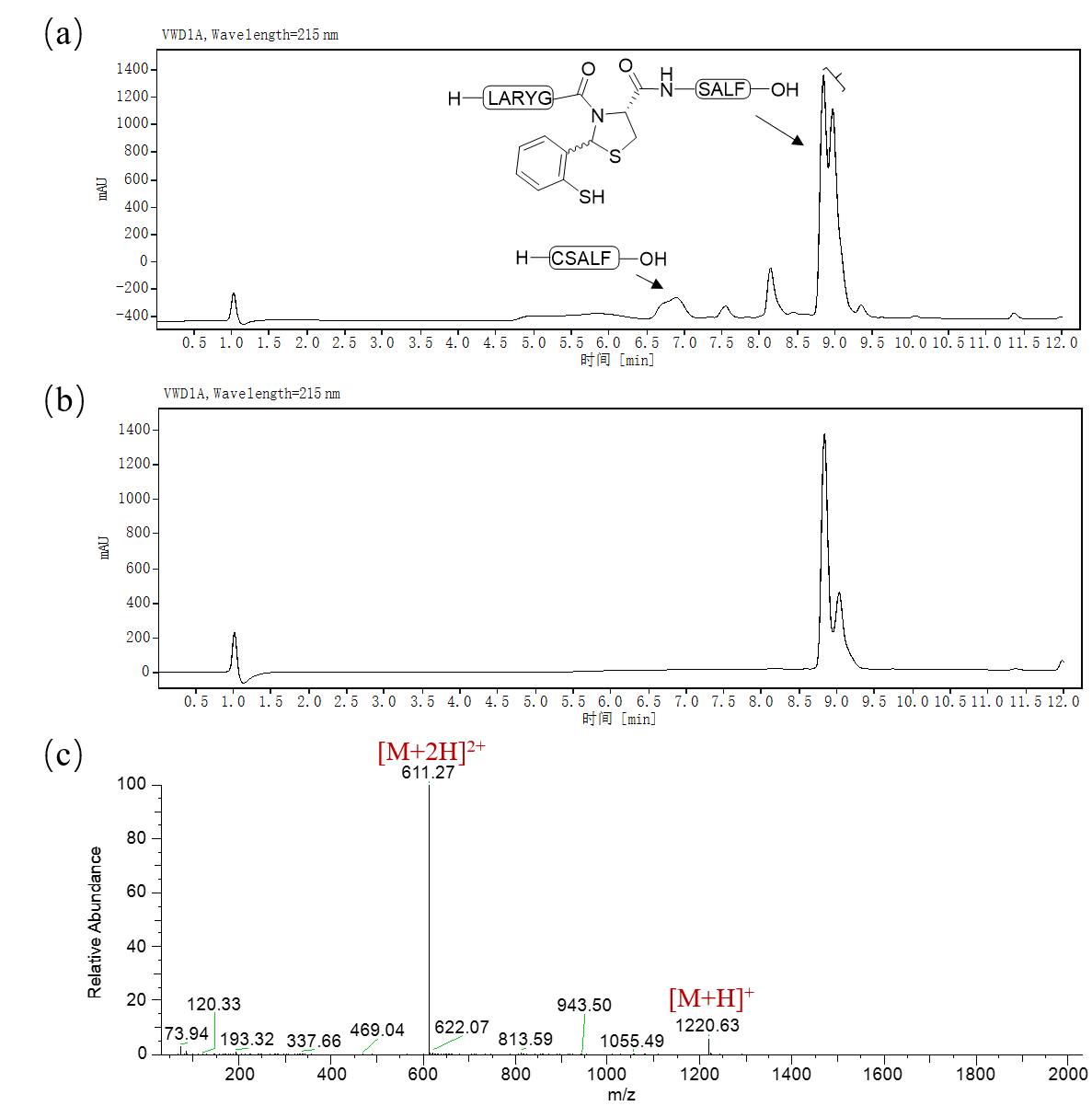


**Figure S65.** (a) Crude HPLC trace for the TSAL ester-Cys ligation between H-LARYG-CO-TSAL and H-CSALF-OH. Gradient: 20-70% ACN/H_2_O with 0.1% TFA over 12 min at a flow rate of 0.3 mL/min. (b) UV trace of the purified N,S-benzylidene acetal intermediate. Gradient: 20-70% ACN/H_2_O with 0.1% TFA over 12 min at a flow rate of 0.3 mL/min. (c) Corresponding MS of the purified N,S-benzylidene acetal intermediate. ESI-MS calcd.for C_57_H_81_N_13_O_13_S_2_ [M+H]^+^ m/z = 1220.55, found 1220.63; [M+2H]^2+^ m/z = 610.78, found 611.27.


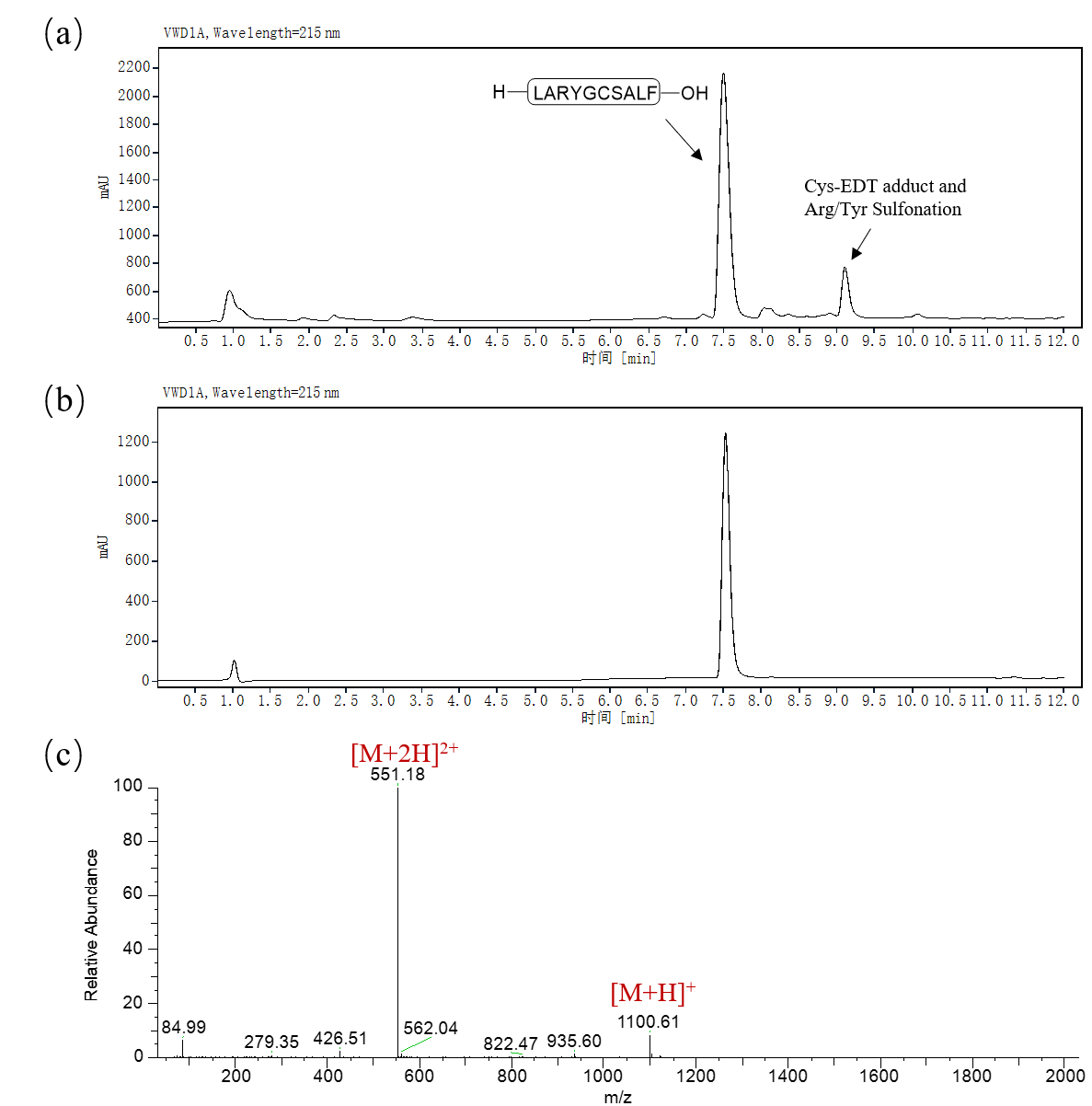


**Figure S66.** (a) Crude HPLC trace for acidolysis of the N,S-benzylidene acetal intermediate to generate H-LARYGCSALF-OH. Gradient: 20-70% ACN/H_2_O with 0.1% TFA over 12 min at a flow rate of 0.3 mL/min. (b) UV trace of purified H-LARYGCSALF-OH. Gradient: 20-70% ACN/H_2_O with 0.1% TFA over 12 min at a flow rate of 0.3 mL/min. (c) Corresponding MS of purified H-LARYGCSALF-OH. ESI-MS calcd.for C_50_H_77_N_13_O_13_S [M+H]^+^ m/z = 1100.55, found 1100.61; [M+2H]^2+^ m/z = 550.78, found 551.18.

10.5 H-LARYH-CO-TSAL+ H-CSALF-OH

The ligation between H-LARYH-CO-TSAL (2.1 mg, 2.7 μmol) and H-CSALF-OH (1.7 mg, 3.2 μmol) was performed as described in the **General procedure for TSAL ester-Cys/Penicillamine ligation**. Purification via preparative HPLC (20-70% ACN/H_2_O over 40 min, 0.1% TFA) followed by lyophilization afforded the N,S-benzylidene acetal intermediate (2.1 mg, 62% yield) as white solids. And the intermediate (4.0 mg) was then treated with TFA/EDT/TMSOTf (90/5/5, v/v/v) for 24 h and carried out as the **General procedure for Acidolysis**. Purification via preparative HPLC (20-70% ACN/H_2_O over 40 min, 0.1%TFA) followed by lyophilization afforded the H-LARYHCSALF-OH (1.9 mg, 53% yield) as white solids.


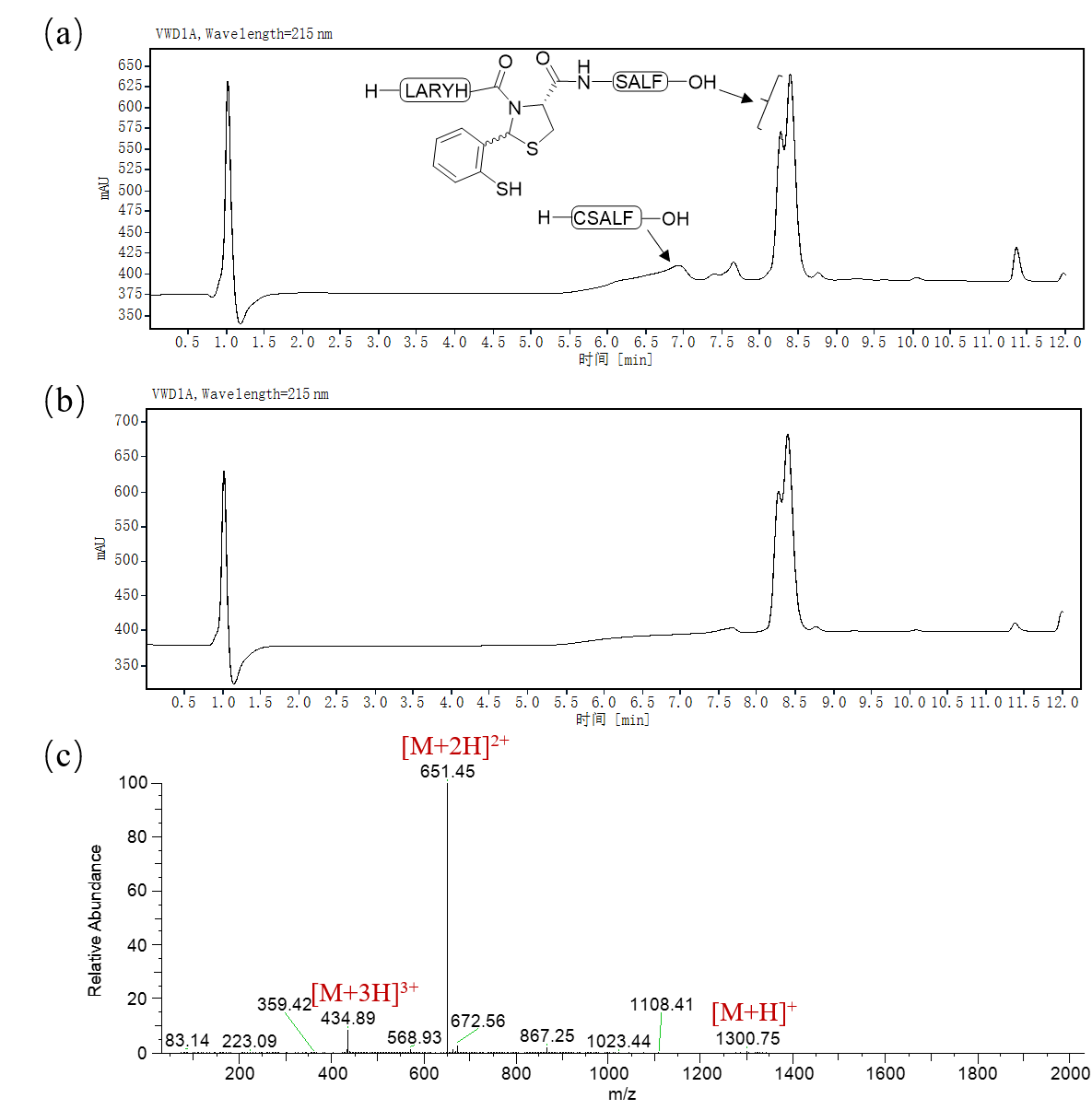


**Figure S67.** (a) Crude HPLC trace for the TSAL ester-Cys ligation between H-LARYH-CO-TSAL and H-CSALF-OH. Gradient: 20-70% ACN/H_2_O with 0.1% TFA over 12 min at a flow rate of 0.3 mL/min. (b) UV trace of the purified N,S-benzylidene acetal intermediate. Gradient: 20-70% ACN/H_2_O with 0.1% TFA over 12 min at a flow rate of 0.3 mL/min. (c) Corresponding MS of the purified N,S-benzylidene acetal intermediate. ESI-MS calcd.for C_61_H_85_N_15_O_13_S_2_ [M+H]^+^ m/z = 1300.59, found 1300.75; [M+2H]^2+^ m/z = 650.80, found 651.45; [M+3H]^3+^ m/z = 434.20, found 434.89.


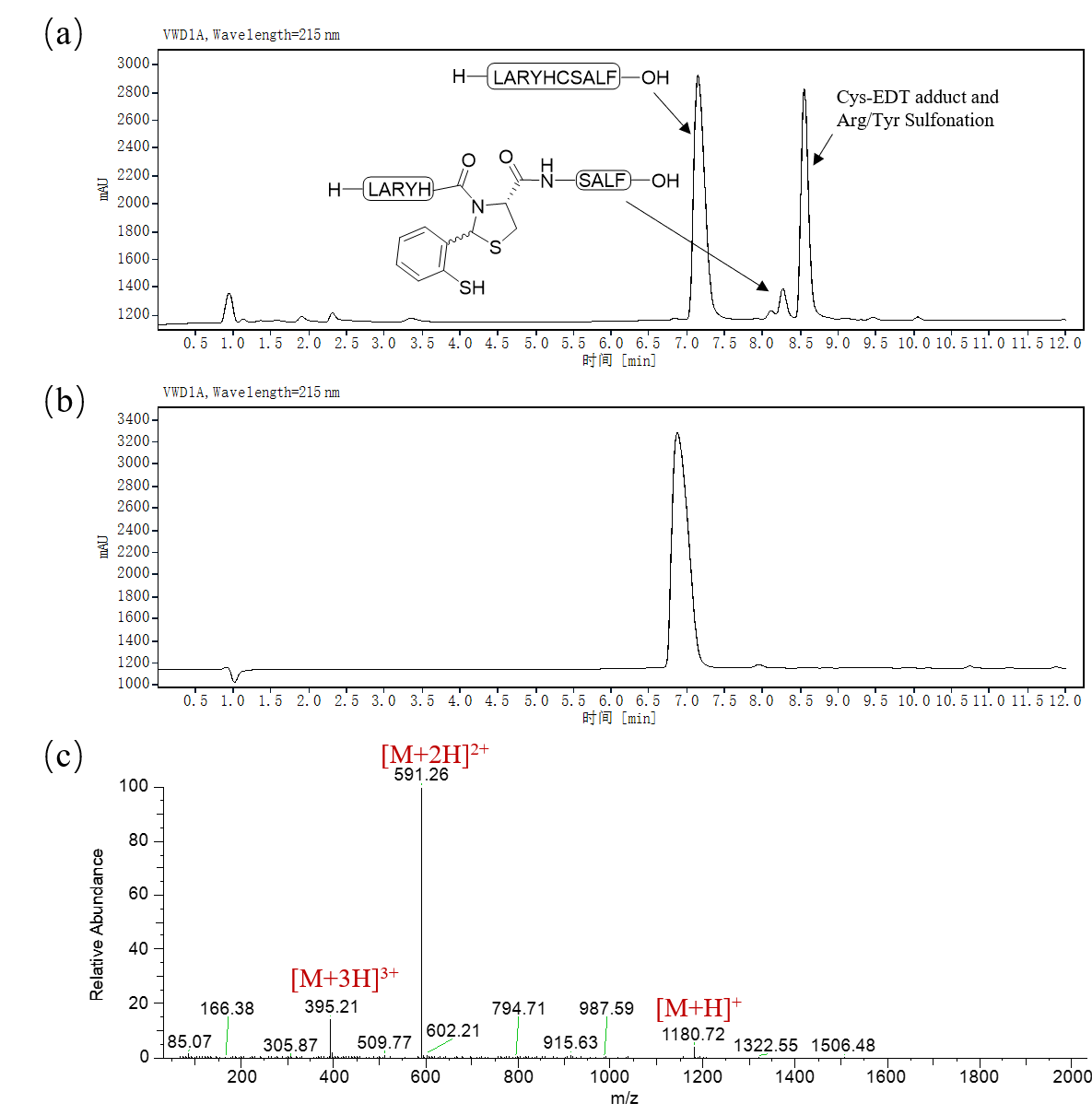


**Figure S68.** (a) Crude HPLC trace for acidolysis of the N,S-benzylidene acetal intermediate to generate H-LARYHCSALF-OH. Gradient: 20-70% ACN/H_2_O with 0.1% TFA over 12 min at a flow rate of 0.3 mL/min. (b) UV trace of purified H-LARYHCSALF-OH. Gradient: 20-70% ACN/H_2_O with 0.1% TFA over 12 min at a flow rate of 0.3 mL/min. (c) Corresponding MS of purified H-LARYHCSALF-OH. ESI-MS calcd.for C_54_H_81_N_15_O_13_S [M+H]^+^ m/z = 1180.59, found 1180.72; [M+2H]^2+^ m/z = 590.80, found 591.26; [M+3H]^3+^ m/z = 394.20, found 395.21.

10.6 H-LARYK-CO-TSAL+ H-CSALF-OH

The ligation between H-LARYK-CO-TSAL (4.0 mg, 5.2 μmol) and H-CSALF-OH (3.4 mg, 6.3 μmol) was performed as described in the **General procedure for TSAL ester-Cys/Penicillamine ligation**. Purification via preparative HPLC (20-70% ACN/H_2_O over 40 min, 0.1% TFA) followed by lyophilization afforded the N,S-benzylidene acetal intermediate (4.2 mg, 76% isolated yield) as white solids. And the intermediate (2.0 mg) was then treated with TFA/EDT/TMSOTf (90/5/5, v/v/v) for 4 h and carried out as the **General procedure for Acidolysis**. Purification via preparative HPLC (20-70% ACN/H_2_O over 40 min, 0.1%TFA) followed by lyophilization afforded the H-LARYKCSALF-OH (1.4 mg, 78% yield) as white solids.


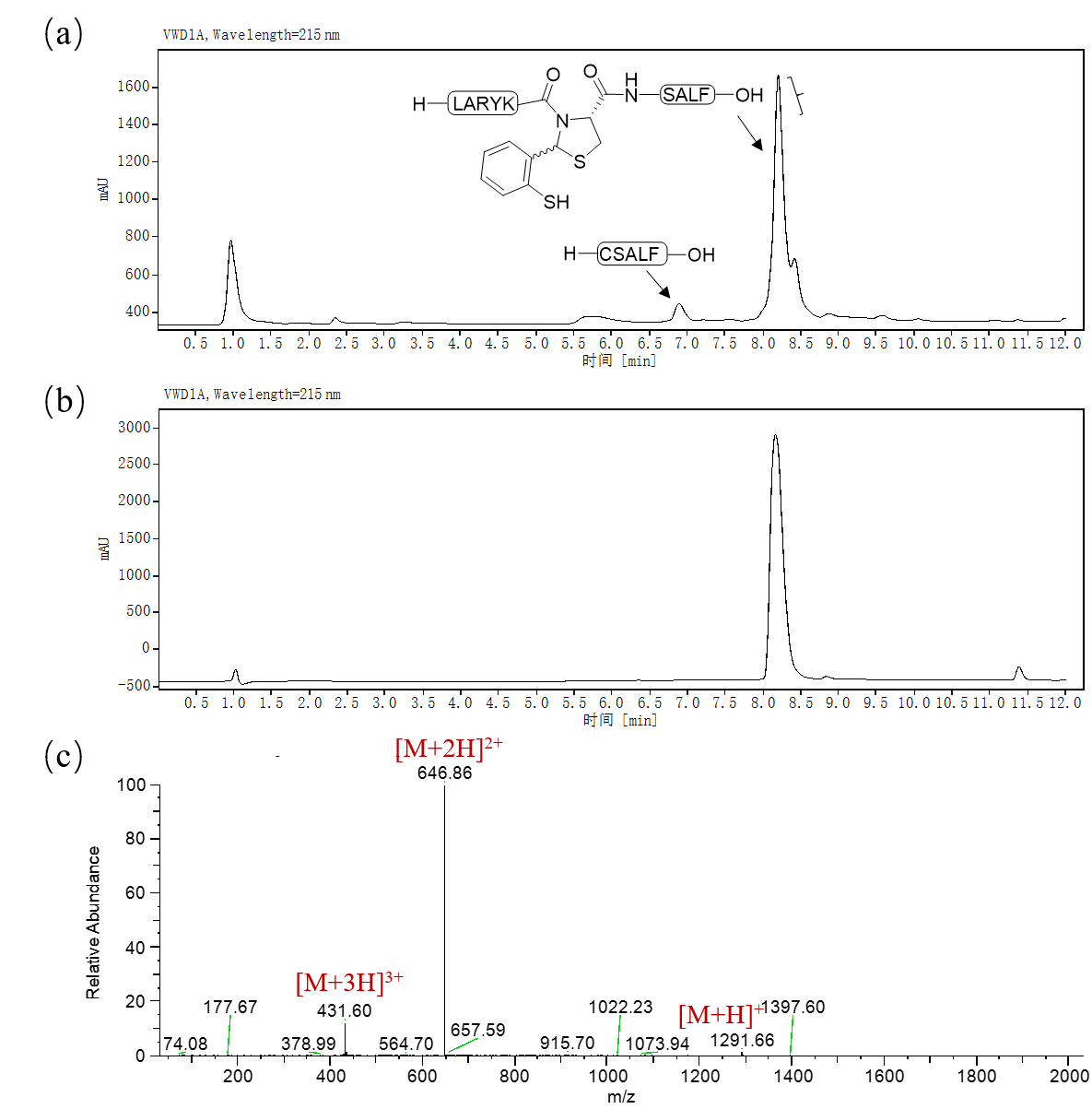


**Figure S69.** (a) Crude HPLC trace for the TSAL ester-Cys ligation between H-LARYK-CO-TSAL and H-CSALF-OH. Gradient: 20-70% ACN/H_2_O with 0.1% TFA over 12 min at a flow rate of 0.3 mL/min. (b) UV trace of the purified N,S-benzylidene acetal intermediate. Gradient: 20-70% ACN/H_2_O with 0.1% TFA over 12 min at a flow rate of 0.3 mL/min. (c) Corresponding MS of the purified N,S-benzylidene acetal intermediate. ESI-MS calcd.for C_61_H_90_N_14_O_13_S_2_ [M+H]^+^ m/z = 1291.63, found 1291.66; [M+2H]^2+^ m/z = 646.32, found 646.86; [M+3H]^3+^ m/z = 431.21, found 431.60.


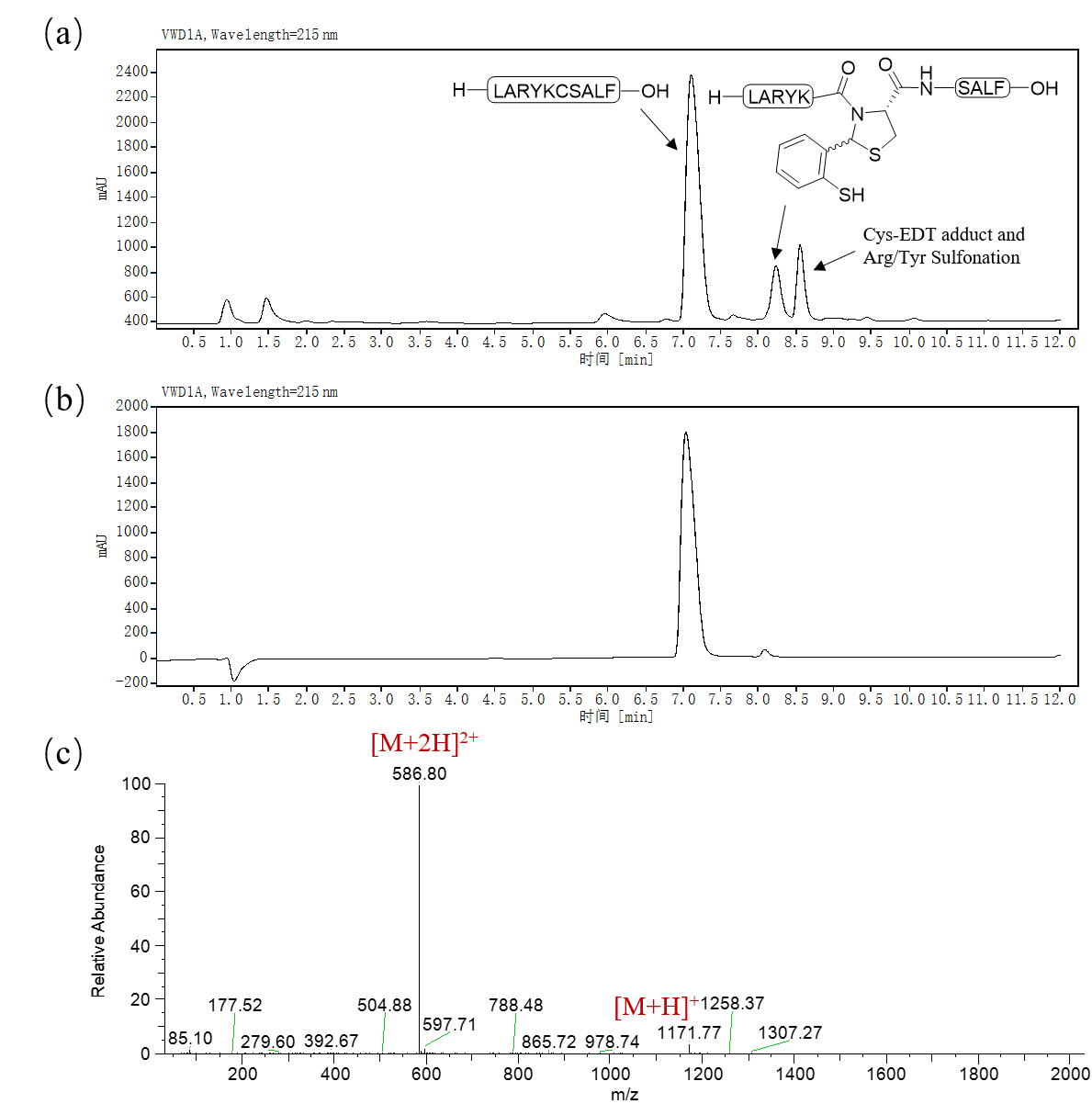


**Figure S70.** (a) Crude HPLC trace for acidolysis of the N,S-benzylidene acetal intermediate to generate H-LARYKCSALF-OH. Gradient: 20-70% ACN/H_2_O with 0.1% TFA over 12 min at a flow rate of 0.3 mL/min. (b) UV trace of purified H-LARYKCSALF-OH. Gradient: 20-70% ACN/H_2_O with 0.1% TFA over 12 min at a flow rate of 0.3 mL/min. (c) Corresponding MS of purified H-LARYKCSALF-OH. ESI-MS calcd.for C_54_H_86_N_14_O_13_S [M+H]^+^ m/z = 1171.62, found 1171.77; [M+2H]^2+^ m/z = 586.31, found 586.80.

10.7 H-LARYM-CO-TSAL+ H-CSALF-OH

The ligation between H-LARYM-CO-TSAL (6.4 mg, 8.3 μmol) and H-CSALF-OH (5.4 mg, 10.0 μmol) was performed as described in the **General procedure for TSAL ester-Cys/Penicillamine ligation**. Purification via preparative HPLC (20-70% ACN/H_2_O over 40 min, 0.1% TFA) followed by lyophilization afforded the N,S-benzylidene acetal intermediate (4.4 mg, 55% yield) as white solids. And the intermediate (1.3 mg) was then treated with TFA/EDT/TMSOTf (90/5/5, v/v/v) for 4 h and carried out as the **General procedure for Acidolysis**. Purification via preparative HPLC (20-70% ACN/H_2_O over 40 min, 0.1%TFA) followed by lyophilization afforded the H-LARYMCSALF-OH (0.9 mg, 76% yield) as white solids.


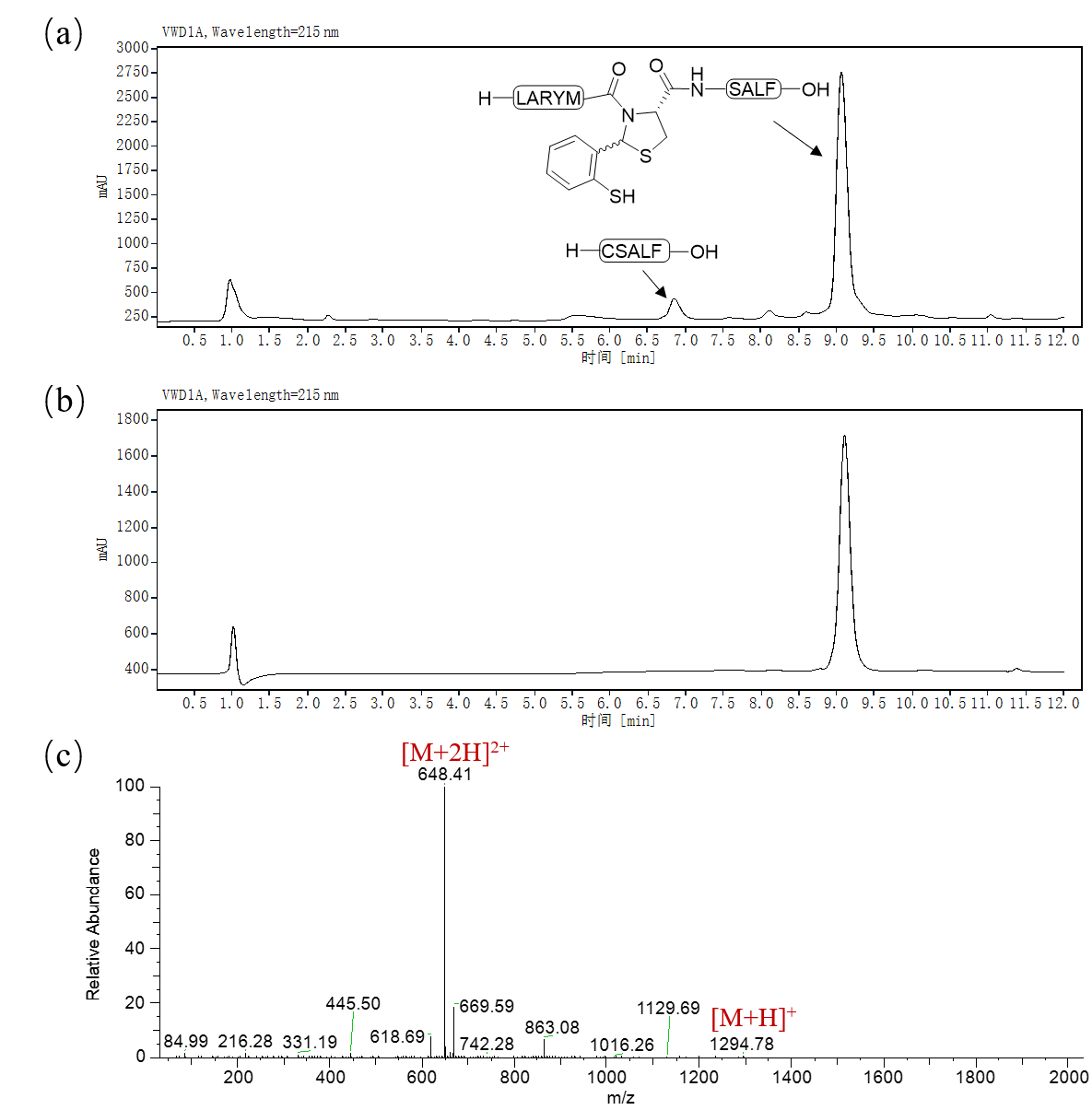


**Figure S71.** (a) Crude HPLC trace for the TSAL ester-Cys ligation between H-LARYM-CO-TSAL and H-CSALF-OH. Gradient: 20-70% ACN/H_2_O with 0.1% TFA over 12 min at a flow rate of 0.3 mL/min. (b) UV trace of the purified N,S-benzylidene acetal intermediate. Gradient: 20-70% ACN/H_2_O with 0.1% TFA over 12 min at a flow rate of 0.3 mL/min. (c) Corresponding MS of the purified N,S-benzylidene acetal intermediate. ESI-MS calcd.for C_60_H_87_N_13_O_13_S_3_ [M+H]^+^ m/z = 1294.57, found 1294.78; [M+2H]^2+^ m/z = 647.79, found 648.41.


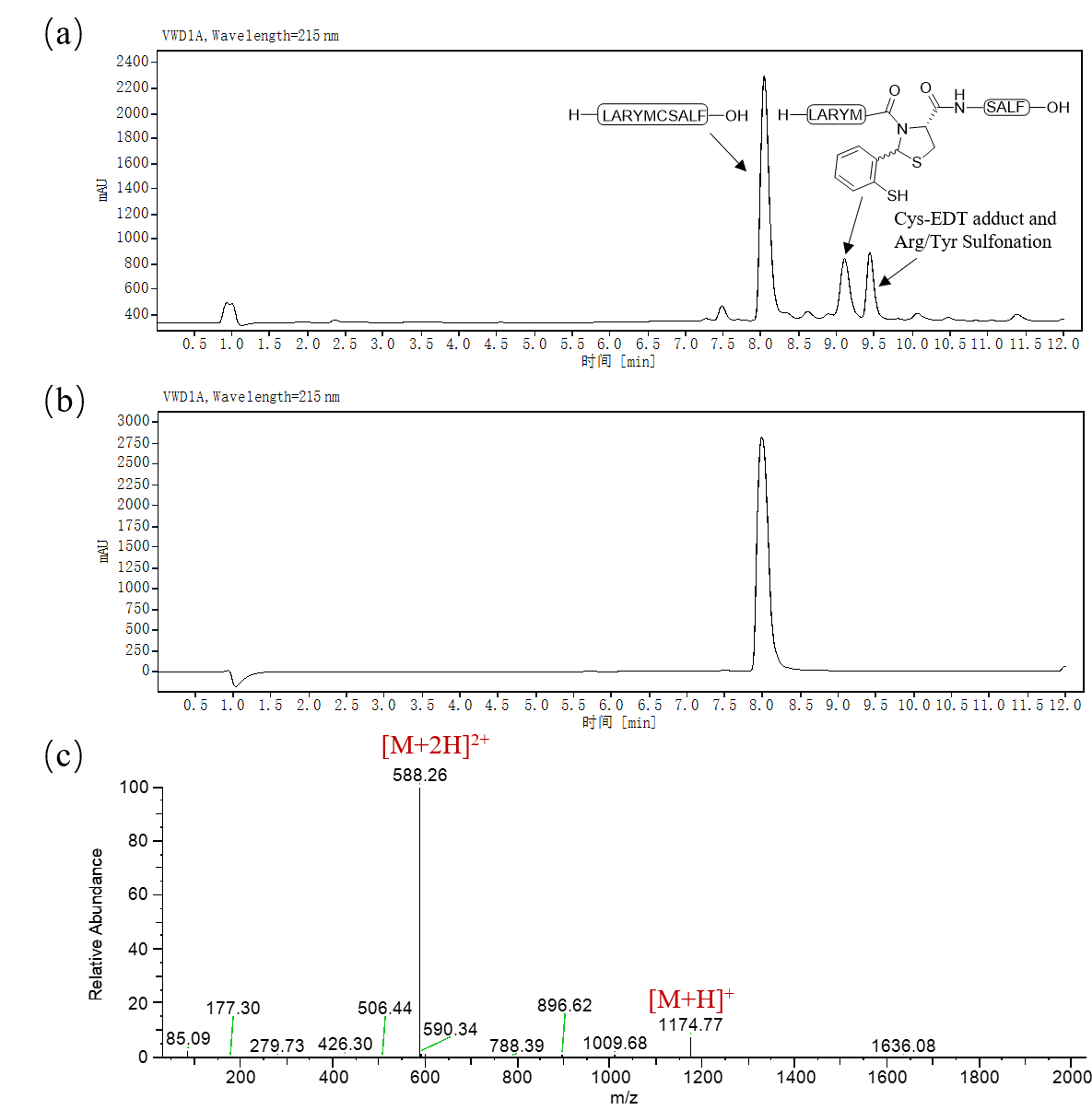


**Figure S72.** (a) Crude HPLC trace for acidolysis of the N,S-benzylidene acetal intermediate to generate H-LARYMCSALF-OH. Gradient: 20-70% ACN/H_2_O with 0.1% TFA over 12 min at a flow rate of 0.3 mL/min. (b) UV trace of purified H-LARYMCSALF-OH. Gradient: 20-70% ACN/H_2_O with 0.1% TFA over 12 min at a flow rate of 0.3 mL/min. (c) Corresponding MS of purified H-LARYMCSALF-OH. ESI-MS calcd.for C_53_H_83_N_13_O_13_S_2_ [M+H]^+^ m/z = 1174.57, found 1174.77; [M+2H]^2+^ m/z = 587.79, found 588.26.

10.8 H-LARYS-CO-TSAL+ H-CSALF-OH

The ligation between H-LARYS-CO-TSAL (5.4 mg, 7.4 μmol) and H-CSALF-OH (4.8 mg, 8.9 μmol) was performed as described in the **General procedure for TSAL ester-Cys/Penicillamine ligation**. Purification via preparative HPLC (20-70% ACN/H_2_O over 40 min, 0.1% TFA) followed by lyophilization afforded the N,S-benzylidene acetal intermediate (3.6 mg, 51% isolated yield) as white solids. And the intermediate (1.8 mg) was then treated with TFA/EDT/TMSOTf (90/5/5, v/v/v) for 4 h and carried out as the **General procedure for Acidolysis**. Purification via preparative HPLC (20-70% ACN/H_2_O over 40 min, 0.1%TFA) followed by lyophilization afforded the H-LARYSCSALF-OH (1.4 mg, 86% yield) as white solids.


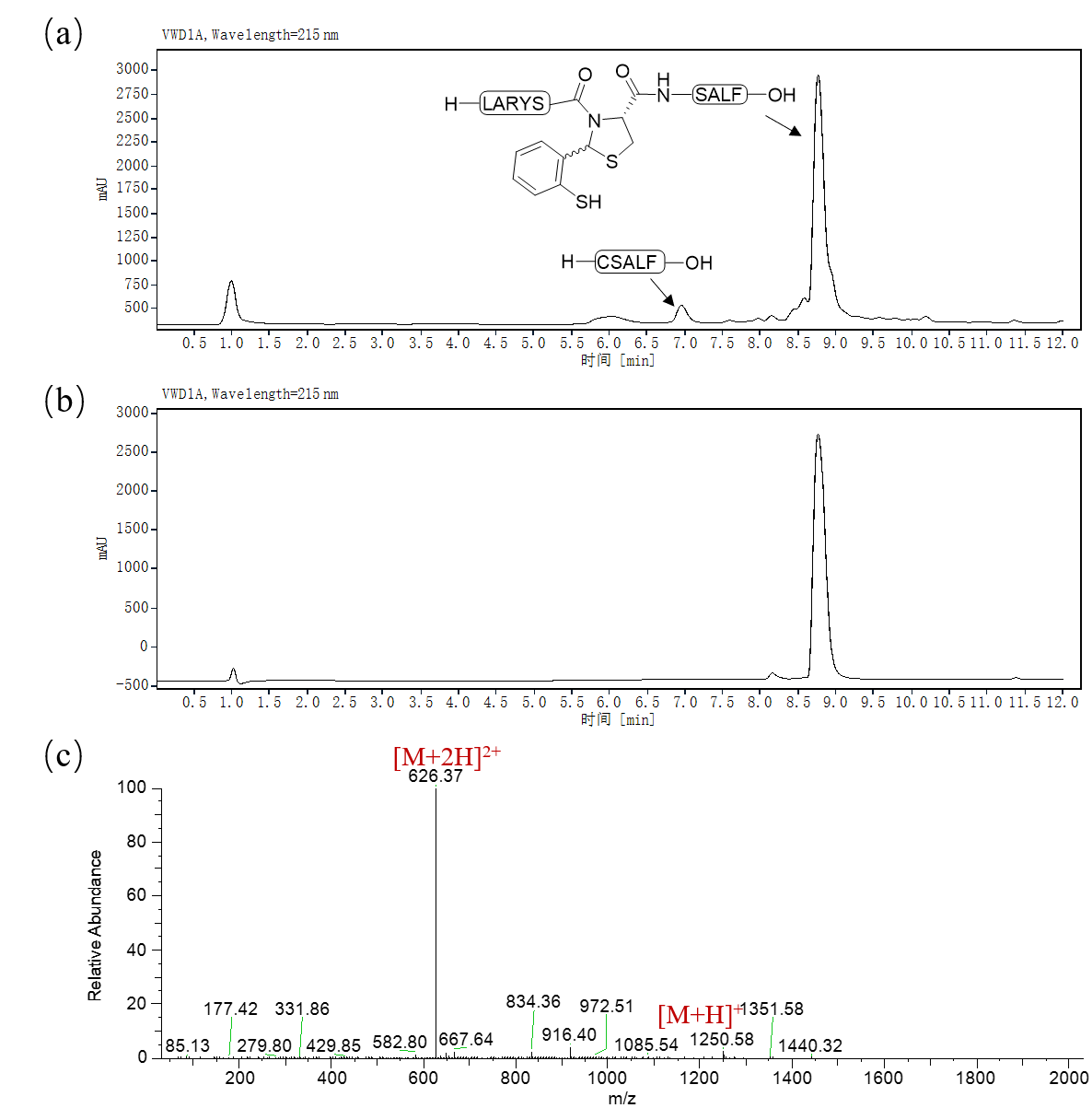


**Figure S73.** (a) Crude HPLC trace for the TSAL ester-Cys ligation between H-LARYS-CO-TSAL and H-CSALF-OH. Gradient: 20-70% ACN/H_2_O with 0.1% TFA over 12 min at a flow rate of 0.3 mL/min. (b) UV trace of the purified N,S-benzylidene acetal intermediate. Gradient: 20-70% ACN/H_2_O with 0.1% TFA over 12 min at a flow rate of 0.3 mL/min. (c) Corresponding MS of the purified N,S-benzylidene acetal intermediate. ESI-MS calcd.for C_58_H_83_N_13_O_14_S_2_ [M+H]^+^ m/z = 1250.56, found 1250.58; [M+2H]^2+^ m/z = 625.78, found 626.37.


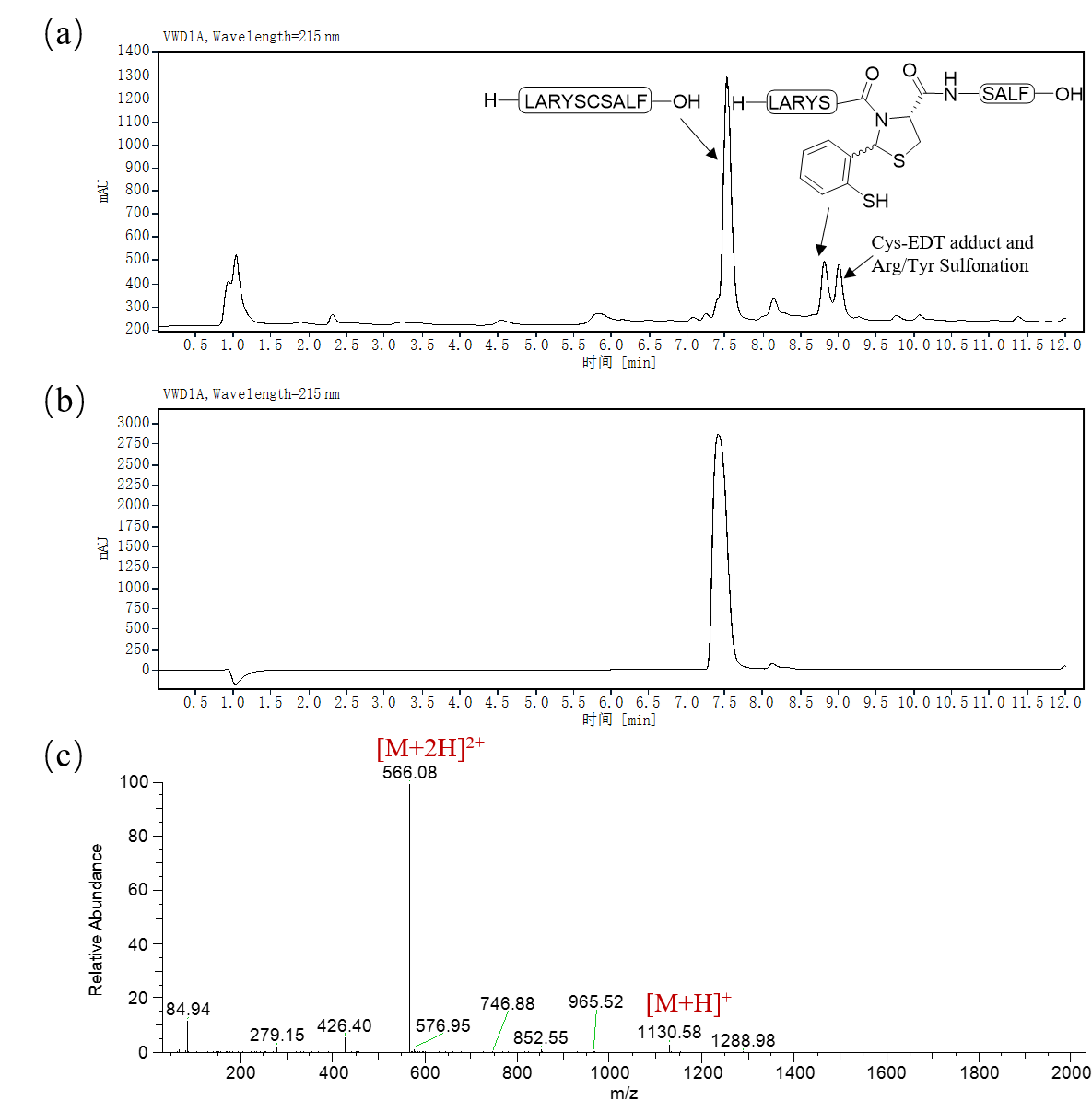


**Figure S74.** (a) Crude HPLC trace for acidolysis of the N,S-benzylidene acetal intermediate to generate H-LARYSCSALF-OH. Gradient: 20-70% ACN/H_2_O with 0.1% TFA over 12 min at a flow rate of 0.3 mL/min. (b) UV trace of purified H-LARYSCSALF-OH. Gradient: 20-70% ACN/H_2_O with 0.1% TFA over 12 min at a flow rate of 0.3 mL/min. (c) Corresponding MS of purified H-LARYSCSALF-OH. ESI-MS calcd.for C_51_H_79_N_13_O_14_S [M+H]^+^ m/z = 1130.56, found 1130.58; [M+2H]^2+^ m/z = 565.78, found 566.08.

10.9 H-LARYT-CO-TSAL+ H-CSALF-OH

The ligation between H-LARYT-CO-TSAL (4.9 mg, 6.6 μmol) and H-CSALF-OH (4.3 mg, 8.0 μmol) was performed as described in the **General procedure for TSAL ester-Cys/Penicillamine ligation**. Purification via preparative HPLC (20-70% ACN/H_2_O over 40 min, 0.1% TFA) followed by lyophilization afforded the N,S-benzylidene acetal intermediate (4.6 mg, 66% isolated yield) as white solids. And the intermediate (2.7 mg) was then treated with TFA/EDT/TMSOTf (90/5/5, v/v/v) for 11 h and carried out as the **General procedure for Acidolysis**. Purification via preparative HPLC (20-70% ACN/H_2_O over 40 min, 0.1%TFA) followed by lyophilization afforded the H-LARYTCSALF-OH (1.9 mg, 78% yield) as white solids.


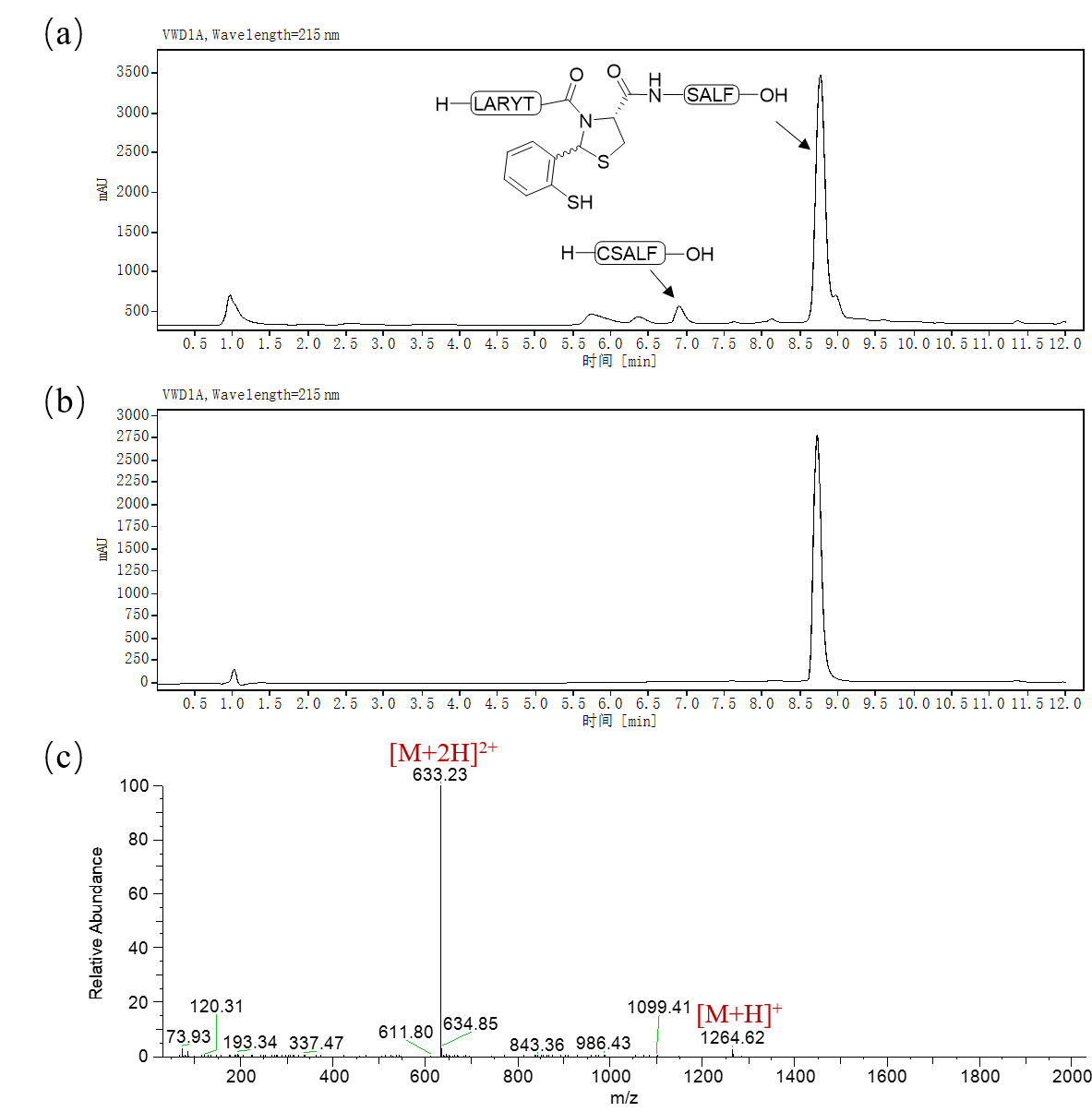


**Figure S75.** (a) Crude HPLC trace for the TSAL ester-Cys ligation between H-LARYT-CO-TSAL and H-CSALF-OH. Gradient: 20-70% ACN/H_2_O with 0.1% TFA over 12 min at a flow rate of 0.3 mL/min. (b) UV trace of the purified N,S-benzylidene acetal intermediate. Gradient: 20-70% ACN/H_2_O with 0.1% TFA over 12 min at a flow rate of 0.3 mL/min. (c) Corresponding MS of the purified N,S-benzylidene acetal intermediate. ESI-MS calcd.for C_59_H_85_N_13_O_14_S_2_ [M+H]^+^ m/z = 1264.58, found 1264.62; [M+2H]^2+^ m/z = 632.79, found 633.23.


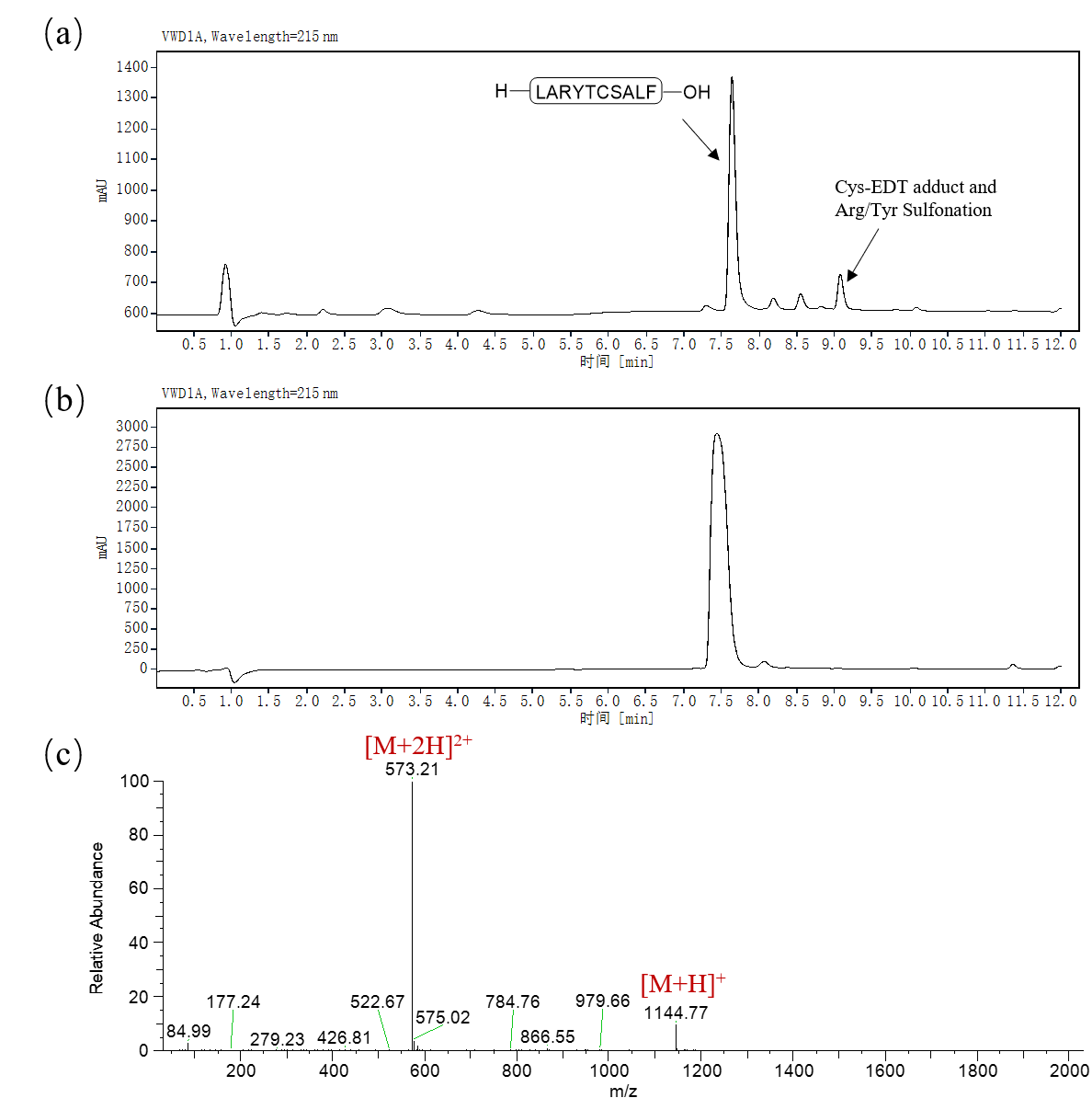


**Figure S76.** (a) Crude HPLC trace for acidolysis of the N,S-benzylidene acetal intermediate to generate H-LARYTCSALF-OH. Gradient: 20-70% ACN/H_2_O with 0.1% TFA over 12 min at a flow rate of 0.3 mL/min. (b) UV trace of purified H-LARYTCSALF-OH. Gradient: 20-70% ACN/H_2_O with 0.1% TFA over 12 min at a flow rate of 0.3 mL/min. (c) Corresponding MS of purified H-LARYTCSALF-OH. ESI-MS calcd.for C_52_H_81_N_13_O_14_S [M+H]^+^ m/z = 1144.57, found 1144.77; [M+2H]^2+^ m/z = 572.79, found 573.21.

10.10 H-LARYP-CO-TSAL+ H-CSALF-OH

The ligation between H-LARYP-CO-TSAL (2.0 mg, 2.7 μmol) and H-CSALF-OH (1.5 mg, 2.7 μmol) was performed as described in the **General procedure for TSAL ester-Cys/Penicillamine ligation**. Purification via preparative HPLC (20-70% ACN/H_2_O over 40 min, 0.1% TFA) followed by lyophilization afforded the N,S-benzylidene acetal intermediate (2.2 mg, 65% yield) as white solids. And the intermediate (2.2 mg) was then treated with TFA/EDT/TMSOTf (90/5/5, v/v/v) for 4 h and carried out as the **General procedure for Acidolysis**. Purification via preparative HPLC (20-70% ACN/H_2_O over 40 min, 0.1%TFA) followed by lyophilization afforded the H-LARYPCSALF-OH (1.4 mg, 70% yield) as white solids.


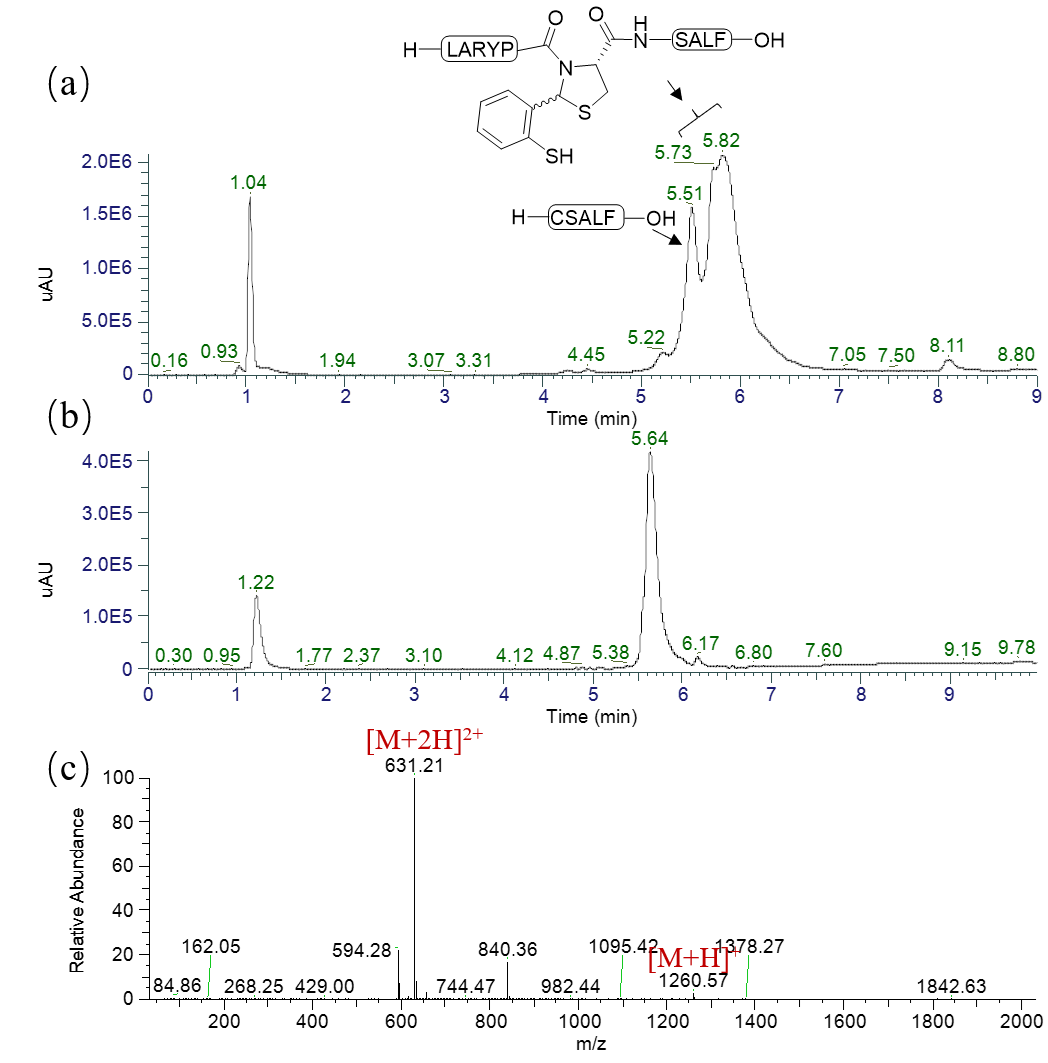


**Figure S77.** (a) Crude HPLC trace for the TSAL ester-Cys ligation between H-LARYP-CO-TSAL and H-CSALF-OH. Gradient: 10-80% ACN/H_2_O with 0.1% TFA over 9 min at a flow rate of 0.3 mL/min. (b) UV trace of the purified N,S-benzylidene acetal intermediate. Gradient: 5-95% ACN/H_2_O with 0.1% TFA over 10 min at a flow rate of 0.3 mL/min. (c) Corresponding MS of the purified N,S-benzylidene acetal intermediate. ESI-MS calcd.for C_60_H_85_N_13_O_13_S_2_ [M+H]^+^ m/z = 1260.58, found 1260.57; [M+2H]^2+^ m/z = 630.79, found 631.21.


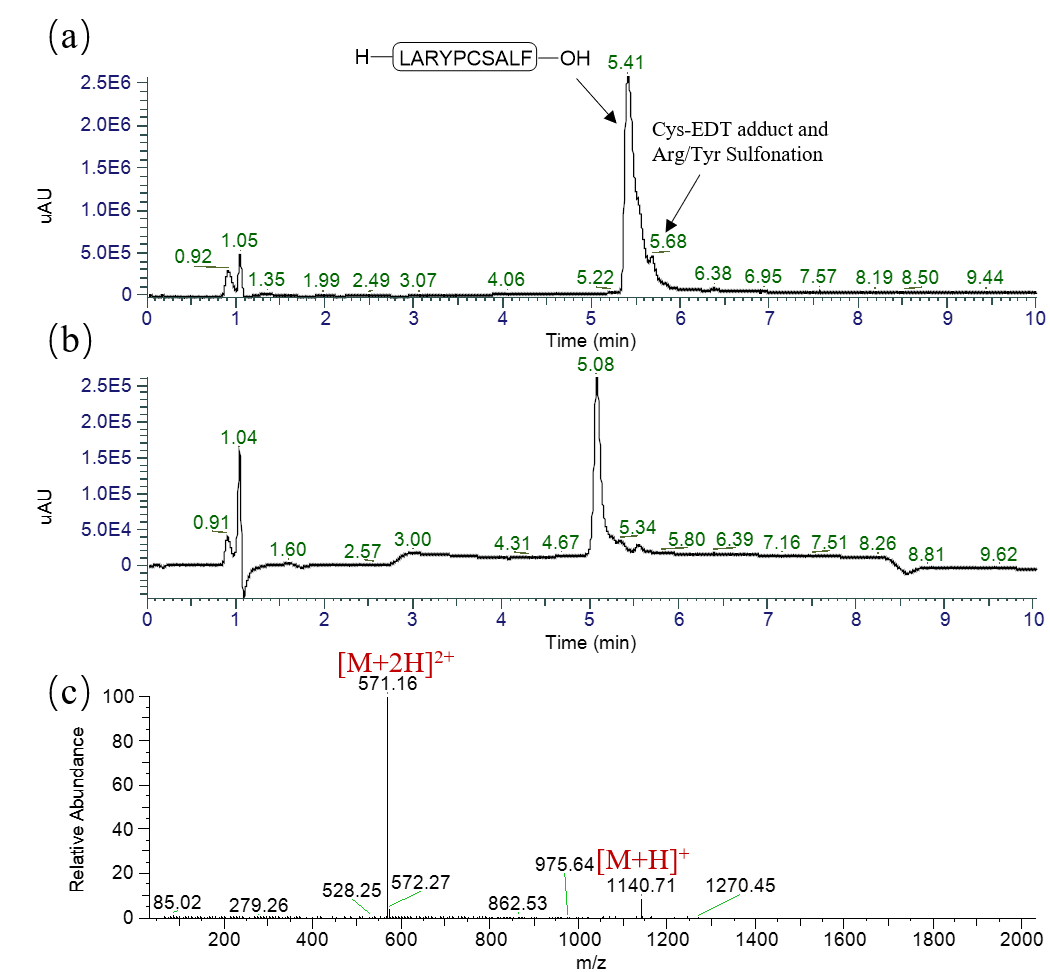


**Figure S78.** (a) Crude HPLC trace for acidolysis of the N,S-benzylidene acetal intermediate to generate H-LARYPCSALF-OH. Gradient: 5-95% ACN/H_2_O with 0.1% TFA over 10 min at a flow rate of 0.3 mL/min. (b) UV trace of purified H-LARYPCSALF-OH. Gradient: 10-95% ACN/H_2_O with 0.1% TFA over 10 min at a flow rate of 0.3 mL/min. (c) Corresponding MS of purified H-LARYPCSALF-OH. ESI-MS calcd.for C_53_H_81_N_13_O_13_S [M+H]^+^ m/z = 1140.58, found 1140.71; [M+2H]^2+^ m/z = 570.79, found 571.16.

10.11 H-LARYV-CO-TSAL+ H-CSALF-OH

The ligation between H-LARYV-CO-TSAL (1.6 mg, 2.2 μmol) and H-CSALF-OH (1.8 mg, 3.2 μmol) was performed as described in the **General procedure for TSAL ester-Cys/Penicillamine ligation**. Purification via preparative HPLC (20-70% ACN/H_2_O over 40 min, 0.1% TFA) followed by lyophilization afforded the N,S-benzylidene acetal intermediate (1.4 mg, 51% yield) as white solids. And the intermediate (1.4 mg) was then treated with TFA/EDT/TMSOTf (90/5/5, v/v/v) for 4 h and carried out as the **General procedure for Acidolysis**. Purification via preparative HPLC (20-70% ACN/H_2_O over 40 min, 0.1%TFA) followed by lyophilization afforded the H-LARYVCSALF-OH (1.0 mg, 78% yield) as white solids.


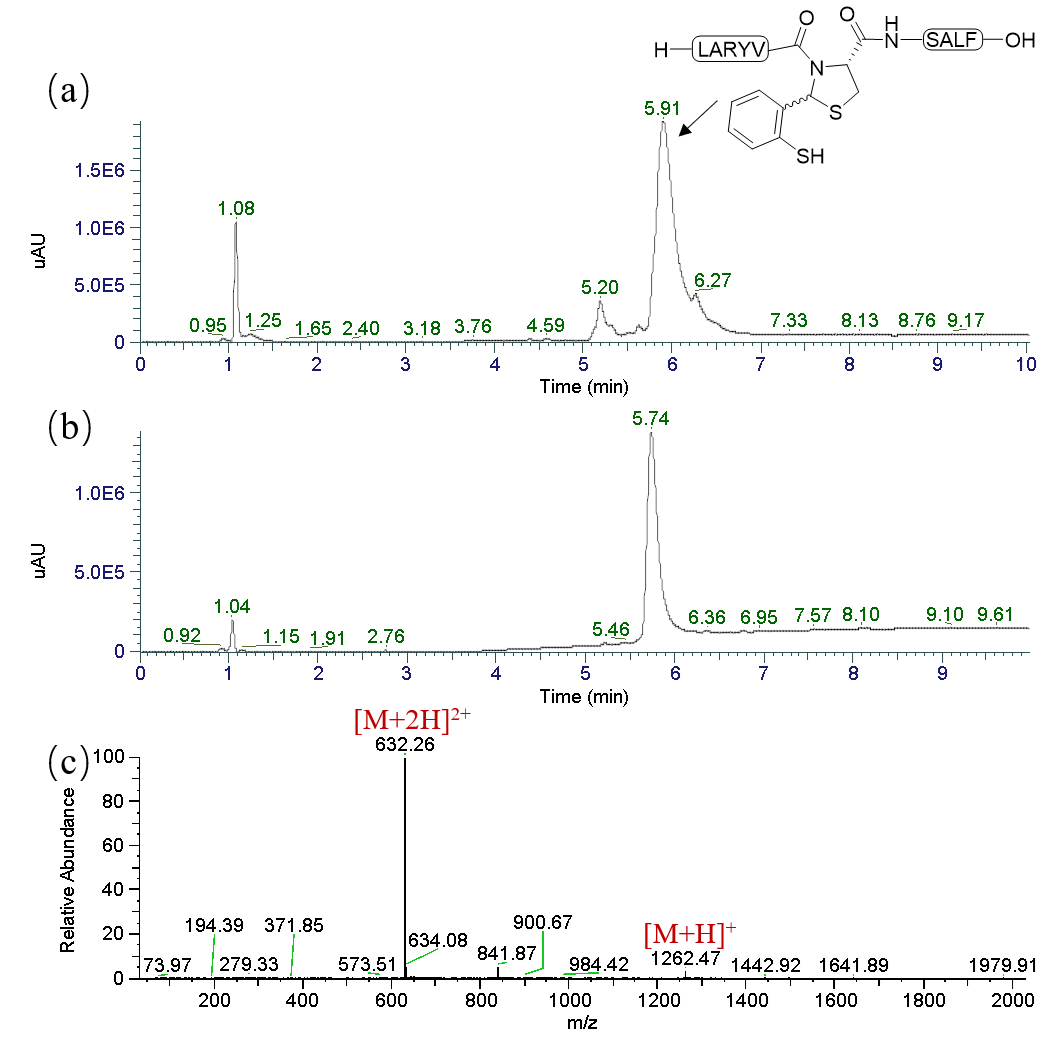


**Figure S79.** (a) Crude HPLC trace for the TSAL ester-Cys ligation between H-LARYV-CO-TSAL and H-CSALF-OH. Gradient: 10-80% ACN/H_2_O with 0.1% TFA over 10 min at a flow rate of 0.3 mL/min. (b) UV trace of the purified N,S-benzylidene acetal intermediate. Gradient: 5-95% ACN/H_2_O with 0.1% TFA over 10 min at a flow rate of 0.3 mL/min. (c) Corresponding MS of the purified N,S-benzylidene acetal intermediate. ESI-MS calcd.for C_60_H_87_N_13_O_13_S_2_ [M+H]^+^ m/z = 1262.60, found 1262.47; [M+2H]^2+^ m/z = 631.80, found 632.26.


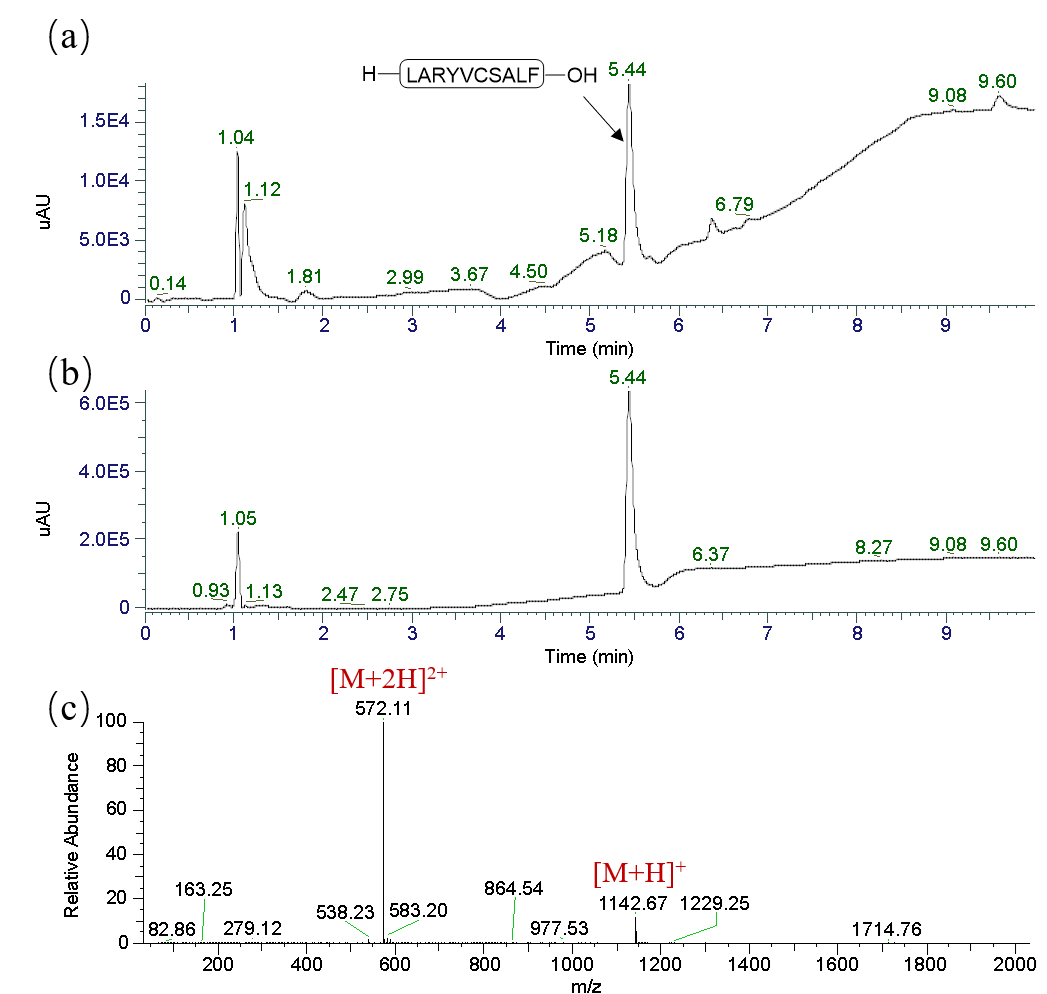


**Figure S80.** (a) Crude HPLC trace for acidolysis of the N,S-benzylidene acetal intermediate to generate H-LARYVCSALF-OH. Gradient: 5-95% ACN/H_2_O with 0.1% TFA over 10 min at a flow rate of 0.3 mL/min. (b) UV trace of purified H-LARYVCSALF-OH. Gradient: 5-95% ACN/H_2_O with 0.1% TFA over 10 min at a flow rate of 0.3 mL/min. (c) Corresponding MS of purified H-LARYVCSALF-OH. ESI-MS calcd.for C_53_H_83_N_13_O_13_S [M+H]^+^ m/z = 1142.60, found 1142.67; [M+2H]^2+^ m/z = 571.80, found 572.11.

10.12 H-LEAGR-CO-TSAL+ H-CSALF-OH

The ligation between H-LEAGR-CO-TSAL (2.6 mg, 3.9 μmol) and H-CSALF-OH (2.5 mg, 4.7 μmol) was performed as described in the **General procedure for TSAL ester-Cys/Penicillamine ligation**. Purification via preparative HPLC (20-70% ACN/H_2_O over 40 min, 0.1% TFA) followed by lyophilization afforded the N,S-benzylidene acetal intermediate (2.5 mg, 53% yield) as white solids. And the intermediate (1.4 mg) was then treated with TFA/EDT/TMSOTf (90/5/5, v/v/v) for 4 h and carried out as the **General procedure for Acidolysis**. Purification via preparative HPLC (20-70% ACN/H_2_O over 40 min, 0.1%TFA) followed by lyophilization afforded the H-LEAGRCSALF-OH (0.7 mg, 56% yield) as white solids.


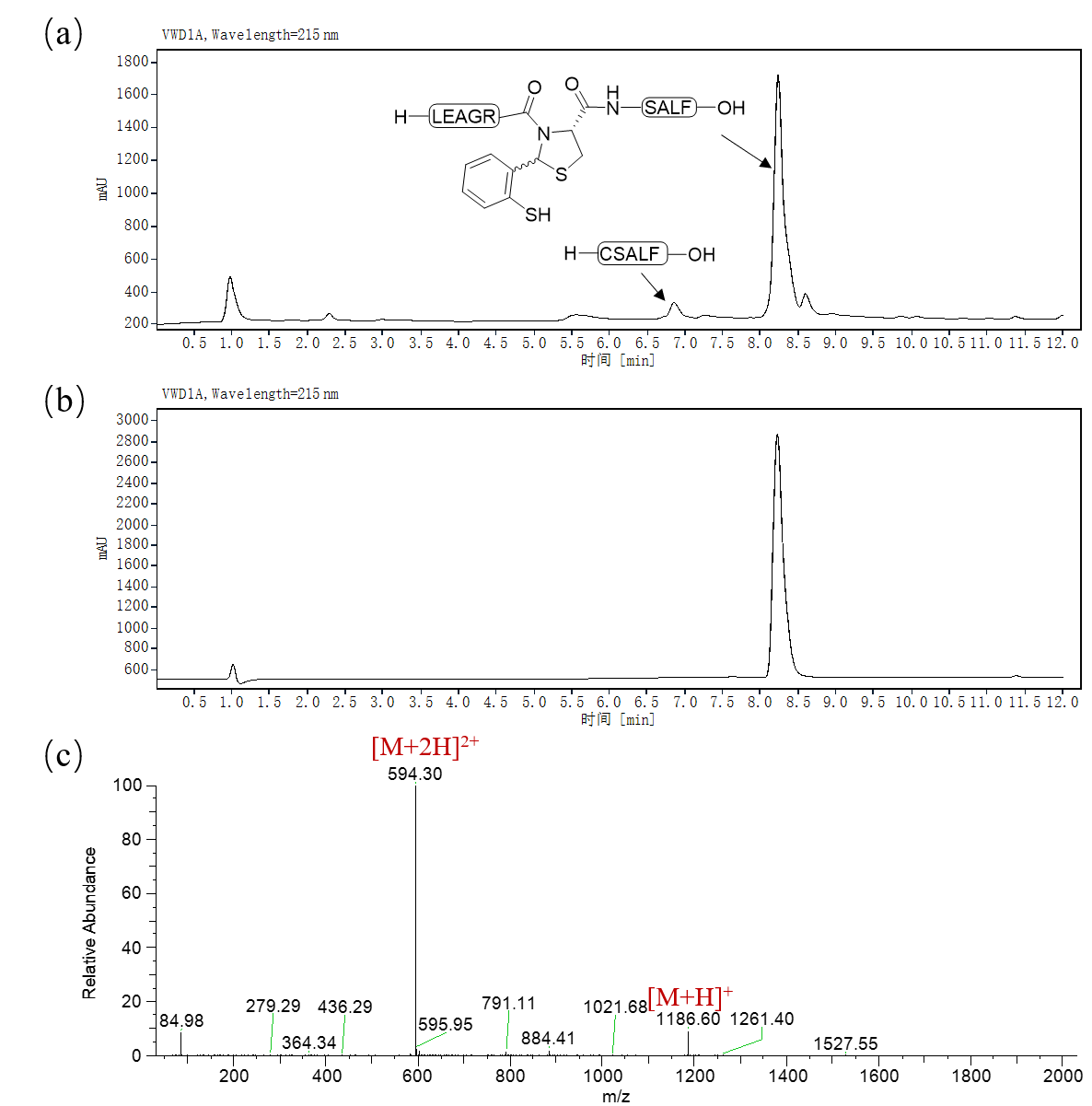


**Figure S81.** (a) Crude HPLC trace for the TSAL ester-Cys ligation between H-LEAGR-CO-TSAL and H-CSALF-OH. Gradient: 20-70% ACN/H_2_O with 0.1% TFA over 12 min at a flow rate of 0.3 mL/min. (b) UV trace of the purified N,S-benzylidene acetal intermediate. Gradient: 20-70% ACN/H_2_O with 0.1% TFA over 12 min at a flow rate of 0.3 mL/min. (c) Corresponding MS of the purified N,S-benzylidene acetal intermediate. ESI-MS calcd.for C_53_H_79_N_13_O_14_S_2_ [M+H]^+^ m/z = 1186.53, found 1186.60; [M+2H]^2+^ m/z = 593.77, found 594.30.


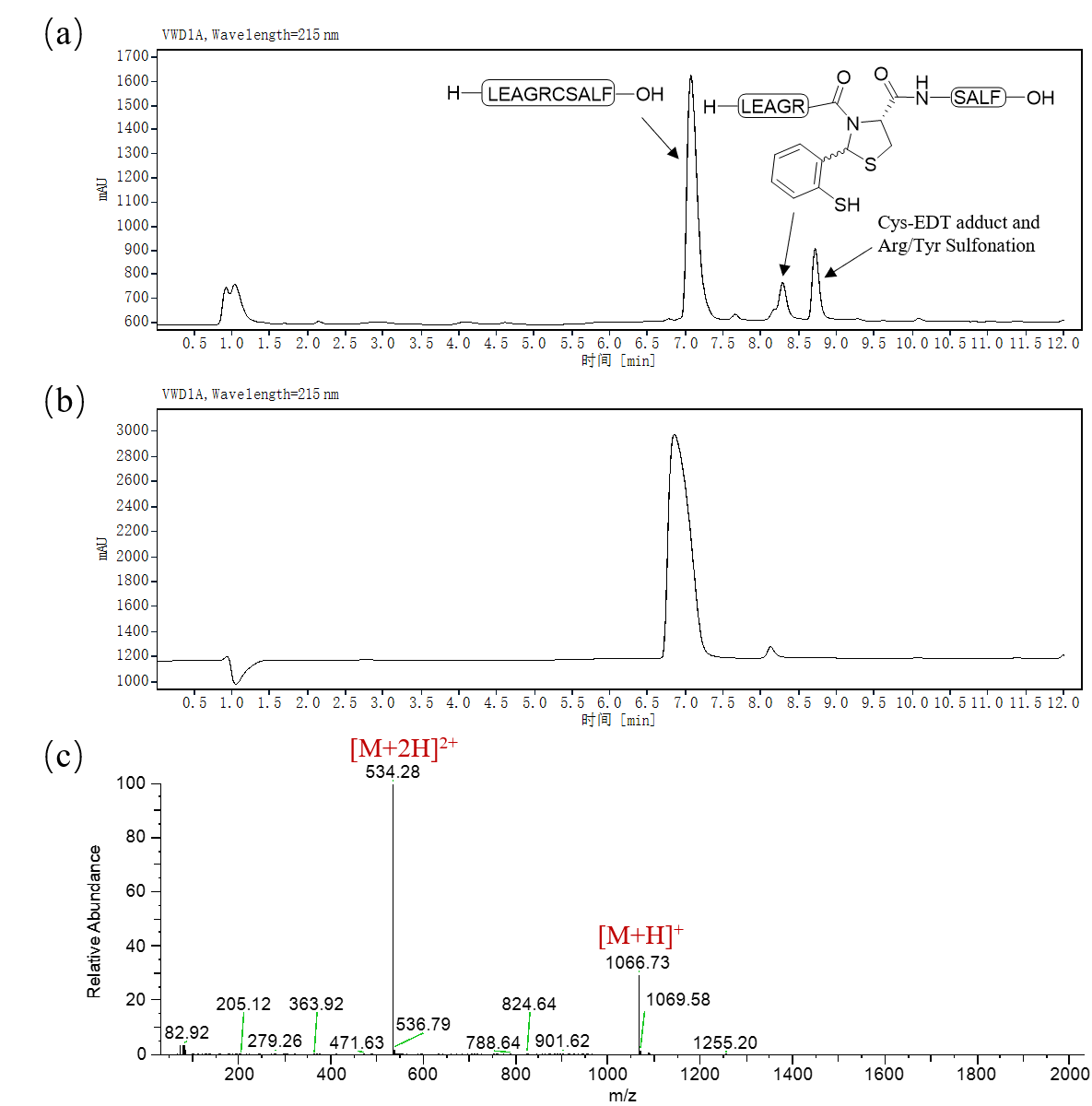


**Figure S82.** (a) Crude HPLC trace for acidolysis of the N,S-benzylidene acetal intermediate to generate H-LEAGRCSALF-OH. Gradient: 20-70% ACN/H_2_O with 0.1% TFA over 12 min at a flow rate of 0.3 mL/min. (b) UV trace of purified H-LEAGRCSALF-OH. Gradient: 20-70% ACN/H_2_O with 0.1% TFA over 12 min at a flow rate of 0.3 mL/min. (c) Corresponding MS of purified H-LEAGRCSALF-OH. ESI-MS calcd.for C_46_H_75_N_13_O_14_S [M+H]^+^ m/z = 1066.53, found 1066.73; [M+2H]^2+^ m/z = 533.77, found 534.28.

10.13 H-GDVGI-CO-TSAL+ H-CSALF-OH

The ligation between H-GDVGI-CO-TSAL (2.7 mg, 4.7 μmol) and H-CSALF-OH (3.8 mg, 7.0 μmol) was performed as described in the **General procedure for TSAL ester-Cys/Penicillamine ligation**. Purification via preparative HPLC (20-70% ACN/H_2_O over 40 min, 0.1% TFA) followed by lyophilization afforded the N,S-benzylidene acetal intermediate (2.5 mg, 49% yield) as white solids. And the intermediate (2.5 mg) was then treated with TFA/EDT/TMSOTf (90/5/5, v/v/v) for 4 h and carried out as the **General procedure for Acidolysis**. Purification via preparative HPLC (20-70% ACN/H_2_O over 40 min, 0.1%TFA) followed by lyophilization afforded the H-GDVGICSALF-OH (1.6 mg, 72% yield) as white solids.


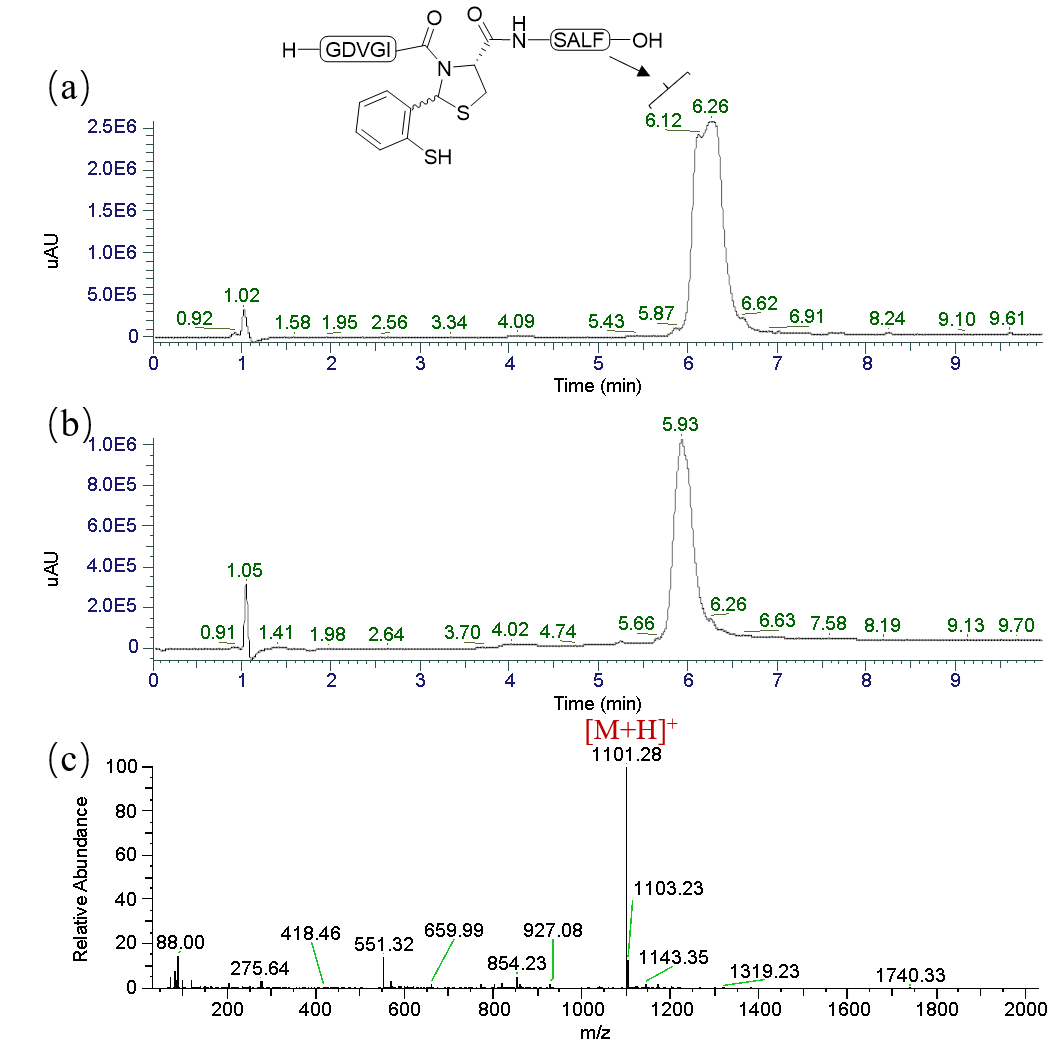


**Figure S83.** (a) Crude HPLC trace for the TSAL ester-Cys ligation between H-GDVGI-CO-TSAL and H-CSALF-OH. Gradient: 5-95% ACN/H_2_O with 0.1% TFA over 10 min at a flow rate of 0.3 mL/min. (b) UV trace of the purified N,S-benzylidene acetal intermediate. Gradient: 5-95% ACN/H_2_O with 0.1% TFA over 10 min at a flow rate of 0.3 mL/min. (c) Corresponding MS of the purified N,S-benzylidene acetal intermediate. ESI-MS calcd.for C_50_H_72_N_10_O_14_S_2_ [M+H]^+^ m/z = 1101.47, found 1101.28.


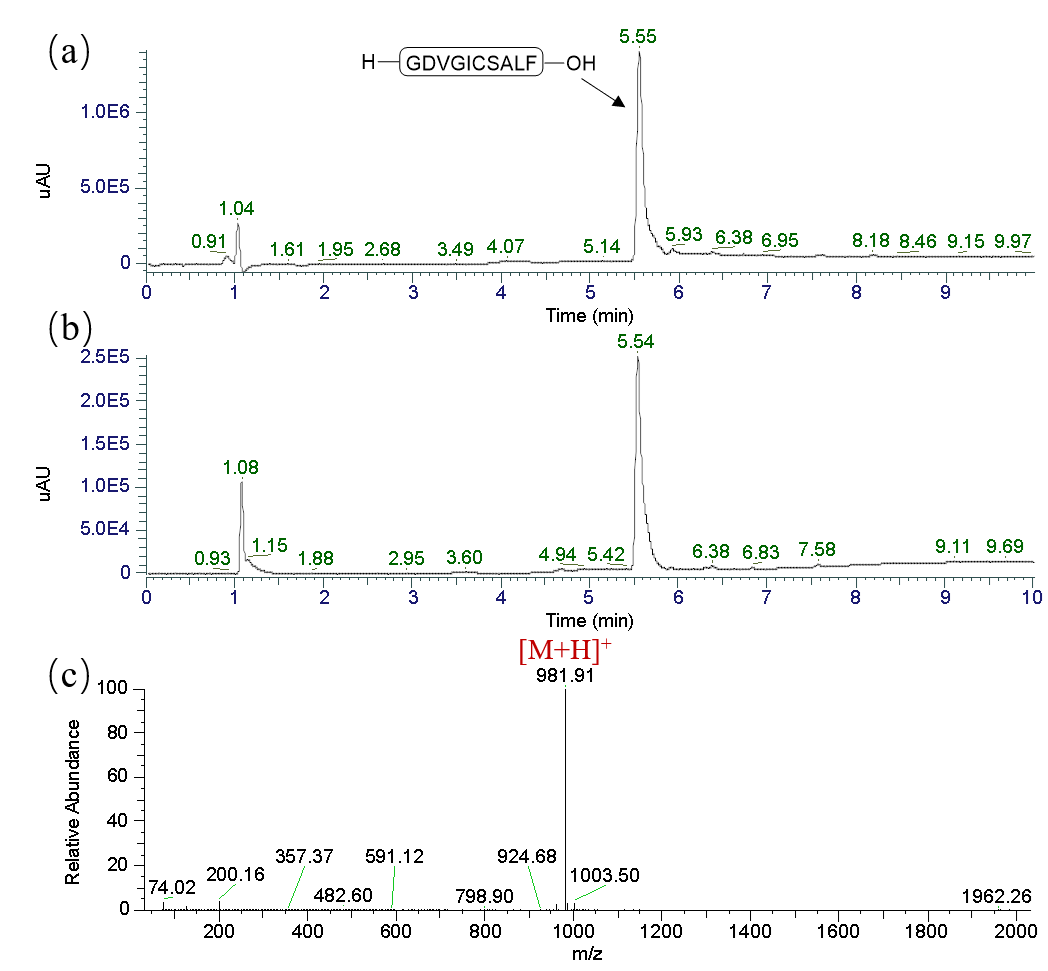


**Figure S84.** (a) Crude HPLC trace for acidolysis of the N,S-benzylidene acetal intermediate to generate H-GDVGICSALF-OH. Gradient: 5-95% ACN/H_2_O with 0.1% TFA over 10 min at a flow rate of 0.3 mL/min. (b) UV trace of purified H-GDVGICSALF-OH. Gradient: 5-95% ACN/H_2_O with 0.1% TFA over 10 min at a flow rate of 0.3 mL/min. (c) Corresponding MS of purified H-GDVGICSALF-OH. ESI-MS calcd.for C_43_H_68_N_10_O_14_S [M+H]^+^ m/z = 981.46, found 981.91.

10.14 H-AGVEGL-CO-TSAL+ H-CSALF-OH

The ligation between H-AGVEGL-CO-TSAL (1.0 mg, 1.5 μmol) and H-CSALF-OH (0.8 mg, 1.5 μmol) was performed as described in the **General procedure for TSAL ester-Cys/Penicillamine ligation**. Purification via preparative HPLC (20-70% ACN/H_2_O over 40 min, 0.1% TFA) followed by lyophilization afforded the N,S-benzylidene acetal intermediate (1.1 mg, 62% yield) as white solids. And the intermediate (1.1 mg) was then treated with TFA/EDT/TMSOTf (90/5/5, v/v/v) for 4 h and carried out as the **General procedure for Acidolysis**. Purification via preparative HPLC (20-70% ACN/H_2_O over 40 min, 0.1%TFA) followed by lyophilization afforded the H-AGVEGLCSALF-OH (0.6 mg, 61% yield) as white solids.


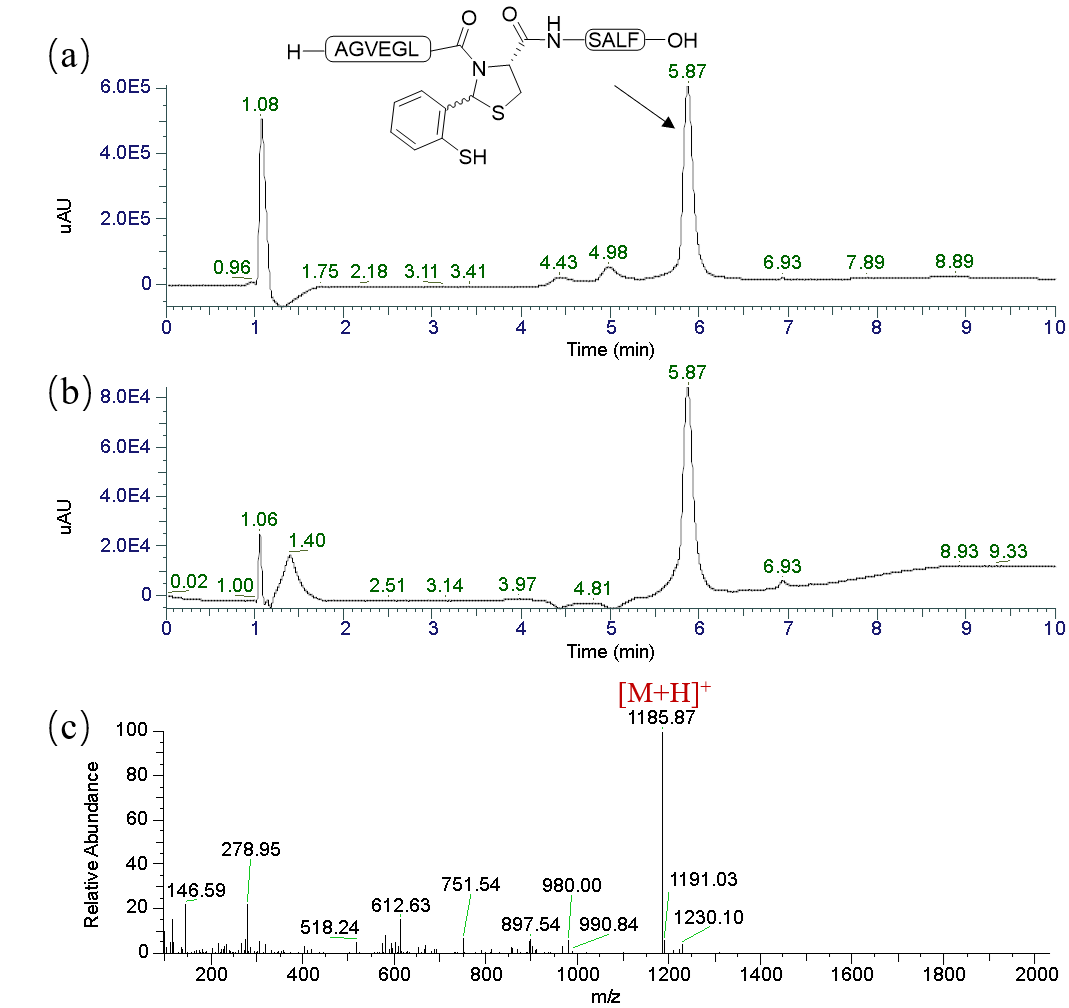


**Figure S85.** (a) Crude HPLC trace for the TSAL ester-Cys ligation between H-AGVEGL-CO-TSAL and H-CSALF-OH. Gradient: 5-95% ACN/H_2_O with 0.1% TFA over 10 min at a flow rate of 0.3 mL/min. (b) UV trace of the purified N,S-benzylidene acetal intermediate. Gradient: 5-95% ACN/H_2_O with 0.1% TFA over 10 min at a flow rate of 0.3 mL/min. (c) Corresponding MS of the purified N,S-benzylidene acetal intermediate. ESI-MS calcd.for C_54_H_79_N_11_O_15_S_2_ [M+H]^+^ m/z = 1186.52, found 1185.87.


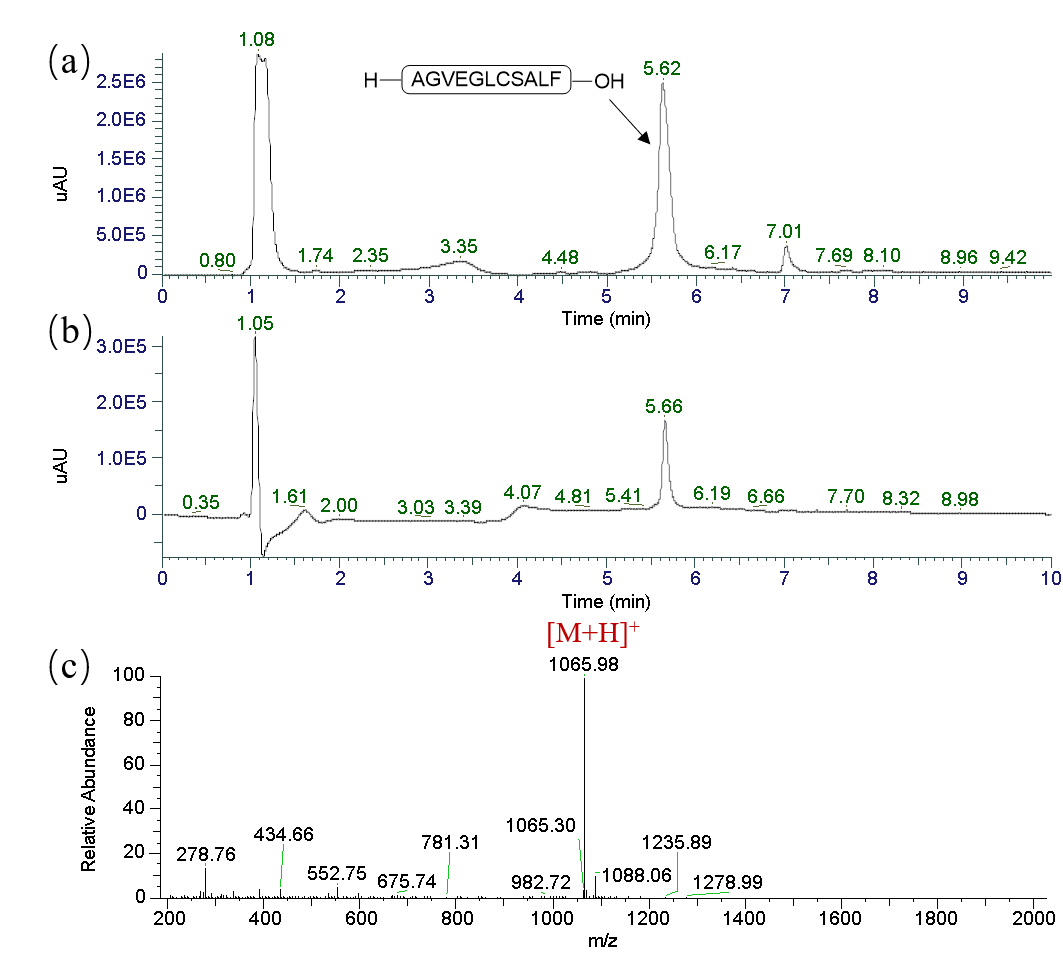


**Figure S86.** (a) Crude HPLC trace for acidolysis of the N,S-benzylidene acetal intermediate to generate H-AGVEGLCSALF-OH. Gradient: 5-95% ACN/H_2_O with 0.1% TFA over 10 min at a flow rate of 0.3 mL/min. (b) UV trace of purified H-AGVEGLCSALF-OH. Gradient: 5-95% ACN/H_2_O with 0.1% TFA over 10 min at a flow rate of 0.3 mL/min. (c) Corresponding MS of purified H-AGVEGLCSALF-OH. ESI-MS calcd.for C_47_H_75_N_11_O_15_S [M+H]^+^ m/z = 1066.52, found 1065.98.

10.15 H-LEAGY-CO-TSAL+ H-CSALF-OH

The ligation between H-LEAGY-CO-TSAL (2.5 mg, 3.7 μmol) and H-CSALF-OH (2.4 mg, 4.5 μmol) was performed as described in the **General procedure for TSAL ester-Cys/Penicillamine ligation**. Purification via preparative HPLC (20-70% ACN/H_2_O over 40 min, 0.1% TFA) followed by lyophilization afforded the N,S-benzylidene acetal intermediate (2.7 mg, 61% yield) as white solids. And the intermediate (1.8 mg) was then treated with TFA/EDT/TMSOTf (90/5/5, v/v/v) for 4 h and carried out as the **General procedure for Acidolysis**. Purification via preparative HPLC (20-70% ACN/H_2_O over 40 min, 0.1%TFA) followed by lyophilization afforded the H-LEAGYCSALF-OH (1.1 mg, 69% yield) as white solids.


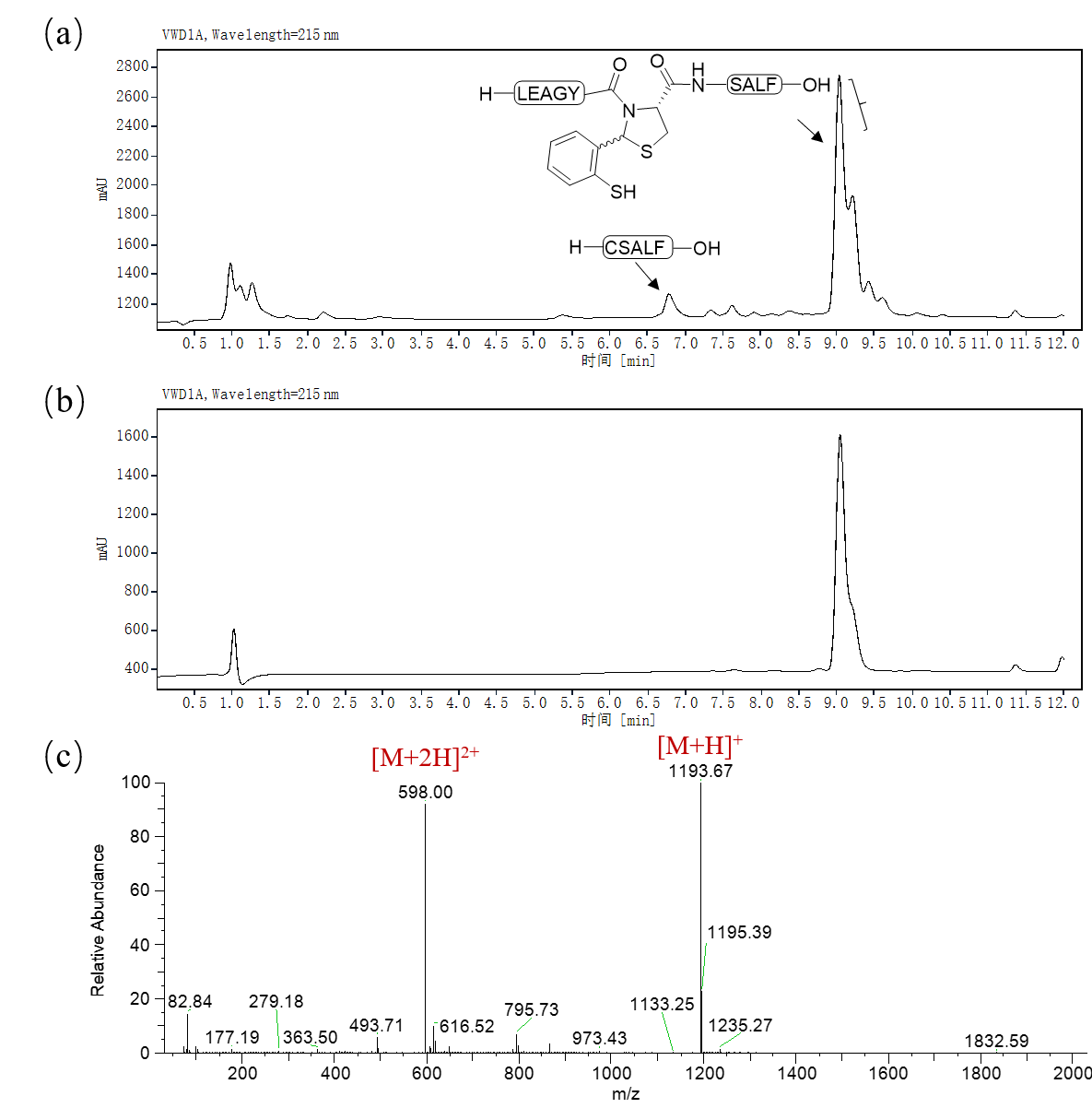


**Figure S86.** (a) Crude HPLC trace for the TSAL ester-Cys ligation between H-LEAGY-CO-TSAL and H-CSALF-OH. Gradient: 20-70% ACN/H_2_O with 0.1% TFA over 12 min at a flow rate of 0.3 mL/min. (b) UV trace of the purified N,S-benzylidene acetal intermediate. Gradient: 20-70% ACN/H_2_O with 0.1% TFA over 12 min at a flow rate of 0.3 mL/min. (c) Corresponding MS of the purified N,S-benzylidene acetal intermediate. ESI-MS calcd.for C_56_H_76_N_10_O_15_S_2_ [M+H]^+^ m/z = 1193.49, found 1193.67; [M+2H]^2+^ m/z = 597.25, found 598.00.


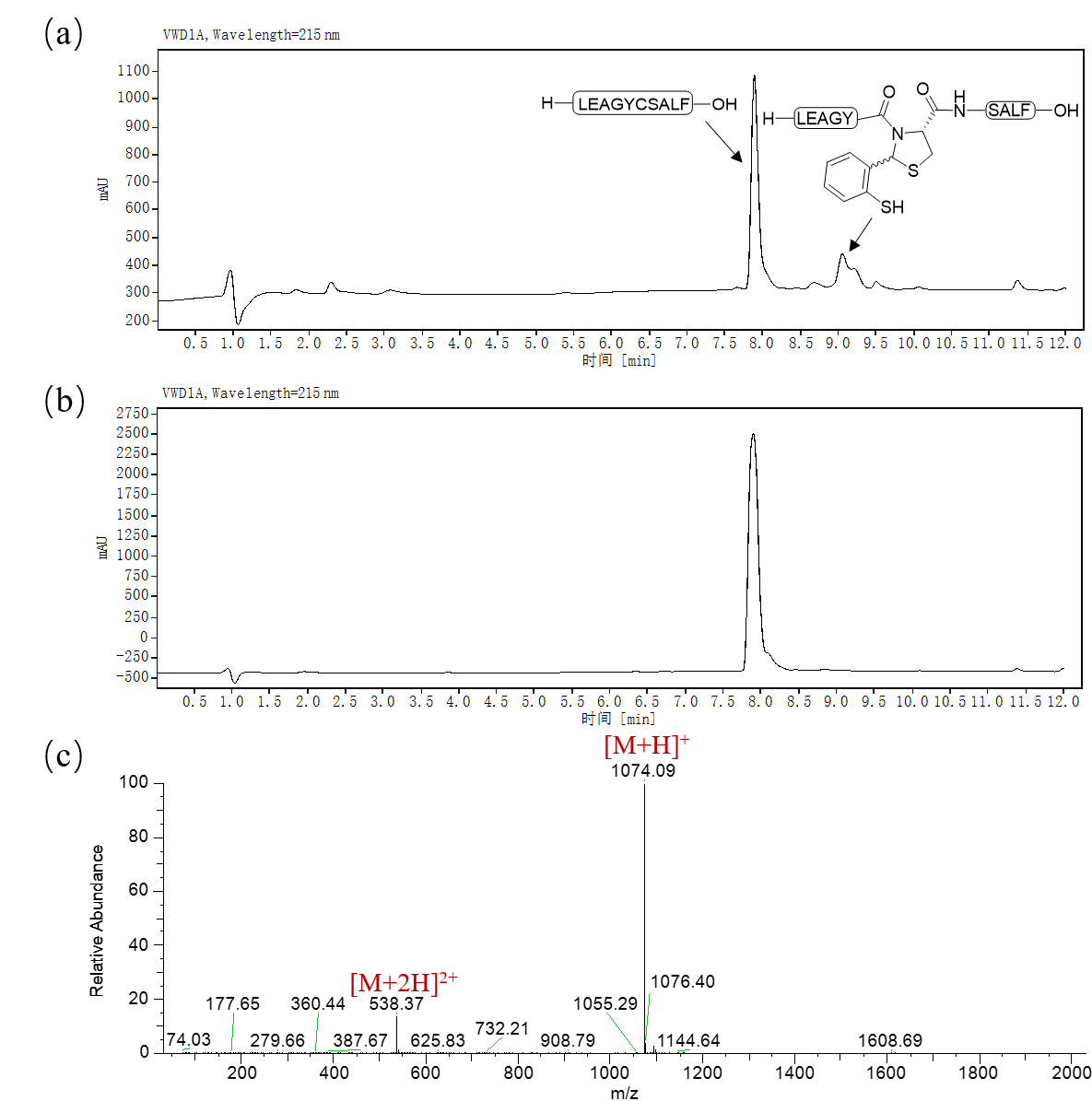


**Figure S88.** (a) Crude HPLC trace for acidolysis of the N,S-benzylidene acetal intermediate to generate H-LEAGYCSALF-OH. Gradient: 20-70% ACN/H_2_O with 0.1% TFA over 12 min at a flow rate of 0.3 mL/min. (b) UV trace of purified H-LEAGYCSALF-OH. Gradient: 20-70% ACN/H_2_O with 0.1% TFA over 12 min at a flow rate of 0.3 mL/min. (c) Corresponding MS of purified H-LEAGYCSALF-OH. ESI-MS calcd.for C_46_H_75_N_13_O_14_S [M+H]^+^ m/z = 1073.49, found 1074.09; [M+2H]^2+^ m/z = 537.25, found 538.37.

**11. TSAL ester-Penicillamine ligation of model peptides**

11.1 H-LARYA-CO-TSAL + H-PenSALF-OH

The ligation between H-LARYA-CO-TSAL (3.2 mg, 4.5 μmol) and H-PenSALF-OH (3.0 mg, 5.3 μmol) was performed as described in the **General procedure for TSAL ester-Cys/Penicillamine ligation**. Purification via preparative HPLC (20-90% ACN/H_2_O over 40 min, 0.1% TFA) followed by lyophilization afforded the N,S-benzylidene acetal intermediate (3.2 mg, 58% isolated yield) as white solids. And the intermediate (2.0 mg) was then treated with TFA/EDT/TMSOTf (90/5/5, v/v/v) for 24 h and carried out as the **General procedure for Acidolysis**. Purification via preparative HPLC (20-90% ACN/H_2_O over 40 min, 0.1%TFA) followed by lyophilization afforded the H-LARYAPenSALF-OH (1.4 mg, 78% yield) as white solids.


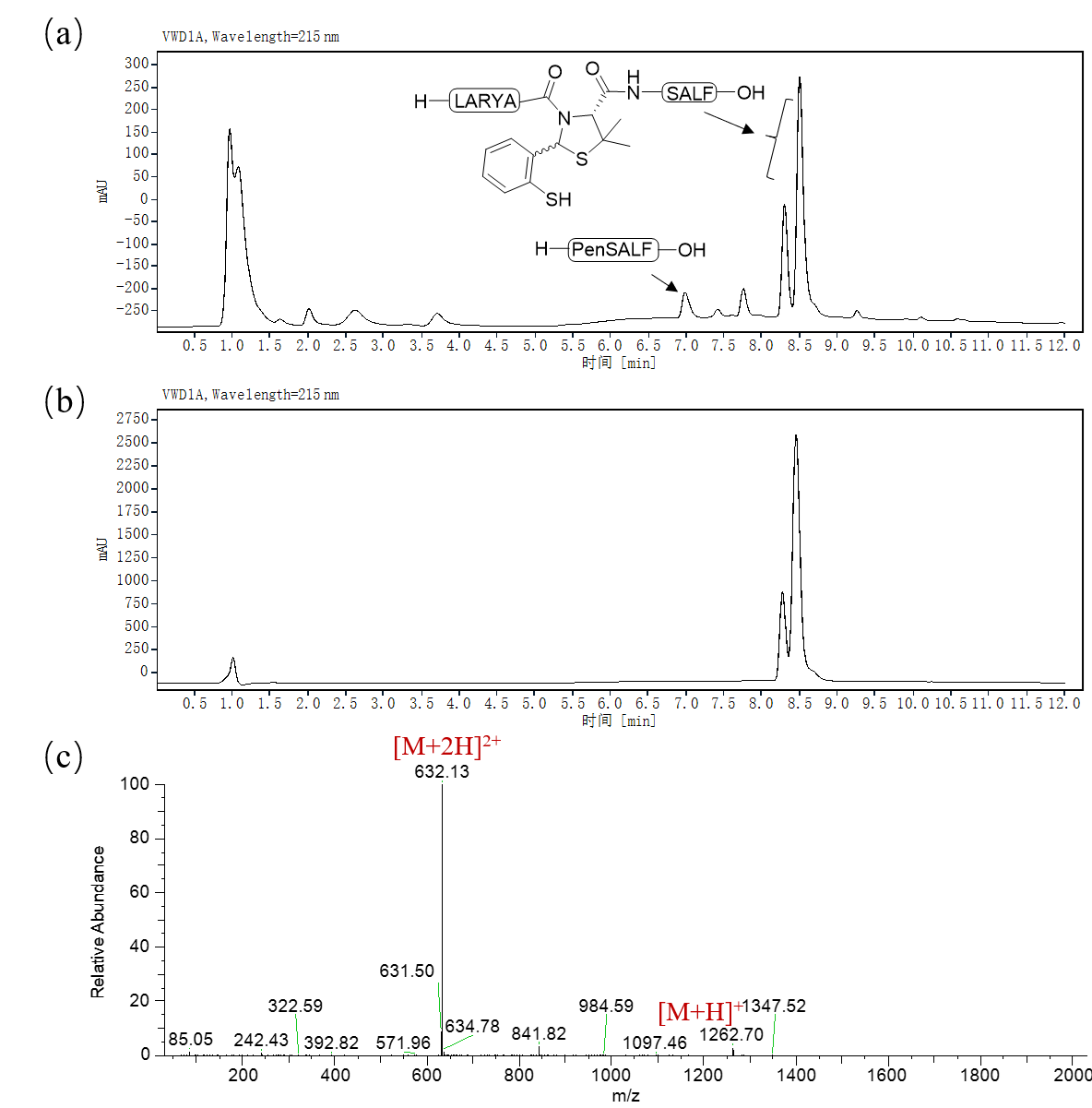


**Figure S89.** (a) Crude HPLC trace for the TSAL ester- Penicillamine ligation between H-LARYA-CO-TSAL and H-PenSALF-OH. Gradient: 20-90% ACN/H_2_O with 0.1% TFA over 12 min at a flow rate of 0.3 mL/min. (b) UV trace of the purified N,S-benzylidene acetal intermediate. Gradient: 20-90% ACN/H_2_O with 0.1% TFA over 12 min at a flow rate of 0.3 mL/min. (c) Corresponding MS of the purified N,S-benzylidene acetal intermediate. ESI-MS calcd.for C_60_H_87_N_13_O_13_S_2_ [M+H]^+^ m/z = 1262.60, found 1262.70; [M+2H]^2+^ m/z = 631.80, found 632.13.


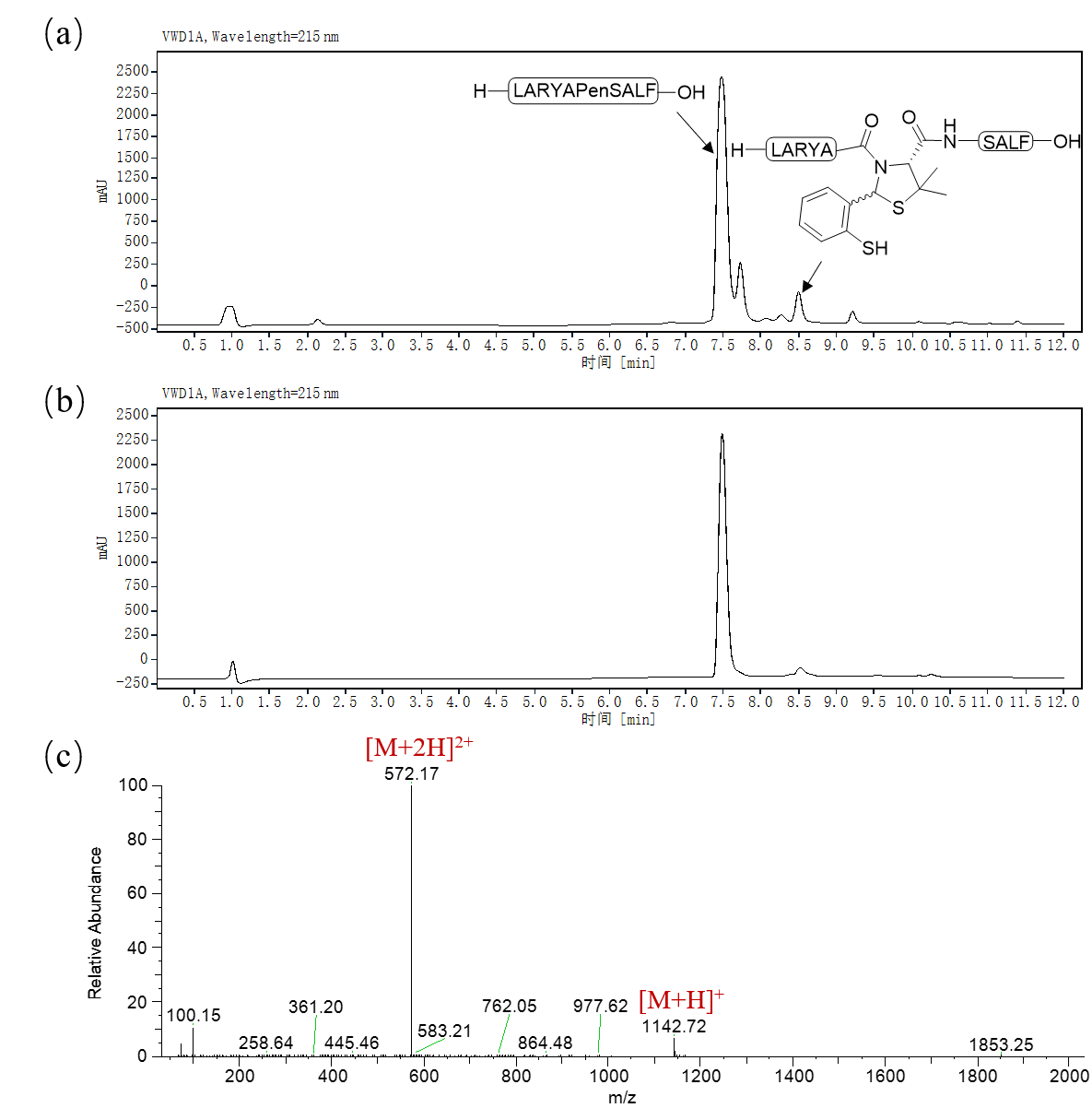


**Figure S90.** (a) Crude HPLC trace for acidolysis of the N,S-benzylidene acetal intermediate to generate H-LARYAPenSALF-OH. Gradient: 20-90% ACN/H_2_O with 0.1% TFA over 12 min at a flow rate of 0.3 mL/min. (b) UV trace of purified H-LARYAPenSALF-OH. Gradient: 20-90% ACN/H_2_O with 0.1% TFA over 12 min at a flow rate of 0.3 mL/min. (c) Corresponding MS of purified H-LARYAPenSALF-OH. ESI-MS calcd.for C_53_H_83_N_13_O_13_S [M+H]^+^ m/z = 1142.60, found 1142.72; [M+2H]^2+^ m/z = 571.80, found 572.17.

11.2 H-LARYK-CO-TSAL + H-PenSALF-OH

The ligation between H-LARYK-CO-TSAL (3.7 mg, 4.8 μmol) and H-PenSALF-OH (3.3 mg, 5.8 μmol) was performed as described in the **General procedure for TSAL ester-Cys/Penicillamine ligation**. Purification via preparative HPLC (20-90% ACN/H_2_O over 40 min, 0.1% TFA) followed by lyophilization afforded the N,S-benzylidene acetal intermediate (1.9 mg, 58% isolated yield) as white solids. And the intermediate (2.3 mg) was then treated with TFA/EDT/TMSOTf (90/5/5, v/v/v) for 24 h and carried out as the **General procedure for Acidolysis**. Purification via preparative HPLC (20-90% ACN/H_2_O over 40 min, 0.1%TFA) followed by lyophilization afforded the H-LARYKPenSALF-OH (1.7 mg, 81% yield) as white solids.


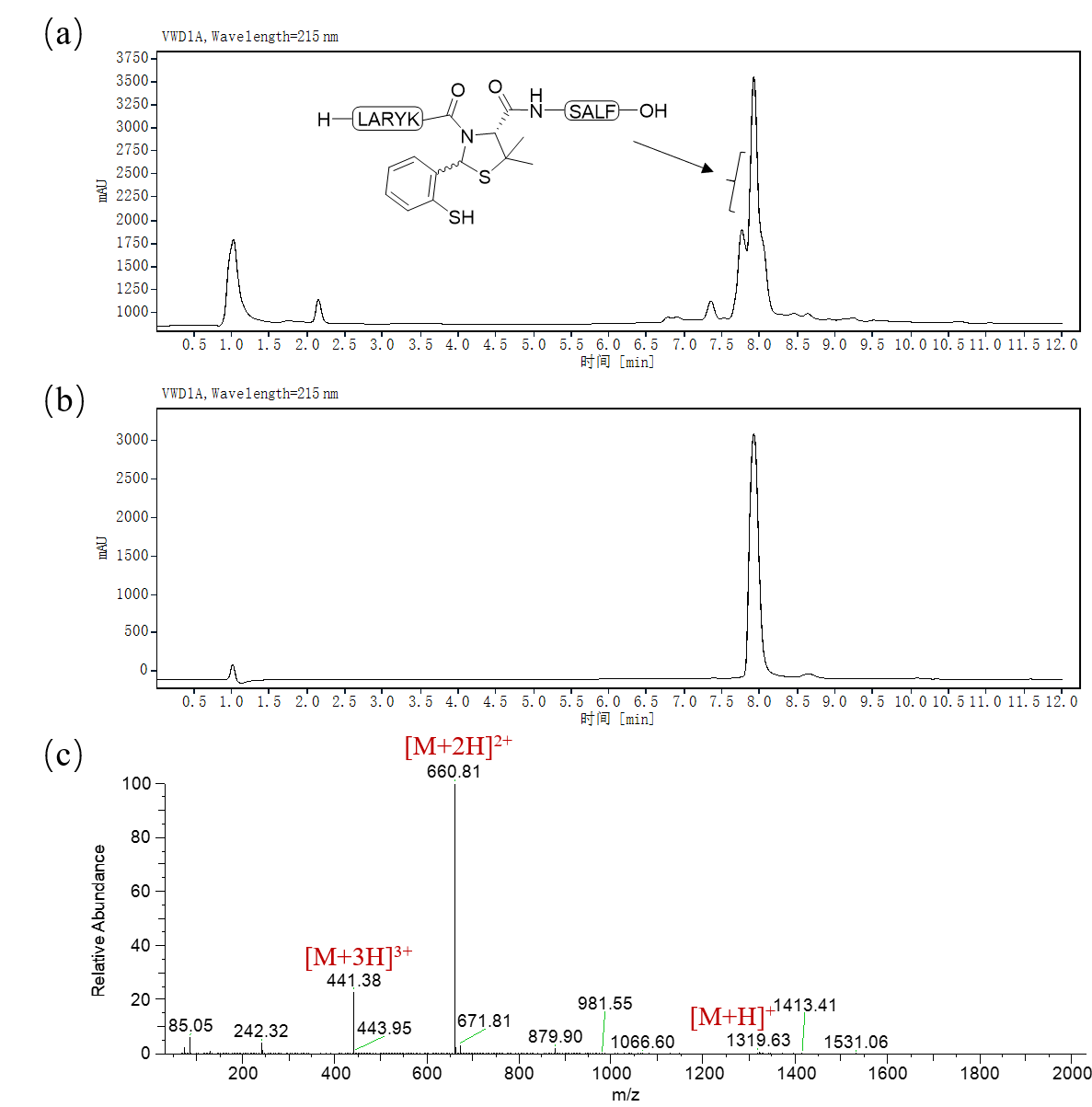


**Figure S91.** (a) Crude HPLC trace for the TSAL ester- Penicillamine ligation between H-LARYK-CO-TSAL and H-PenSALF-OH. Gradient: 20-90% ACN/H_2_O with 0.1% TFA over 12 min at a flow rate of 0.3 mL/min. (b) UV trace of the purified N,S-benzylidene acetal intermediate. Gradient: 20-90% ACN/H_2_O with 0.1% TFA over 12 min at a flow rate of 0.3 mL/min. (c) Corresponding MS of the purified N,S-benzylidene acetal intermediate. ESI-MS calcd.for C_63_H_94_N_14_O_13_S_2_ [M+H]^+^ m/z = 1319.66, found 1319.63; [M+2H]^2+^ m/z = 660.33, found 660.81; [M+3H]^3+^ m/z = 440.55, found 441.38.


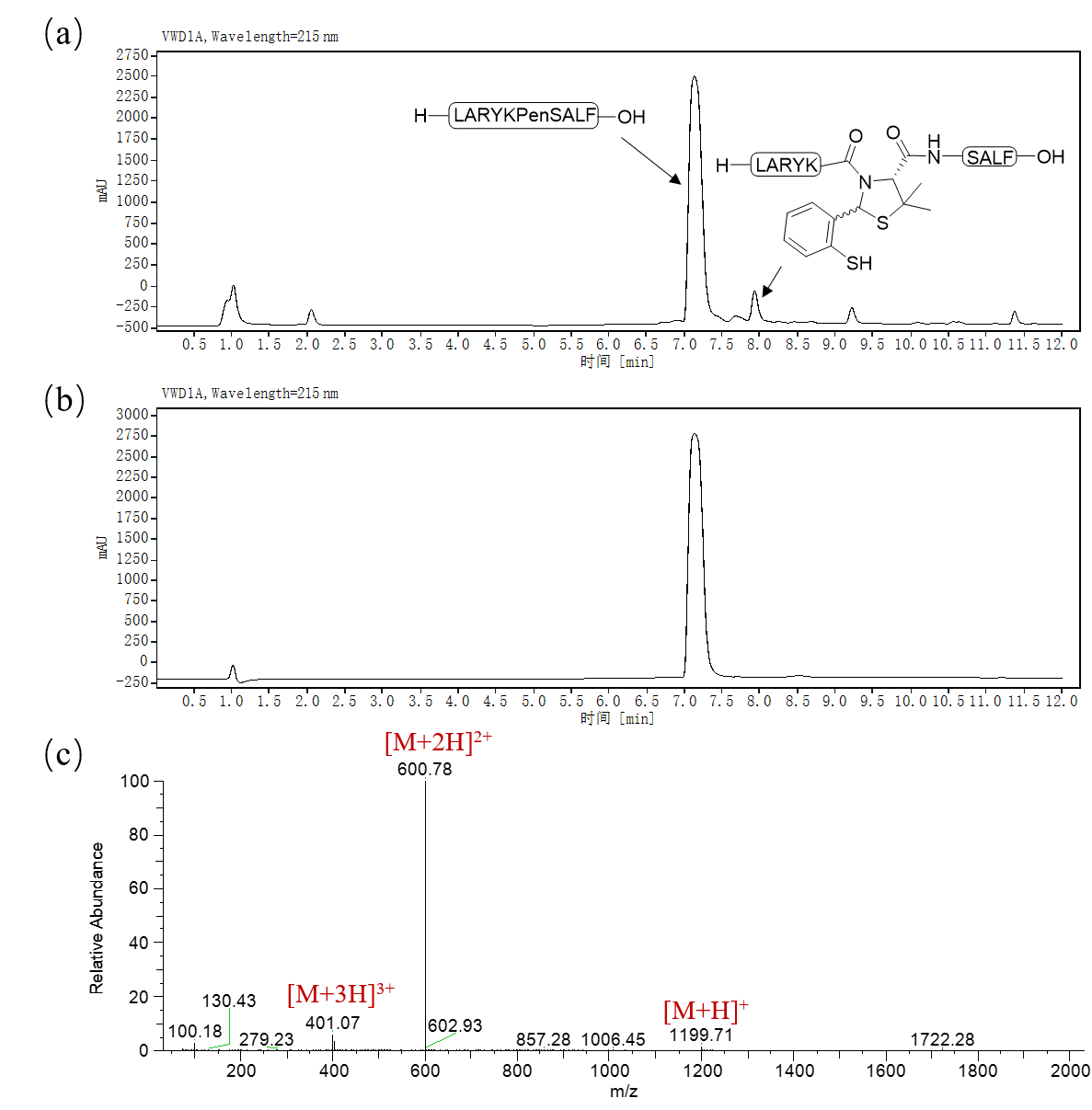


**Figure S92.** (a) Crude HPLC trace for acidolysis of the N,S-benzylidene acetal intermediate to generate H-LARYKPenSALF-OH. Gradient: 20-90% ACN/H_2_O with 0.1% TFA over 12 min at a flow rate of 0.3 mL/min. (b) UV trace of purified H-LARYKPenSALF-OH. Gradient: 20-90% ACN/H_2_O with 0.1% TFA over 12 min at a flow rate of 0.3 mL/min. (c) Corresponding MS of purified H-LARYKPenSALF-OH. ESI-MS calcd.for C_56_H_90_N_14_O_13_S [M+H]^+^ m/z = 1199.65, found 1199.71; [M+2H]^2+^ m/z = 600.33, found 600.78; [M+3H]^3+^ m/z = 400.55, found 401.07.

11.3 H-LARYM-CO-TSAL + H-PenSALF-OH

The ligation between H-LARYM-CO-TSAL (6.9 mg, 8.9 μmol) and H-PenSALF-OH (6.1 mg, 10.8 μmol) was performed as described in the **General procedure for TSAL ester-Cys/Penicillamine ligation**. Purification via preparative HPLC (20-90% ACN/H_2_O over 40 min, 0.1% TFA) followed by lyophilization afforded the N,S-benzylidene acetal intermediate (5.6 mg, 57% isolated yield) as white solids. And the intermediate (4.8 mg) was then treated with TFA/EDT/TMSOTf (90/5/5, v/v/v) for 24 h and carried out as the **General procedure for Acidolysis**. Purification via preparative HPLC (20-90% ACN/H_2_O over 40 min, 0.1%TFA) followed by lyophilization afforded the H-LARYMPenSALF-OH (3.2 mg, 73% yield) as white solids.


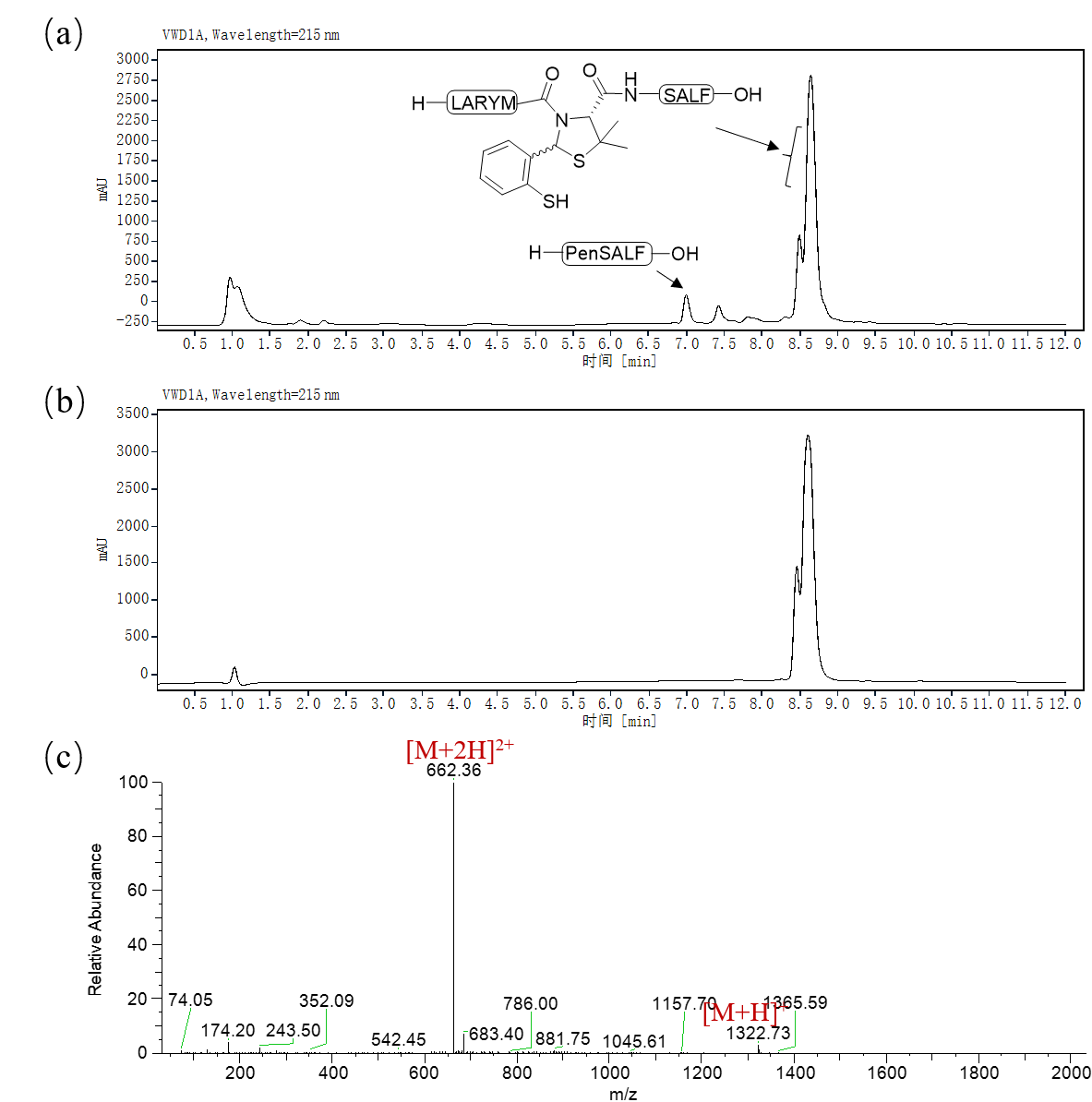


**Figure S93.** (a) Crude HPLC trace for the TSAL ester- Penicillamine ligation between H-LARYM-CO-TSAL and H-PenSALF-OH. Gradient: 20-90% ACN/H_2_O with 0.1% TFA over 12 min at a flow rate of 0.3 mL/min. (b) UV trace of the purified N,S-benzylidene acetal intermediate. Gradient: 20-90% ACN/H_2_O with 0.1% TFA over 12 min at a flow rate of 0.3 mL/min. (c) Corresponding MS of the purified N,S-benzylidene acetal intermediate. ESI-MS calcd.for C_62_H_91_N_13_O_13_S_3_ [M+H]^+^ m/z = 1322.60, found 1322.73; [M+2H]^2+^ m/z = 661.80, found 662.36.


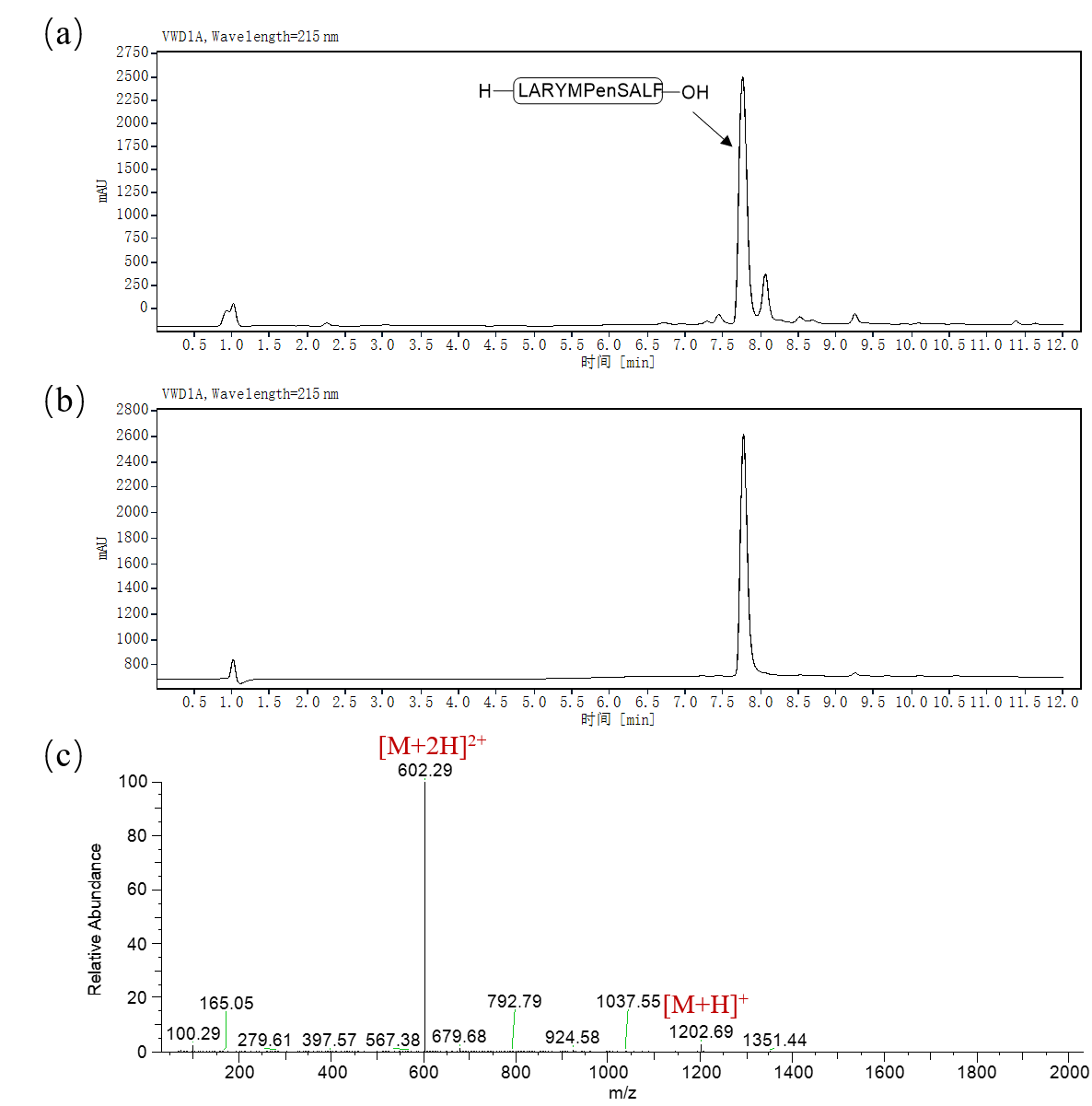


**Figure S94.** (a) Crude HPLC trace for acidolysis of the N,S-benzylidene acetal intermediate to generate H-LARYMPenSALF-OH. Gradient: 20-90% ACN/H_2_O with 0.1% TFA over 12 min at a flow rate of 0.3 mL/min. (b) UV trace of purified H-LARYMPenSALF-OH. Gradient: 20-90% ACN/H_2_O with 0.1% TFA over 12 min at a flow rate of 0.3 mL/min. (c) Corresponding MS of purified H-LARYMPenSALF-OH. ESI-MS calcd.for C_55_H_87_N_13_O_13_S_2_ [M+H]^+^ m/z = 1202.60, found 1202.69; [M+2H]^2+^ m/z = 601.80, found 602.29.

11.4 H-LEAGR-CO-TSAL + H-PenSALF-OH

The ligation between H-LEAGR-CO-TSAL (6.4 mg, 9.6 μmol) and H-PenSALF-OH (6.4 mg, 11.3 μmol) was performed as described in the **General procedure for TSAL ester-Cys/Penicillamine ligation**. Purification via preparative HPLC (20-90% ACN/H_2_O over 40 min, 0.1% TFA) followed by lyophilization afforded the N,S-benzylidene acetal intermediate (5.2 mg, 58% isolated yield) as white solids. And the intermediate (5.2 mg) was then treated with TFA/EDT/TMSOTf (90/5/5, v/v/v) for 24 h and carried out as the **General procedure for Acidolysis**. Purification via preparative HPLC (20-90% ACN/H_2_O over 40 min, 0.1%TFA) followed by lyophilization afforded the H-LEAGRPenSALF-OH (3.1 mg, 66% yield) as white solids.


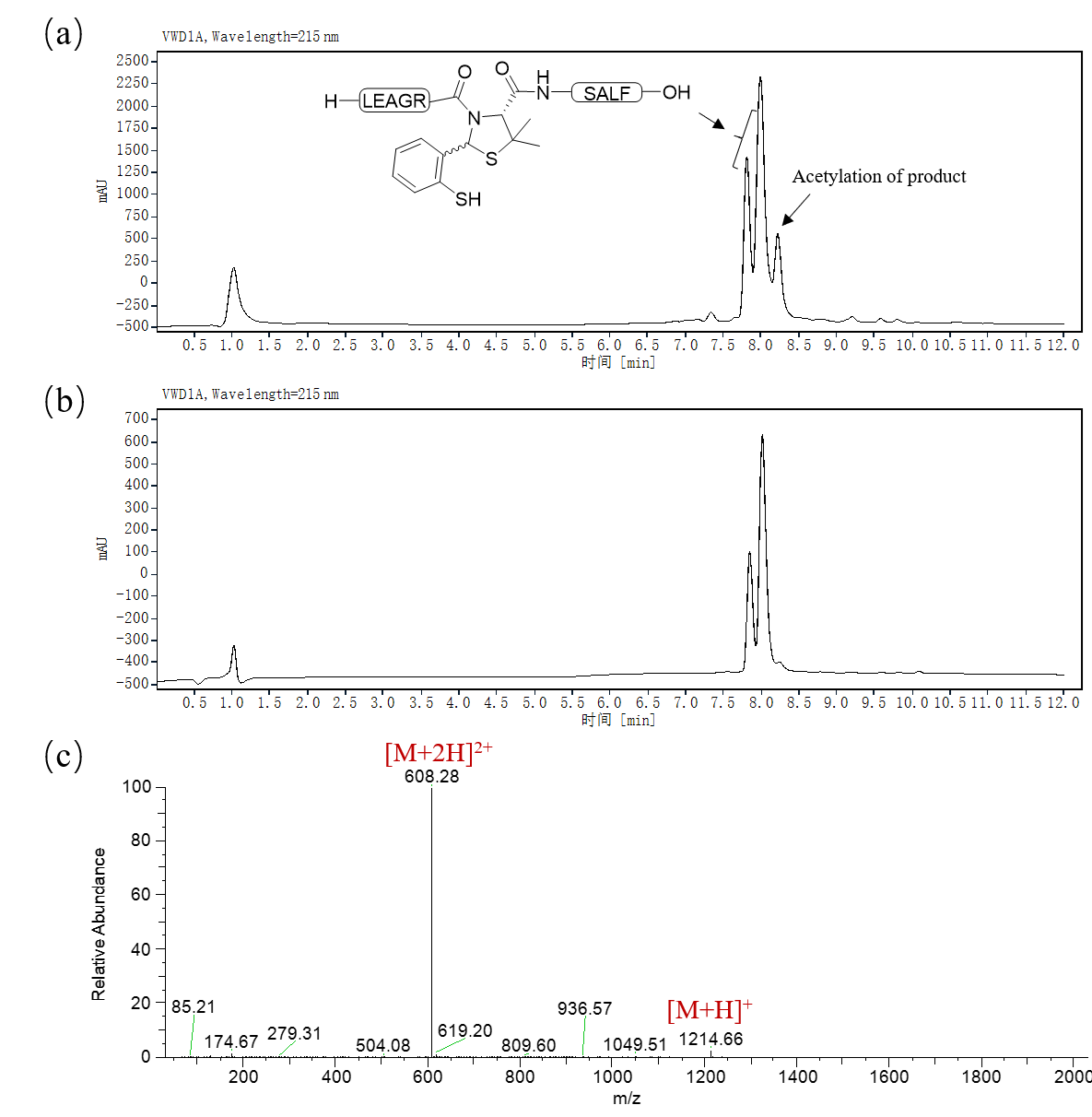


**Figure S95.** (a) Crude HPLC trace for the TSAL ester- Penicillamine ligation between H-LEAGR-CO-TSAL and H-PenSALF-OH. Gradient: 20-90% ACN/H_2_O with 0.1% TFA over 12 min at a flow rate of 0.3 mL/min. (b) UV trace of the purified N,S-benzylidene acetal intermediate. Gradient: 20-90% ACN/H_2_O with 0.1% TFA over 12 min at a flow rate of 0.3 mL/min. (c) Corresponding MS of the purified N,S-benzylidene acetal intermediate. ESI-MS calcd.for C_55_H_83_N_13_O_14_S_2_ [M+H]^+^ m/z = 1214.56, found 1214.66; [M+2H]^2+^ m/z = 607.78, found 608.28.


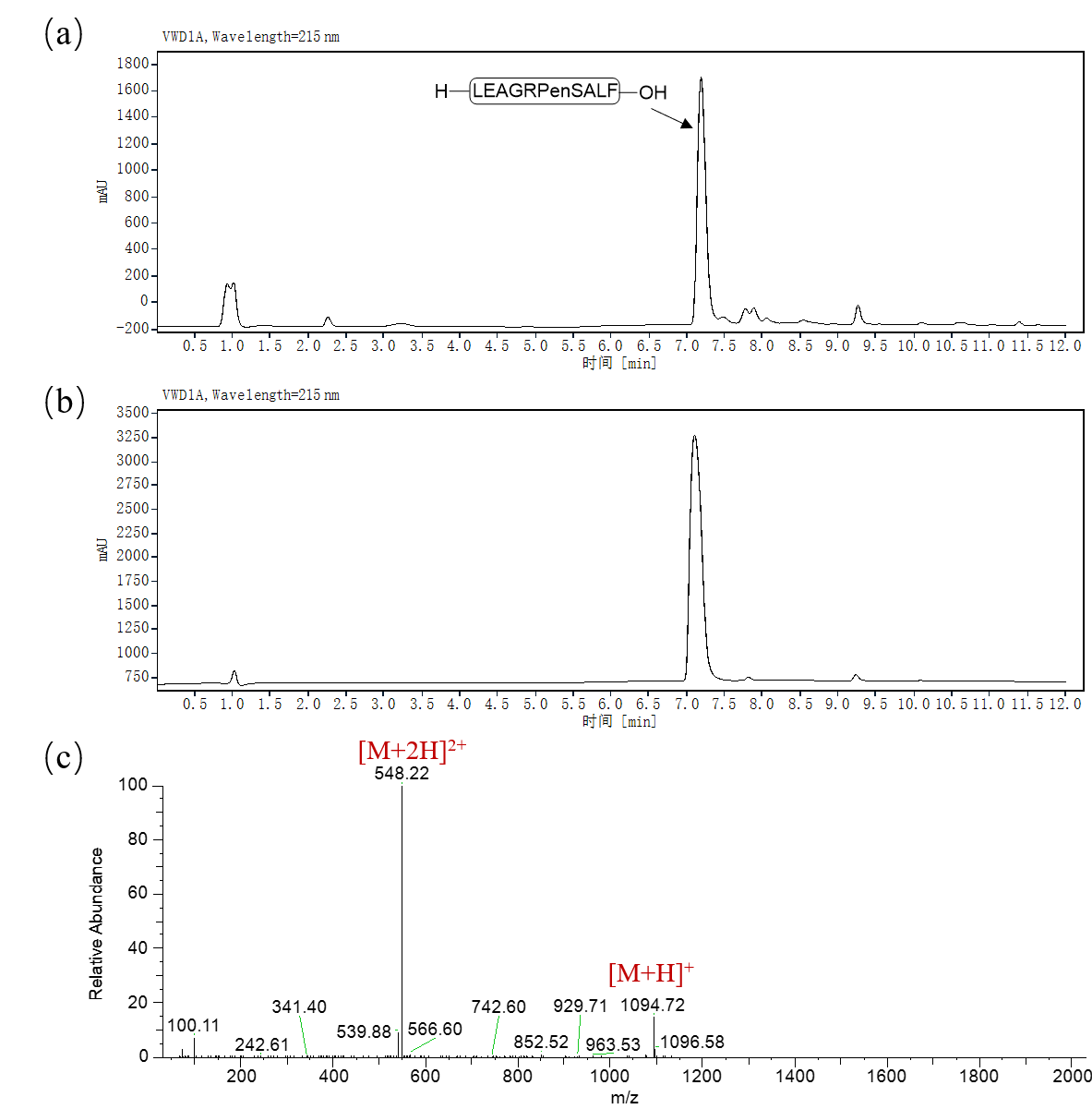


**Figure S96.** (a) Crude HPLC trace for acidolysis of the N,S-benzylidene acetal intermediate to generate H-LEAGRPenSALF-OH. Gradient: 20-90% ACN/H_2_O with 0.1% TFA over 12 min at a flow rate of 0.3 mL/min. (b) UV trace of purified H-LEAGRPenSALF-OH. Gradient: 20-90% ACN/H_2_O with 0.1% TFA over 12 min at a flow rate of 0.3 mL/min. (c) Corresponding MS of purified H-LEAGRPenSALF-OH. ESI-MS calcd.for C_48_H_79_N_13_O_14_S [M+H]^+^ m/z = 1094.56, found 1094.72; [M+2H]^2+^ m/z = 547.78, found 548.22.

11.5 H-LARYS-CO-TSAL + H-PenSALF-OH

The ligation between H-LARYS-CO-TSAL (9.9 mg, 13.6 μmol) and H-PenSALF-OH (8.3 mg, 14.6 μmol) was performed as described in the **General procedure for TSAL ester-Cys/Penicillamine ligation**. Purification via preparative HPLC (20-90% ACN/H_2_O over 40 min, 0.1% TFA) followed by lyophilization afforded the N,S-benzylidene acetal intermediate (4.7 mg, 47% isolated yield) as white solids. And the intermediate (4.7 mg) was then treated with TFA/EDT/TMSOTf (90/5/5, v/v/v) for 24 h and carried out as the **General procedure for Acidolysis**. Purification via preparative HPLC (20-90% ACN/H_2_O over 40 min, 0.1%TFA) followed by lyophilization afforded the H-LARYSPenSALF-OH (2.1 mg, 50% yield) as white solids.


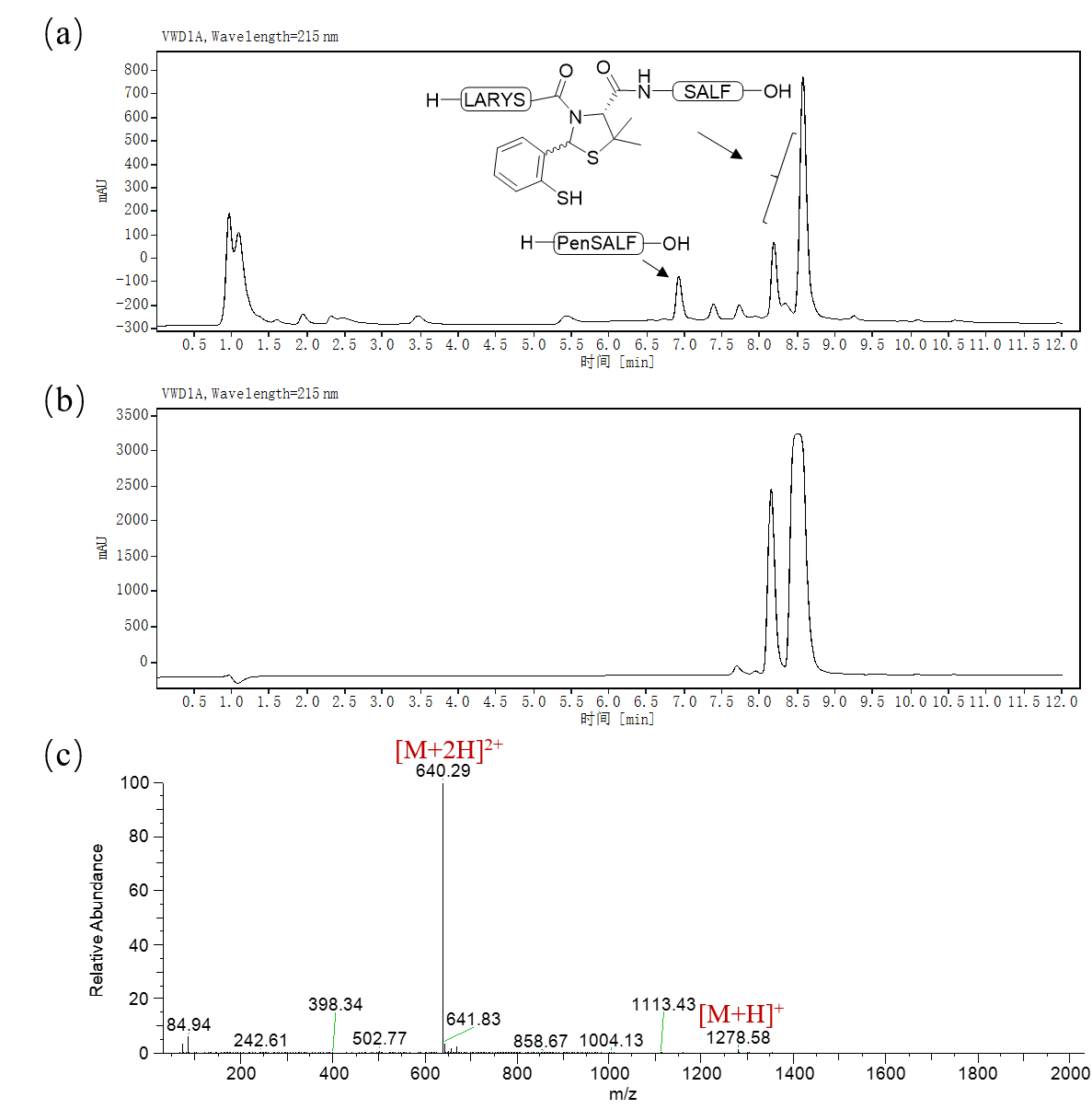


**Figure S97.** (a) Crude HPLC trace for the TSAL ester- Penicillamine ligation between H-LARYS-CO-TSAL and H-PenSALF-OH. Gradient: 20-90% ACN/H_2_O with 0.1% TFA over 12 min at a flow rate of 0.3 mL/min. (b) UV trace of the purified N,S-benzylidene acetal intermediate. Gradient: 20-90% ACN/H_2_O with 0.1% TFA over 12 min at a flow rate of 0.3 mL/min. (c) Corresponding MS of the purified N,S-benzylidene acetal intermediate. ESI-MS calcd.for C_60_H_87_N_13_O_14_S_2_ [M+H]^+^ m/z = 1278.59, found 1278.58; [M+2H]^2+^ m/z = 639.80, found 640.29.


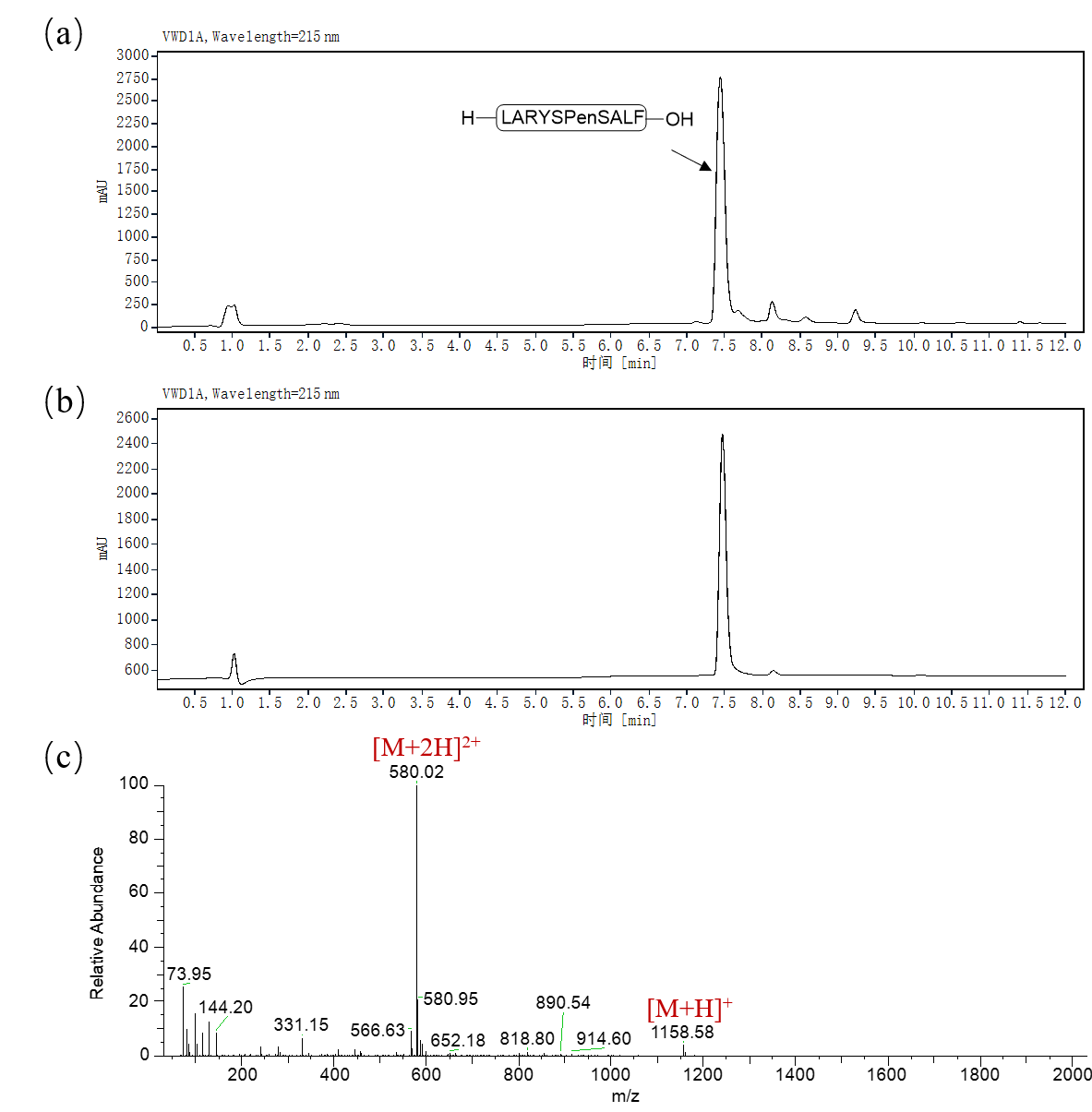


**Figure S98.** (a) Crude HPLC trace for acidolysis of the N,S-benzylidene acetal intermediate to generate H-LARYSPenSALF-OH. Gradient: 20-90% ACN/H_2_O with 0.1% TFA over 12 min at a flow rate of 0.3 mL/min. (b) UV trace of purified H-LARYSPenSALF-OH. Gradient: 20-90% ACN/H_2_O with 0.1% TFA over 12 min at a flow rate of 0.3 mL/min. (c) Corresponding MS of purified H-LARYSPenSALF-OH. ESI-MS calcd.for C_53_H_83_N_13_O_14_S [M+H]^+^ m/z = 1158.59, found 1158.58; [M+2H]^2+^ m/z = 579.80, found 580.02.

11.6 H-LARYT-CO-TSAL + H-PenSALF-OH

The ligation between H-LARYT-CO-TSAL (8.9 mg, 12.0 μmol) and H-PenSALF-OH (8.3 mg, 14.6 μmol) was performed as described in the **General procedure for TSAL ester-Cys/Penicillamine ligation**. Purification via preparative HPLC (20-90% ACN/H_2_O over 40 min, 0.1% TFA) followed by lyophilization afforded the N,S-benzylidene acetal intermediate (7.2 mg, 67% isolated yield) as white solids. And the intermediate (6.1 mg) was then treated with TFA/EDT/TMSOTf (90/5/5, v/v/v) for 24 h and carried out as the **General procedure for Acidolysis**. Purification via preparative HPLC (20-90% ACN/H_2_O over 40 min, 0.1%TFA) followed by lyophilization afforded the H-LARYTPenSALF-OH (3.7 mg, 67% yield) as white solids.


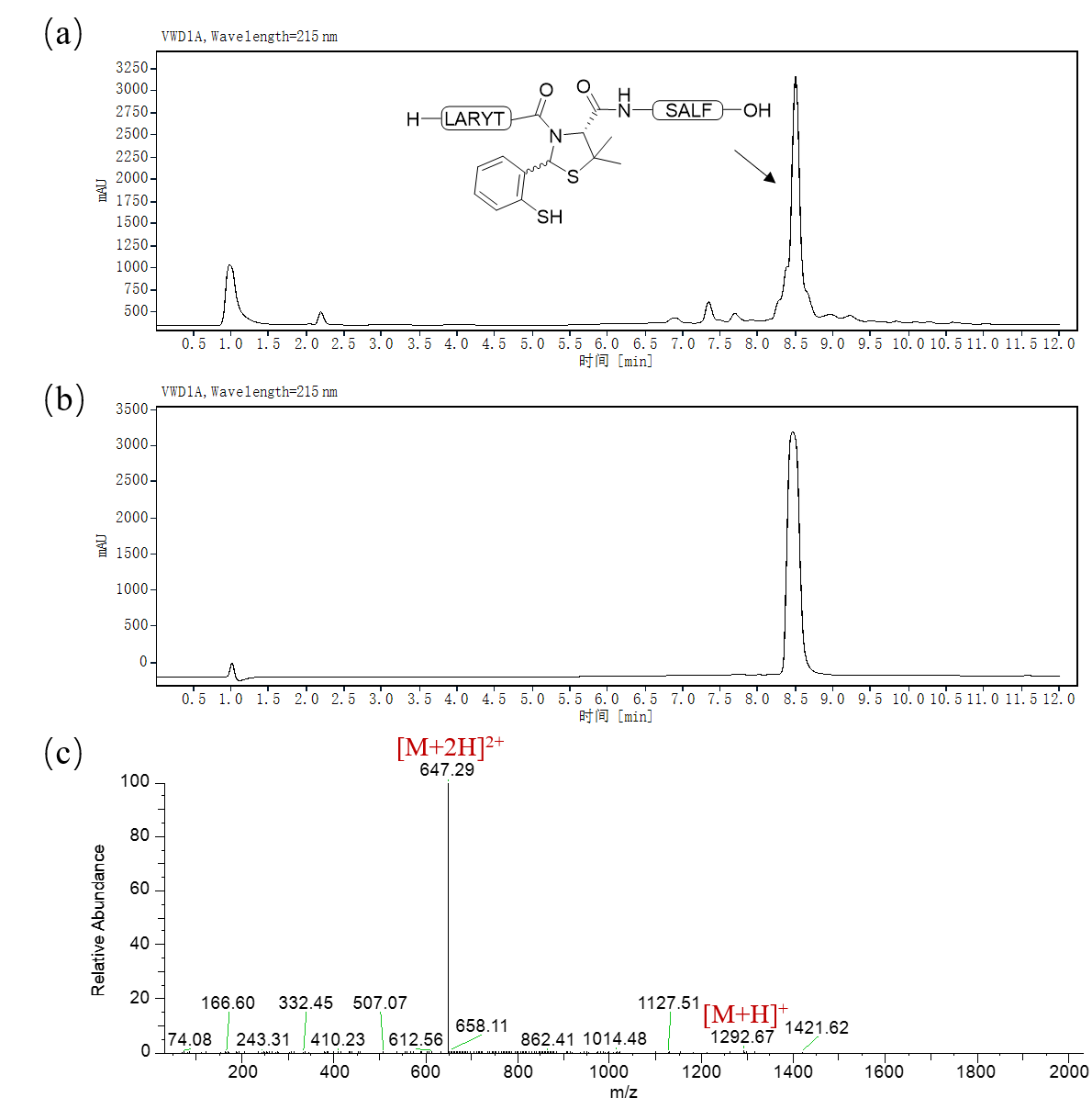


**Figure S99.** (a) Crude HPLC trace for the TSAL ester- Penicillamine ligation between H-LARYT-CO-TSAL and H-PenSALF-OH. Gradient: 20-90% ACN/H_2_O with 0.1% TFA over 12 min at a flow rate of 0.3 mL/min. (b) UV trace of the purified N,S-benzylidene acetal intermediate. Gradient: 20-90% ACN/H_2_O with 0.1% TFA over 12 min at a flow rate of 0.3 mL/min. (c) Corresponding MS of the purified N,S-benzylidene acetal intermediate. ESI-MS calcd.for C_61_H_89_N_13_O_14_S_2_ [M+H]^+^ m/z = 1292.61, found 1292.67; [M+2H]^2+^ m/z = 646.81, found 647.29.

**Figure S100.** (a) Crude HPLC trace for acidolysis of the N,S-benzylidene acetal intermediate to generate H-LARYTPenSALF-OH. Gradient: 20-90% ACN/H_2_O with 0.1% TFA over 12 min at a flow rate of 0.3 mL/min. (b) UV trace of purified H-LARYTPenSALF-OH. Gradient: 20-90% ACN/H_2_O with 0.1% TFA over 12 min at a flow rate of 0.3 mL/min. (c) Corresponding MS of purified H-LARYTPenSALF-OH. ESI-MS calcd.for C_54_H_85_N_13_O_14_S [M+H]^+^ m/z = 1172.61, found 1172.80; [M+2H]^2+^ m/z = 586.81, found 587.24.

11.7 H-LARYP-CO-TSAL + H-PenSALF-OH

The ligation between H-LARYP-CO-TSAL (10.3 mg, 14.0 μmol) and H-PenSALF-OH (8.0 mg, 14.0 μmol) was performed as described in the **General procedure for TSAL ester-Cys/Penicillamine ligation**. Purification via preparative HPLC (20-90% ACN/H_2_O over 40 min, 0.1% TFA) followed by lyophilization afforded the N,S-benzylidene acetal intermediate (11.7 mg, 65% isolated yield) as white solids. And the intermediate (2.5 mg) was then treated with TFA/EDT/TMSOTf (90/5/5, v/v/v) for 24 h and carried out as the **General procedure for Acidolysis**. Purification via preparative HPLC (20-90% ACN/H_2_O over 40 min, 0.1%TFA) followed by lyophilization afforded the H-LARYPPenSALF-OH (1.0 mg, 44% yield) as white solids.

**Figure S101.** (a) Crude HPLC trace for the TSAL ester- Penicillamine ligation between H-LARYP-CO-TSAL and H-PenSALF-OH. Gradient: 5-95% ACN/H_2_O with 0.1% TFA over 10 min at a flow rate of 0.3 mL/min. (b) UV trace of the purified N,S-benzylidene acetal intermediate. Gradient: 10-80% ACN/H_2_O with 0.1% TFA over 10 min at a flow rate of 0.3 mL/min. (c) Corresponding MS of the purified N,S-benzylidene acetal intermediate. ESI-MS calcd.for C_62_H_89_N_13_O_13_S_2_ [M+2H]^2+^ m/z = 644.81, found 645.22.

**Figure S102.** (a) Crude HPLC trace for acidolysis of the N,S-benzylidene acetal intermediate to generate H-LARYPPenSALF-OH. Gradient: 5-95% ACN/H_2_O with 0.1% TFA over 7 min at a flow rate of 0.3 mL/min. (b) UV trace of purified H-LARYPPenSALF-OH. Gradient: 5-95% ACN/H_2_O with 0.1% TFA over 10 min at a flow rate of 0.3 mL/min. (c) Corresponding MS of purified H-LARYPPenSALF-OH. ESI-MS calcd.for C_55_H_85_N_13_O_13_S [M+H]^+^ m/z = 1168.61, found 1168.71; [M+2H]^2+^ m/z = 584.81, found 585.21.

11.8 H-LARYV-CO-TSAL + H-PenSALF-OH

The ligation between H-LARYV-CO-TSAL (2.0 mg, 2.7 μmol) and H-PenSALF-OH (2.3 mg, 4.1 μmol) was performed as described in the **General procedure for TSAL ester-Cys/Penicillamine ligation**. Purification via preparative HPLC (20-90% ACN/H_2_O over 40 min, 0.1% TFA) followed by lyophilization afforded the N,S-benzylidene acetal intermediate (2.2 mg, 63% isolated yield) as white solids. And the intermediate (2.2 mg) was then treated with TFA/EDT/TMSOTf (90/5/5, v/v/v) for 24 h and carried out as the **General procedure for Acidolysis**. Purification via preparative HPLC (20-90% ACN/H_2_O over 40 min, 0.1%TFA) followed by lyophilization afforded the H-LARYVPenSALF-OH (1.3 mg, 65% yield) as white solids.

**Figure S103.** (a) Crude HPLC trace for the TSAL ester- Penicillamine ligation between H-LARYV-CO-TSAL and H-PenSALF-OH. Gradient: 5-95% ACN/H_2_O with 0.1% TFA over 10 min at a flow rate of 0.3 mL/min. (b) UV trace of the purified N,S-benzylidene acetal intermediate. Gradient: 5-95% ACN/H_2_O with 0.1% TFA over 10 min at a flow rate of 0.3 mL/min. (c) Corresponding MS of the purified N,S-benzylidene acetal intermediate. ESI-MS calcd.for C_124_H_180_N_26_O_26_S_4_ [M+3H]^3+^ m/z = 860.42, found 860.37; [M+4H]^4+^ m/z = 645.56, found 645.90.

**Figure S104.** (a) Crude HPLC trace for acidolysis of the N,S-benzylidene acetal intermediate to generate H-LARYVPenSALF-OH. Gradient: 5-95% ACN/H_2_O with 0.1% TFA over 10 min at a flow rate of 0.3 mL/min. (b) UV trace of purified H-LARYVPenSALF-OH. Gradient: 5-95% ACN/H_2_O with 0.1% TFA over 10 min at a flow rate of 0.3 mL/min. (c) Corresponding MS of purified H-LARYVPenSALF-OH. ESI-MS calcd.for C_55_H_87_N_13_O_13_S [M+H]^+^ m/z = 1170.63, found 1170.68; [M+2H]^2+^ m/z = 585.82, found 586.17.

11.9 Ac-SYSMEHFRWGKP-CO-TSAL + H-PenGKKRRPVKVYP-OH

The ligation between Ac-SYSMEHFRWGKP-CO-TSAL (4.7 mg, 2.8 μmol) and H-PenGKKRRPVKVYP-OH (6.1 mg, 4.2 μmol) was performed as described in the **General procedure for TSAL ester-Cys/Penicillamine ligation**. Purification via preparative HPLC (20-90% ACN/H_2_O over 40 min, 0.1% TFA) followed by lyophilization afforded the N,S-benzylidene acetal intermediate (4.8 mg, 55% isolated yield) as white solids. And the intermediate (1.5 mg) was then treated with TFA/EDT/TMSOTf (90/5/5, v/v/v) for 24 h and carried out as the **General procedure for Acidolysis**. Purification via preparative HPLC (20-90% ACN/H_2_O over 40 min, 0.1%TFA) followed by lyophilization afforded the Ac-SYSMEHFRWGKPPenGKKRRPVKVYP-OH (0.8 mg, 55% yield) as white solids. Finally, the product of acidolysis was carried out as the **General procedure for Desulfurization**, Ac-SYSMEHFRWGKPVGKKRRPVKVYP-OH (0.6 mg, 76%) was obatined by purification and lyophilization.

**Figure S105.** (a) Crude HPLC trace for the TSAL ester- Penicillamine ligation between Ac-SYSMEHFRWGKP-CO-TSAL and H-PenGKKRRPVKVYP-OH. Gradient: 5-95% ACN/H_2_O with 0.1% TFA over 10 min at a flow rate of 0.3 mL/min. (b) UV trace of the purified N,S-benzylidene acetal intermediate. Gradient: 5-95% ACN/H_2_O with 0.1% TFA over 10 min at a flow rate of 0.3 mL/min. (c) Corresponding MS of the purified N,S-benzylidene acetal intermediate. ESI-MS calcd.for C_145_H_216_N_40_O_32_S_3_ [M+3H]^3+^ m/z = 1043.19, found 1043.59; [M+4H]^4+^ m/z = 782.64, found 783.10; [M+5H]^5+^ m/z = 626.31, found 626.74.

**Figure S106.** (a) Crude HPLC trace for acidolysis of the N,S-benzylidene acetal intermediate to generate Ac-SYSMEHFRWGKPPenGKKRRPVKVYP-OH. Gradient: 5-95% ACN/H_2_O with 0.1% TFA over 10 min at a flow rate of 0.3 mL/min. (b) UV trace of purified Ac-SYSMEHFRWGKPPenGKKRRPVKVYP-OH. Gradient: 5-95% ACN/H_2_O with 0.1% TFA over 10 min at a flow rate of 0.3 mL/min. (c) Corresponding MS of purified Ac-SYSMEHFRWGKPPenGKKRRPVKVYP-OH. ESI-MS calcd.for C_138_H_212_N_40_O_32_S_2_ [M+3H]^3+^ m/z = 1003.19, found 1003.64; [M+4H]^4+^ m/z = 752.64, found 753.11; [M+5H]^5+^ m/z = 602.31, found 602.96.

**Figure S107.** (a) Crude HPLC trace for NaBEt_4_**-**desulfurization of Ac-SYSMEHFRWGKPPenGKKRRPVKVYP-OH. Gradient: 5-95% ACN/H_2_O with 0.1% TFA over 10 min at a flow rate of 0.3 mL/min. (b) UV trace of purified Ac-SYSMEHFRWGKPVGKKRRPVKVYP-OH. Gradient: 5-95% ACN/H_2_O with 0.1% TFA over 10 min at a flow rate of 0.3 mL/min. (c) Corresponding MS of purified Ac-SYSMEHFRWGKPVGKKRRPVKVYP-OH. ESI-MS calcd.for C_138_H_212_N_40_O_32_S_2_ [M+3H]^3+^ m/z = 992.53, found 992.77; [M+4H]^4+^ m/z = 744.65, found 744.92; [M+5H]^5+^ m/z = 595.92, found 596.30.

**12. Synthesis of Ubiquitin (1-76)**

**12.1 Synthesis of 1.**

Ub(1-45)-NHNH_2_ (**1**) was assembled according to general Fmoc-SPPS procedure (0.05 mmol). The crude peptide was purified by preparative HPLC (10-60% ACN/H_2_O over 50 min) and lyophilized to afford the Ub(1-45)-NHNH_2_ (**1**) (70.0 mg, 27% yield) as a white powder.

**Figure S108.** UV trace and corresponding MS from LC-MS analysis of purified Ub(1-45)-NHNH_2_ (**1**). Gradient: 5-95% ACN/H_2_O with 0.1% TFA over 10 min at a flow rate of 0.3 mL/min. ESI-MS calcd.for C_229_H_382_N_60_O_70_S [M+4H]^4+^ m/z = 1282.70, [M+5H]^5+^ m/z = 1026.36, [M+6H]^6+^ m/z = 855.47, [M+7H]^7+^ m/z = 733.40, [M+8H]^8+^ m/z = 641.85, found 1282.93, 1026.69, 856.04, 733.98, 642.41.

**12.2 Synthesis of 1a.**

Ub(1-45)-CO-TSAL^SCA^ (**1a**) was obtained according to **General procedure for synthesis of model C-terminus peptide TSAL^SCA^ -esters** on 25.8 mg Ub(1-45)-NHNH_2_ (**1**). The crude peptide was purified by preparative HPLC (10-60% ACN/H_2_O over 50 min) and lyophilized to afford the Ub(1-45)-CO-TSAL^SCA^ (**1a**) (13.9 mg, 52% yield) as a white powder.

**Figure S109.** UV trace and corresponding MS from LC-MS analysis of purified Ub(1-45)-CO-TSAL^SCA^ (**1a**). Gradient: 5-95% ACN/H_2_O with 0.1% TFA over 10 min at a flow rate of 0.3 mL/min. ESI-MS calcd.for C_237_H_387_N_61_O_71_S_2_ [M+5H]^5+^ m/z = 1058.96, [M+6H]^6+^ m/z = 882.64, [M+7H]^7+^ m/z = 756.69, found 1059.18, 882.85, 756.79.

**12.3 Synthesis of 2.**

Ub(1-45)-CO-TSAL (**2**) was obtained according to **Pyruvic acid treatment of peptide TSAL^SCA^-esters for C-terminus peptide TSAL-esters** on 6.7 mg Ub(1-45)-CO-TSAL^SCA^ (**1a**). The crude peptide was purified by preparative HPLC (10-60% ACN/H_2_O over 50 min) and lyophilized to afford the H- Ub(1-45)-CO-TSAL (**2**) (3.3 mg, 50% yield) as a white powder.

**Figure S110.** UV trace and corresponding MS from LC-MS analysis of purified Ub(1-45)-CO-TSAL (**2**). Gradient: 20-90% ACN/H_2_O with 0.1% TFA over 7 min at a flow rate of 0.3 mL/min. ESI-MS calcd.for C_236_H_384_N_58_O_71_S_2_ [M+5H]^5+^ m/z = 1047.55, [M+6H]^6+^ m/z = 873.13, [M+7H]^7+^ m/z = 748.54, [M+8H]^8+^ m/z = 655.10, found 1047.52, 873.25, 748.53, 655.03.

**12.4 Synthesis of 3.**

Peptide **3** was assembled according to general Fmoc-SPPS procedure (0.025 mmol). The crude peptide was purified by preparative HPLC (10-60% ACN/H_2_O over 30 min) and lyophilized to afford the peptide **3** (27.2 mg, 31% yield) as a white powder.

**Figure S111.** UV trace and corresponding MS from LC-MS analysis of purified peptide **3**. Gradient: 20-90% ACN/H_2_O with 0.1% TFA over 7 min at a flow rate of 0.3 mL/min. ESI-MS calcd.for C_149_H_251_N_47_O_48_S [M+3H]^3+^ m/z = 1167.95, [M+4H]^4+^ m/z = 876.21, [M+5H]^5+^ m/z = 701.17, [M+6H]^6+^ m/z = 584.47, found 1168.05, 876.44, 701.69, 584.88.

**12.5 Synthesis of 4.**

The ligation between peptide **2** (3.3 mg, 0.6 μmol) and **3** (2.5 mg, 0.7 μmol) was performed as described in the **General procedure for TSAL ester-Cys/Penicillamine ligation**. Peptide **2** and **3** were dissolved in 315 μL pyridine/acetic acid (1/6, mol/mol) and reacted for 12 h.Purification via preparative HPLC (20-70% ACN/H_2_O over 40 min, 0.1% TFA) followed by lyophilization afforded the N,S-benzylidene acetal intermediate **4** (2.2 mg, 47% yield) as white solids.

**Figure S112.** Crude HPLC trace for the ligation between peptide **2** and **3**. (a) ligation at t = 0 h, gradient: 20-90% ACN/H_2_O with 0.1% TFA over 7 min at a flow rate of 0.3 mL/min. (b) ligation at t = 4 h, gradient: 20-90% ACN/H_2_O with 0.1% TFA over 7 min at a flow rate of 0.3 mL/min. (c) ligation at t = 12 h, gradient: 20-90% ACN/H_2_O with 0.1% TFA over 7 min at a flow rate of 0.3 mL/min.

**Figure S113.** UV trace and corresponding MS from LC-MS analysis of purified peptide **4**. Gradient: 20-90% ACN/H_2_O with 0.1% TFA over 7 min at a flow rate of 0.3 mL/min. ESI-MS calcd.for C_385_H_633_N_105_O_118_S_3_ [M+7H]^7+^ m/z = 1246.09, [M+8H]^8+^ m/z = 1090.45, [M+9H]^9+^ m/z = 969.40, [M+10H]^10+^ m/z = 872.56, [M+11H]^11+^ m/z = 793.33, [M+12H]^12+^ m/z = 727.30, [M+13H]^13+^ m/z = 671.43, [M+14H]^14+^ m/z = 623.54, found 1245.65, 1090.88, 969.89, 872.57, 793.61, 727.36, 671.61, 623.67.

**12.6 Synthesis of 5.**

Peptide **4** (4.1 mg) was treated with TFA/EDT/TMSOTf (90/5/5, v/v/v) for 4 h and carried out as the **General procedure for Acidolysis**. Purification via preparative HPLC (20-70% ACN/H_2_O over 40 min, 0.1%TFA) followed by lyophilization afforded peptide **5** (2.3 mg, 56% yield) as white solids.

**Figure S114.** (a) Crude HPLC trace for acidolysis of the N,S-benzylidene acetal intermediate to generate peptide **5**. Gradient: 10-80% ACN/H_2_O with 0.1% TFA over 7 min at a flow rate of 0.3 mL/min. (b) UV trace of purified peptide **5**. Gradient: 10-80% ACN/H_2_O with 0.1% TFA over 7 min at a flow rate of 0.3 mL/min. (c) Corresponding MS of purified peptide **5**. ESI-MS calcd.for C_378_H_629_N_105_O_118_S_2_ [M+8H]^8+^ m/z = 1075.33, [M+9H]^9+^ m/z = 955.96, [M+10H]^10+^ m/z = 860.46, [M+11H]^11+^ m/z = 782.33, [M+12H]^12+^ m/z = 717.22, [M+13H]^13+^ m/z = 662.12, found 1075.68, 956.23, 860.87, 782.79, 717.74, 662.99.

**12.7 Synthesis of 6.**

Desulfurization of peptide **5** was carried out as the **General procedure for Desulfurization**. Peptide **5** (1.2 mg) was dissolved in 600 μL pH 4.5 buffer containing 0.5 M citrate, 6 M Gn.HCl and 0.1 M TCEP, and the freshly prepared NaBEt_4_ solution was added to the substrate solution at the final concentration of 0.1 M. Purification via preparative HPLC (20-70% ACN/H_2_O over 40 min, 0.1% TFA) followed by lyophilization afforded peptide **6** (0.7 mg, 58% yield) as white solids.

**Figure S115.** (a) Crude HPLC trace for NaBEt_4_**-**desulfurization of peptide **5**. Gradient: 10-80% ACN/H_2_O with 0.1% TFA over 7 min at a flow rate of 0.3 mL/min. (b) UV trace of purified peptide **6**. Gradient: 10-80% ACN/H_2_O with 0.1% TFA over 7 min at a flow rate of 0.3 mL/min. (c) Corresponding MS of purified peptide **6**. ESI-MS calcd.for C_378_H_629_N_105_O_118_S [M+9H]^9+^ m/z = 952.40, [M+10H]^10+^ m/z = 857.26, [M+11H]^11+^ m/z = 779.42, [M+12H]^12+^ m/z = 714.55, [M+13H]^13+^ m/z = 659.66, found 952.18, 857.58, 779.61, 714.47, 660.48.

**13. Synthesis of Hyalomin-3 (1-59)**

**13.1 Synthesis of 7.**

Hyal-3(1-37)-NHNH_2_ (**7**) was assembled according to general Fmoc-SPPS procedure (0.1 mmol). The crude peptide was purified by preparative HPLC (10-60% ACN/H_2_O over 50 min) and lyophilized to afford the Hyal-3(1-37)-NHNH_2_ (**7**) (50.2 mg, 13% yield) as a white powder.

**Figure S116.** UV trace and corresponding MS from LC-MS analysis of purified Hyal-3(1-37)-NHNH_2_ (**7**). Gradient: 10-80% ACN/H_2_O with 0.1% TFA over 7 min at a flow rate of 0.3 mL/min. ESI-MS calcd.for C_156_H_240_N_48_O_70_ [M+4H]^4+^ m/z = 977.67, found 977.89.

**13.2 Synthesis of 7a.**

Hyal-3(1-37)-CO-TSAL^SCA^ (**7a**) was obtained according to **General procedure for synthesis of model C-terminus peptide TSAL^SCA^ -esters** on 44.0 mg Hyal-3(1-37)-NHNH_2_ (**7**). The crude peptide was purified by preparative HPLC (10-60% ACN/H_2_O over 50 min) and lyophilized to afford the Hyal-3(1-37)-CO-TSAL^SCA^ (**7a**) (36.6 mg, 80% yield) as a white powder.

**Figure S117.** UV trace and corresponding MS from LC-MS analysis of purified Hyal-3(1-37)-CO-TSAL^SCA^ (**7a**). Gradient: 10-80% ACN/H_2_O with 0.1% TFA over 7 min at a flow rate of 0.3 mL/min. ESI-MS calcd.for C_164_H_245_N_49_O_71_S [M+4H]^4+^ m/z = 1018.42, found 1018.72.

**13.3 Synthesis of 8.**

Hyal-3(1-37)-CO-TSAL (**8**) was obtained according to **Pyruvic acid treatment of peptide TSAL^SCA^-esters for C-terminus peptide TSAL-esters** on 29.0 mg Hyal-3(1-37)-CO-TSAL^SCA^ (**7a**). The crude peptide was purified by preparative HPLC (10-60% ACN/H_2_O over 50 min) and lyophilized to afford the Hyal-3(1-37)-CO-TSAL (**8**) (18.0 mg, 63% yield) as a white powder.

**Figure S118.** UV trace and corresponding MS from LC-MS analysis of purified Hyal-3(1-37)-CO-TSAL (**8**). Gradient: 10-80% ACN/H_2_O with 0.1% TFA over 7 min at a flow rate of 0.3 mL/min. ESI-MS calcd.for C_164_H_245_N_49_O_71_S [M+3H]^3+^ m/z = 1338.88, [M+4H]^4+^ m/z = 1004.41, [M+5H]^5+^ m/z = 803.73, found 1338.71, 1004.43, 803.68.

**13.4 Synthesis of 9.**

Peptide **9** was assembled according to general Fmoc-SPPS procedure (0.1 mmol). The crude peptide was purified by preparative HPLC (20-60% ACN/H_2_O over 30 min) and lyophilized to afford the peptide **9** (85.1 mg, 38% yield) as a white powder.

**Figure S119.** UV trace and corresponding MS from LC-MS analysis of purified peptide **9**. Gradient: 10-80% ACN/H_2_O with 0.1% TFA over 7 min at a flow rate of 0.3 mL/min. ESI-MS calcd.for C_95_H_152_N_28_O_32_S [M+2H]^2+^ m/z = 1116.05, [M+3H]^3+^ m/z = 744.36, found 1116.23, 744.86.

**13.5 Synthesis of 10.**

The ligation between peptide **8** (10.6 mg, 2.7 μmol) and **9** (4.7 mg, 2.1 μmol) was performed as described in the **General procedure for TSAL ester-Cys/Penicillamine ligation**. Peptide **8** and **9** were dissolved in 1054 μL 0.2 M phosphate solution containing 6 M Gn∙HCl (pH 3.0) and reacted for 24 h.Purification via preparative HPLC (10-50% ACN/H_2_O over 40 min, 0.1% TFA) followed by lyophilization afforded the N,S-benzylidene acetal intermediate **10** (6.5 mg, 50% yield) as white solids.

**Figure S120.** (a-d) Crude HPLC trace for the ligation between peptide **8** and **9** in 0.2 M phosphate solution containing 6 M Gn∙HCl (pH 3.0) for 0 h, 8 h, 18 h, 30 h, respectively. gradient: 10-80% ACN/H_2_O with 0.1% TFA over 7 min at a flow rate of 0.3 mL/min. (e) Solubility of peptide **8** and **9** in different solvents. A and A′: pyridine/acetic acid (1/6, mol/mol); B and B′: 0.2 M phosphate solution containing 6 M Gn∙HCl (pH 3.0).

**Figure S121.** UV trace and corresponding MS from LC-MS analysis of purified peptide **10**. Gradient: 10-80% ACN/H_2_O with 0.1% TFA over 7 min at a flow rate of 0.3 mL/min. ESI-MS calcd.for C_258_H_392_N_74_O_102_S_2_ [M+6H]^6+^ m/z = 1038.79, [M+7H]^7+^ m/z = 890.53, found 1038.34, 890.60.

**13.6 Synthesis of 11.**

Peptide **10** (2.2 mg) was treated with TFA/EDT/TMSOTf (90/5/5, v/v/v) for 8 h and carried out as the **General procedure for Acidolysis**. Purification via preparative HPLC (10-60% ACN/H_2_O over 40 min, 0.1%TFA) followed by lyophilization afforded peptide **11** (1.6 mg, 74% yield) as white solids.

**Figure S122.** (a) Crude HPLC trace for acidolysis of the N,S-benzylidene acetal intermediate **10** to generate peptide **11**. Gradient: 10-80% ACN/H_2_O with 0.1% TFA over 10 min at a flow rate of 0.3 mL/min. (b) UV trace of purified peptide **11**. Gradient: 10-80% ACN/H_2_O with 0.1% TFA over 10 min at a flow rate of 0.3 mL/min. (c) Corresponding MS of purified peptide **11**. ESI-MS calcd.for C_251_H_388_N_74_O_102_S [M+6H]^6+^ m/z = 1018.45, [M+7H]^7+^ m/z = 873.10, [M+8H]^8+^ m/z = 764.09, found 1018.64, 873.20, 764.30.

**13.7 Synthesis of 12.**

Desulfurization of peptide **11** was carried out as the **General procedure for Desulfurization**. Peptide **11** (1.0 mg) was dissolved in 500 μL pH 4.5 buffer containing 0.5 M citrate, 6 M Gn.HCl and 0.1 M TCEP, and the freshly prepared NaBEt_4_ solution was added to the substrate solution at the final concentration of 0.1 M. Purification via preparative HPLC (10-60% ACN/H_2_O over 40 min, 0.1% TFA) followed by lyophilization afforded peptide **12** (0.7 mg, 71% yield) as white solids.

**Figure S123.** (a) Crude HPLC trace for NaBEt_4_**-**desulfurization of peptide **11**. Gradient: 10-80% ACN/H_2_O with 0.1% TFA over 7 min at a flow rate of 0.3 mL/min. (b) UV trace of purified peptide **12**. Gradient: 10-80% ACN/H_2_O with 0.1% TFA over 7 min at a flow rate of 0.3 mL/min. (c) Corresponding MS of purified peptide **12**. ESI-MS calcd.for C_251_H_388_N_74_O_102_ [M+5H]^5+^ m/z = 1215.75, [M+6H]^6+^ m/z = 1013.29, [M+7H]^7+^ m/z = 868.68, found 1215.75, 1013.33, 868.59.

**14. Synthesis of Ubiquitin-fold modifier 1 (1-83)**

**14.1 Synthesis of 13.**

UFM1(1-30)-NHNH_2_ (**13**) was assembled according to general Fmoc-SPPS procedure (0.1 mmol). The crude peptide was purified by preparative HPLC (10-60% ACN/H_2_O over 50 min) and lyophilized to afford the UFM1(1-30)-NHNH_2_ (**13**) (74.0 mg, 22% yield) as a white powder.

**Figure S124.** UV trace and corresponding MS from LC-MS analysis of purified UFM1(1-30)-NHNH_2_ (**13**). Gradient: 10-80% ACN/H_2_O with 0.1% TFA over 7 min at a flow rate of 0.3 mL/min. ESI-MS calcd.for C_155_H_252_N_38_O_44_S [M+3H]^3+^ m/z = 1128.95, [M+4H]^4+^ m/z = 846.96, [M+5H]^5+^ m/z = 677.77, found 1128.70, 846.94, 678.19.

**14.2 Synthesis of 13a.**

UFM1(1-30)-CO-TSAL^SCA^ (**13a**) was obtained according to **General procedure for synthesis of model C-terminus peptide TSAL^SCA^ -esters** on 52.0 mg UFM1(1-30)-NHNH_2_ (**13**). The crude peptide was purified by preparative HPLC (10-60% ACN/H_2_O over 50 min) and lyophilized to afford the UFM1(1-30)-CO-TSAL^SCA^ (**13a**) (32.1 mg, 59% yield) as a white powder.

**Figure S125.** UV trace and corresponding MS from LC-MS analysis of purified UFM1(1-30)-CO-TSAL^SCA^ (**13a**). Gradient: 10-80% ACN/H_2_O with 0.1% TFA over 7 min at a flow rate of 0.3 mL/min. ESI-MS calcd.for C_163_H_257_N_39_O_45_S_2_ [M+3H]^3+^ m/z = 1183.28, [M+4H]^4+^ m/z = 887.71, [M+5H]^5+^ m/z = 710.37, found 1183.23, 887.65, 710.21.

**14.3 Synthesis of 16.**

UFM1(1-30)-CO-TSAL (**16**) was obtained according to **Pyruvic acid treatment of peptide TSAL^SCA^-esters for C-terminus peptide TSAL-esters** on 31.0 mg UFM1(1-30)-CO-TSAL^SCA^ (**13a**). The crude peptide was purified by preparative HPLC (10-60% ACN/H_2_O over 50 min) and lyophilized to afford the UFM1(1-30)-CO-TSAL (**16**) (22.0 mg, 72% yield) as a white powder.

**Figure S126.** UV trace and corresponding MS from LC-MS analysis of purified UFM1(1-30)-CO-TSAL (**16**). Gradient: 10-80% ACN/H_2_O with 0.1% TFA over 7 min at a flow rate of 0.3 mL/min. ESI-MS calcd.for C_162_H_254_N_36_O_45_S_2_ [M+3H]^3+^ m/z = 1163.94, [M+4H]^4+^ m/z = 873.21, found 1163.94, 873.51.

**14.4 Synthesis of 14.**

UFM1(31-59)-CO-TSAL (**14**) was obtained according to **Direct coupling for C-terminus Gly and Pro peptide TSAL esters** on 174.0 mg fully protected crude peptide UFM1(31-59)-OH. The crude peptide was purified by preparative HPLC (20-60% ACN/H_2_O over 50 min) and lyophilized to afford the UFM1(31-59)-CO-TSAL (**14**) (10.6 mg, 8% yield) as a white powder.

**Figure S127.** UV trace and corresponding MS from LC-MS analysis of purified UFM1(31-59)-CO-TSAL (**14**). Gradient: 10-80% ACN/H_2_O with 0.1% TFA over 7 min at a flow rate of 0.3 mL/min. ESI-MS calcd.for C_143_H_221_N_33_O_41_S_2_ [M+3H]^3+^ m/z = 1041.52, [M+4H]^4+^ m/z = 781.39, found 1042.00, 781.52.

**14.5 Synthesis of 15.**

UFM1(60-83)-OH (**15)** was assembled according to general Fmoc-SPPS procedure (0.2 mmol). The crude peptide was purified by preparative HPLC (10-60% ACN/H_2_O over 50 min) and lyophilized to afford the UFM1(60-83)-OH (**15)** (44.0 mg, 8% yield) as a white powder.

**Figure S128.** UV trace and corresponding MS from LC-MS analysis of purified UFM1(60-83)-OH (**15)**. Gradient: 10-80% ACN/H_2_O with 0.1% TFA over 7 min at a flow rate of 0.3 mL/min. ESI-MS calcd.for C_115_H_192_N_38_O_33_S [M+TFA+3H]^3+^ m/z = 927.81, [M+3H]^3+^ m/z = 889.81, [M+TFA+4H]^4+^ m/z = 696.11, [M+4H]^4+^ m/z = 667.61, found 926.49, 890.10, 694.81, 667.97.

**14.6 Synthesis of 17.**

The ligation between peptide **14** (6.6 mg, 2.1 μmol) and **15** (10.3 mg, 3.9 μmol) was performed as described in the **General procedure for TSAL ester-Cys/Penicillamine ligation**. Peptide **14** and **15** were dissolved in 1056 μL 0.2 M phosphate solution containing 6 M Gn∙HCl (pH 3.0) and reacted for 48 h. After that, 8.8 mg MeONH_2_∙HCl dissolved in 106 μL 0.2 M phosphate solution containing 6 M Gn∙HCl (pH 7.0) was added and reacted for another 4 h to enable the removal of Thz group. Purification via preparative HPLC (10-50% ACN/H_2_O over 40 min, 0.1% TFA) followed by lyophilization afforded the N,S-benzylidene acetal intermediate **17** (5.9 mg, 49% yield) as white solids.

**Figure S129.** Crude HPLC trace for the ligation between peptide **14** and **15**. (a-d) ligation at t = 0 h, 24 h, 36 h, and 48 h, respectively. gradient: 20-70% ACN/H_2_O with 0.1% TFA over 10 min at a flow rate of 0.3 mL/min. (e) MeONH_2_∙HCl was added to remove Thz group. Gradient: 20-70% ACN/H_2_O with 0.1% TFA over 10 min at a flow rate of 0.3 mL/min.

**Figure S130.** UV trace and corresponding MS from LC-MS analysis of purified peptide **17**. Gradient: 20-70% ACN/H_2_O with 0.1% TFA over 10 min at a flow rate of 0.3 mL/min. ESI-MS calcd.for C_257_H_411_N_71_O_73_S_3_ [M+4H]^4+^ m/z = 1440.75, [M+5H]^5+^ m/z = 1152.80, [M+6H]^6+^ m/z = 960.83, [M+7H]^7+^ m/z = 823.71, [M+8H]^8+^ m/z = 720.87, [M+9H]^9+^ m/z = 640.89, found 1441.09,1152.95, 960.94, 823.80, 721.05, 641.01

**14.7 Synthesis of 18.**

The ligation between peptide **16** (5.9 mg, 1.7 μmol) and **17** (4.1 mg, 0.7 μmol) was performed as described in the **General procedure for TSAL ester-Cys/Penicillamine ligation**. Peptide **16** and **17** were dissolved in 714 μL 0.2 M phosphate solution containing 6 M Gn∙HCl (pH 3.0) and reacted for 42 h. Purification via preparative HPLC (10-50% ACN/H_2_O over 50 min, 0.1% TFA) followed by lyophilization afforded the N,S-benzylidene acetal intermediate **18** (1.5 mg, 23% yield) as white solids.

**Figure S131.** Crude HPLC trace for the ligation between peptide **16** and **17**. (a-c) ligation at t = 0 h, 24 h, and 42 h, respectively. gradient: 20-60% ACN/H_2_O with 0.1% TFA over 10 min at a flow rate of 0.3 mL/min.

**Figure S132.** UV trace and corresponding MS from LC-MS analysis of purified peptide **18**. Gradient: 20-60% ACN/H_2_O with 0.1% TFA over 10 min at a flow rate of 0.3 mL/min. ESI-MS calcd.for C_419_H_663_N_107_O_117_S_5_ [M+9H]^9+^ m/z = 1026.53, [M+10H]^10+^ m/z = 923.98, [M+11H]^11+^ m/z = 840.07, [M+12H]^12+^ m/z = 770.15, [M+13H]^13+^ m/z = 710.98, [M+14H]^14+^ m/z = 660.27, found 1026.57, 924.26, 840.34, 770.40, 711.38, 660.48.

**14.8 Synthesis of 19.**

Peptide **18** (1.5 mg) was treated with TFA/EDT/TMSOTf (90/5/5, v/v/v) for 4 h and carried out as the **General procedure for Acidolysis**. Purification via preparative HPLC (10-60% ACN/H_2_O over 40 min, 0.1%TFA) followed by lyophilization afforded peptide **19** (1.0 mg, 68% yield) as white solids.

**Figure S133.** (a) Crude HPLC trace for acidolysis of the N,S-benzylidene acetal intermediate **18** to generate peptide **19**. Gradient: 5-95% ACN/H_2_O with 0.1% TFA over 7 min at a flow rate of 0.3 mL/min. (b) UV trace of purified peptide **19**. Gradient: 5-95% ACN/H_2_O with 0.1% TFA over 7 min at a flow rate of 0.3 mL/min. (c) Corresponding MS of purified peptide **19**. ESI-MS calcd.for C_405_H_655_N_107_O_117_S_3_ [M+8H]^8+^ m/z = 1124.72, [M+9H]^9+^ m/z = 999.87, [M+10H]^10+^ m/z = 899.98, [M+11H]^11+^ m/z = 818.25, [M+12H]^12+^ m/z = 750.15, [M+13H]^13+^ m/z = 692.52, [M+14H]^14+^ m/z = 643.13, found 1125.00, 1000.18, 900.27, 818.70, 750.55, 692.71, 643.55.

**14.9 Synthesis of 20.**

Desulfurization of peptide **19** was carried out as the **General procedure for Desulfurization**. Peptide **19** (0.9 mg) was dissolved in 450 μL pH 4.5 buffer containing 0.5 M citrate, 6 M Gn.HCl and 0.1 M TCEP, and the freshly prepared NaBEt_4_ solution was added to the substrate solution at the final concentration of 0.1 M. Purification via preparative HPLC (10-60% ACN/H_2_O over 40 min, 0.1% TFA) followed by lyophilization afforded peptide **20** (0.5 mg, 56% yield) as white solids.

**Figure S134.** (a) Crude HPLC trace for NaBEt_4_**-**desulfurization of peptide **19**. Gradient: 10-80% ACN/H_2_O with 0.1% TFA over 7 min at a flow rate of 0.3 mL/min. (b) UV trace of purified peptide **20**. Gradient: 10-80% ACN/H_2_O with 0.1% TFA over 7 min at a flow rate of 0.3 mL/min. (c) Corresponding MS of purified peptide **20**. ESI-MS calcd.for C_405_H_655_N_107_O_117_S [M+8H]^8+^ m/z = 1116.73, [M+9H]^9+^ m/z = 992.76, [M+10H]^10+^ m/z = 893.58, [M+11H]^11+^ m/z = 812.44, [M+12H]^12+^ m/z = 744.82, found 1116.78, 993.02, 893.74, 812.85, 745.09.

**15. Synthesis of *Mtb* Chorismate Mutase (1-83)**

**15.1 Synthesis of 21.**

*Mtb* CM(1-22)-NHNH_2_ (**21**) was assembled according to general Fmoc-SPPS procedure (0.1 mmol). The crude peptide was purified by preparative HPLC (10-60% ACN/H_2_O over 50 min) and lyophilized to afford the *Mtb* CM(1-22)-NHNH_2_ (**21**) (34.2 mg, 13% yield) as a white powder.

**Figure S135.** UV trace and corresponding MS from LC-MS analysis of purified *Mtb* CM(1-22)-NHNH_2_ (**21**). Gradient: 5-95% ACN/H_2_O with 0.1% TFA over 10 min at a flow rate of 0.3 mL/min. ESI-MS calcd.for C_110_H_185_N_31_O_39_ [M+2H]^2+^ m/z = 1283.68, [M+3H]^3+^ m/z = 856.12, [M+4H]^4+^ m/z = 642.34, found 1283.33, 856.30, 642.83.

**15.2 Synthesis of 21a.**

*Mtb* CM(1-22)-CO-TSAL^SCA^ (**21a**) was obtained according to **General procedure for synthesis of model C-terminus peptide TSAL^SCA^ -esters** on 26.5 mg *Mtb* CM(1-22)-NHNH_2_ (**21**). The crude peptide was purified by preparative HPLC (10-60% ACN/H_2_O over 50 min) and lyophilized to afford the *Mtb* CM(1-22)-CO-TSAL^SCA^ (2**1a**) (14.0 mg, 50% yield) as a white powder.

**Figure S136.** UV trace and corresponding MS from LC-MS analysis of purified *Mtb* CM(1-22)-CO-TSAL^SCA^ (2**1a**). Gradient: 5-95% ACN/H_2_O with 0.1% TFA over 7 min at a flow rate of 0.3 mL/min. ESI-MS calcd.for C_118_H_190_N_32_O_40_S [M+2H]^2+^ m/z = 1365.18, [M+3H]^3+^ m/z = 910.45, [M+4H]^4+^ m/z = 683.09, found 1365.23, 910.68, 683.60.

**15.3 Synthesis of 25.**

*Mtb* CM(1-22)-CO-TSAL (**25**) was obtained according to **Pyruvic acid treatment of peptide TSAL^SCA^-esters for C-terminus peptide TSAL-esters** on 31.4 mg *Mtb* CM(1-22)-CO-TSAL^SCA^ (2**1a**). The crude peptide was purified by preparative HPLC (10-60% ACN/H_2_O over 60 min) and lyophilized to afford the *Mtb* CM(1-22)-CO-TSAL (2**5**) (21.1 mg, 50% yield) as a white powder.

**Figure S137.** UV trace and corresponding MS from LC-MS analysis of purified *Mtb* CM(1-22)-CO-TSAL (2**5**). Gradient: 10-80% ACN/H_2_O with 0.1% TFA over 7 min at a flow rate of 0.3 mL/min. ESI-MS calcd.for C_117_H_187_N_29_O_40_S [M+2H]^2+^ m/z = 1336.66, [M+3H]^3+^ m/z = 891.44, found 1336.48, 891.73.

**15.4 Synthesis of 22.**

The fully protected crude peptide Thz-*Mtb* CM(24-44)-OH (216 mg, 0.06 mmol) was dissolved in 6 mL dry DMF, DIEA (60 μL, 0.36 mmol) and benzotriazol-1-yl-oxytripyrrolidinophosphonium hexafluorophosphate (PyBOP) (94 mg, 0.18 mmol) were added, and α, α-dimethoxy-salicylaldehyde (504 mg, 3.00 mmol) was finally added. The reaction mixture is stirred at room temperature for overnight. After that, the solvent was removed under reduced pressure and the resulting residue was treated with TFA/H_2_O (95:5, v/v). After global deprotection for 3 h, TFA was blown off and the oily residue was triturated with diethyl ether and centrifuged. The precipitate was pelleted and the ether was subsequently decanted. The resulting solid was purified by HPLC and lyophilization to give the Thz-*Mtb* CM(24-44)-SAL (**22**) (63.4 mg, 44% yield) as white solid.

**Figure S138.** UV trace and corresponding MS from LC-MS analysis of purified Thz-*Mtb* CM(24-44)-SAL (**22**). Gradient: 10-80% ACN/H_2_O with 0.1% TFA over 5 min at a flow rate of 0.3 mL/min. ESI-MS calcd.for C_103_H_178_N_34_O_28_S_2_ [M+3H]^3+^ m/z = 802.43, [M+4H]^4+^ m/z = 602.08, found 802.85, 602.41.

**15.5 Synthesis of 23.**

*Mtb* CM(45-69)-NHNH_2_ (**23**) was assembled according to general Fmoc-SPPS procedure (0.15 mmol). The crude peptide was purified by preparative HPLC (20-60% ACN/H_2_O over 50 min) and lyophilized to afford the *Mtb* CM(45-69)-NHNH_2_ (**23**) (71.6 mg, 16% yield) as a white powder.

**Figure S139.** UV trace and corresponding MS from LC-MS analysis of purified *Mtb* CM(45-69)-NHNH_2_ (**23**). Gradient: 10-80% ACN/H_2_O with 0.1% TFA over 7 min at a flow rate of 0.3 mL/min. ESI-MS calcd.for C_126_H_212_N_40_O_39_S [M+2H]^2+^ m/z = 1472.28, [M+3H]^3+^ m/z = 981.85, [M+4H]^4+^ m/z = 736.64, [M+5H]^5+^ m/z = 589.51, found 1473.61, 982.39, 737.19, 589.91.

**15.6 Synthesis of 26.**

*Mtb* CM(45-69)-CO-TSAL^SCA^ (**26**) was obtained according to **General procedure for synthesis of model C-terminus peptide TSAL^SCA^ -esters** on 36.0 mg *Mtb* CM(45-69)-NHNH_2_ (**23**). The crude peptide was purified by preparative HPLC (10-60% ACN/H_2_O over 50 min) and lyophilized to afford the *Mtb* CM(45-69)-CO-TSAL^SCA^ (**26**) (17.5 mg, 46% yield) as a white powder.

**Figure S140.** UV trace and corresponding MS from LC-MS analysis of purified *Mtb* CM(45-69)-CO-TSAL^SCA^ (**26**). Gradient: 10-80% ACN/H_2_O with 0.1% TFA over 7 min at a flow rate of 0.3 mL/min. ESI-MS calcd.for C_134_H_217_N_41_O_40_S_2_ [M+3H]^3+^ m/z = 1036.19, [M+4H]^4+^ m/z = 777.39, [M+5H]^5+^ m/z = 622.11, found 1036.88, 777.80, 622.62.

**15.7 Synthesis of 27.**

Thz-*Mtb* CM(24-44)-SAL (**22**) dissolved in DMSO (8.1 mg, 3.4 μmol) and *Mtb* CM(45-69)-CO-TSAL^SCA^ (**26**) dissolved in DMSO (7.5 mg, 2.4 μmol) were added to 1208 μL pyridine/acetic acid (1/1, mol/mol) and reacted for 6 h at room temperature. After that, the solvent was blown off and the residue was triturated with diethyl ether and centrifuged. The precipitate is pelleted and the ether is subsequently decanted. The resulting solid was purified by HPLC (20-60% ACN/H_2_O over 50 min, 0.1% TFA) and then lyophilization to give the N,O-benzylidene acetal intermediate peptide **27** (5.7 mg, 43% yield) as white solid.

**Figure S141.** (a) Crude HPLC trace for STL ligation between peptide **22** and **26** at t = 0 h. Gradient: 20-70% ACN/H_2_O with 0.1% formic acid (FA) over 10 min at a flow rate of 0.3 mL/min. (b) Crude HPLC trace for STL ligation between peptide **22** and **26** at t = 5 h. Gradient: 20-70% ACN/H_2_O with 0.1% FA over 10 min at a flow rate of 0.3 mL/min. (c) UV trace of purified peptide **27**. Gradient: 20-70% ACN/H_2_O with 0.1% FA over 10 min at a flow rate of 0.3 mL/min. (d) Corresponding MS of purified peptide **27**. ESI-MS calcd.for C_237_H_393_N_75_O_67_S_4_ [M+6H]^6+^ m/z = 916.48, [M+7H]^7+^ m/z = 785.69, [M+8H]^8+^ m/z = 687.61, [M+9H]^9+^ m/z = 611.32, found 916.84, 786.18, 688.30, 612.13.

**5.8 Synthesis of 28.**

Thz-*Mtb* CM(24-69)-CO-TSAL (**28**) was obtained according to **Pyruvic acid treatment of peptide TSAL^SCA^-esters for C-terminus peptide TSAL-esters** on 33.6 mg peptide **27**. The crude peptide was purified by preparative HPLC (10-60% ACN/H_2_O over 50 min) and lyophilized to afford the Thz-*Mtb* CM(24-69)-CO-TSAL (**28**) (19.0 mg, 58% yield) as a white powder.

**Figure S142.** UV trace and corresponding MS from LC-MS analysis of purified Thz-*Mtb* CM(24-69)-CO-TSAL (**28**). Gradient: 20-70% ACN/H_2_O with 0.1% FA over 10 min at a flow rate of 0.3 mL/min. ESI-MS calcd.for C_237_H_393_N_75_O_67_S_4_ [M+5H]^5+^ m/z = 1067.36, [M+6H]^6+^ m/z = 889.63, [M+7H]^7+^ m/z = 762.69, [M+8H]^8+^ m/z = 667.48, [M+9H]^9+^ m/z = 593.42, found 1067.54, 890.02, 763.35, 667.91, 593.57.

**15.9 Synthesis of 24.**

*Mtb* CM(70-83)-OH (**24**) was assembled according to general Fmoc-SPPS procedure (0.1 mmol). The crude peptide was purified by preparative HPLC (10-60% ACN/H_2_O over 30 min) and lyophilized to afford the *Mtb* CM(70-83)-OH (**24**) (30.0 mg, 19% yield) as a white powder.

**Figure S143.** UV trace and corresponding MS from LC-MS analysis of purified *Mtb* CM(70-83)-OH (**24**). Gradient: 5-95% ACN/H_2_O with 0.1% TFA over 10 min at a flow rate of 0.3 mL/min. ESI-MS calcd.for C_69_H_125_N_25_O_15_S [M+2H]^2+^ m/z = 788.98, [M+3H]^3+^ m/z = 526.32, found 789.72, 527.01.

**15.10 Synthesis of 29.**

The ligation between peptide **28** (3.8 mg, 0.7 μmol) and **24** (4.3 mg, 2.7 μmol) was performed as described in the **General procedure for TSAL ester-Cys/Penicillamine ligation**. Peptide **28** and **24** were dissolved in 357 μL 0.2 M phosphate solution containing 6 M Gn∙HCl (pH 3.0) and reacted for 30 h. After that, 3.0 mg MeONH_2_∙HCl dissolved in 36 μL 0.2 M phosphate solution containing 6 M Gn∙HCl (pH 7.0) was added and reacted for another 4h to enable the removal of Thz group. Purification via preparative HPLC (10-50% ACN/H_2_O over 60 min, 0.1% TFA) followed by lyophilization afforded the N,S-benzylidene acetal intermediate **29** (3.1 mg, 63% yield) as white solids.

**Figure S144.** Crude HPLC trace for the ligation between peptide **28** and **24**. (a) ligation at t = 0 h, gradient: 20-70% ACN/H_2_O with 0.1% FA over 10 min at a flow rate of 0.3 mL/min. (b) ligation at t = 30 h, gradient: 20-70% ACN/H_2_O with 0.1% FA over 10 min at a flow rate of 0.3 mL/min. (c) MeONH_2_∙HCl was added to remove Thz group. Gradient: 20-70% ACN/H_2_O with 0.1% TFA over 10 min at a flow rate of 0.3 mL/min.

**Figure S145.** UV trace and corresponding MS from LC-MS analysis of purified peptide **29**. Gradient: 20-70% ACN/H_2_O with 0.1% FA over 10 min at a flow rate of 0.3 mL/min. ESI-MS calcd.for C_298_H_509_N_97_O_80_S_5_ [M+7H]^7+^ m/z = 985.25, [M+8H]^8+^ m/z = 862.22, [M+9H]^9+^ m/z = 766.53, [M+10H]^10+^ m/z = 689.97, [M+11H]^11+^ m/z = 627.34, [M+12H]^12+^ m/z = 575.15, found 983.95, 861.36, 765.88, 689.72, 627.36, 575.22.

**15.11 Synthesis of 30.**

The ligation between peptide **25** (2.5 mg, 0.9 μmol) and **29** (3.1 mg, 0.5 μmol) was performed as described in the **General procedure for TSAL ester-Cys/Penicillamine ligation**. Peptide **25** and **29** were dissolved in 902 μL 0.2 M phosphate solution containing 6 M Gn∙HCl (pH 3.0) and reacted for 27 h. Purification via preparative HPLC (10-50% ACN/H_2_O over 60 min, 0.1% TFA) followed by lyophilization afforded the N,S-benzylidene acetal intermediate **30** (2.0 mg, 47% yield) as white solids.

**Figure S146.** Crude HPLC trace for the ligation between peptide **25** and **29**. (a) ligation at t = 0 h, gradient: 20-70% ACN/H_2_O with 0.1% FA over 10 min at a flow rate of 0.3 mL/min. (b) ligation at t = 27 h, gradient: 20-70% ACN/H_2_O with 0.1% FA over 10 min at a flow rate of 0.3 mL/min.

**Figure S147.** UV trace and corresponding MS from LC-MS analysis of purified peptide **30**. Gradient: 20-70% ACN/H_2_O with 0.1% FA over 10 min at a flow rate of 0.3 mL/min. ESI-MS calcd.for C_414_H_694_N_126_O_119_S_6_ [M+10H]^10+^ m/z = 954.11, [M+11H]^11+^ m/z = 867.46, [M+12H]^12+^ m/z = 795.26, [M+13H]^13+^ m/z = 734.16, [M+14H]^14+^ m/z = 681.79, [M+15H]^15+^ m/z = 636.40, [M+16H]^16+^ m/z = 596.69, [M+17H]^17+^ m/z = 561.65, found 954.04, 867.21, 795.64, 734.74, 682.35, 636.91, 596.95, 561.25.

**15.12 Synthesis of 31.**

Peptide **30** (4.4 mg) was treated with TFA/EDT/TMSOTf (90/5/5, v/v/v) for 8 h and carried out as the **General procedure for Acidolysis**. Purification via preparative HPLC (10-60% ACN/H_2_O over 40 min, 0.1%TFA) followed by lyophilization afforded peptide **31** (2.3 mg, 54% yield) as white solids.

**Figure S148.** (a) Crude HPLC trace for acidolysis of the N,S-benzylidene acetal intermediate **30** to generate peptide **31**. Gradient: 10-80% ACN/H_2_O with 0.1% FA over 7 min at a flow rate of 0.3 mL/min. (b) UV trace of purified peptide **31**. Gradient: 10-80% ACN/H_2_O with 0.1% FA over 7 min at a flow rate of 0.3 mL/min. (c) Corresponding MS of purified peptide **31**. ESI-MS calcd.for C_400_H_686_N_126_O_119_S_4_ [M+10H]^10+^ m/z = 930.11, [M+11H]^11+^ m/z = 845.64, [M+12H]^12+^ m/z = 775.25, [M+13H]^13+^ m/z = 715.70, [M+14H]^14+^ m/z = 664.65, [M+15H]^15+^ m/z = 620.40, [M+16H]^16+^ m/z = 581.69, found 930.73, 845.88, 775.95, 715.99, 664.84, 620.97, 582.19.

**15.13 Synthesis of 32.**

Desulfurization of peptide **31** was carried out as the **General procedure for Desulfurization**. Peptide **31** (2.3 mg) was dissolved in 1000 μL pH 4.5 buffer containing 0.5 M citrate, 6 M Gn.HCl and 0.1 M TCEP, and the freshly prepared NaBEt_4_ solution was added to the substrate solution at the final concentration of 0.1 M. Purification via preparative HPLC (10-60% ACN/H_2_O over 50 min, 0.1% TFA) followed by lyophilization afforded peptide **32** (1.6 mg, 70% yield) as white solids.

**Figure S149.** (a) Crude HPLC trace for NaBEt_4_**-**desulfurization of peptide **31**. Gradient: 10-80% ACN/H_2_O with 0.1% FA over 7 min at a flow rate of 0.3 mL/min. (b) UV trace of purified peptide **32**. Gradient: 10-80% ACN/H_2_O with 0.1% FA over 7 min at a flow rate of 0.3 mL/min. (c) Corresponding MS of purified peptide **32**. ESI-MS calcd.for C_400_H_686_N_126_O_119_S_2_ [M+10H]^10+^ m/z = 923.71, [M+11H]^11+^ m/z = 839.83, [M+12H]^12+^ m/z = 769.93, [M+13H]^13+^ m/z = 710.78, [M+14H]^14+^ m/z = 660.08, found 923.93, 840.27, 769.96, 711.46, 660.88.

**16. Synthesis of thiol-salicylaldehyde semicarbazone**

16.1 Synthesis of (2Z,2'E)-2,2'-((disulfanediylbis(2,1-phenylene))bis(methaneylylidene))bis (hydrazine-1-carboxamide) (**33**)

2,2'-dithiodibenzaldehyde was synthesized according to previous method^3^. In a flat-bottomed flask, 2,2'-dithiodibenzaldehyde (1.39 g, 5.07 mmol) and semicarbazide hydrochloride (1.70 g, 15.22 mmol) were dissolved in anhydrous ethanol (25 mL). The mixture was stirred under reflux reaction at 100°C for 2 h. Then the mixture was filtered and washed with ethanol (3ⅹ10 mL), water (3ⅹ10 mL), and Et_2_O (3ⅹ10 mL). After drying under vacuum, **33** was obtained as a white solid (1.75 g, 89%) and directly used in the next step without further purification.

16.2 Synthesis of (Z)-2-(2-mercaptobenzylidene)hydrazine-1-carboxamide (**34**)

In a flat-bottomed flask, a solution of **33** (1.75 g, 4.50 mmol) was dissolved in dry MeOH (10 mL). TCEP∙HCl (1.94g, 6.75 mmol) and Et_3_N (940 μL, 6.75 mmol) were added to the solution. The mixture was stirred at room temperature for 1 h. Then the reaction mixture was filtered and washed with MeOH (3ⅹ10 mL), water (3ⅹ10 mL), and Et_2_O (3ⅹ10 mL). After drying under vacuum, **34** was obtained as a white solid (1.31 g, 75%). ^1^H NMR (400 MHz, DMSO) δ 10.49 (s, 1H), 8.28 (s, 1H), 8.03–7.87 (m, 1H), 7.70–7.50 (m, 1H), 7.41–7.25 (m, 2H), 6.41 (s, 2H), 5.51 (s, 1H).^13^C NMR (151 MHz, DMSO-*d*6) δ 156.12, 137.48, 133.50, 131.37, 130.59, 128.69, 127.61, 124.93. MS (ESI) : m/z calculated for C_8_H_9_N_3_OS [M+H]^+^:196.05, found:196.08.

**^1^H and ^13^C NMR spectra**

**17. References**

1. Huang, Y.; Chen, C.; Li, S.; Gao, S.;Shi, J.; Li, Y. Facile synthesis of C-terminal peptide hydrazide and thioester of NY-ESO-1 (A39-A68) from an Fmoc-hydrazine 2-chlorotrityl chloride resin. *Tetrahedron*. **2014**, *70* (18), 2951−2955.

2. Sun, Z.; Ma, W.; Cao, Y.; Wei, T.; Chow, H.Y.; Tan, Y.; Cheung, C.H.P.; Liu, J.; Lee, H.K.; Liu, H.; Li, X. Superfast Desulfurization for Protein Chemical Synthesis and Modification. *Chem.* **2022**, *8* (9), 2542−2557.

3. Tung, C. L.; Wong, C. T. T.; Li, X. Peptide 2-formylthiophenol esters do not proceed through a Ser/Thr ligation pathway, but participate in a peptide aminolysis to enable peptide condensation and cyclization. *Org. Biomol. Chem.* **2015**, *13* (25), 6922−6926.
